# Modular Synthesis of Furans with up to Four Different Substituents by a *trans*‐Carboboration Strategy

**DOI:** 10.1002/anie.202005560

**Published:** 2020-06-03

**Authors:** Hongming Jin, Alois Fürstner

**Affiliations:** ^1^ Max-Planck-Institut für Kohlenforschung 45470 Mülheim/Ruhr Germany

**Keywords:** carboboration, cascade reactions, cross coupling, furans, *trans*-addition

## Abstract

Propargyl alcohols, on treatment with MHMDS (M=Na, K), B_2_(pin)_2_, an acid chloride and a palladium/copper co‐catalyst system, undergo a reaction cascade comprised of *trans*‐diboration, regioselective acylation, cyclization and dehydration to give trisubstituted furylboronic acid pinacol ester derivatives in good yields; subsequent Suzuki coupling allows a fourth substituent of choice to be introduced and hence tetrasubstituted (arylated) furans to be formed. In terms of modularity, the method seems unrivaled, not least because each product can be attained by two orthogonal but convergent ways (“diagonal split”). This asset is illustrated by the “serial” formation of a “library” of all twelve possible furan isomers that result from systematic permutation of four different substituents about the heterocyclic core.

## Graphical abstract

**As you like it**: Propargyl alcohols can be transformed in a single step into 3‐borylated‐2,4,5‐triarylated furans by an efficient *trans*‐carboboration/cyclization/dehydration cascade. A subsequent Suzuki coupling allows a fourth substituent of choice to be introduced. The modularity of the method is unrivaled in that it allows four different substituents to be introduced in a programmable manner and hence all isomers of the multiply arylated furan to be attained.

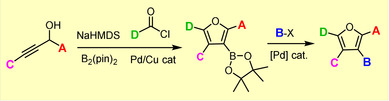

Highly arylated (hetero)arenes are privileged motifs in material science that find numerous applications, not least in optoelectronic devices or as organic field‐effect transistors to mention but a few.^1^ Since their physical properties are not only determined by the nature of the peripheral aryl groups but also by their order, programmable approaches to such compounds are in high demand. The structural diversity and hence the synthetic challenge increase massively with the number of different substituents attached to the core: thus, permutation of four different groups about a five‐membered heterocyclic ring leads to no less than 12 regioisomers (Figure 1). While the preparation of any such “library” in meaningful amounts is a non‐trivial task, the furan series (X=O) poses particular challenges. Although many methods for the preparation of highly substituted furans are documented in the literature,^2^, ^3^, ^4^, ^5^, ^6^ few of them—if any—will provide access to an *entire* such tableau. The problem is partly rooted in the fact that furans—unlike thiophenes and many other heterocycles^7^—are rather refractory to multiple regioselective substitution or C−H activation reactions.^8^, ^9^ The increasing awareness of the peculiar physical properties of polyarylated furans, however, fuels the demand,^10^ in addition to their well‐established but more traditional role as building blocks in organic synthesis and/or as pharmacophores.^11^, ^12^ The recent discovery of the intriguing mechanochromic properties of tetraarylfurans and derived ring‐opened products provides an instructive example:^13^ the fluorescence emission as response to external mechanical forces (grinding, shearing etc.) critically depends on the packing mode in the solid state and hence on the nature as well as on the order of the substituents about the ring. Any systematic exploration of structure/property relationships mandates modular and efficient synthesis routes.

**Figure 1:**
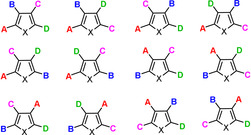
Permutation of four different substituents about a five‐membered heterocyclic ring leads to 12 regioisomers.

We saw an opportunity to meet the challenge by a conceptually new strategy based on the net *trans*‐carboboration of propargyl alcohols. As previously communicated, this unorthodox transformation is readily accomplished on treatment of a substrate of type **A** with B_2_(pin)_2_, R^3^X, a palladium catalyst and a base, preferentially NaHMDS (Scheme 1).^14^ The reaction is thought to proceed via a bis‐borylated intermediate of type **B**,^15^ which succumbs to cross‐coupling exclusively at the endocyclic borate site as long as no external base is added to the mixture; the exocyclic borane moiety subsists. This mechanism accounts for the observed regio‐ and stereoselectivity and explains why different electrophiles R^3^‐X (R^3^=aryl‐, allyl‐, benzyl‐, methyl‐, alkynyl) participate well in the addition process to give the desired products **C** in good yield and outstanding *trans*‐selectivity.^14^, ^16^, ^17^

**Scheme 1:**
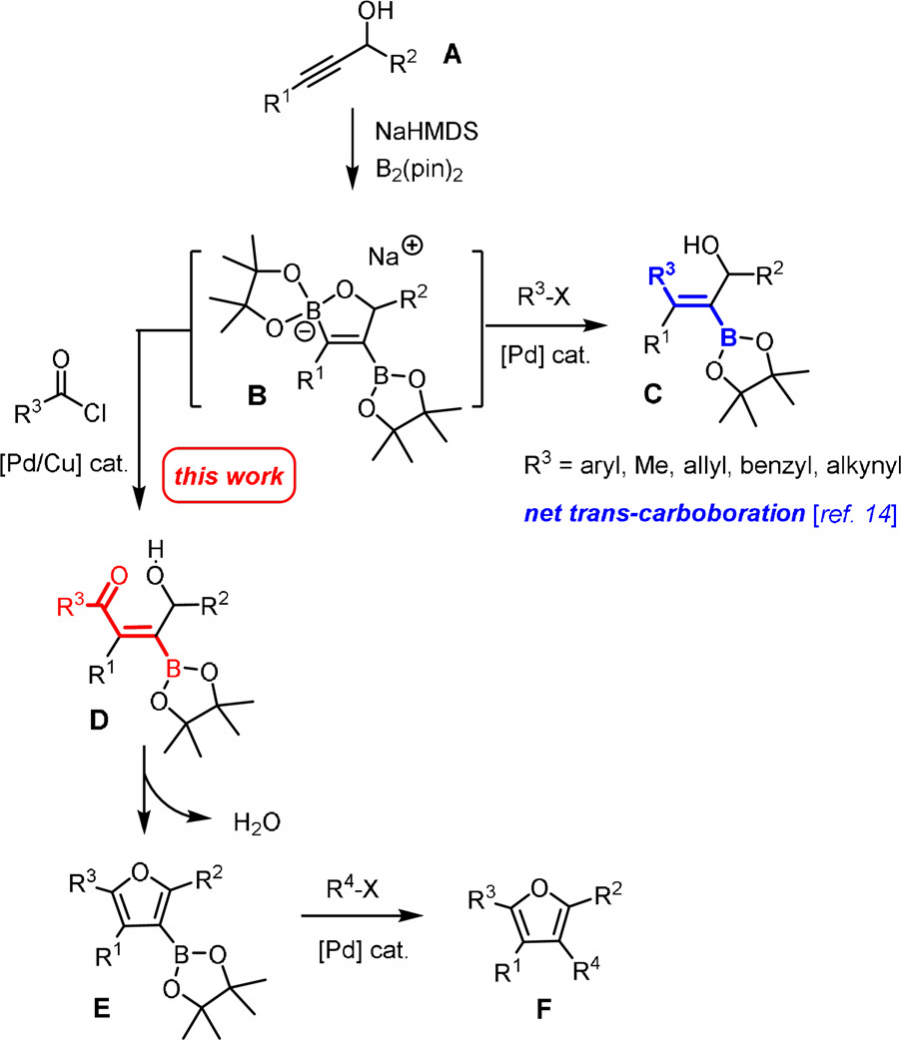
Extension of the net *trans*‐carboboration of propargyl alcohols to the formation of tetrasubstituted furans.

We reasoned that this methodology can be re‐programmed into an uniquely flexible and expedient synthesis of furylboronic acid derivatives **E** and (tetra)substituted furans **F** by using an acid chloride as the electrophilic partner. The selected screening results compiled in Table 1 prove that this is indeed the case: regioselective *trans*‐acylboration of **1 a** is followed by spontaneous cyclization and dehydration with formation of the 3‐borylated furan **2 a**. In accordance with our previous observations,^14^ the choice of base for the deprotonation of the propargyl alcohol substrate is arguably the most critical parameter: NaHMDS and KHMDS proved similarly effective, whereas *n*BuLi and LiHMDS basically failed to afford the desired product.^18^ This outcome is partly ascribed to the fact that the stability of borate complexes in solution—and hence of the key intermediates of type **B**—is known to be cation‐dependent, in the rough order K^+^ ≈ Na^+^ > Li^+^ ≫ MgX^+^, ZnX^+^.^19^ However, the released hexamethyldisilazane also seems to play an important but as yet not fully understood role, since other sodium bases such as NaH and NaO*t*Bu were found to be much less suitable than NaHMDS. The yield of **2 a** was further improved upon supplementing the mixture with CuTC^20^ and a P‐based ligand, preferentially P(OPh)_3_: the derived copper catalyst supposedly promotes the actual acylation step. Under these conditions, compound **2 a** was obtained in 69 % yield after flash chromatography.

**Table 1:** Reaction optimization: selected screening data.^[a]^ 
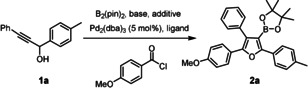

Entry	Base	Ligand (20 mol %)	Additive (15 mol %)	*T* [°C]	Yield [%]^[b]^
1	*n*‐BuLi	P(2‐furyl)_3_	–	75	0
2	LiHMDS	P(2‐furyl)_3_	–	75	<5
3	NaO*t*Bu	P(2‐furyl)_3_	–	75	0
4	NaH	P(2‐furyl)_3_	–	75	12
5	NaHMDS	P(2‐furyl)_3_	—	75	61
6	KHMDS	P(2‐furyl)_3_	–	75	63
7	KHMDS	P(2‐furyl)_3_	CuTC	RT	69
8	KHMDS	PPh_3_	CuTC	RT	72
9	KHMDS	P(*o*‐tol)_3_	CuTC	RT	59
10	KHMDS	P(OPh)_3_	CuTC	RT	75 (69)

[a] All reactions were performed in 1,4‐dioxane; [b] NMR yield (isolated yield); TC=thiophene‐2‐carboxylate.

This convenient reaction cascade^21^ works well on (multi)gram scale as illustrated by the formation of **4 a** (Scheme 2). As expected, the subsequent Suzuki coupling proceeded smoothly at ambient temperature when [(dppf)PdCl_2_] was used as the catalyst and aqueous KOH as the promotor.^22^ Furan **5 a** thus formed in only two operations also shows that poly‐functionalized products can be reached that promise a rich downstream chemistry.

**Scheme 2:**
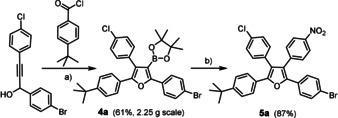
Upscaling Experiment: a) (i) KHMDS, 1,4‐dioxane, 0 °C; (ii) B_2_(pin)_2_, *t*BuC_6_H_4_C(O)Cl, Pd_2_(dba)_3_ (5 mol %), CuTC (15 mol %), P(OPh)_3_ (20 mol %), RT, 61 %; b) 1‐iodo‐4‐nitrobenzene, [(dppf)PdCl_2_] (5 mol %), aq. KOH, THF, RT, 87 %; dba=dibenzylideneacetone; dppf=1,1′‐bis(diphenylphosphino)‐ferrocene; KHMDS potassium hexamethyldisilazide; RT=room temperature.

At the strategy level, the new method combines several assets: first, the formation of a tetra‐substituted furan requires only simple components: a terminal alkyne and an aldehyde to give the propargyl alcohol substrate, an acid chloride, and an aryl halide. Secondly, the deconvolution into the four constituents is possible along two orthogonal ways (Scheme 3): this “diagonal split” enriches the pool of building blocks from which to choose and hence allows additional criteria to be accounted for such as the availability, stability, cost etc. of the required building blocks. Yet another valuable dividend becomes apparent if the concept of “diagonal split” is systematically applied to an entire “library” of regioisomeric furans as shown in Figure 1. Eight different propargyl alcohols are required to prepare such a comprehensive compound collection if one were to pursue only the disconnections color‐coded in red (or, alternatively, in blue). The logic of “diagonal split” reduces the number of mandatory propargyl alcohols to six while increasing the number of candidates to twelve, from which one can choose those substrates that are most readily available (for the full analysis, see the SI).

**Scheme 3:**
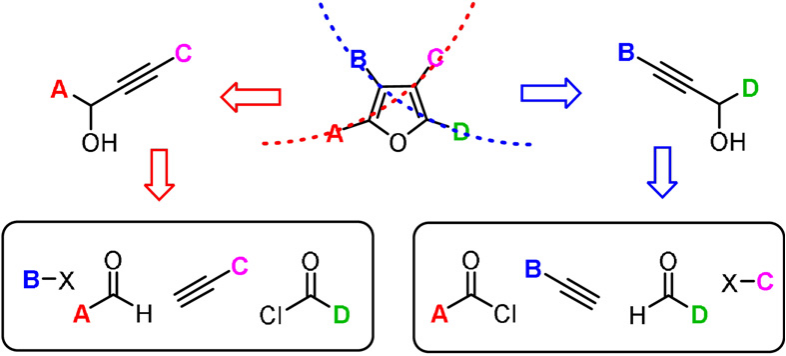
The concept of “diagonal split” spelled out for a specific tetra‐substituted furan isomer; the application to a generic “library” of the 12 possible regioisomeric furans formed upon permutation of four different substituents about the heterocyclic core is contained in the SI.

From the conceptual viewpoint, “diagonal split” hence reduces the workload while increasing the flexibility. The latter aspect is illustrated by the fact that we deliberately chose only three (rather than four) different aldehydes, yet were able to obtain all possible 12 tetraarylated furan isomers **3 a**–**3 l** depicted in Figure 2; it is important to note that the specific choice (PhCHO, TolCHO, *t*BuC_6_H_4_CHO) is only one of several possibilities to reach the goal. From the practical perspective it is noteworthy that the one‐pot *trans*‐carboboration/cyclization/dehydration cascade worked uniformly well in all cases, with yields of the different borylated furans **2 a**–**2 l** ranging from 60–72 %; the subsequent cross coupling events to give the final “library” were invariably high yielding, many of them close to quantitative. Since both transformations can be carried out in a “serial” manner, the overall effort necessary for the preparation of this unique isomer collection was perfectly manageable.

**Figure 2:**
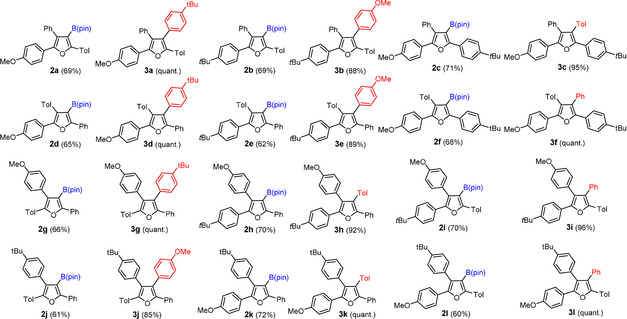
An ensemble of all possible furan isomers formed upon permutation of four different aryl substituents in the periphery; furan formation and the cross coupling reactions transforming **2** into **3** were performed under the conditions shown in Scheme 2; for the structures of **2 c**, **2 e** and **3 a** in the solid state, see the SI; Tol=*p*‐tolyl.

The modularity manifest in this case study has arguably little precedent in the literature, but the opportunities are much larger. The method is compatible with numerous functional groups and heterocyclic rings in the periphery (Scheme 2 and Figure 3); many of these compounds would be difficult to make by any other method known to date.^2^, ^3^, ^4^ The power of boronic acid chemistry in general and cross coupling in particular allows the ‐B(pin) substituent to be used in many different ways;^23^ the introduction of non‐aromatic groups as the fourth substituent is an obvious possibility.^22^ Alternatively, such products can be reached by starting from propargylic alcohols that do already bear aliphatic substituents; although this aspect has not been investigated in great detail, the formation of the methylated product **4 j** shows that the reaction proceeds similarly well, as expected (Scheme 4). Trisubstituted furans are also within reach if one is willing to sacrifice the ‐B(pin) group, as illustrated by the formation of **7**. Likewise, building blocks such as **9** are accessible if one uses a tertiary rather than secondary propargyl alcohol. Further conceivable extensions are subject to ongoing investigations.

**Figure 3:**
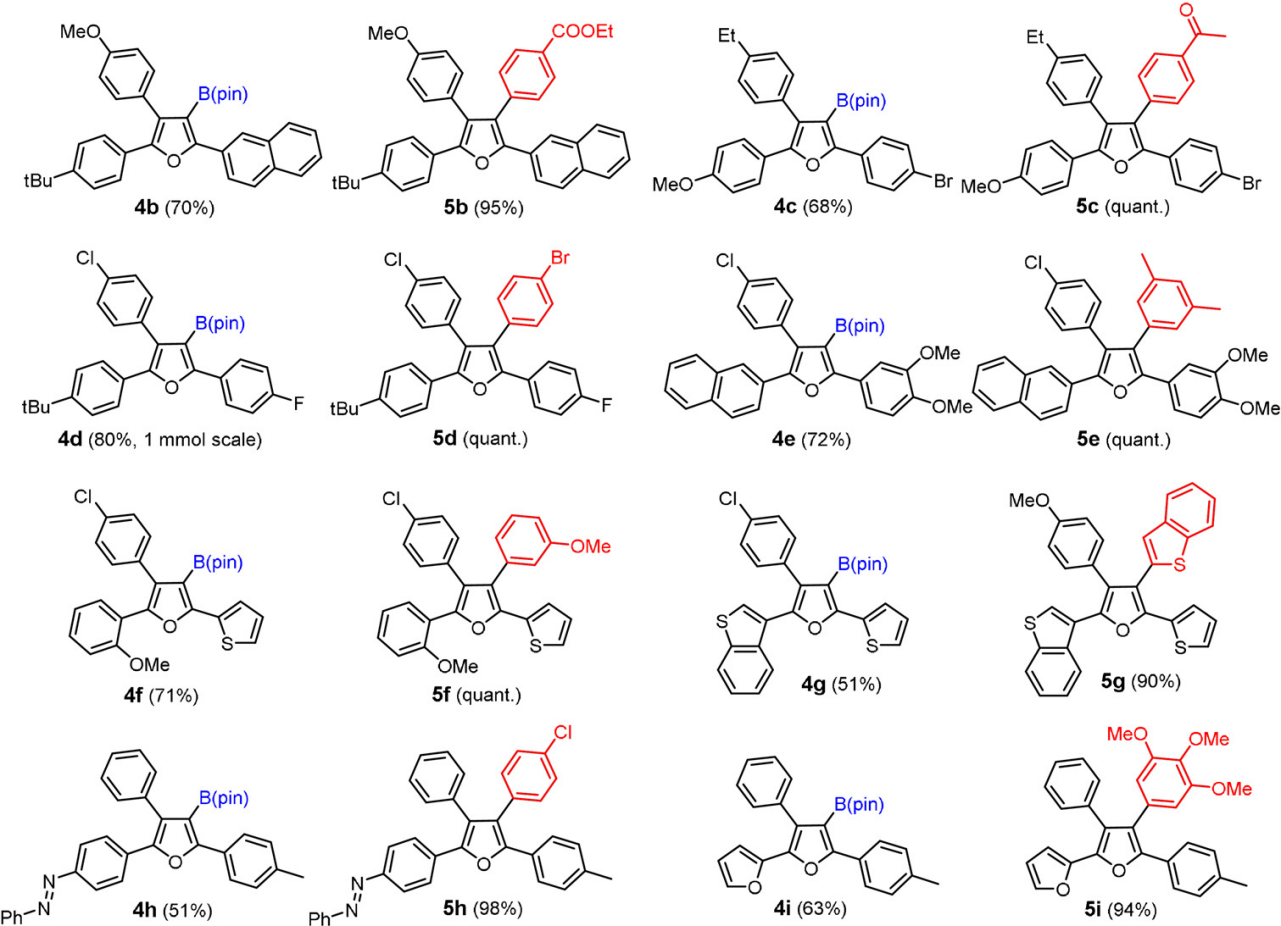
Collection of borylated furans and derived tetra‐arylfuran derivatives illustrating the functional group tolerance of the new method under the conditions specified in Scheme 2 for the formation of **4 a** and **5 a**; for the structures of **4 d**, **5 g** in the solid state, see the SI.

**Scheme 4:**
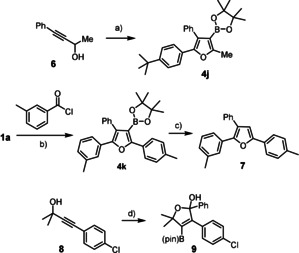
a) (i) KHMDS, 1,4‐dioxane, 0 °C; (ii) B_2_(pin)_2_, *t*BuC_6_H_4_C(O)Cl, Pd_2_(dba)_3_ (5 mol %), CuTC (15 mol %), P(OPh)_3_ (20 mol %), RT, 64 %; b) (i) KHMDS, 1,4‐dioxane, 0 °C; (ii) B_2_(pin)_2_, *m*‐MeC_6_H_4_C(O)Cl, Pd_2_(dba)_3_ (5 mol %), CuTC (15 mol %), P(OPh)_3_ (20 mol %), RT, 63 %; c) [(dppf)PdCl_2_] (5 mol %), aq. KOH, THF, RT, 95 %; d) (i) NaHMDS, 1,4‐dioxane, 0 °C; (ii) B_2_(pin)_2_, PhC(O)Cl, Pd_2_(dba)_3_ (5 mol %), CuTC (15 mol %), P(OPh)_3_ (20 mol %), RT, 79 %; for the structure of borylated lactol **9** in the solid state, see the SI.

The polysubstituted furans available by this method provide ample opportunity for downstream functionalization as illustrated by a few representative examples shown in Scheme 5. Specifically, a Diels–Alder reaction of **3 j** with benzyne generated in situ from 2‐trimethylsilylphenyl triflate (**10**)^24^ furnished **11** in high yield, which can be reductively aromatized with the aid of low‐valent titanium^25^ to give the tetra‐arylated naphthalene derivative **12**.^26^ The ease of these transformations suggests that higher polyarylated acenes should also be accessible, a class of exceptionally important compounds for material science.^1^, ^26^ Equally enabling is oxidative hydrolysis,13a as manifested in the formation of ene‐1,4‐dione **13** bearing four different aryl groups on its backbone. Compounds of this type show interesting physical properties in their own right13a and open entry into other heterocycles as exemplified by the formation of pyrrole **14** and pyridazine **15** by reductive ring closure or treatment with hydrazine, respectively.

**Scheme 5:**
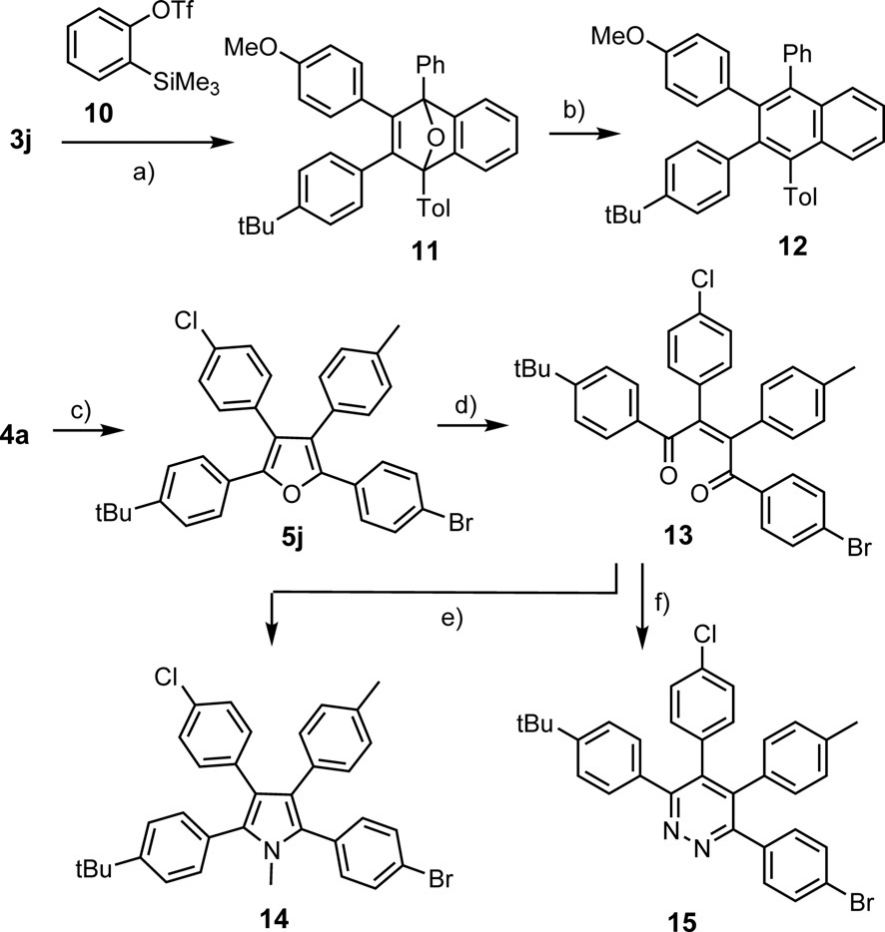
a) **10**, KF, 18‐crown‐6, THF, RT, 90 %; b) TiCl_4_, LiAlH_4_, THF, Et_3_N, reflux, then **11**, RT, 53 %; c) 1‐iodo‐4‐methylbenzene, [(dppf)PdCl_2_] (5 mol %), aq. KOH, THF, RT, 88 %; d) KNO_3_, aq. HOAc, O_2_, 100 °C, 91 %; e) (i) MeNH_2_, MeOH, reflux; (ii) NaBH_4_, RT, 68 %; f) N_2_H_4_⋅H_2_O, MeOH, reflux, 91 %.

In summary, this study presents a straightforward, concise and highly effective synthesis of differentially substituted furans and related building blocks; in terms of its modularity, the new method currently appears unrivaled. Key to success is the stereo‐ and regioselective *trans*‐acylboration of the triple bond of a propargyl alcohol. The potential of reactions taking an entirely non‐canonical *trans*‐addition mode has only recently begun to be fully appreciated^17^, ^27^, ^28^ and basic mechanistic understanding been gained;^29^, ^30^ they continue to be subject to intense study in this laboratory.

## Conflict of interest

The authors declare no conflict of interest.

## Supporting information

As a service to our authors and readers, this journal provides supporting information supplied by the authors. Such materials are peer reviewed and may be re‐organized for online delivery, but are not copy‐edited or typeset. Technical support issues arising from supporting information (other than missing files) should be addressed to the authors.

Supplementary
