# Supplementary material for: Modular Synthesis of Furans with up to Four Different Substituents by a trans‐Carboboration Strategy
Source: Angew Chem Int Ed Engl. 2020 Jun 3;59(32):13618–22. doi: 10.1002/anie.202005560 (PMC7496670; doi:10.1002/anie.202005560)
Supplement: Supplementary file 1 — Supplementary [file ANIE-59-13618-s001.pdf]

Angewandte Chemie
Eine Zeitschrift der Gesellschaft Deutscher Chemiker

Supporting Information

Modular Synthesis of Furans with up to Four Different Substituents by a *trans*-Carboboration Strategy

*Hongming Jin and Alois Fürstner**

anie_202005560_sm_miscellaneous_information.pdf

SUPPORTING CRYSTALLOGRAPHIC INFORMATION

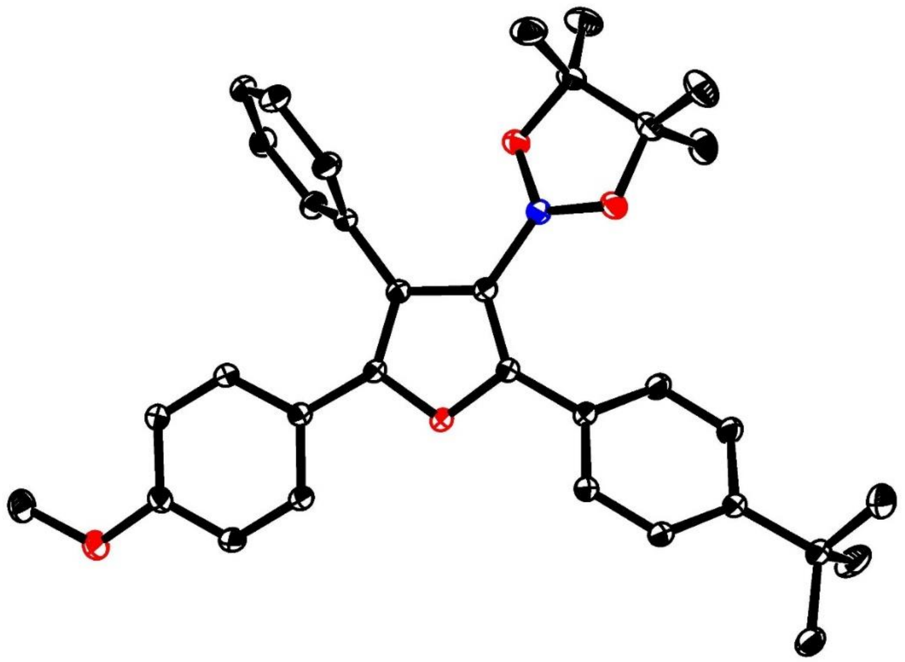

Figure S-1. Structure of the borylated furan derivative **2c** in the solid state; hydrogen atoms not shown for clarity

X-ray Crystal Structure Analysis of Compound 2c: C_{33} H_{37} B O_4, Mr = 508.43 g · mol^{-1}, colorless block, crystal size 0.23 x 0.16 x 0.15 mm^3, triclinic, space group $P\bar{1}$ [2], a = 9.5191(9) Å, b = 11.0554(10) Å, c = 14.6423(13) Å, α = 76.301(7)°, β = 73.345(7)°, γ = 84.497(7)°, V = 1433.6(2) Å^3, T = 100(2) K, Z = 2, D_{calc} = 1.178 g · cm^3, λ = 0.71073 Å, μ(Mo-$K\alpha$) = 0.075 mm^{-1}, Gaussian absorption correction (T_{min} = 0.98, T_{max} = 0.99), Bruker-AXS Kappa Mach3 APEX-II diffractometer with a FR591 rotating Mo-anode X-ray source, 2.656 < Θ < 37.784°, 134143 measured reflections, 15376 independent reflections, 9496 reflections with $I > 2\sigma(I)$, R_{int} = 0.0765.

The structure was solved by *SHELXT* and refined by full-matrix least-squares (*SHELXL*) against F^2 to R_1 = 0.050 [$I > 2\sigma(I)$], wR_2 = 0.147, 351 parameters. The H atoms were found and refined, S = 1.031, residual electron density 0.5 (0.67 Å from C4)/ -0.3 (0.68 Å from C17) e · Å^{-3}. **CCDC- 1994182**.

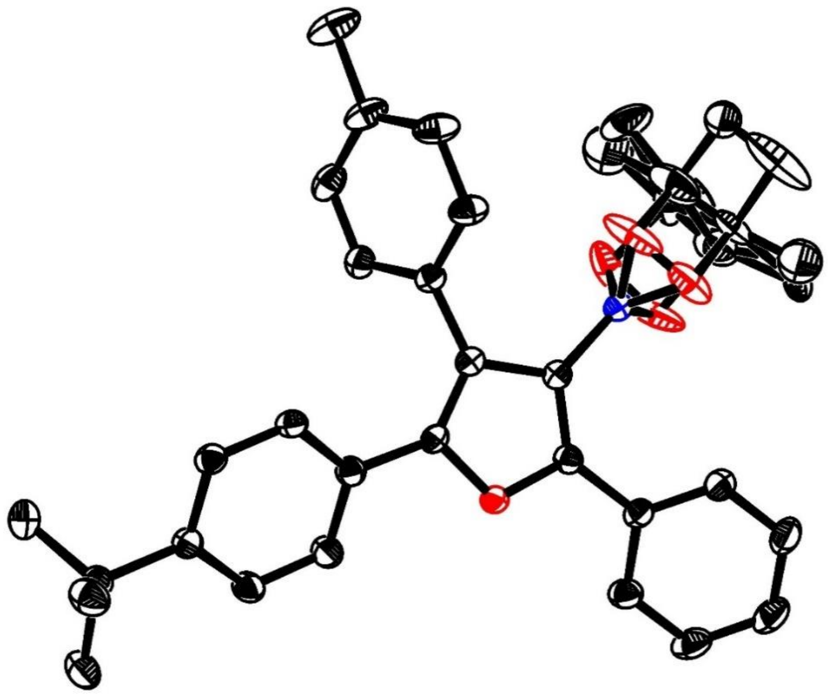

Figure S-2. Structure of the borylated furan derivative **2e** in the solid state; the pinacol boronate unit is disordered over two positions; hydrogen atoms not shown for clarity

X-ray Crystal Structure Analysis of Compound 2e: $C_{33} H_{37} B O_3$, Mr = 492.43 g · mol^{-1}, colorless plate, crystal size 0.179 x 0.082 x 0.040 mm^3, monoclinic, space group $P2_1/n$ [14], a = 6.2460(3) Å, b = 17.3964(7) Å, c = 26.2333(11) Å, β = 95.655(2)°, V = 2836.6(2) Å^3, T = 100(2) K, Z = 4, D_{calc} = 1.153 g · cm^3, λ = 0.71073 Å, μ(Mo-$K\alpha$) = 0.071 mm^{-1}, Gaussian absorption correction (T_{min} = 0.99, T_{max} = 1.00), Bruker-AXS Kappa Mach3 diffractometer with APEX-II detector and IμS micro focus X-ray source, 2.468 < Θ < 36.568°, 118209 measured reflections, 13856 independent reflections, 6050 reflections with $I > 2\sigma(I)$, R_{int} = 0.1268, extinction coefficient = 0.0139(14).

The structure was solved by *SHELXT* and refined by full-matrix least-squares (*SHELXL*) against F^2 to R_1 = 0.077 [$I > 2\sigma(I)$], wR_2 = 0.209, 399 parameters. The H atoms were refined using a riding model, S = 1.028, residual electron density 0.4 (0.52 Å from C32B)/ -0.4 (0.77 Å from H31C) e · Å^{-3}. **CCDC- 1994178**.

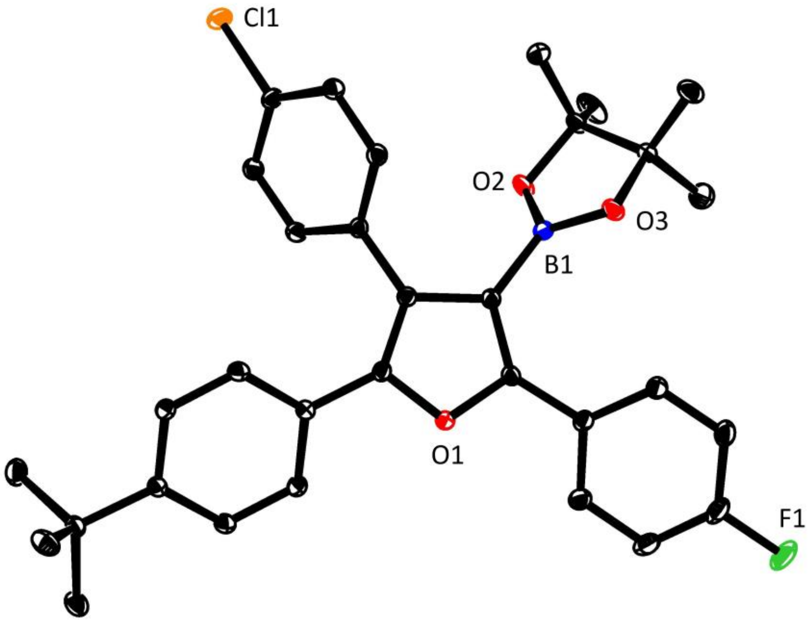

Figure S-3. Structure of the borylated furan derivative **4d** in the solid state; hydrogen atoms not shown for clarity

X-ray Crystal Structure Analysis of Compound 4d: $C_{32} H_{33} B Cl F O_3$, Mr = 530.84 g · mol^{-1}, colorless plate, crystal size 0.275 x 0.077 x 0.031 mm^3, triclinic, space group $P\bar{1}$ [2], a = 6.1791(3) Å, b = 11.3477(5) Å, c = 19.8021(9) Å, α = 78.187(2)°, β = 85.789(2)°, γ = 89.764(2)°, V = 1355.33(11) Å^3, T = 100(2) K, Z = 2, D_{calc} = 1.301 g · cm^3, λ = 0.71073 Å, μ(Mo-Kα) = 0.180 mm^{-1}, Gaussian absorption correction (T_{min} = 0.97, T_{max} = 1.00), Bruker-AXS Kappa Mach3 diffractometer with APEX-II detector and IμS micro focus X-ray source, 3.065 < Θ < 32.577°, 44300 measured reflections, 9862 independent reflections, 7858 reflections with $I > 2\sigma(I)$, R_{int} = 0.0358.

The structure was solved by *SHELXT* and refined by full-matrix least-squares (*SHELXL*) against F^2 to R_1 = 0.042 [$I > 2\sigma(I)$], wR_2 = 0.110, 350 parameters. The H atoms were found and refined, S = 1.026, residual electron density 0.5 (0.73 Å from C5)/ -0.3 (0.49 Å from Cl1) e · Å^{-3}. **CCDC- 1994179.**

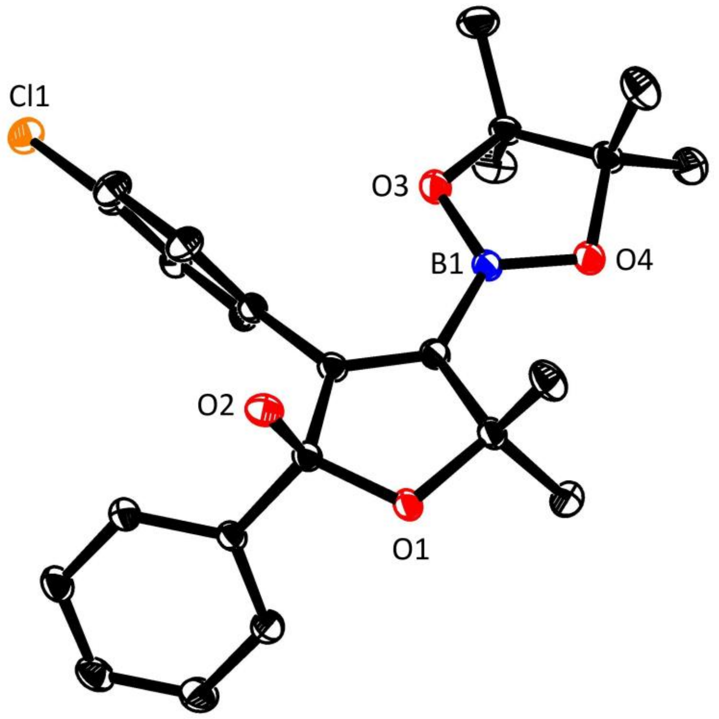

Figure S-4. Structure of the borylated lactol derivative **9** in the solid state; hydrogen atoms not shown for clarity

X-ray Crystal Structure Analysis of Compound 9: $C_{24}H_{28}BClO_4$, *Mr* = 426.72 g · mol^{-1}, colorless block, crystal size 0.45 x 0.40 x 0.38 mm^3, monoclinic, space group $P2_1/c$ [14], *a* = 10.6832(6) Å, *b* = 19.0415(18) Å, *c* = 12.0728(13) Å, β = 110.861(6)°, *V* = 2294.9(4) Å^3, *T* = 100(2) K, *Z* = 4, D_{calc} = 1.235 g · cm^3, λ = 0.71073 Å, μ(*Mo*-*K*α) = 0.193 mm^{-1}, Gaussian absorption correction (T_{min} = 0.93, T_{max} = 0.96), Bruker-AXS Kappa Mach3 APEX-II diffractometer with a FR591 rotating Mo-anode X-ray source, 2.799 < Θ < 35.946°, 57024 measured reflections, 10756 independent reflections, 9057 reflections with $I > 2\sigma(I)$, R_{int} = 0.0313.

The structure was solved by *SHELXT* and refined by full-matrix least-squares (*SHELXL*) against F^2 to R_1 = 0.044 [$I > 2\sigma(I)$], wR_2 = 0.132, 281 parameters. The H atoms were refined using a riding model, *S* = 1.080, residual electron density 1.1 (0.91 Å from O4)/ -0.4 (0.58 Å from Cl1) e · Å^{-3}. **CCDC- 1994180**.

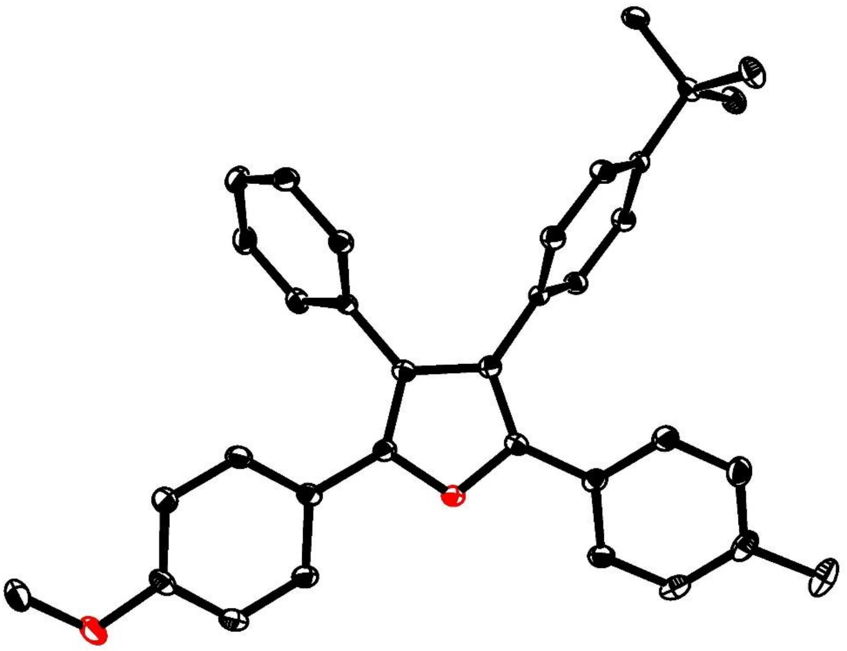

Figure S-5. Structure of the tetra-arylated furan derivative **3a** in the solid state; hydrogen atoms not shown for clarity

X-ray Crystal Structure Analysis of Compound 3a: $C_{34} H_{32} O_2$, Mr = 472.59 g · mol^{-1}, colorless prism, crystal size 0.114 x 0.111 x 0.051 mm^3, monoclinic, space group $C2/c$ [15], a = 43.647(3) Å, b = 6.0410(5) Å, c = 21.1430(16) Å, β = 108.649(3)°, V = 5282.1(7) Å^3, T = 100(2) K, Z = 8, D_{calc} = 1.189 g · cm^3, λ = 0.71073 Å, μ(Mo-$K\alpha$) = 0.072 mm^{-1}, Gaussian absorption correction (T_{min} = 0.99, T_{max} = 1.00), Bruker-AXS Kappa Mach3 diffractometer with APEX-II detector and IµS micro focus X-ray source, 2.955 < Θ < 28.282°, 77093 measured reflections, 6541 independent reflections, 5563 reflections with $I > 2\sigma(I)$, R_{int} = 0.0322.

The structure was solved by *SHELXT* and refined by full-matrix least-squares (*SHELXL*) against F^2 to R_1 = 0.039 [$I > 2\sigma(I)$], wR_2 = 0.101, 330 parameters. The H atoms were refined using a riding model, S = 1.033, residual electron density 0.3 (0.75 Å from C24)/ -0.2 (0.58 Å from C24) e · Å^{-3}. **CCDC- 1994181**.

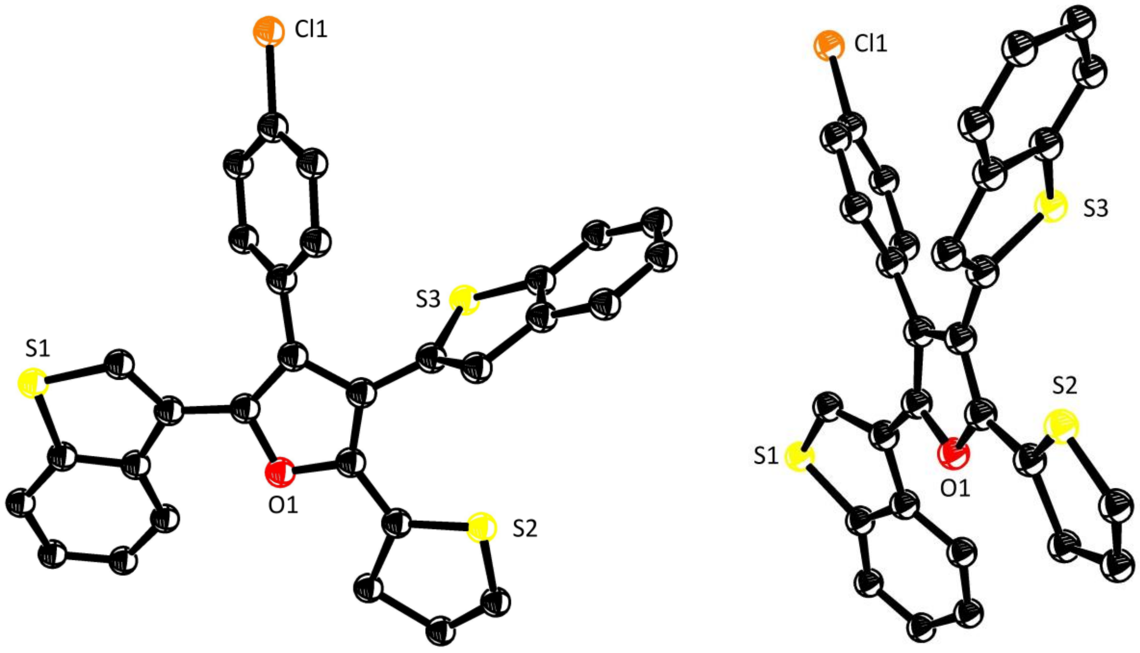

Figure S-6. Structure of the tetra-arylated furan derivative **5g** in the solid state in two different orientation; hydrogen atoms not shown for clarity

X-ray Crystal Structure Analysis of Compound 5g: $C_{31} H_{19} Cl_3 O S_3$, *Mr* = 609.99 g · mol^{-1}, colorless needle, crystal size 0.155 x 0.135 x 0.071 mm^3, triclinic, space group $P\bar{1}$ [2], a = 9.4222(6) Å, b = 10.4782(7) Å, c = 14.6161(10) Å, α = 107.826(4)°, β = 94.429(4)°, γ = 97.117(4)°, V = 1352.99(16) Å^3, T = 100(2) K, Z = 2, D_{calc} = 1.497 g · cm^3, λ = 1.54178 Å, μ(*Cu-K*α) = 5.430 mm^{-1}, Gaussian absorption correction (T_{min} = 0.60, T_{max} = 0.80), Bruker AXS Enraf-Nonius KappaCCD diffractometer with a FR591 rotating Cu-anode X-ray source, 3.200 < Θ < 63.992°, 18458 measured reflections, 4242 independent reflections, 2698 reflections with $I > 2\sigma(I)$, R_{int} = 0.0985, extinction coefficient = 0.0034(6).

The structure was solved by *SHELXT* and refined by full-matrix least-squares (*SHELXL*) against F^2 to R_1 = 0.064 [$I > 2\sigma(I)$], wR_2 = 0.193, 352 parameters. The H atoms were found and refined, S = 1.087, residual electron density 0.5 (1.38 Å from H20A)/ -0.6 (0.14 Å from H20A) e · Å^{-3}. **CCDC- 1994177**.

RETROSYNTHETIC ANALYSIS OF THE COMPLETE LIBRARY OF TETRA-ARYLFURAN ISOMERS

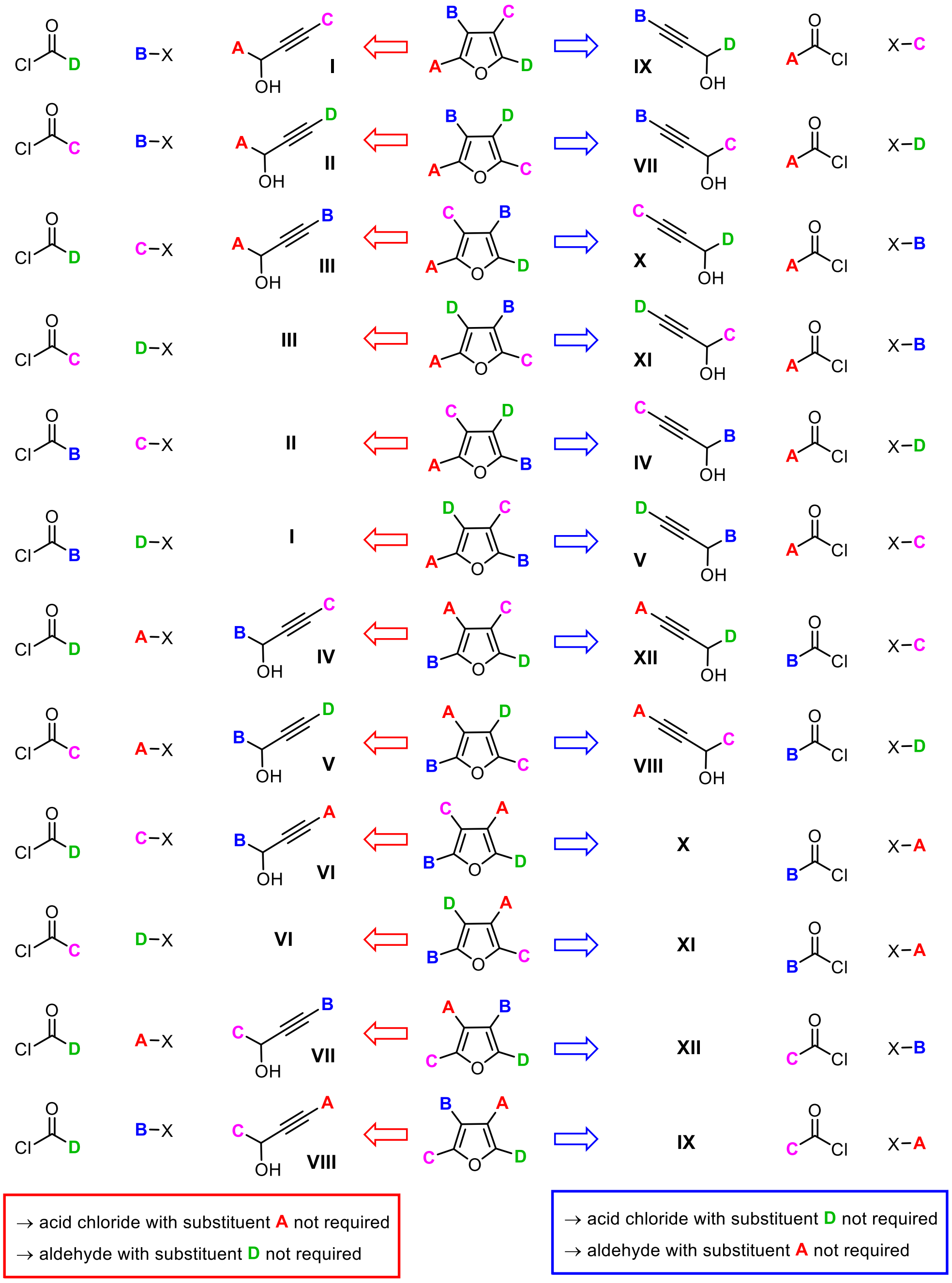

Scheme S-1. Logic of "diagonal split" as applied to the ensemble of 12 isomeric furans formed by permutation of four generic substituents about the heterocyclic core; if the disconnections follow exclusively the "red" or the "blue" path, they lead back to eight propargyl alcohols in either case, which are necessary to make the entire compound collection; if both pathways are considered in parallel, one requires a minimum of six propargyl alcohols, which can be chosen from the pool of twelve candidates (**I** – **XII**).

PROCEDURES AND CHARACTERIZATION DATA

General Methods. Unless stated otherwise, all reactions were carried out under Argon atmosphere in flame-dried glassware. The solvents were purified by distillation over the indicated drying agents under Argon: THF, 1,4-dioxane, hexane, toluene (Na/K); CH_2Cl_2, 1,2-dichloroethane (CaH_2) (stored over molecular sieves and degassed via freeze-pump-thaw cycles). If not mentioned otherwise, NMR spectra were recorded at room temperature using either a Bruker DPX 300, AMX 300 or AV 400 spectrometer in the solvents indicated. Chemical shifts (δ) are given in ppm and coupling constants (*J*) in Hz. The following abbreviations (and combinations thereof) are used to indicate the signal multiplicity: s (singlet), brs (broad singlet), d (doublet), t (triplet), q (quartet) and m (multiplet). Mass spectra (MS and HRMS): Finnigan MAT 8200 (70 eV), ESI-MS: ESQ 3000 (Bruker), Bruker APEX III FT-MS (7 T magnet) or MAT 95 (Finnigan) spectrometer. Infrared Spectroscopy (IR): Spectrum One Perkin Elmer, wavenumbers ($\tilde{\nu}$) in cm^{-1}. Flash Chromatography was carried out using Merck silica gel 60 (40-63 µm) using the indicated eluents. Analytical Thin Layer Chromatography (TLC) was carried out on precoated Macherey-Nagel sheets (POLYGRAM® SIL G/UV254). Preparative TLC: Macherey-Nagel precoated plates (SIL G-100 UV 254; silica gel layer: 1.0 mm); detection was accomplished using UV-light (254 nm), $KMnO_4$ (in 1.5 M Na_2CO_3 (aq.)), molybdatophosphoric acid (5 % in ethanol), vanillin/H_2SO_4 (in ethanol) or anisaldehyde/HOAc (in ethanol).

Borylated Furan Derivatives

Representative Procedure for the Preparation of Borylated Furan Derivatives. Preparation of 2-(5-(4-methoxyphenyl)-4-phenyl-2-(*p*-tolyl)furan-3-yl)-4,4,5,5-tetramethyl-1,3,2-dioxaborolane (2a). A flame-dried tube equipped with a magnetic stir bar was charged under Ar with 1,4-dioxane (2 mL), 3-phenyl-1-(p-tolyl)prop-2-yn-1-ol (44.4 mg, 0.2 mmol), and KHMDS (40 mg, 0.2 mmol). $B_2(pin)_2$ (55.8 mg, 0.22 mmol), $Pd_2(dba)_3$ (9.1 mg, 0.01 mmol), triphenyl phosphite (12.4 mg, 0.04 mmol) CuTC (5.7 mg, 0.03 mmol) and 4-methoxybenzoyl chloride (68 mg, 0.4 mmol) were sequentially added. The tube was sealed and the mixture stirred at room temperature for 24 h. The reaction was quenched with aq. sat. NH_4Cl and $NaHCO_3$ (2×1 mL), the aqueous phase was extracted with EtOAc (3×5mL), and the combined organic layers were dried (Na_2SO_4), filtered and evaporated. The residue was purified by flash chromatography (SiO_2, hexanes/EtOAc) or thin layer chromatography to provide the title compound as a white solid (64.3 mg, 69%). mp: 123-124 °C; ^1H NMR (400 MHz, CD_2Cl_2) δ = 7.73-7.67 (m, 2H), 7.33-7.22 (m, 7H), 7.17-7.10 (m, 2H), 6.72-6.64 (m, 2H), 3.67 (s, 3H), 2.29 (s, 3H), 1.07 (s, 12H) ppm; ^{13}C NMR (101 MHz, CD_2Cl_2) δ = 158.9, 157.0, 147.9, 138.0, 135.2, 130.1, 129.0, 128.8, 128.2, 127.2, 127.1, 126.9, 126.2, 123.7, 113.7, 83.7, 55.2, 24.4, 21.1 ppm; ^{11}B NMR (128 MHz, CD_2Cl_2) δ = 30.4 ppm; IR (ATR): ṽ = 2976, 1507, 1308, 1246, 1142, 1068, 950, 831. 820, 698 cm^{-1}; HRMS (ESI) *m/z* calcd. for $[C_{30}H_{31}BO_4+Na]^+$: 489.2208; found: 489.2205.

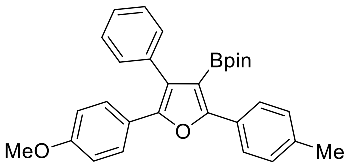

Upscaling: 2-(5-(4-(*tert*-Butyl)phenyl)-4-(4-chlorophenyl)-2-(4-fluorophenyl)furan-3-yl)-4,4,5,5-tetra-methyl-1,3,2-dioxaborolane (4d). A flame-dried tube equipped with a magnetic stir bar was charged under Ar with 1,4-dioxane (10 mL), 3-(4-chlorophenyl)-1-(4-fluorophenyl)prop-2-yn-1-ol (274 mg, 1.05 mmol), and KHMDS (210 mg, 1.05 mmol). $B_2(pin)_2$ (320 mg, 1.26 mmol), $Pd_2(dba)_3$ (23 mg, 0.025 mmol), triphenyl phosphite (26.2 µL, 0.1 mmol) CuTC (14 mg, 0.075 mmol) and 4-*tert*-butylbenzoyl chloride (390 µL, 2.0 mmol) were

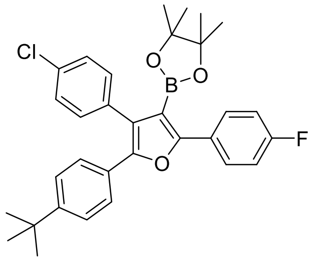

sequentially added. The tube was sealed and the mixture stirred at room temperature for 24 h. The reaction was quenched with aq. sat. NH_4Cl and $NaHCO_3$ (2×1 mL), the aqueous phase was extracted by methyl *tert*-butyl ether (3×10mL), and the combined organic layer were dried (Na_2SO_4), filtered and concentrated. The product was recrystallized and the collected crystals washed with cold pentane. White solid (448 mg, 80%); mp: 174-175 °C; ^1H NMR (400 MHz, CD_2Cl_2) δ = 7.91-7.79 (m, 2H), 7.33-7.17 (m, 8H), 7.10-7.00 (m, 2H), 1.20 (s, 9H), 1.09 (s, 12H) ppm; ^{13}C NMR (101 MHz, CD_2Cl_2) δ = 163.9, 161.4, 156.7, 150.6, 148.5, 133.7, 133.0, 131.7, 128.4 (d, *J* =8 Hz), 128.3, 127.8 (d, *J* = 3 Hz), 127.6, 126.6, 125.4 (d, *J* = 11 Hz), 115.2 (d, *J* = 21 Hz), 83.8, 34.5, 30.9, 24.4 ppm; ^{11}B NMR (128 MHz, CD_2Cl_2) δ = 30.7 ppm; IR (ATR): ṽ = 2965, 1501, 1352, 1318, 1234, 1142, 1066, 952, 835, 687, 556, 515 cm^{-1}; HRMS (ESI) *m/z* calcd. for $[C_{32}H_{33}BClFO_3+H]^+$: 530.2190; found: 530.2193.

Multigram Scale Experiment. 2-(2-(4-Bromophenyl)-5-(4-(*tert*-butyl)phenyl)-4-(4-chlorophenyl)-furan-3-yl)-4,4,5,5-tetramethyl-1,3,2-dioxaborolane (4a). A flame-dried tube equipped with a magnetic stir bar was charged under Ar with 1,4-dioxane (30 mL) and KHMDS (1.24 g, 6.22 mmol). 3-(4-Chlorophenyl)-1-(4-bromophenyl) prop-2-yn-1-ol (2.00 g, 6.22 mmol) was slowly added at 0 °C. After stirring for 10 min, the resulting mixture was warmed to room temperature. $B_2(pin)_2$ (1.9 g, 7.5 mmol), $Pd_2(dba)_3$ (283 mg, 0.31 mmol), triphenyl phosphite (0.33 mL, 1.24 mmol), CuTC (177 mg, 0.93 mmol) and 4-*tert*-butylbenzoyl chloride (2.44 mL, 12.5 mmol) were sequentially added. The tube was sealed and the mixture stirred at room temperature for 48 h. The reaction was quenched with aq. sat. NH_4Cl and $NaHCO_3$ (2×10 mL), the aqueous phase was extracted by CH_2Cl_2 (3×20mL), and the combined organic layer were dried (Na_2SO_4), filtered and concentrated. The residue was washed with cold pentane and then purified by flash chromatography (SiO_2, hexanes/CH_2Cl_2 = 3:1) to provide the title compound as a white solid (2.25 g, 61%). mp: 239-240 °C; ^1H NMR (400 MHz, CD_2Cl_2) δ = 7.79-7.71 (m, 2H), 7.51-7.44 (m, 2H), 7.33-7.26 (m, 4H), 7.26-7.19 (m, 4H), 1.20 (s, 9H), 1.09 (s, 12H) ppm; ^{13}C NMR (101 MHz, CD_2Cl_2) δ = 156.2, 150.8, 148.9, 133.6, 133.0, 131.7, 131.4, 130.4, 128.3, 127.9, 127.5, 126.8, 125.5, 125.4, 121.9, 83.9, 34.5, 30.9, 24.4 ppm; ^{11}B NMR (128 MHz, CD_2Cl_2) δ = 30.5 ppm; IR (ATR): ṽ = 2964, 1543, 1484, 1388, 1372, 1364, 1348, 1313, 1270, 1236, 1213, 1166, 1143, 1116, 1100, 1086, 1065, 1018, 1008, 985, 960, 951, 859, 827, 800, 740, 713, 689, 649 cm^{-1}; HRMS (EI) *m/z* calcd. for $[C_{32}H_{33}BClBrO_3]^+$: 590.1389; found: 590.1396.

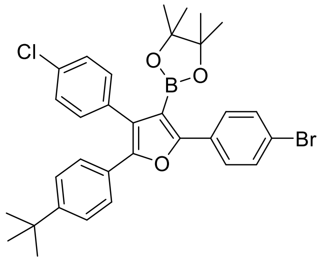

The following compounds were prepared in analogy to the representative procedure:

2-(5-(4-(*tert*-Butyl)phenyl)-4-phenyl-2-(*p*-tolyl)furan-3-yl)-4,4,5,5-tetramethyl-1,3,2-dioxaborolane (2b). White solid (83.2 mg, 69%); mp: 194-195 °C; ^1H NMR (400 MHz, CD_2Cl_2) δ = 7.86-7.56 (m, 2H), 7.38-7.22 (m, 7H), 7.22-7.10 (m, 4H), 2.30 (s, 3H), 1.19 (s, 9H), 1.06 (s, 12H) ppm; ^{13}C NMR (101 MHz, CD_2Cl_2) δ = 157.2, 150.2, 147.9, 138.1, 135.2, 130.1, 129.0, 128.8, 128.2, 128.1, 127.9, 127.1, 126.2, 125.3, 125.2, 83.7, 34.5, 31.0, 24.4, 21.1 ppm; ^{11}B NMR (128 MHz, CD_2Cl_2) δ = 30.2 ppm; IR (ATR): ṽ = 2966, 2865, 1505, 1393, 1355, 1326, 1243, 1166, 1142, 1098, 1074, 1065, 988, 951, 857, 835, 819, 774, 702 cm^{-1}; HRMS (ESI) *m/z* calcd. for $[C_{33}H_{37}BO_3+Na]^+$: 515.2728; found: 515.2732.

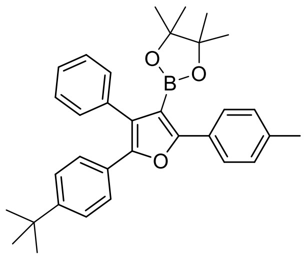

2-(2-(4-(*tert*-Butyl)phenyl)-5-(4-methoxyphenyl)-4-phenylfuran-3-yl)-4,4,5,5-tetramethyl-1,3,2-dioxaborolane (2c).

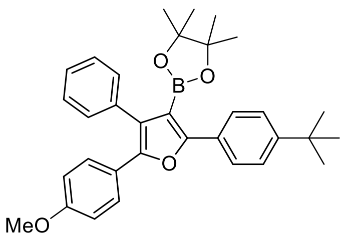

White solid (85.6 mg, 71%); mp: 121-122 °C; ^{1}H NMR (400 MHz, CD_2Cl_2) δ = 7.79-7.68 (m, 2H), 7.41-7.33 (m, 2H), 7.33-7.20 (m, 7H), 6.77-6.63 (m, 2H), 3.68 (s, 3H), 1.28 (s, 9H), 1.09 (s, 12H) ppm; ^{13}C NMR (101 MHz, CD_2Cl_2) δ = 159.0, 156.9, 151.2, 148.0, 135.3, 130.2, 128.9, 128.3, 127.3, 127.2, 127.0, 126.0, 125.4, 123.8, 113.8, 83.8, 55.3, 34.7, 31.1, 24.5 ppm; ^{11}B NMR (128 MHz, CD_2Cl_2) δ = 30.4 ppm; IR (ATR): ṽ = 2962, 1609, 1592, 1509, 1486, 1390, 1353, 1309, 1247, 1176, 1143, 1068, 1032, 986, 951, 856, 832, 791, 772, 698 cm^{-1}; HRMS (ESI) *m/z* calcd. for $[C_{33}H_{37}BO_4+Na]^+$: 531.2677; found: 531.2675.

2-(5-(4-Methoxyphenyl)-2-phenyl-4-(*p*-tolyl)furan-3-yl)-4,4,5,5-tetramethyl-1,3,2-dioxaborolane (2d).

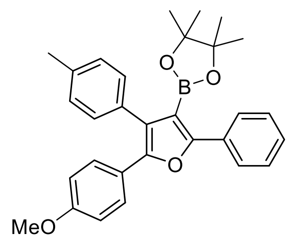

White solid (78.3 mg, 65%); mp: 154-155 °C; ^{1}H NMR (400 MHz, CD_2Cl_2) δ = 7.87-7.72 (m, 2H), 7.39-7.27 (m, 4H), 7.26-7.18 (m, 1H), 7.19-7.03 (m, 4H), 6.77-6.62 (m, 2H), 3.66 (s, 3H), 2.29 (s, 3H), 1.08 (s, 12H) ppm; ^{13}C NMR (101 MHz, CD_2Cl_2) δ = 158.9, 156.4, 148.2, 136.8, 131.9, 131.7, 129.9, 128.8, 128.3, 127.8, 127.3, 126.8, 126.2, 123.7, 113.7, 83.7, 55.2, 24.4, 21.0 ppm; ^{11}B NMR (128 MHz, CD_2Cl_2) δ = 30.8 ppm; IR (ATR): ṽ = 2980, 2928, 1605, 1518, 1497, 1489, 1353, 1308, 1248, 1173, 1143, 1101, 1063, 1030, 984, 949, 856, 832, 823, 770, 696, 524 cm^{-1}; HRMS (ESI) *m/z* calcd. for $[C_{30}H_{31}BO_4+Na]^+$: 489.2207; found: 489.2206.

2-(5-(4-(*tert*-Butyl)phenyl)-2-phenyl-4-(*p*-tolyl)furan-3-yl)-4,4,5,5-tetramethyl-1,3,2-dioxaborolane (2e).

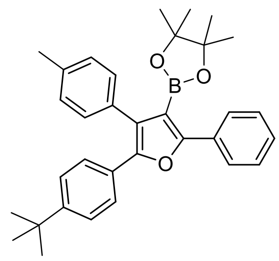

White solid (80 mg, 62%); mp: 152-154 °C; ^{1}H NMR (400 MHz, CD_2Cl_2) δ = 7.87-7.73 (m, 2H), 7.36-7.29 (m, 4H), 7.27-7.21 (m, 1H), 7.21-7.13 (m, 4H), 7.13-7.08 (m, 2H), 2.31 (s, 3H), 1.20 (s, 9H), 1.08 (s, 12H) ppm; ^{13}C NMR (101 MHz, CD_2Cl_2) δ = 156.7, 150.3, 148.2, 136.9, 131.9, 131.6, 129.8, 128.8, 128.3, 128.1, 127.9, 127.8, 126.2, 125.4, 125.2, 83.7, 34.4, 30.9, 24.4, 21.0 ppm; ^{11}B NMR (128 MHz, CD_2Cl_2) δ = 30.1 ppm; IR (ATR): ṽ = 2960, 2905, 1610, 1563, 1516, 1496, 1356, 1315, 1246, 1239, 1173, 1141, 1110, 1061, 1036, 985, 954, 856, 838, 767, 690 cm^{-1}; HRMS (ESI) *m/z* calcd. for $[C_{33}H_{37}BO_4+Na]^+$: 531.2677; found: 531.2684.

2-(2-(4-(*tert*-Butyl)phenyl)-5-(4-methoxyphenyl)-4-(*p*-tolyl)furan-3-yl)-4,4,5,5-tetramethyl-1,3,2-dioxaborolane (2f).

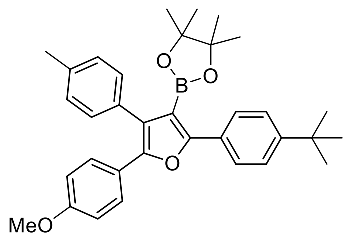

White solid (90 mg, 68%); mp: 192-193 °C; ^{1}H NMR (400 MHz, CD_2Cl_2) δ = 7.79-7.63 (m, 2H), 7.40-7.24 (m, 4H), 7.18-7.11 (m, 2H), 7.11-7.03 (m, 2H), 6.75-6.60 (m, 2H), 3.66 (s, 3H), 2.29 (s, 3H), 1.26 (s, 9H), 1.08 (s, 12H) ppm; ^{13}C NMR (101 MHz, CD_2Cl_2) δ = 158.9, 156.7, 151.1, 147.8, 136.8, 131.9, 129.9, 128.8, 127.2, 126.8, 125.9, 125.3, 123.8, 113.7, 83.7, 55.2, 34.6, 31.0, 24.4, 21.0 ppm; ^{11}B NMR (128 MHz, CD_2Cl_2) δ = 30.4 ppm; IR (ATR): ṽ = 2962, 2867, 1517, 1494, 1354, 1308, 1247, 1177, 1143, 1113, 1067, 1032, 987, 952, 857, 829, 788 cm^{-1}; HRMS (ESI) *m/z* calcd. for $[C_{34}H_{39}BO_4+Na]^+$: 545.2833; found: 545.2833.

2-(4-(4-Methoxyphenyl)-2-phenyl-5-(*p*-tolyl)furan-3-yl)-4,4,5,5-tetramethyl-1,3,2-dioxaborolane (2g). White solid (80 mg, 66%); mp: 126-128 °C; ^{1}H NMR (400 MHz, CD_2Cl_2) δ = 7.84-7.76 (m, 2H), 7.38-7.14 (m, 7H), 7.02-6.93 (m, 2H), 6.87-6.77 (m, 2H), 3.75 (s, 3H), 2.21 (s, 3H), 1.09 (s, 12H) ppm; ^{13}C NMR (101 MHz, CD_2Cl_2) δ = 159.0, 156.6, 148.3, 137.1, 131.6, 131.1, 128.9, 128.3, 128.1, 127.9, 127.4, 127.1, 126.2, 125.6, 113.6, 83.7, 55.2, 24.4, 20.9 ppm; ^{11}B NMR (128 MHz, CD_2Cl_2) δ = 30.7 ppm; IR (ATR): ṽ = 2971, 1780, 1608, 1558, 1517, 1494, 1391, 1353, 1308, 1238, 1171, 1142, 1106, 1064, 1030, 984, 949, 838, 818, 769, 746, 713, 686 cm^{-1}; HRMS (ESI) *m/z* calcd. for $[C_{30}H_{31}BO_4+Na]^+$: 489.2207; found: 489.2213.

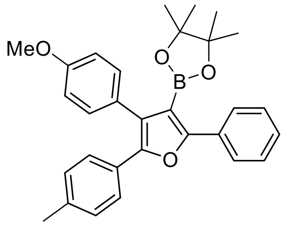

2-(5-(4-(*tert*-Butyl)phenyl)-4-(4-methoxyphenyl)-2-phenylfuran-3-yl)-4,4,5,5-tetramethyl-1,3,2-dioxaborolane (2h). White solid (89 mg, 70%); mp: 160-162 °C; ^{1}H NMR (400 MHz, CD_2Cl_2) δ = 7.89-7.72 (m, 2H), 7.40-7.27 (m, 4H), 7.27-7.14 (m, 5H), 6.92-6.73 (m, 2H), 3.75 (s, 3H), 1.20 (s, 9H), 1.09 (s, 12H) ppm; ^{13}C NMR (101 MHz, CD_2Cl_2) δ = 159.0, 156.6, 150.2, 148.2, 131.6, 131.2, 128.3, 128.1, 127.9, 127.5, 127.2, 126.2, 125.3, 125.2, 113.6, 83.7, 55.2, 34.5, 30.9, 24.4 ppm; ^{11}B NMR (128 MHz, CD_2Cl_2) δ = 30.8 ppm; IR (ATR): ṽ = 2971, 1780, 1608, 1558, 1517, 1494, 1391, 1353, 1308, 1238, 1171, 1142, 1106, 1064, 1030, 984, 949, 838, 818, 769, 746, 713, 686 cm^{-1}; HRMS (ESI) *m/z* calcd. for $[C_{33}H_{37}BO_4+Na]^+$: 531.2677; found: 531.2684.

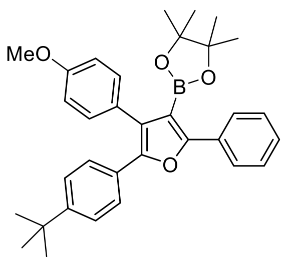

2-(5-(4-(*tert*-Butyl)phenyl)-4-(4-methoxyphenyl)-2-(*p*-tolyl)furan-3-yl)-4,4,5,5-tetramethyl-1,3,2-dioxaborolane (2i). White solid (77.4 mg, 70%); mp: 179-181 °C; ^{1}H NMR (400 MHz, CD_2Cl_2) δ = 7.75-7.63 (m, 2H), 7.37-7.27 (m, 2H), 7.24-7.09 (m, 6H), 6.89-6.76 (m, 2H), 3.74 (s, 3H), 2.30 (s, 3H), 1.20 (s, 9H), 1.09 (s, 12H) ppm; ^{13}C NMR (101 MHz, CD_2Cl_2) δ = 159.0, 157.1, 150.1, 147.9, 138.0, 131.2, 129.0, 128.9, 128.2, 127.5, 127.3, 126.2, 125.24, 125.21, 113.6, 83.6, 55.2, 34.5, 31.0, 24.4, 21.0 ppm; ^{11}B NMR (128 MHz, CD_2Cl_2) δ = 30.8 ppm; IR (ATR): ṽ = 2957, 1505, 1493, 1461, 1389, 1356, 1311, 1284, 1271, 1238, 1166, 1142, 1117, 1099, 1065, 1036, 1020, 985, 951, 857, 833, 817, 787, 694, 647 cm^{-1}; HRMS (ESI) *m/z* calcd. for $[C_{34}H_{39}BO_4]^+$: 522.2936; found: 522.2940.

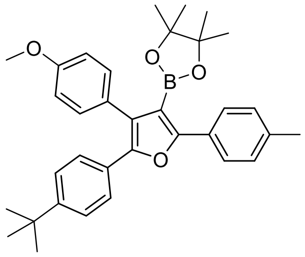

2-(4-(4-(*tert*-Butyl)phenyl)-2-phenyl-5-(*p*-tolyl)furan-3-yl)-4,4,5,5-tetramethyl-1,3,2-dioxaborolane (2j). White solid (85.1 mg, 61%); mp: 176-178 °C; ^{1}H NMR (400 MHz, CD_2Cl_2) δ = 7.87-7.75 (m, 2H), 7.39-7.27 (m, 6H), 7.25-7.18 (m, 3H), 7.02-6.93 (m, 2H), 2.21 (s, 3H), 1.27 (s, 9H), 1.06 (s, 12H) ppm; ^{13}C NMR (101 MHz, CD_2Cl_2) δ = 156.4, 150.2, 148.1, 137.1, 132.0, 131.6, 129.6, 128.9, 128.3, 128.2, 127.9, 127.8, 126.1, 125.6, 125.1, 83.7, 34.5, 31.2, 24.4, 21.0 ppm; ^{11}B NMR (128 MHz, CD_2Cl_2) δ = 30.8 ppm; IR (ATR): ṽ = 2977, 1558, 1493, 1352, 1306, 1237, 1143, 1109, 1063, 1029, 986, 949, 859, 837, 817, 766, 711, 684 cm^{-1}; HRMS (EI) *m/z* calcd. for $[C_{33}H_{37}BO_3]^+$: 492.2830; found: 492.2834.

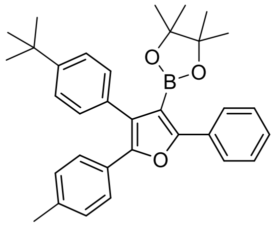

2-(4-(4-(*tert*-Butyl)phenyl)-5-(4-methoxyphenyl)-2-phenylfuran-3-yl)-4,4,5,5-tetramethyl-1,3,2-dioxaborolane (2k).

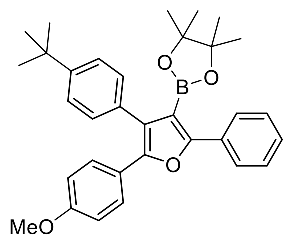

White solid (106.8 mg, 72%); mp: 151-152 ºC; ^{1}H NMR (400 MHz, CD_2Cl_2) δ = 7.87-7.69 (m, 2H), 7.43-7.26 (m, 6H), 7.26-7.14 (m, 3H), 6.79-6.61 (m, 2H), 3.66 (s, 3H), 1.27 (s, 9H), 1.06 (s, 12H) ppm; ^{13}C NMR (101 MHz, CD_2Cl_2) δ = 158.9, 156.1, 150.2, 148.0, 132.0, 131.7, 129.6, 128.3, 127.8, 127.1, 126.9, 126.0, 125.1, 123.8, 113.7, 83.7, 55.2, 34.5, 31.2, 24.4 ppm; ^{11}B NMR (128 MHz, CD_2Cl_2) δ = 30.1 ppm; IR (ATR): ṽ = 2961, 2944, 1606, 1518, 1492, 1380, 1350, 1304, 1245, 1178, 1141, 1107, 1063, 1031, 984, 948, 857, 831, 790, 765, 685 cm^{-1}; HRMS (ESI) *m/z* calcd. for $[C_{33}H_{37}BO_4+Na]^+$: 531.2677; found: 531.2674.

2-(4-(4-(*tert*-Butyl)phenyl)-5-(4-methoxyphenyl)-2-(*p*-tolyl)furan-3-yl)-4,4,5,5-tetramethyl-1,3,2-dioxaborolane (2l).

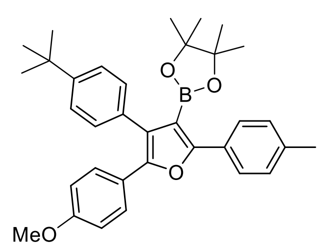

White solid (81.2 mg, 60%); mp: 167-169 ºC; ^{1}H NMR (400 MHz, CD_2Cl_2) δ = 7.77-7.59 (m, 2H), 7.39-7.33 (m, 2H), 7.33-7.28 (m, 2H), 7.23-7.17 (m, 2H), 7.17-7.10 (m, 2H), 6.79-6.64 (m, 2H), 3.68 (s, 3H), 2.30 (s, 3H), 1.27 (s, 9H), 1.06 (s, 12H) ppm; ^{13}C NMR (101 MHz, CD_2Cl_2) δ = 158.8, 156.5, 150.1, 147.6, 137.9, 132.0, 129.6, 129.0, 128.9, 127.0, 126.8, 126.0, 125.0, 123.8, 113.7, 83.6, 55.2, 34.4, 31.1, 24.4, 21.0 ppm; ^{11}B NMR (128 MHz, CD_2Cl_2) δ = 30.6 ppm; IR (ATR): ṽ = 2957, 2863, 1596, 1578, 1507, 1495, 1387, 1353, 1308, 1250, 1177, 1143, 1103, 1067, 1028, 986, 950, 830, 820, 789, 724, 678, 593 cm^{-1}; HRMS (ESI) *m/z* calcd. for $[C_{34}H_{39}BO_4+Na]^+$: 545.2833; found: 545.2831.

2-(5-(4-(*tert*-Butyl)phenyl)-4-(4-methoxyphenyl)-2-(naphthalen-2-yl)furan-3-yl)-4,4,5,5-tetramethyl-1,3,2-dioxaborolane (4b).

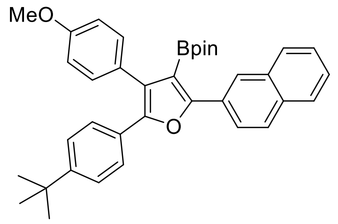

White solid (118.5 mg, 70%); mp: 160-162 ºC; ^{1}H NMR (400 MHz, CD_2Cl_2) δ = 8.32 (s, 1H), 7.98-7.93 (m, 1H), 7.82-7.74 (m, 3H), 7.44-7.36 (m, 4H), 7.26-7.19 (m, 4H), 6.88-6.82 (m, 2H), 3.76 (s, 3H), 1.21 (s, 9H), 1.13 (s, 12H) ppm; ^{13}C NMR (101 MHz, CD_2Cl_2) δ = 159.1, 156.6, 150.3, 148.5, 133.4, 132.9, 131.2, 129.0, 128.15, 128.11, 127.9, 127.8, 127.7, 127.2, 126.4, 126.2, 125.4, 125.3, 124.8, 124.2, 113.6, 83.8, 55.2, 34.5, 31.0, 24.5 ppm; ^{11}B NMR (128 MHz, CD_2Cl_2) δ = 30.8 ppm; IR (ATR): ṽ = 2960, 2933, 1517, 1343, 1306, 1240, 1144, 1097, 1067, 1032, 957, 832, 820, 754, 690 cm^{-1}; HRMS (ESI) *m/z* calcd. for $[C_{37}H_{39}BO_4+Na]^+$: 581.2834; found: 581.2830.

2-(2-(4-Bromophenyl)-4-(4-ethylphenyl)-5-(4-methoxyphenyl)furan-3-yl)-4,4,5,5-tetramethyl-1,3,2-dioxaborolane (4c).

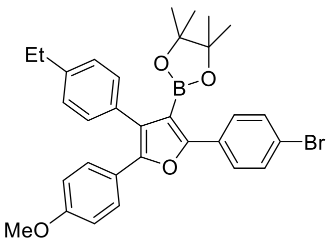

Light yellow solid (85.1 mg, 68%); mp: 155-156 ºC; ^{1}H NMR (400 MHz, CD_2Cl_2) δ = 7.77-7.68 (m, 2H), 7.50-7.41 (m, 2H), 7.37-7.29 (m, 2H), 7.21-7.09 (m, 4H), 6.75-6.67 (m, 2H), 3.68 (s, 3H), 2.61 (q, *J* = 8.0 Hz, 2H), 1.18 (t, *J* = 8.0 Hz, 3H), 1.08 (s, 12H) ppm; ^{13}C NMR (101 MHz, CD_2Cl_2) δ = 159.0, 155.2, 148.4, 143.4, 132.0, 131.4, 130.7, 129.9, 127.7, 127.6, 127.2, 127.1, 123.5, 121.5, 113.7, 83.8, 55.2, 28.7, 24.4, 15.6 ppm; ^{11}B NMR (128 MHz, CD_2Cl_2) δ = 30.7 ppm; IR (ATR): ṽ = 2973, 2930, 1518, 1496, 1484, 1353, 1313, 1247, 1177, 1142, 1066, 1009, 951, 829 cm^{-1}; HRMS (ESI) *m/z* calcd. for $[C_{31}H_{32}BBrO_4+Na]^+$: 581.1469; found: 581.1468.

2-(4-(4-Chlorophenyl)-2-(3,4-dimethoxyphenyl)-5-(naphthalen-2-yl)furan-3-yl)-4,4,5,5-tetramethyl-1,3,2-dioxaborolane (4e). White solid (92 mg, 72%); mp: 173-174 °C; ^{1}H NMR (400 MHz, CD_2Cl_2) δ = 7.89 (s, 1H), 7.70-7.62 (m, 2H), 7.62-7.55 (m, 2H), 7.51-7.46 (m, 1H), 7.41-7.32 (m, 3H), 7.32-7.25 (m, 4H), 6.87 (d, *J* = 8.0 Hz, 1H), 3.86 (s, 3H), 3.81 (s, 3H), 1.09 (s, 12H) ppm; ^{13}C NMR (101 MHz, CD_2Cl_2) δ = 158.5, 149.7, 149.0, 147.8, 133.8, 133.3, 133.1, 132.4, 131.8, 128.3, 128.1, 128.0, 127.9, 127.7, 127.5, 126.3, 126.0, 124.4, 124.3, 123.8, 119.8, 111.2, 110.6, 83.7, 56.0, 55.8, 24.5 ppm; ^{11}B NMR (128 MHz, CD_2Cl_2) δ = 31.2 ppm; IR (ATR): ṽ = 2976, 2933, 1504, 1310, 1264, 1224, 1142, 1089, 1018, 856, 818, 742, 635 cm^{-1}; HRMS (ESI) *m/z* calcd. for $[C_{34}H_{32}BClO_5+Na]^+$: 589.1924; found: 589.1925.

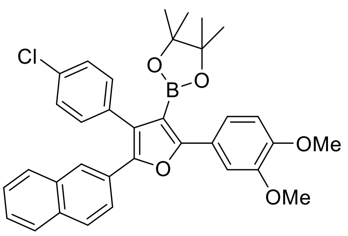

2-(4-(4-Chlorophenyl)-5-(2-methoxyphenyl)-2-(thiophen-2-yl)furan-3-yl)-4,4,5,5-tetramethyl-1,3,2-dioxaborolane (4f). White solid (122 mg, 71%); mp: 138-139 °C; ^{1}H NMR (400 MHz, CD_2Cl_2) δ = 7.70-7.66 (m, 1H), 7.31-7.20 (m, 3H), 7.17-7.06 (m, 4H), 7.03-6.97 (m, 1H), 6.90-6.83 (m, 1H), 6.81-6.75 (m, 1H), 3.38 (s, 3H), 1.18 (s, 12H) ppm; ^{13}C NMR (101 MHz, CD_2Cl_2) δ = 156.9, 154.1, 146.4, 134.0, 133.7, 132.2, 131.0, 130.7, 130.1, 128.4, 127.5, 127.4, 125.8, 125.6, 120.4, 119.5, 111.5, 83.9, 54.9, 24.6 ppm; ^{11}B NMR (128 MHz, CD_2Cl_2) δ = 30.4 ppm; IR (ATR): ṽ = 2976, 2933, 1498, 1435, 1343, 1313, 1252, 1141, 1089, 1061, 1026, 958, 926, 853, 830, 753, 700, 665, 525 cm^{-1}; HRMS (ESI) *m/z* calcd. for $[C_{27}H_{26}BClO_4S+Na]^+$: 515.1226; found: 515.1224.

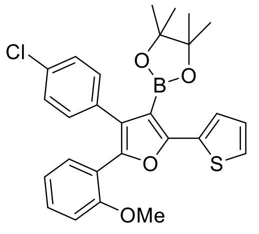

2-(5-(Benzo[b]thiophen-3-yl)-4-(4-chlorophenyl)-2-(thiophen-2-yl)furan-3-yl)-4,4,5,5-tetramethyl-1,3,2-dioxaborolane (4g). Yellow solid (61 mg, 51%); mp: 181-182 °C; ^{1}H NMR (400 MHz, CD_2Cl_2) δ = 8.12-8.05 (m, 1H), 7.79-7.72 (m, 2H), 7.35-7.16 (m, 7H), 7.07 (s, 1H), 7.03-6.99 (m, 1H), 1.14 (s, 12H) ppm; ^{13}C NMR (101 MHz, CD_2Cl_2) δ = 153.7, 145.0, 139.8, 137.1, 133.9, 133.0, 132.9, 131.5, 128.6, 128.3, 127.7, 125.9, 125.8, 125.7, 125.3, 124.7, 124.0, 122.6, 84.0, 24.6 ppm; ^{11}B NMR (128 MHz, CD_2Cl_2) δ = 30.0 ppm; IR (ATR): ṽ = 2975, 1514, 1385, 1311, 1140, 1087, 1071, 1012, 967, 851, 828, 758, 697 cm^{-1}; HRMS (ESI) *m/z* calcd. for $[C_{28}H_{24}BClO_3S_2+H]^+$: 519.1021; found: 519.1019.

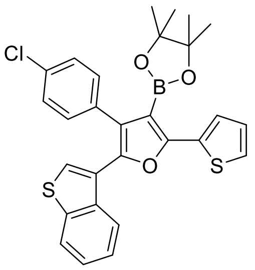

(*E*)-1-Phenyl-2-(4-(3-phenyl-4-(4,4,5,5-tetramethyl-1,3,2-dioxaborolan-2-yl)-5-(p-tolyl)furan-2-yl)phenyl)diazene (4h). Red solid (55 mg, 51%); mp: 179-180 °C; ^{1}H NMR (400 MHz, CD_2Cl_2) δ = 7.82-7.65 (m, 6H), 7.56-7.49 (m, 2H), 7.43-7.24 (m, 8H), 7.19-7.12 (m, 2H), 2.30 (s, 3H), 1.06 (s, 12H) ppm; ^{13}C NMR (101 MHz, CD_2Cl_2) δ = 158.3, 152.8, 151.0, 147.0, 138.6, 134.8, 133.4, 130.9, 130.5, 130.0, 129.14, 129.10, 128.5, 128.4, 127.5, 126.4, 125.9, 123.0, 122.7, 83.8, 24.4, 21.1 ppm; ^{11}B NMR (128 MHz, CD_2Cl_2) δ = 30.2 ppm; IR (ATR): ṽ = 2976, 2921, 1598, 1505, 1392, 1353, 1313, 1247, 1141, 1105, 1066, 985, 949, 848, 820, 770, 684, 548 cm^{-1}; HRMS (ESI) *m/z* calcd. for $[C_{35}H_{33}BN_2O_3+H]^+$: 541.2657; found: 541.2657.

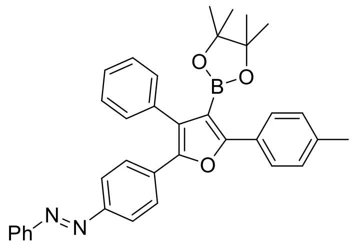

4,4,5,5-Tetramethyl-2-(3-phenyl-5-(*p*-tolyl)-[2,2'-bifuran]-4-yl)-1,3,2-dioxaborolane (4i). White solid (65 mg, 63%); mp: 111-113 °C; ^{1}H NMR (400 MHz, CD_2Cl_2) δ = 7.75-7.62 (m, 2H), 7.37-7.22 (m, 6H), 7.17-7.11 (m, 2H), 6.35-6.22 (m, 2H), 2.30 (s, 3H), 1.09 (s, 12H) ppm; ^{13}C NMR (101 MHz, CD_2Cl_2) δ = 157.9, 146.0, 141.8, 140.8, 138.4, 133.8, 130.0, 129.0, 128.4, 128.2, 127.8, 127.3, 126.4, 111.1, 106.8, 83.8, 24.4, 21.1 ppm; ^{11}B NMR (128 MHz, CD_2Cl_2) δ = 30.7 ppm; IR (ATR): ṽ = 2975, 1504, 1485, 1402, 1315, 1247, 1139, 1078, 962, 853, 819, 735, 698 cm^{-1}; HRMS (ESI) *m/z* calcd. for $[C_{27}H_{27}BO_4+Na]^+$: 449.1895; found: 449.1892.

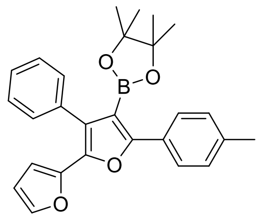

2-(5-(4-(*tert*-Butyl)phenyl)-2-methyl-4-phenylfuran-3-yl)-4,4,5,5-tetramethyl-1,3,2-dioxaborolane (4j). White solid (45.2 mg, 64%); mp: 159-160 °C; ^{1}H NMR (400 MHz, CD_2Cl_2) δ = 7.26-7.12 (m, 9 H), 2.44 (s, 3 H), 1.18 (s, 9 H), 1.11 (s, 12 H) ppm; ^{13}C NMR (101 MHz, CD_2Cl_2) δ = 160.7, 149.9, 147.3, 135.1, 130.2, 128.3, 127.8, 126.7, 126.1, 125.3, 125.1, 82.9, 34.4, 30.9, 24.4, 13.8 ppm; ^{11}B NMR (128 MHz, CD_2Cl_2) δ = 30.0 ppm; IR (ATR): ṽ = 2964, 2868, 1600, 1579, 1513, 1445, 1408, 1355, 1316, 1243, 1141, 1077, 1054, 979, 954, 855, 834, 761, 699 cm^{-1}; HRMS (EI) *m/z* calcd. for $[C_{27}H_{33}BO_3]$: 416.2522; found: 416.2526.

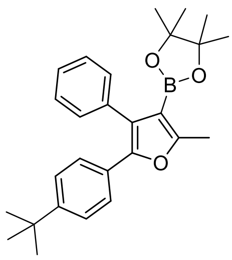

4,4,5,5-Tetramethyl-2-(4-phenyl-5-(*m*-tolyl)-2-(*p*-tolyl)furan-3-yl)-1,3,2-dioxaborolane (4k). White solid (60 mg, 63%); mp: 122-123 °C; ^{1}H NMR (400 MHz, CD_2Cl_2) δ = 7.77-7.67 (m, 2H), 7.32-7.22 (m, 6H), 7.18-7.12 (m, 2H), 7.12-7.07 (m, 1H), 7.04-6.96 (m, 1H), 6.95-6.88 (m, 1H), 2.30 (s, 3H), 2.17 (s, 3H), 1.07 (s, 12H) ppm; ^{13}C NMR (101 MHz, CD_2Cl_2) δ = 157.4, 147.9, 138.2, 138.0, 135.1, 130.8, 130.1, 129.0, 128.7, 128.3, 128.1, 128.0, 127.9, 127.2, 126.3, 126.2, 122.9, 83.7, 24.4, 21.1, 21.0 ppm; ^{11}B NMR (128 MHz, CD_2Cl_2) δ = 30.8 ppm; IR (ATR): ṽ = 2981, 2919, 1553, 1501, 1395, 1357, 1303, 1250, 1142, 1092, 1067, 984, 967, 852, 814, 789, 771, 699 cm^{-1}; HRMS (ESI) *m/z* calcd. for $[C_{30}H_{31}BO_3+Na]^+$: 473.2258; found: 473.2256.

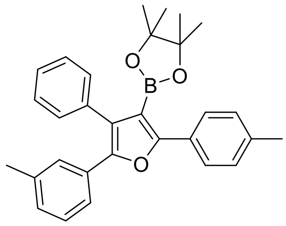

3-(4-Chlorophenyl)-5,5-dimethyl-2-phenyl-4-(4,4,5,5-tetramethyl-1,3,2-dioxaborolan-2-yl)-2,5-dihydrofuran-2-ol (9). White solid (98.4 mg, 79%); mp: 141-142 °C; ^{1}H NMR (400 MHz, [D_6]-acetone) δ = 7.51-7.45 (m, 2H), 7.39-7.34 (m, 2H), 7.26-7.14 (m, 5H), 5.60 (s, 1H), 1.6 (s, 3H), 1.59 (s, 3H), 1.25 (s, 12H) ppm; ^{13}C NMR (101 MHz, acetone-d_6) δ = 151.3, 143.1, 133.5, 132.8, 130.7, 127.4, 127.2, 126.8, 110.3, 89.2, 83.6, 28.7, 27.7, 24.1, 23.9 ppm; ^{11}B NMR (128 MHz, acetone-d_6) δ = 29.9 ppm; IR (ATR): ṽ = 3470, 2977, 1614, 1372, 1323, 1297, 1162, 1123, 1107, 1084, 1063, 1016, 844, 667 cm^{-1}; HRMS (ESI) *m/z* calcd. for $[C_{24}H_{28}BClO_4+Na]^+$: 449.1661; found: 449.1665.

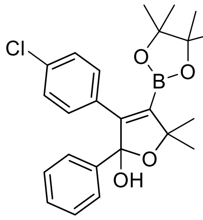

Tetrasubstituted Furan Derivatives

Representative Procedure for the Suzuki Cross Coupling. 3-(4-(*tert*-Butyl)phenyl)-5-(4-methoxyphenyl)-4-phenyl-2-(*p*-tolyl)furan (3a). A flame-dried Schlenk flask was charged with 3-borylfuran **2a** (0.15 mmol, 70 mg) and THF (1.5 mL). Pd(dppf)Cl_2 (5.5 mg, 5 mol %), 1-(*tert*-butyl)-4-iodobenzene (78 mg, 0.3 mmol), KOH solution (9 M, 66.5 µL, 0.6 mmol) were added under Ar atmosphere and the resulting mixture was stirred at room temperature for 12 h. After evaporation of all volatile materials, the crude product was purified by flash chromatography on silica to afford the title compound as a white solid material (74.0 mg, quant.); mp: 137-138 °C; ^{1}H NMR (400 MHz, CD_2Cl_2) δ = 7.42-6.87 (m, 15H), 6.79-6.61 (m, 2H), 3.67 (s, 3H), 2.21 (s, 3H), 1.20 (s, 9H) ppm; ^{13}C NMR (101 MHz, CD_2Cl_2) δ = 159.0, 150.1, 147.5, 147.4, 137.1, 133.7, 130.5, 130.3, 130.0, 129.0, 128.3, 127.1, 127.0, 125.6, 125.2, 124.3, 123.7, 113.8, 55.2, 34.4, 31.1, 20.9 ppm; IR (ATR): ṽ = 2957, 1608, 1510, 1298, 1249, 1177, 1107, 1033, 946, 832, 821, 775, 699 cm^{-1}; HRMS (ESI) *m/z* calcd. for $[C_{34}H_{32}O_2+Na]^+$: 495.2294; found: 495.2291.

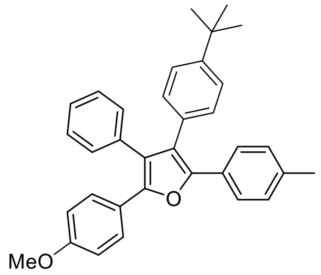

The following compounds were prepared analogously:

2-(4-(*tert*-Butyl)phenyl)-4-(4-methoxyphenyl)-3-phenyl-5-(*p*-tolyl)furan (3b). White solid (50 mg, 88%); mp: 150-151 °C; ^{1}H NMR (400 MHz, CD_2Cl_2) δ = 7.37-7.24 (m, 4H), 7.24-7.13 (m, 5H), 7.13-7.05 (m, 2H), 7.05-6.91 (m, 4H), 6.76-6.62 (m, 2H), 3.66 (s, 3H), 2.21 (s, 3H), 1.19 (s, 9H) ppm; ^{13}C NMR (101 MHz, CD_2Cl_2) δ = 158.8, 150.4, 147.6, 147.4, 137.2, 133.7, 131.5, 130.5, 129.0, 128.33, 128.29, 128.2, 127.1, 125.6, 125.5, 125.3, 125.2, 124.8, 124.2, 113.7, 55.1, 34.5, 30.9, 20.9 ppm; IR (ATR): ṽ = 2952, 2866, 1517, 1498, 1460, 1285, 1245, 1173, 1118, 1030, 946, 832, 820, 775, 700, 559 cm^{-1}; HRMS (ESI) *m/z* calcd. for $[C_{34}H_{32}O_2+Na]^+$: 495.2294; found: 495.2293.

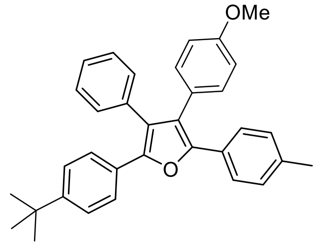

2-(4-(*tert*-Butyl)phenyl)-5-(4-methoxyphenyl)-4-phenyl-3-(*p*-tolyl)furan (3c). White solid (27.1 mg, 95%); mp: 190-192 °C; ^{1}H NMR (400 MHz, CD_2Cl_2) δ = 7.38-7.26 (m, 4H), 7.23-7.13 (m, 5H), 7.12-7.05 (m, 2H), 7.03-6.93 (m, 4H), 6.76-6.67 (m, 2H), 3.68 (s, 3H), 2.23 (s, 3H), 1.20 (s, 9H) ppm; ^{13}C NMR (101 MHz, CD_2Cl_2) δ = 159.0, 150.2, 147.3, 136.9, 133.7, 130.5, 130.4, 130.2, 129.0, 128.3, 127.1, 127.0, 125.2, 125.1, 124.5, 123.8, 123.7, 113.8, 55.2, 34.5, 31.0, 21.0 ppm; IR (ATR): ṽ = 2959, 2866, 1606, 1598, 1510, 1495, 1296, 1244, 1175, 1113, 1031, 947, 835, 828, 775, 700 cm^{-1}; HRMS (EI) *m/z* calcd. for $[C_{34}H_{32}O_2]^+$: 472.2402; found: 472.2409.

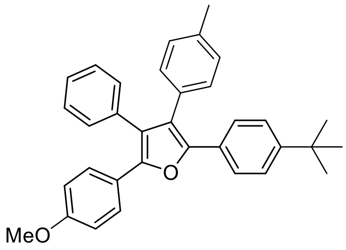

3-(4-(*tert*-Butyl)phenyl)-5-(4-methoxyphenyl)-2-phenyl-4-(*p*-tolyl)furan (3d). White solid (43 mg, quant); mp: 194-195 °C; ^{1}H NMR (400 MHz, CD_2Cl_2) δ = 7.44-7.27 (m, 4H), 7.26-7.18 (m, 2H), 7.18-7.06 (m, 3H), 7.06-6.89 (m, 6H), 6.78-6.61 (m, 2H), 3.67 (s, 3H), 2.23 (s, 3H), 1.21 (s, 9H) ppm; ^{13}C NMR (101 MHz, CD_2Cl_2) δ = 159.0, 150.2, 147.8, 147.1, 136.9, 131.2, 130.4, 130.3, 129.9, 129.1, 128.3, 127.2, 126.9, 125.6, 125.3, 125.1, 123.8, 123.7, 113.7, 55.2, 34.4, 31.1, 21.0 ppm; IR (ATR): ṽ = 2965, 2950, 1604, 1516, 1497, 1443, 1242, 1177, 1108, 1027, 946, 847, 833, 800, 770, 695, 659, 520 cm^{-1}; HRMS (ESI) *m/z* calcd. for $[C_{34}H_{32}O_2+Na]^+$: 495.2294;

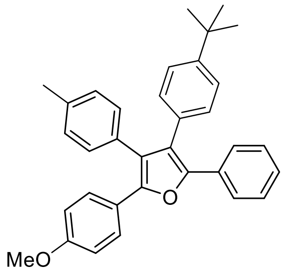

found: 495.2292.

2-(4-(*tert*-Butyl)phenyl)-4-(4-methoxyphenyl)-5-phenyl-3-(*p*-tolyl)furan (3e). White solid (21.3 mg, 89%); mp: 203-204 °C; ^{1}H NMR (400 MHz, CD_2Cl_2) δ = 7.47-7.38 (m, 2H), 7.38-7.30 (m, 2H), 7.24-7.14 (m, 4H), 7.14-7.07 (m, 1H), 7.06-6.93 (m, 6H), 6.77-6.65 (m, 2H), 3.68 (s, 3H), 2.24 (s, 3H), 1.21 (s, 9H) ppm; ^{13}C NMR (101 MHz, CD_2Cl_2) δ = 158.8, 150.4, 147.7, 147.2, 136.9, 131.4, 131.1, 130.4, 130.3, 129.1, 128.3, 128.2, 127.1, 125.5, 125.4, 125.3, 125.0, 124.9, 113.8, 55.1, 34.5, 31.0, 21.0 ppm; IR (ATR): ṽ = 2955, 2865, 1609, 1563, 1512, 1496, 1461, 1285, 1240, 1175, 1123, 1108, 1026, 945, 845, 825, 767, 695, 685 cm^{-1}; HRMS (ESI) *m/z* calcd. for $[C_{34}H_{32}O_2+Na]^+$: 495.2294; found: 495.2292.

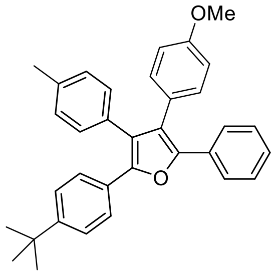

2-(4-(*tert*-Butyl)phenyl)-5-(4-methoxyphenyl)-3-phenyl-4-(*p*-tolyl)furan (3f). White solid (17.7 mg, 98%); mp: 149-151 °C; ^{1}H NMR (400 MHz, CD_2Cl_2) δ = 7.40-7.25 (m, 4H), 7.25-7.14 (m, 5H), 7.14-7.07 (m, 2H), 7.05-6.89 (m, 4H), 6.77-6.66 (m, 2H), 3.69 (s, 3H), 2.23 (s, 3H), 1.20 (s, 9H) ppm; ^{13}C NMR (101 MHz, CD_2Cl_2) δ = 159.0, 150.2, 147.5, 147.2, 136.8, 133.7, 130.5, 130.3, 130.2, 129.0, 128.3, 128.2, 127.1, 127.0, 125.3, 125.2, 124.7, 123.8, 123.7, 113.7, 55.2, 34.5, 30.9, 20.9 ppm; IR (ATR): ṽ = 2958, 2866, 1607, 1516, 1497, 1460, 1440, 1296, 1249, 1177, 1107, 1049, 1024, 945, 829, 802, 776, 702, 678, 527 cm^{-1}; HRMS (ESI) *m/z* calcd. for $[C_{34}H_{32}O_2+Na]^+$: 495.2294; found: 495.2289.

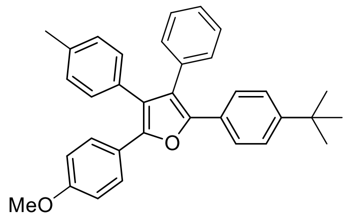

3-(4-(*tert*-Butyl)phenyl)-4-(4-methoxyphenyl)-2-phenyl-5-(*p*-tolyl)furan (3g). White solid (46 mg, quant); mp: 235-236 °C; ^{1}H NMR (400 MHz, CD_2Cl_2) δ = 7.45-7.35 (m, 2H), 7.34-7.26 (m, 2H), 7.25-7.09 (m, 5H), 7.07-6.91 (m, 6H), 6.80-6.66 (m, 2H), 3.69 (s, 3H), 2.23 (s, 3H), 1.22 (s, 9H) ppm; ^{13}C NMR (101 MHz, CD_2Cl_2) δ = 158.8, 150.2, 147.8, 147.2, 137.2, 131.5, 131.1, 130.2, 129.9, 129.0, 128.3, 128.2, 127.1, 125.6, 125.5, 125.2, 124.2, 113.7, 55.1, 34.4, 31.0, 21.0 ppm; IR (ATR): ṽ = 2958, 2868, 1600, 1517, 1495, 1463, 1441, 1285, 1245, 1188, 1174, 1116, 1106, 1026, 943, 847, 833, 821, 801, 770, 712, 691, 681, 561 cm^{-1}; HRMS (EI) *m/z* calcd. for $[C_{34}H_{32}O_2]^+$: 472.2402; found: 472.2408.

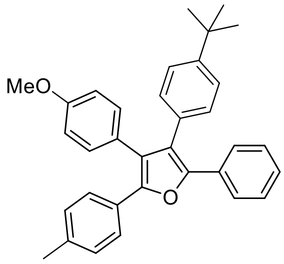

2-(4-(*tert*-Butyl)phenyl)-3-(4-methoxyphenyl)-5-phenyl-4-(*p*-tolyl)furan (3h). White solid (43.4 mg, 92%); mp: 204-206 °C; ^{1}H NMR (400 MHz, CD_2Cl_2) δ = 7.46-7.38 (m, 2H), 7.38-7.30 (m, 2H), 7.26-7.07 (m, 5H), 7.06-6.91 (m, 6H), 6.78-6.65 (m, 2H), 3.69 (s, 3H), 2.24 (s, 3H), 1.21 (s, 9H) ppm; ^{13}C NMR (101 MHz, CD_2Cl_2) δ = 158.8, 150.3, 147.8, 147.1, 136.9, 131.5, 131.1, 130.3, 130.2, 129.1, 128.3, 128.2, 127.1, 127.0, 125.6, 125.4, 125.3, 125.2, 124.5, 113.7, 55.1, 34.5, 31.0, 21.0 ppm; IR (ATR): ṽ = 2959, 2867, 1600, 1514, 1494, 1462, 1441, 1408, 1286, 1265, 1244, 1174, 1121, 1106, 1051, 1026, 945, 839, 824, 796, 738, 652 cm^{-1}; HRMS (EI) *m/z* calcd. for $[C_{34}H_{32}O_2]^+$: 472.2402; found: 472.2401.

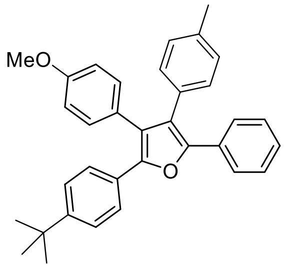

2-(4-(*tert*-Butyl)phenyl)-3-(4-methoxyphenyl)-4-phenyl-5-(*p*-tolyl)furan (3i). White solid (63.6 mg, 96%); mp: 200-201 °C; ^{1}H NMR (400 MHz, CD_2Cl_2) δ = 7.38-7.31 (m, 2H), 7.31-7.25 (m, 2H), 7.23-7.13 (m, 5H), 7.12-7.05 (m, 2H), 7.03-6.94 (m, 4H), 6.80-6.61 (m, 2H), 3.69 (s, 3H), 2.23 (s, 3H), 1.22 (s, 9H) ppm; ^{13}C NMR (101 MHz, CD_2Cl_2) δ = 158.9, 150.4, 147.6, 137.4, 133.7, 131.6, 130.6, 129.1, 128.4, 128.35, 128.30, 127.1, 125.7, 125.6, 125.4, 125.3, 124.8, 124.4, 113.9, 55.2, 34.6, 31.1, 21.1 ppm; IR (ATR): ṽ = 2958, 1512, 1494, 1242, 1176, 1117, 1032, 946, 834, 818, 775, 699, 648, 627 cm^{-1}; HRMS (EI) *m/z* calcd. for $[C_{34}H_{32}O_2]^+$: 472.2402; found: 472.2402.

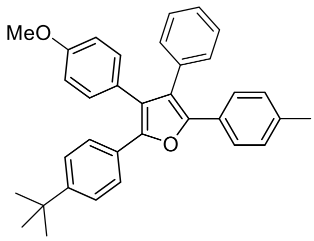

3-(4-(*tert*-Butyl)phenyl)-4-(4-methoxyphenyl)-5-phenyl-2-(*p*-tolyl)furan (3j). White solid (65.6 mg, 85%); mp: 213-214 °C; ^{1}H NMR (400 MHz, CD_2Cl_2) δ = 7.46-7.35 (m, 2H), 7.33-7.24 (m, 2H), 7.22-7.08 (m, 5H), 7.06-6.93 (m, 6H), 6.77-6.65 (m, 2H), 3.67 (s, 3H), 2.21 (s, 3H), 1.21 (s, 9H) ppm; ^{13}C NMR (101 MHz, CD_2Cl_2) δ = 158.9, 150.1, 147.9, 147.2, 137.3, 131.5, 131.4, 130.3, 130.0, 129.0, 128.31, 128.27, 127.1, 125.7, 125.54, 125.50, 125.2, 124.9, 124.6, 113.8, 55.1, 34.4, 31.1, 21.0 ppm; IR (ATR): ṽ = 2961, 1601, 1510, 1286, 1246, 1175, 1106, 1028, 944, 834, 821, 770, 691, 660, 638 cm^{-1}; HRMS (EI) *m/z* calcd. for $[C_{34}H_{32}O_2]^+$: 472.2402; found: 472.2398.

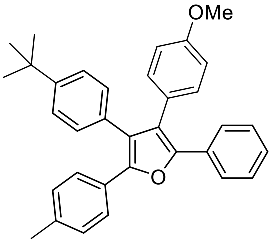

3-(4-(*tert*-Butyl)phenyl)-2-(4-methoxyphenyl)-5-phenyl-4-(*p*-tolyl)furan (3k). White solid (45.2 mg, 98%); mp: 173-175 °C; ^{1}H NMR (400 MHz, CD_2Cl_2) δ = 7.44-7.34 (m, 2H), 7.34-7.28 (m, 2H), 7.23-7.05 (m, 5H), 7.05-6.90 (m, 6H), 6.76-6.64 (m, 2H), 3.66 (s, 3H), 2.22 (s, 3H), 1.20 (s, 9H) ppm; ^{13}C NMR (101 MHz, CD_2Cl_2) δ = 159.0, 150.1, 147.9, 147.0, 136.9, 131.2, 130.4, 130.3, 130.2, 130.0, 129.1, 128.3, 127.2, 127.0, 125.6, 125.2, 125.1, 123.8, 123.7, 113.8, 55.2, 34.4, 31.1, 21.0 ppm; IR (ATR): ṽ = 2947, 2902, 1606, 1570, 1513, 1497, 1460, 1443, 1300, 1254, 1178, 1108, 1031, 946, 841, 831, 825, 756, 693, 677 cm^{-1}; HRMS (EI) *m/z* calcd. for $[C_{34}H_{32}O_2]^+$: 472.2402; found: 472.2404.

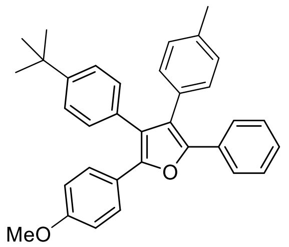

3-(4-(*tert*-Butyl)phenyl)-2-(4-methoxyphenyl)-4-phenyl-5-(*p*-tolyl)furan (3l). White solid (180 mg, 98%); mp: 157-158 °C; ^{1}H NMR (400 MHz, CD_2Cl_2) δ = 7.37-7.29 (m, 2H), 7.29-7.23 (m, 2H), 7.23-7.14 (m, 5H), 7.13-7.06 (m, 2H), 7.05-6.94 (m, 4H), 6.77-6.67 (m, 2H), 3.70 (s, 3H), 2.22 (s, 3H), 1.21 (s, 9H) ppm; ^{13}C NMR (101 MHz, CD_2Cl_2) δ = 158.9, 150.1, 147.5, 147.3, 137.1, 133.7, 130.5, 130.3, 130.0, 129.0, 128.3, 128.2, 127.1, 127.0, 125.6, 125.2, 124.5, 123.8, 123.6, 113.7, 55.2, 34.4, 31.0, 20.9 ppm; IR (ATR): ṽ = 2958, 2928, 1606, 1511, 1497, 1298, 1252, 1175, 1108, 1050, 1034, 946, 832, 824, 775, 700 cm^{-1}; HRMS (EI) *m/z* calcd. for $[C_{34}H_{32}O_2]^+$: 472.2402; found: 472.2401.

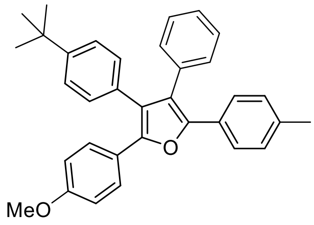

2-(4-Bromophenyl)-5-(4-(*tert*-butyl)phenyl)-4-(4-chlorophenyl)-3-(4-nitrophenyl)furan (5a). Yellow solid (66.4 mg, 87%); mp: 236-238 ^{o}C; ^{1}H NMR (400 MHz, CD_2Cl_2) δ = 8.11-7.94 (m, 2H), 7.39-7.30 (m, 4H), 7.29-7.23 (m, 5H), 7.23-7.16 (m, 3H), 7.05-6.98 (m, 2H), 1.22 (s, 9H) ppm; ^{13}C NMR (101 MHz, CD_2Cl_2) δ = 151.4, 149.2, 147.3, 147.2, 140.0, 133.6, 131.8, 131.7, 131.2, 131.0, 128.99, 128.96, 127.6, 127.1, 125.6, 125.5, 123.8, 123.4, 122.7, 121.9, 34.6, 30.9 ppm; IR (ATR): ṽ = 2961, 1601, 1516, 1484, 1397, 1341, 1285, 1268, 1120, 1102, 1089, 1073, 1045, 1014, 1006, 946, 862, 852, 836, 825, 778, 749, 738, 720, 699, 677, 656 cm^{-1}; HRMS (EI) *m/z* calcd. for $[C_{32}H_{25}BrClNO_3]^+$: 585.0706; found: 585.0707.

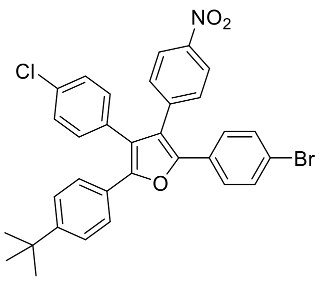

Methyl 4-(5-(4-(*tert*-butyl)phenyl)-4-(4-methoxyphenyl)-2-(naphthalen-2-yl)furan-3-yl)benzoate (5b). White solid (32.8 mg, 95%); mp: 195-197 ^{o}C; ^{1}H NMR (400 MHz, CD_2Cl_2) δ = 7.95 (s, 1H), 7.87-7.79 (m, 2H), 7.72-7.59 (m, 3H), 7.45-7.39 (m, 3H), 7.39-7.32 (m, 2H), 7.28-7.18 (m, 4H), 7.08-6.96 (m, 2H), 6.80-6.68 (m, 2H), 3.79 (s, 3H), 3.70 (s, 3H), 1.22 (s, 9H) ppm; ^{13}C NMR (101 MHz, CD_2Cl_2) δ = 166.7, 159.0, 150.7, 148.5, 147.7, 138.5, 133.3, 132.6, 131.5, 130.6, 129.5, 129.0, 128.1, 128.0, 127.94, 127.91, 127.6, 126.4, 126.2, 125.4, 124.9, 124.8, 124.7, 124.1, 123.8, 113.9, 55.1, 51.9, 34.5, 30.9 ppm; IR (ATR): ṽ = 2953, 1720, 1609, 1495, 1434, 1272, 1245, 1175, 1111, 1101, 1018, 957, 934, 833, 819, 748, 707, 619 cm^{-1}; HRMS (ESI) *m/z* calcd. for $[C_{39}H_{34}O_4+Na]^+$: 589.2349; found: 589.2345.

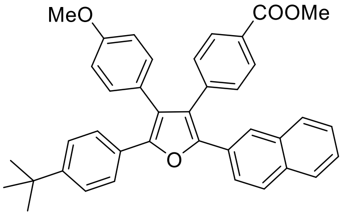

1-(4-(2-(4-Bromophenyl)-4-(4-ethylphenyl)-5-(4-methoxyphenyl)furan-3-yl)phenyl)ethan-1-one (5c). White solid (44.1 mg, quant.); mp: 182-183 ^{o}C; ^{1}H NMR (400 MHz, CD_2Cl_2) δ = 7.81-7.65 (m, 2H), 7.39-7.27 (m, 4H), 7.27-7.20 (m, 2H), 7.20-7.12 (m, 2H), 7.07-6.90 (m, 4H), 6.78-6.66 (m, 2H), 3.68 (s, 3H), 2.54 (q, J = 8.0 Hz, 2H), 2.46 (s, 3H), 1.12 (t, J = 8.0 Hz, 3H) ppm; ^{13}C NMR (101 MHz, CD_2Cl_2) δ = 197.4, 159.3, 148.5, 146.3, 143.5, 138.3, 136.0, 131.6, 130.5, 130.2, 129.8, 129.6, 128.4, 127.9, 127.3, 124.8, 123.4, 123.3, 121.2, 113.8, 55.2, 28.5, 26.4, 15.0 ppm; IR (ATR): ṽ = 2964, 1681, 1609, 1518, 1495, 1301, 1251, 1175, 1104, 1007, 945, 829, 801, 594 cm^{-1}; HRMS (ESI) *m/z* calcd. for $[C_{33}H_{27}BrO_3+Na]^+$: 573.1036; found: 573.1032.

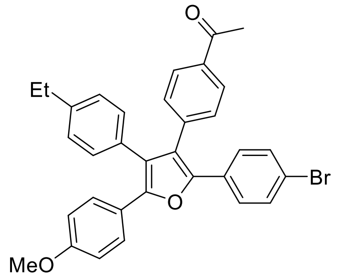

3-(4-Bromophenyl)-5-(4-(*tert*-butyl)phenyl)-4-(4-chlorophenyl)-2-(4-fluorophenyl)furan (5d). White solid (105 mg, quant.); mp: 207-209 ^{o}C; ^{1}H NMR (400 MHz, CD_2Cl_2) δ = 7.41-7.34 (m, 2H), 7.34-7.27 (m, 4H), 7.27-7.20 (m, 2H), 7.20-7.13 (m, 2H), 7.06-6.97 (m, 2H), 6.97-6.84 (m, 4H), 1.21 (s, 9H) ppm; ^{13}C NMR (101 MHz, CD_2Cl_2) δ = 163.4, 160.9, 151.0, 148.3, 146.9, 133.2, 132.0, 131.9, 131.82, 131.80, 131.6, 128.8, 127.7 (d, J =8 Hz), 127.5, 126.8 (d, J = 4 Hz), 125.5 (d, J = 3 Hz), 123.3, 122.9, 121.5, 115.5 (d, J = 21 Hz), 34.5, 30.9 ppm; IR (ATR): ṽ = 2963, 2866, 1505, 1486, 1228, 1156, 1117, 1093, 1074, 1013, 943, 834, 824, 738, 686, 618, 557 cm^{-1}; HRMS (EI) *m/z* calcd. for $[C_{32}H_{25}BrClFO]^+$: 558.0761; found: 558.0764.

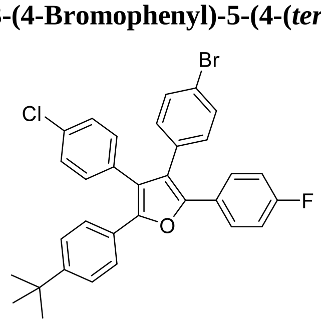

3-(4-Chlorophenyl)-5-(3,4-dimethoxyphenyl)-4-(3,5-dimethylphenyl)-2-(naphthalen-2-yl)furan (5e)
White solid (32.6 mg, quant.); mp: 191-193 °C; ^{1}H NMR (400 MHz, CD_2Cl_2) δ = 7.94 (s, 1H), 7.71-7.60 (m, 3H), 7.46-7.40 (m, 1H), 7.40-7.32 (m, 2H), 7.21-7.14 (m, 3H), 7.13-7.06 (m, 2H), 6.90-6.81 (m, 2H), 6.80-6.70 (m, 3H), 3.73 (s, 3H), 3.45 (s, 3H), 2.15 (s, 6H) ppm; ^{13}C NMR (101 MHz, CD_2Cl_2) δ = 148.78, 148.76, 148.1, 147.2, 138.1, 133.4, 133.1, 133.0, 132.5, 132.1, 131.8, 129.0, 128.5, 128.3, 128.2, 128.1, 127.9, 127.6, 126.4, 126.1, 124.5, 124.2, 123.8, 123.7, 118.1, 111.3, 109.1, 55.7, 55.2, 20.9 ppm; IR (ATR): ṽ = 2952, 2930, 1591, 1508, 1462, 1268, 1250, 1226, 1140, 1089, 1025, 1015, 891, 855, 808, 739, 695 cm^{-1}; HRMS (ESI) *m/z* calcd. for $[C_{36}H_{29}ClO_3+Na]^+$: 567.1697; found: 567.1694.

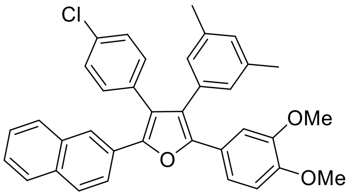

3-(4-Chlorophenyl)-2-(2-methoxyphenyl)-4-(3-methoxyphenyl)-5-(thiophen-2-yl)furan (5f). White solid (28.7 mg, quant.); mp: 163-164 °C; ^{1}H NMR (400 MHz, CD_2Cl_2) δ = 7.44-7.35 (m, 1H), 7.30-7.23 (m, 1H), 7.22-7.14 (m, 1H), 7.13-7.07 (m, 1H), 7.06-6.98 (m, 3H), 6.96-6.69 (m, 8H), 3.63 (s, 3H), 3.34 (s, 3H) ppm; ^{13}C NMR (101 MHz, CD_2Cl_2) δ = 159.7, 156.7, 146.1, 144.8, 133.8, 132.9, 132.4, 132.1, 130.9, 130.5, 130.1, 129.6, 127.8, 127.2, 124.7, 124.6, 123.8, 122.9, 122.7, 120.5, 119.6, 116.0, 113.4, 111.5, 55.2, 54.8 ppm; IR (ATR): ṽ = 2934, 2833, 1609, 1580, 1498, 1482, 1461, 1432, 1282, 1246, 1229, 1089, 1044, 1021, 966, 928, 832, 747, 696 cm^{-1}; HRMS (ESI) *m/z* calcd. for $[C_{28}H_{21}ClO_3S+Na]^+$: 495.0792; found: 495.0788.

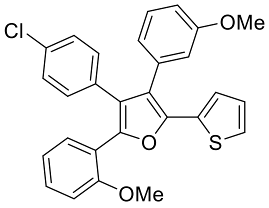

3-(Benzo[b]thiophen-2-yl)-5-(benzo[b]thiophen-3-yl)-4-(4-chlorophenyl)-2-(thiophen-2-yl)furan (5g). Yellow solid (47.2 mg, 90%); mp: 202-203 °C; ^{1}H NMR (400 MHz, CD_2Cl_2) δ = 8.17-8.10 (m, 1H), 7.83-7.77 (m, 1H), 7.75-7.65 (m, 2H), 7.37-7.19 (m, 7H), 7.19-7.16 (m, 1H), 7.16-7.10 (m, 4H), 6.96-6.86 (m, 1H) ppm; ^{13}C NMR (101 MHz, CD_2Cl_2) δ = 146.1, 145.1, 141.0, 139.9, 139.8, 136.9, 133.5, 133.3, 132.0, 131.3, 130.8, 128.7, 127.5, 126.0, 125.9, 125.7, 125.5, 125.0, 124.9, 124.83, 124.81, 124.5, 124.3, 124.0, 123.7, 122.7, 122.2, 116.0 ppm; IR (ATR): ṽ = 3056, 2924, 2852, 1730, 1577, 1486, 1422, 1124, 1086, 1013, 991, 951, 835, 823, 760, 747, 736, 696, 517 cm^{-1}; HRMS (ESI) *m/z* calcd. for $[C_{30}H_{17}ClOS_3+H]^+$: 524.0130; found: 524.0127.

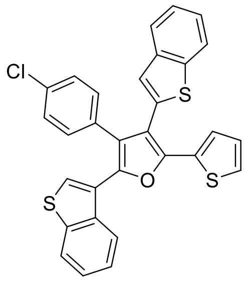

(*E*)-1-(4-(4-(4-Chlorophenyl)-3-phenyl-5-(*p*-tolyl)furan-2-yl)phenyl)-2-phenyldiazene (5h). Red solid (51.3 mg, 98%); mp: 248-249 °C; ^{1}H NMR (400 MHz, THF-d_8) δ = 8.00-7.85 (m, 2H), 7.85-7.76 (m, 2H), 7.70-7.60 (m, 2H), 7.54-7.39 (m, 5H), 7.36-7.28 (m, 3H), 7.28-7.19 (m, 4H), 7.18-7.08 (m, 4H), 2.32 (s, 3H) ppm; ^{13}C NMR (101 MHz, THF-d_8) δ = 153.5, 152.0, 149.9, 147.6, 138.6, 133.9, 133.8, 133.6, 132.6, 132.5, 131.7, 131.0, 129.8, 129.7, 129.4, 129.3, 128.4, 128.3, 127.5, 126.7, 126.6, 124.4, 123.7, 123.4, 21.1 ppm; IR (ATR): ṽ = 3068, 3027, 2952, 2919, 1599, 1508, 1485, 1441, 1382, 1225, 1152, 1088, 1016, 945, 848, 838, 817, 764, 699, 684 cm^{-1}; HRMS (ESI) *m/z* calcd. for $[C_{35}H_{25}ClN_2O+H]^+$: 525.1728; found: 525.1726.

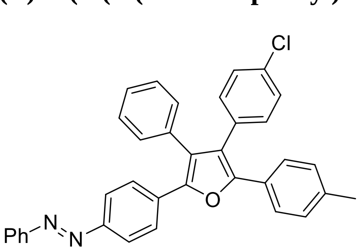

3-Phenyl-5-(*p*-tolyl)-4-(3,4,5-trimethoxyphenyl)-2,2'-bifuran (5i). White solid (22 mg, 94%); mp: 118-120 °C; ^{1}H NMR (400 MHz, CD_2Cl_2) δ = 7.46-7.35 (m, 2H), 7.31 (s, 1H), 7.27-7.10 (m, 5H), 7.10-6.98 (m, 2H), 6.52-6.07 (m, 4H), 3.68 (s, 3H), 3.47 (s, 6H), 2.25 (s, 3H) ppm; ^{13}C NMR (101 MHz, CD_2Cl_2) δ = 153.2, 148.0, 146.1, 142.0, 140.5, 137.7, 137.3, 132.4, 130.3, 129.0, 128.0, 127.9, 127.7, 127.3, 125.8, 124.6, 123.7, 111.2, 107.6, 106.9, 60.5, 55.9, 21.0 ppm; IR (ATR): ṽ = 2939, 2831, 1582, 1500, 1460, 1408, 1295, 1235, 1125, 1003, 961, 896, 843, 821, 779, 744, 725, 711, 699 cm^{-1}; HRMS (ESI) *m/z* calcd. for $[C_{30}H_{26}O_5+Na]^+$: 489.1672; found: 489.1670.

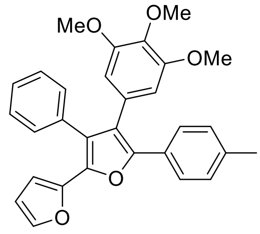

2-(4-Bromophenyl)-5-(4-(*tert*-butyl)phenyl)-4-(4-chlorophenyl)-3-(*p*-tolyl)furan (5j). White solid (245 mg, 88%); mp: 208-210 °C; ^{1}H NMR (400 MHz, CD_2Cl_2) δ = 7.46-7.35 (m, 6H), 7.34-7.30 (m, 2H), 7.29-7.22 (m, 2H), 7.20-7.08 (m, 4H), 7.07-7.01 (m, 2H), 2.34 (s, 3H), 1.30 (s, 9H) ppm; ^{13}C NMR (101 MHz, CD_2Cl_2) δ = 150.9, 148.3, 146.4, 137.4, 133.0, 131.9, 131.8, 131.4, 130.0, 129.8, 129.5, 129.3, 128.6, 127.6, 127.0, 125.6, 125.5, 125.4, 123.6, 120.9, 34.5, 30.9, 21.0 ppm; IR (ATR): ṽ = 2957, 2867, 1511, 1485, 1362, 1267, 1118, 1069, 1008, 942, 820, 765, 681, 518, 499 cm^{-1}; HRMS (EI) *m/z* calcd. for $C_{32}H_{28}BrClO$: 554.1012; found: 554.1016.

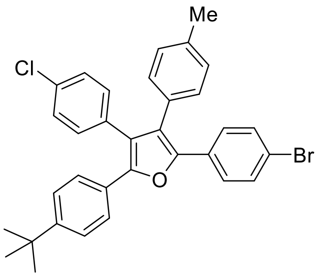

Derivatives

Deborylation. 3-Phenyl-2-(*m*-tolyl)-5-(*p*-tolyl)furan (7). A flame-dried Schlenk flask was charged with 3-borylfuran **4k** (45 mg, 0.1 mmol) and THF (1.0 mL). Pd(dppf)Cl_2 (3.6 mg, 5 mol %) and aq. KOH (9 M, 66.5 µL, 0.6 mmol) were added under Ar and the resulting mixture was stirred at room temperature for 12 h. After evaporation of all volatile materials, the crude product was purified by flash chromatography on silica to give the title compound as a white solid (30.7 mg, 95%); mp: 109-110 °C; ^{1}H NMR (400 MHz, CD_2Cl_2) δ = 7.65-7.50 (m, 2H), 7.46-6.86 (m, 11H), 6.70 (s, 1H), 2.29 (s, 3H), 2.22 (s, 3H) ppm; ^{13}C NMR (101 MHz, CD_2Cl_2) δ = 152.6, 147.7, 138.2, 137.6, 134.4, 131.0, 129.4, 128.6, 128.5, 128.25, 128.20, 127.8, 127.2, 126.6, 124.4, 123.7, 123.2, 108.7, 21.2, 21.0 ppm; IR (ATR): ṽ = 3024, 2918, 1603, 1498, 1142, 1050, 932, 808, 762, 698, 688 cm^{-1}; HRMS (EI) *m/z* calcd. for $[C_{24}H_{20}O]^+$: 324.1514; found: 324.1515.

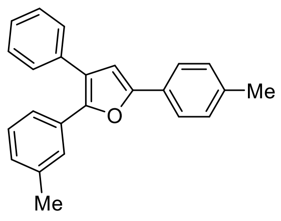

2-(4-(*tert*-Butyl)phenyl)-3-(4-methoxyphenyl)-4-phenyl-1-(*p*-tolyl)-1,4-dihydro-1,4-epoxynaphthalene (11). KF (23 mg, 0.4 mmol) was added to a solution of furan **3j** (43 mg, 0.091 mmol), 18-crown-6 (105 mg, 0.4 mmol) and 2-(trimethylsilyl)-phenyl-trifluormethansulfonate (**10**) (44 µL, 0.8 mmol) in THF (0.5 mL) under Argon. The mixture was stirred at room temperature for 12 h, all volatile materials were evaporated, and the residue was purified by flash chromatography on silica to afford the title compound as a white solid (45.1 mg, 90%). mp: 220-221 °C; ^{1}H NMR (400 MHz, CD_2Cl_2) δ = 7.45-7.39 (m, 1H), 7.39-7.28 (m, 5H), 7.27-7.20 (m, 3H), 7.11-6.96 (m, 6H), 6.67-6.60 (m, 2H), 6.59-6.48 (m, 4H), 3.59 (s, 3H), 2.26 (s, 3H), 1.11 (s, 9H) ppm; ^{13}C NMR (101 MHz, CD_2Cl_2) δ = 158.9, 152.7, 151.8, 151.6, 151.3, 150.1, 138.2, 135.0, 131.9, 131.4, 129.6, 128.96, 128.89, 128.1, 128.0, 127.9, 127.4, 126.7, 125.0, 124.96, 124.7, 120.8, 120.2, 113.4, 94.2, 93.9, 55.0, 34.4, 31.0, 21.0 ppm; IR (ATR): ṽ = 2962, 1599, 1500, 1451, 1285, 1242, 1173, 1107, 1021, 944, 837, 816, 755, 744, 701,

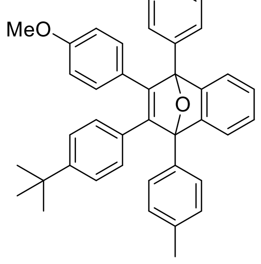

680, 643, 600 cm^{-1}; HRMS (ESI) *m/z* calcd. for $[C_{40}H_{36}O_2+H]^+$: 549.2788; found: 549.2789.

2-(4-(*tert*-Butyl)phenyl)-3-(4-methoxyphenyl)-4-phenyl-1-(*p*-tolyl)naphthalene (12). A solution of titanium tetrachloride (1 M in CH_2Cl_2, 0.39 mL) was added to a solution of lithium aluminum hydride (5.9 mg, 0.156 mmol) in THF (0.5 mL) and triethylamine (7.5 µL, 0.06 mmol). The resulting mixture was stirred at room temperature for 10 min and then at reflux temperature for 30 min. After cooling to room temperature, a solution of **11** (31 mg, 0.056 mmol) in THF (0.50 mL) was added and stirring continued for 24 h. The reaction was quenched with sat. aq. K_2CO_3 at 0 °C and the product was extracted with MTBE. The combined organic layers were dried over Na_2SO_4, the solvent was evaporated and the residue purified by flash chromatography (SiO_2, hexanes/CH_2Cl_2 = 4:1) to provide the title compound as a white solid material (16 mg, 53%); mp: 200-201 °C; ^1H NMR (400 MHz, CD_2Cl_2) δ = 7.51-7.42 (m, 2H), 7.29-7.23 (m, 2H), 7.21-7.10 (m, 5H), 7.03-6.95 (m, 4H), 6.85-6.79 (m, 2H), 6.71-6.61 (m, 4H), 6.36-6.23 (m, 2H), 3.49 (s, 3H), 2.23 (s, 3H), 1.05 (s, 9H) ppm; ^{13}C NMR (101 MHz, CD_2Cl_2) δ = 157.1, 148.0, 140.0, 139.3, 138.7, 138.3, 138.2, 137.9, 136.8, 135.9, 133.1, 132.3, 132.2, 132.0, 131.3, 131.1, 131.0, 128.1, 127.5, 126.8, 126.7, 126.3, 125.6, 125.5, 123.3, 111.8, 54.8, 34.0, 30.9, 20.9 ppm; IR (ATR): ṽ = 2961, 1610, 1511, 1462, 1372, 1287, 1244, 1178, 1108, 1038, 829, 807, 785, 768, 702, 653 cm^{-1}; HRMS (ESI) *m/z* calcd. for $[C_{40}H_{36}O+Na]^+$: 555.2658; found: 555.2664.

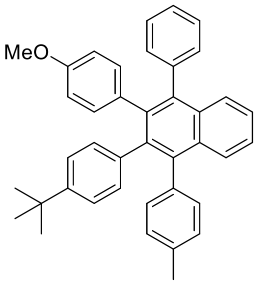

(Z)-1-(4-Bromophenyl)-4-(4-(*tert*-butyl)phenyl)-3-(4-chlorophenyl)-2-(*p*-tolyl)but-2-ene-1,4-dione (13). Furan **5j** (100 mg, 0.18 mmol) and potassium nitrate (30 mg, 0.27 mmol) were suspended in aqueous acetic acid (80 % *v/v*, 1 mL) under O_2 atmosphere and the mixture was stirred at 100 °C bath temperature for 30 min. The mixture was cooled, diluted with cold water, and the resulting pale-yellow solid material was filtered off and dried under vacuum (94 mg, 91%). mp: 186-188 °C; ^1H NMR (400 MHz, CD_2Cl_2) δ = 7.68-7.60 (m, 2H), 7.60-7.53 (m, 2H), 7.43-7.33 (m, 2H), 7.32-7.24 (m, 2H), 7.16-7.07 (m, 2H), 7.07-7.00 (m, 2H), 7.00-6.86 (m, 4H), 2.19 (s, 3H), 1.19 (s, 9H) ppm; ^{13}C NMR (101 MHz, CD_2Cl_2) δ = 196.0, 195.6, 157.2, 144.5, 143.4, 139.1, 135.2, 134.3, 134.1, 133.6, 131.6, 131.3, 131.1, 129.8, 129.6, 129.5, 128.9, 128.0, 125.4, 35.0, 30.7, 21.0 ppm; IR (ATR): ṽ = 2967, 1668, 1602, 1485, 1396, 1260, 1088, 1007, 822, 797, 783, 721 cm^{-1}; HRMS (EI) *m/z* calcd. for $C_{33}H_{28}BrClO_2$: 570.0961; found: 570.0959.

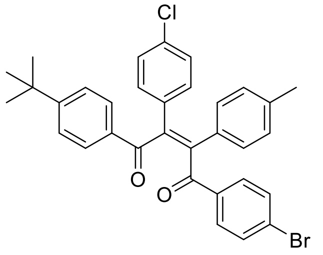

2-(4-Bromophenyl)-5-(4-(*tert*-butyl)phenyl)-4-(4-chlorophenyl)-1-methyl-3-(*p*-tolyl)-1*H*-pyrrole (14). Methylamine (40% in MeOH, 0.01 mL, 0.08 mmol) was added to a solution of diketone **13** (12 mg, 0.021 mmol) in MeOH (0.5 mL). The reaction was stirred at reflux temperature for 30 min before it was cooled to room temperature. $NaBH_4$ (0.55 M in diglyme, 20 µL, 0.011 mmol) was then slowly added, and the mixture was stirred for another 30 min. The solution was evaporated and the residue was purified by flash chromatography (SiO_2, hexanes/CH_2Cl_2 = 5:1) to provide the title compound as a white solid material (8 mg, 68%). mp: 255-256 °C; ^1H NMR (400 MHz, CD_2Cl_2) δ = 7.44-7.34 (m, 2H), 7.33-7.26 (m, 2H), 7.19-7.06 (m, 4H), 6.99-6.89 (m, 2H), 6.87-6.78 (m, 4H), 6.78-6.70 (m, 2H), 3.28 (s, 3H), 2.15 (s, 3H), 1.24 (s, 9H) ppm; ^{13}C NMR (101 MHz, CD_2Cl_2) δ = 150.6, 135.2, 134.7, 132.8, 132.4, 132.3, 132.1, 131.9, 131.3, 130.8, 130.7, 130.6, 130.3, 129.3, 128.4, 127.5, 125.2, 122.5, 121.3, 120.9, 34.5, 32.9, 31.0, 20.7 ppm; IR (ATR): ṽ = 2960, 2923, 1508, 1483, 1392, 1360, 1264, 1108, 1073, 1011, 846, 830,

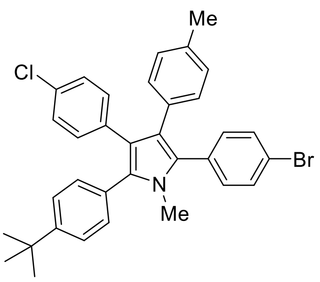

821, 808, 764, 561, 514 cm^{-1}; HRMS (ESI) *m/z* calcd. for $[C_{34}H_{31}ClNBr]^+$: 567.1323; found: 567.1324.

3-(4-Bromophenyl)-6-(4-(*tert*-butyl)phenyl)-5-(4-chlorophenyl)-4-(*p*-tolyl)pyridazine (15). Diketone **13** (15 mg, 0.026 mmol) and $N_2H_4{\cdot}H_2O$ (5 µL, 0.1 mmol) were suspended in dry MeOH (0.5 mL) and the resulting mixture was stirred at reflux temperature for 1 h. After reaching ambient temperature, the mixture was purified by flash chromatography (SiO_2, hexanes/EtOAc) to provide the title compound as a white solid material (13.5 mg, 91%). mp: 237-238 °C; ^1H NMR (400 MHz, CD_2Cl_2) δ = 7.36-7.28 (m, 2H), 7.26-7.14 (m, 6H), 7.04-6.95 (m, 2H), 6.90-6.82 (m, 2H), 6.79-6.72 (m, 2H), 6.70-6.60 (m, 2H), 2.17 (s, 3H), 1.22 (s, 9H) ppm; ^{13}C NMR (101 MHz, CD_2Cl_2) δ = 159.0, 158.1, 151.9, 138.3, 138.0, 137.2, 137.0, 134.5, 134.2, 133.6, 131.9, 131.8, 131.7, 131.1, 130.2, 129.8, 128.9, 128.2, 125.1, 122.8, 34.7, 31.1, 21.1 ppm; IR (ATR): ṽ = 2967, 1482, 1464, 1396, 1370, 1265, 1090, 1071, 1010, 842, 826, 805, 736, 704 cm^{-1}; HRMS (ESI) *m/z* calcd. for $[C_{33}H_{28}BrClN_2+H]^+$: 567.1197; found: 567.1202.

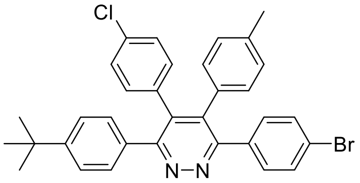

^{1}H and ^{13}C NMR Spectra

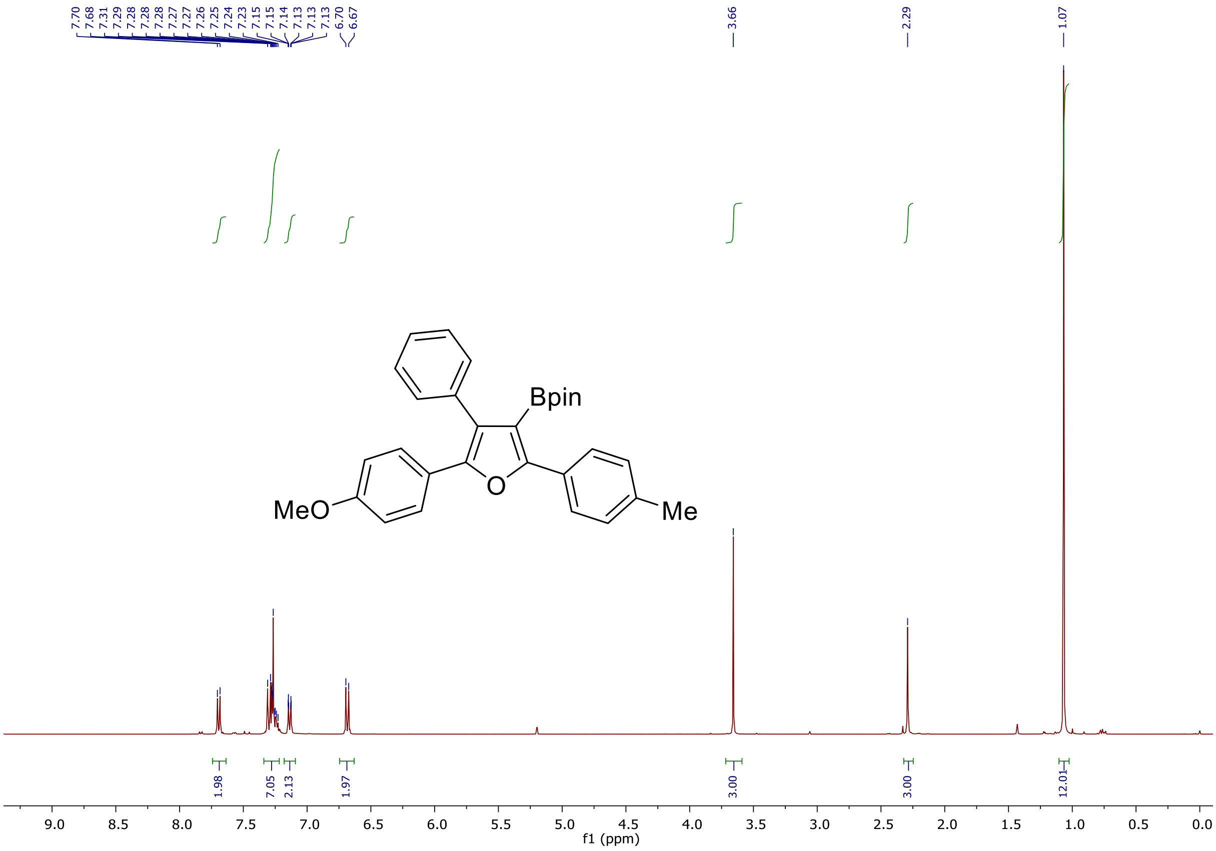

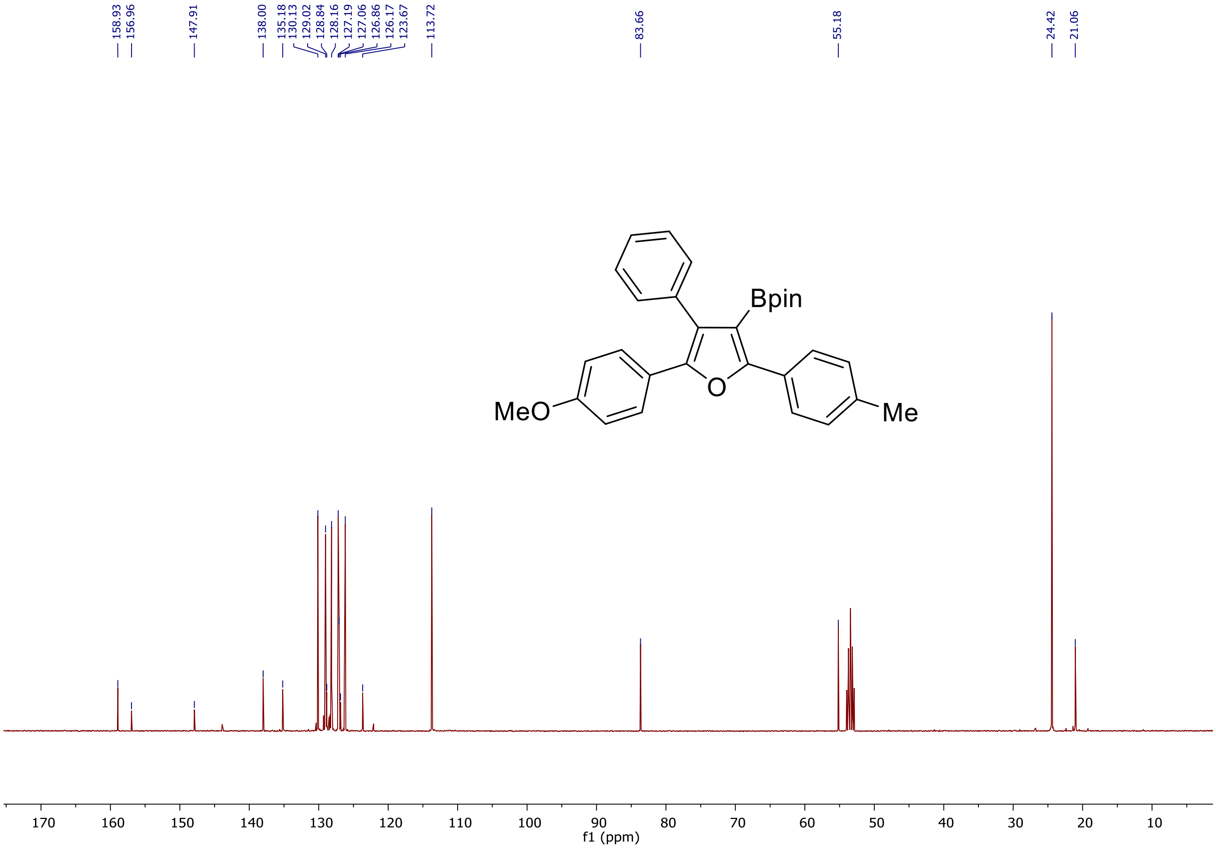

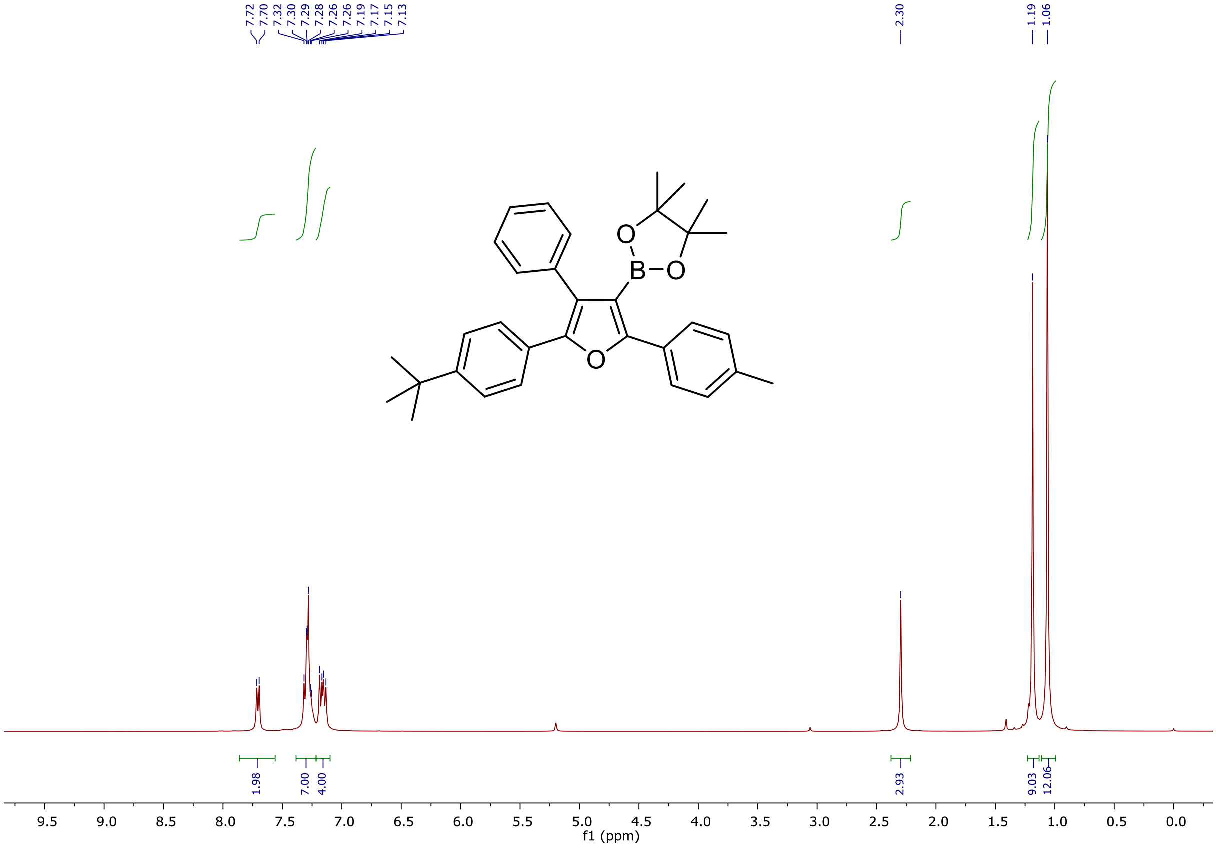

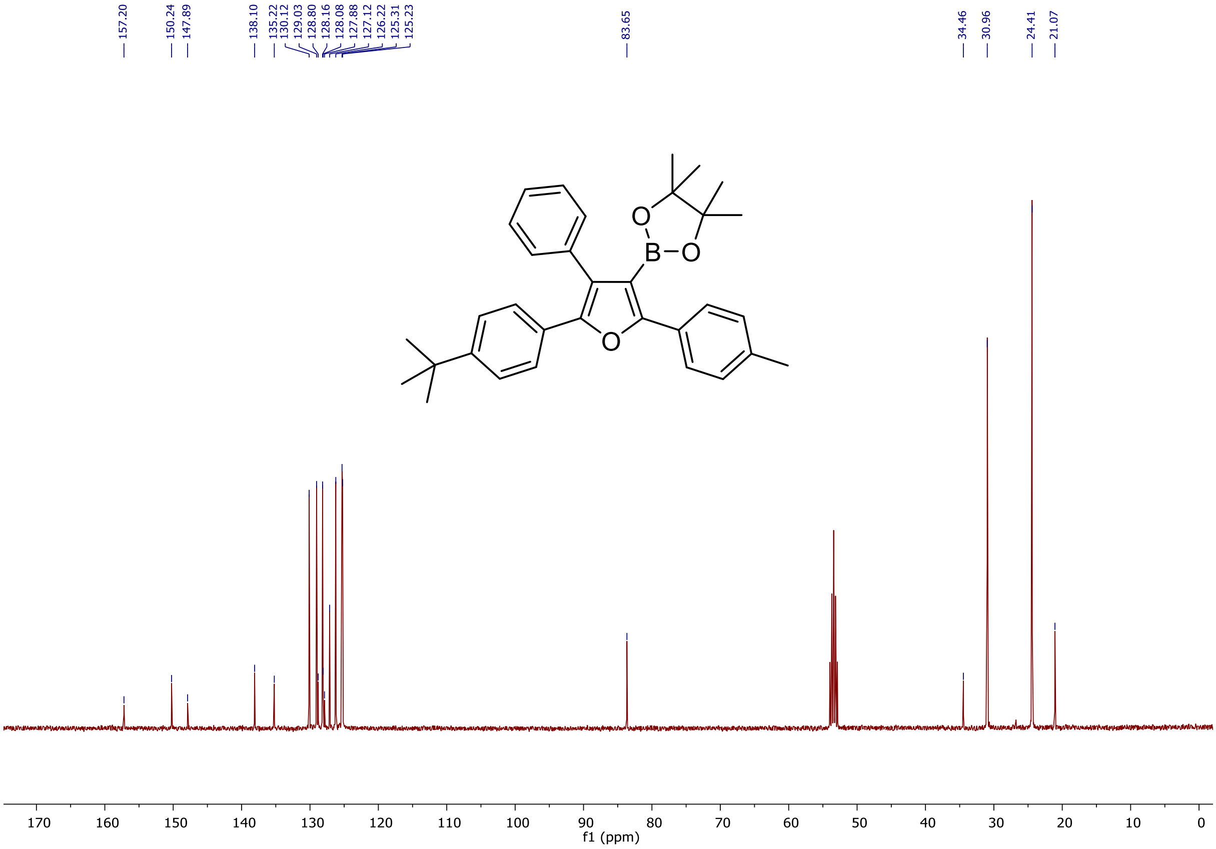

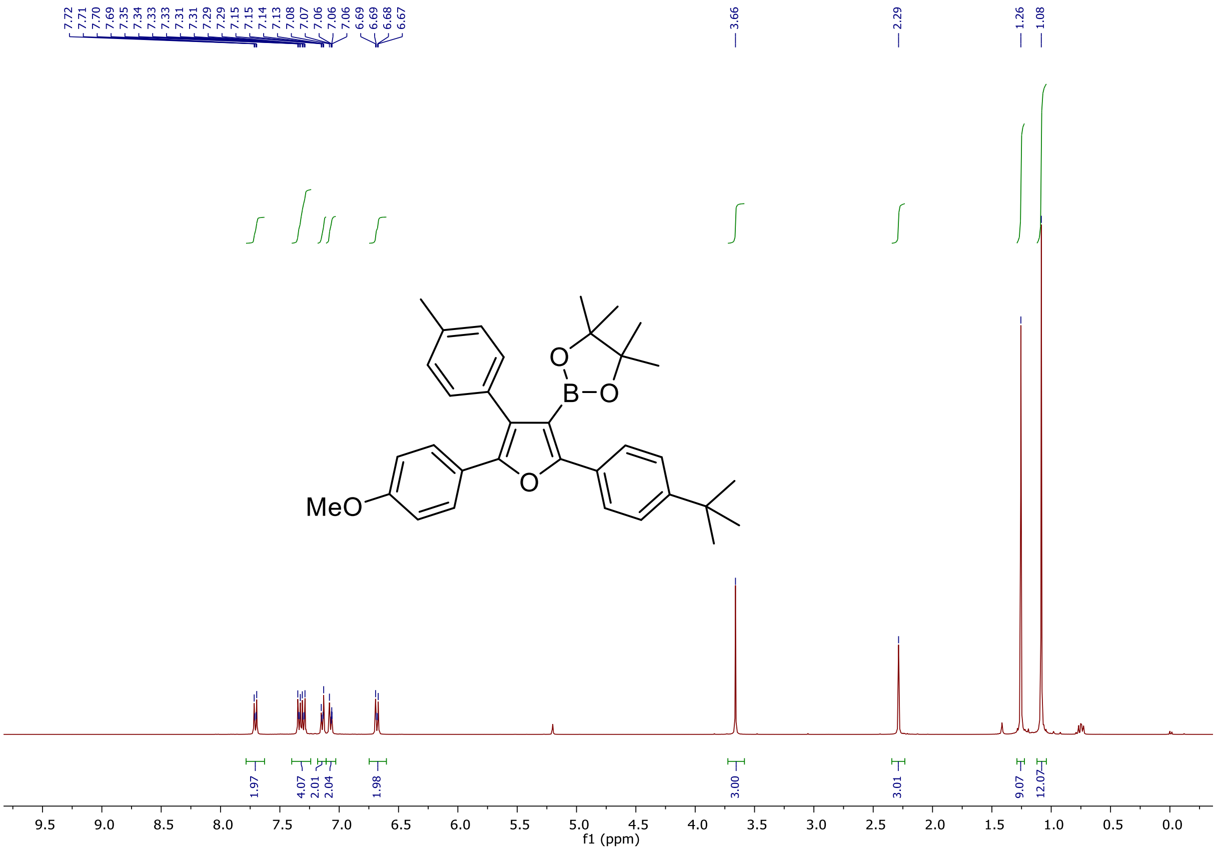
7.72 7.71 7.70 7.69 7.35 7.34 7.33 7.31 7.31 7.29 7.29 7.15 7.15 7.14 7.13 7.08 7.07 7.06 7.06 7.06 6.69 6.69 6.68 6.67
3.66
2.29
1.26
1.08
MeO
O
B
1.97 4.07 2.01 2.04 1.98 3.00 3.01 9.07 12.07
9.5 9.0 8.5 8.0 7.5 7.0 6.5 6.0 5.5 5.0 4.5 4.0 3.5 3.0 2.5 2.0 1.5 1.0 0.5 0.0
f1 (ppm)

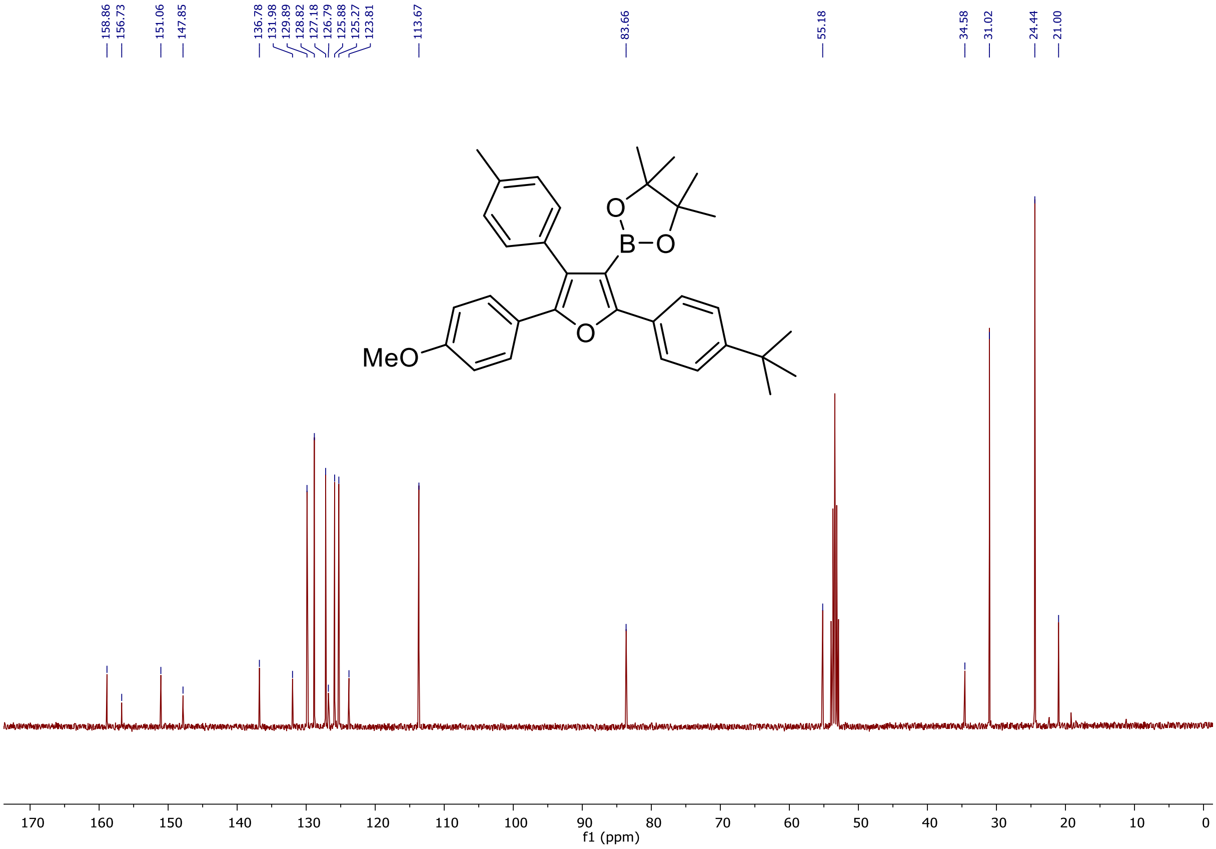
158.86 156.73 151.06 147.85
136.78 131.98 129.89 128.82 127.18 126.79 125.88 125.27 123.81
113.67
83.66
55.18
34.58 31.02 24.44 21.00
MeO
O
B
170 160 150 140 130 120 110 100 90 80 70 60 50 40 30 20 10 0
f1 (ppm)

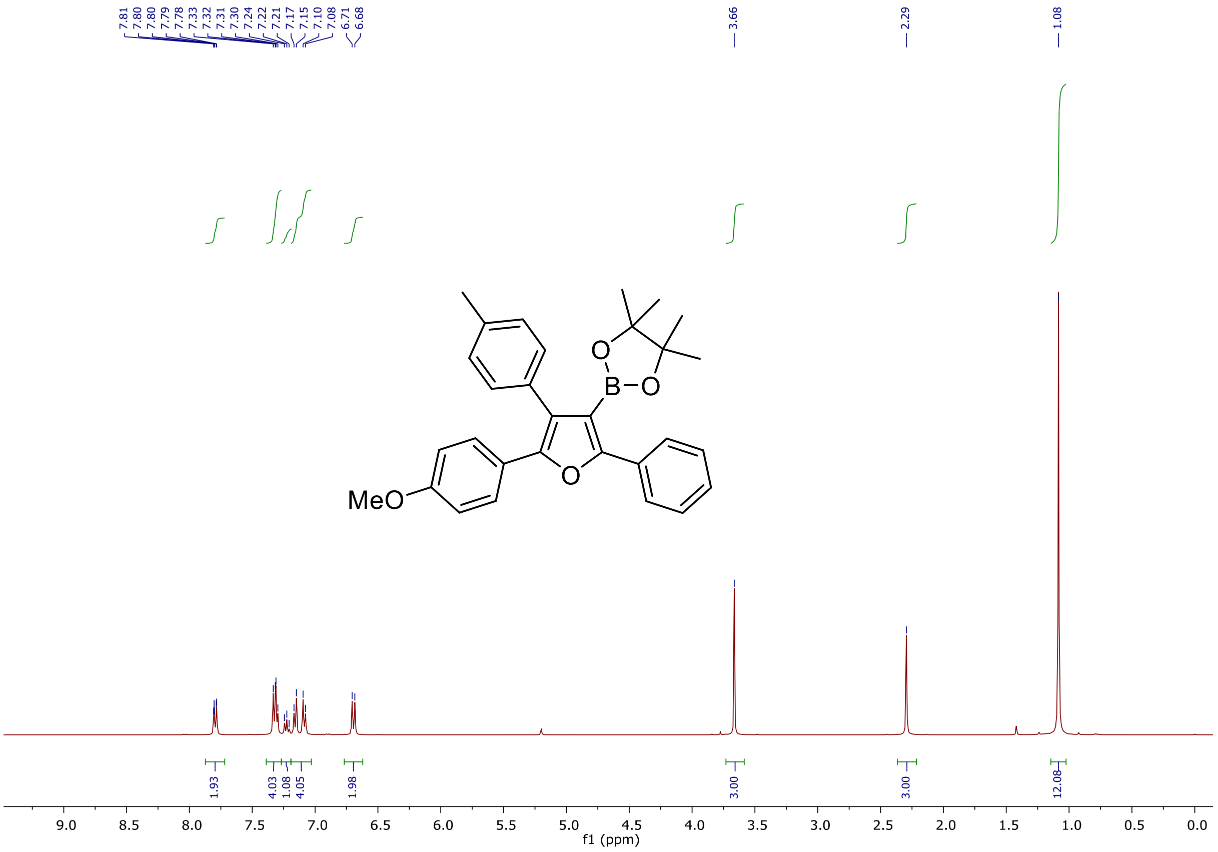

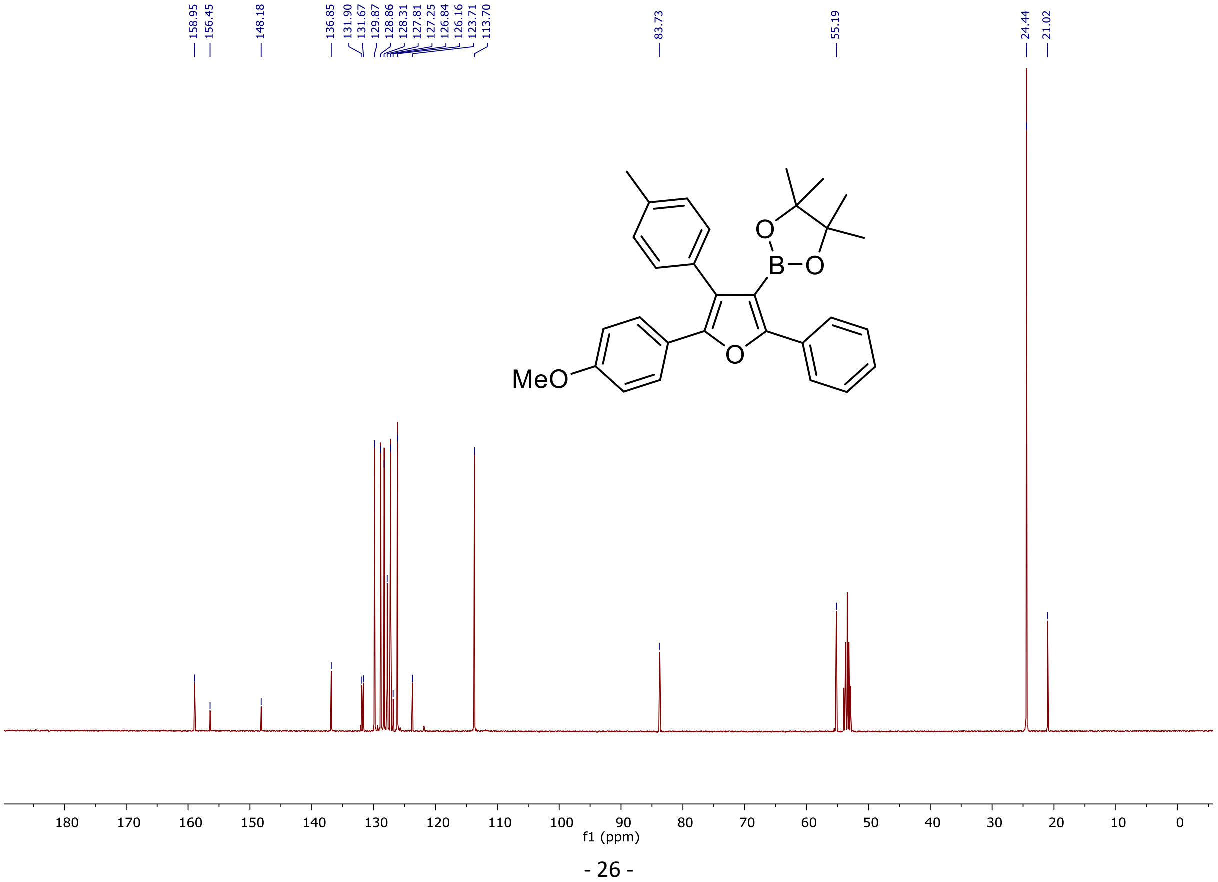

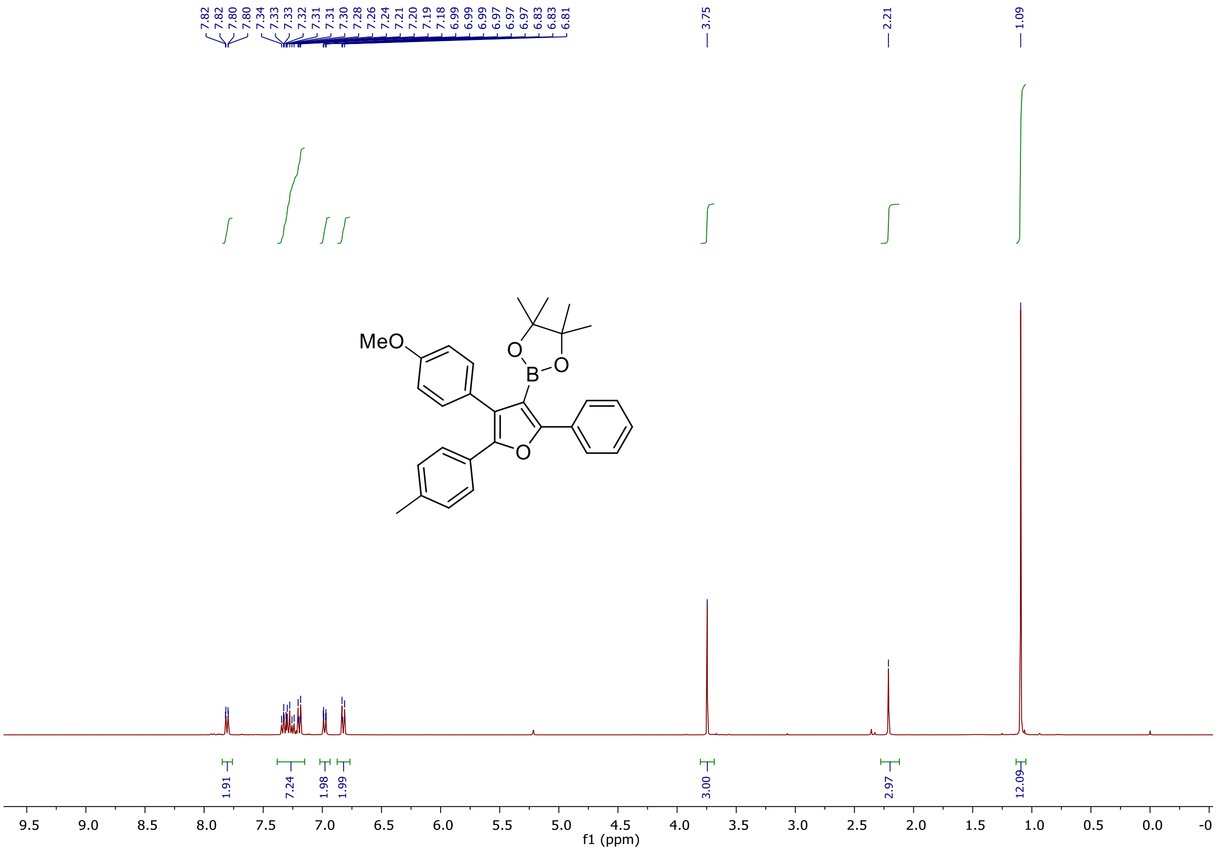
7.82
7.82
7.80
7.80
7.34
7.33
7.33
7.32
7.31
7.31
7.30
7.28
7.26
7.24
7.21
7.20
7.19
7.18
6.99
6.99
6.99
6.97
6.97
6.97
6.83
6.83
6.81
3.75
2.21
1.09
MeO
O
B
O
O
1.91
7.24
1.98
1.99
3.00
2.97
12.09
f1 (ppm)

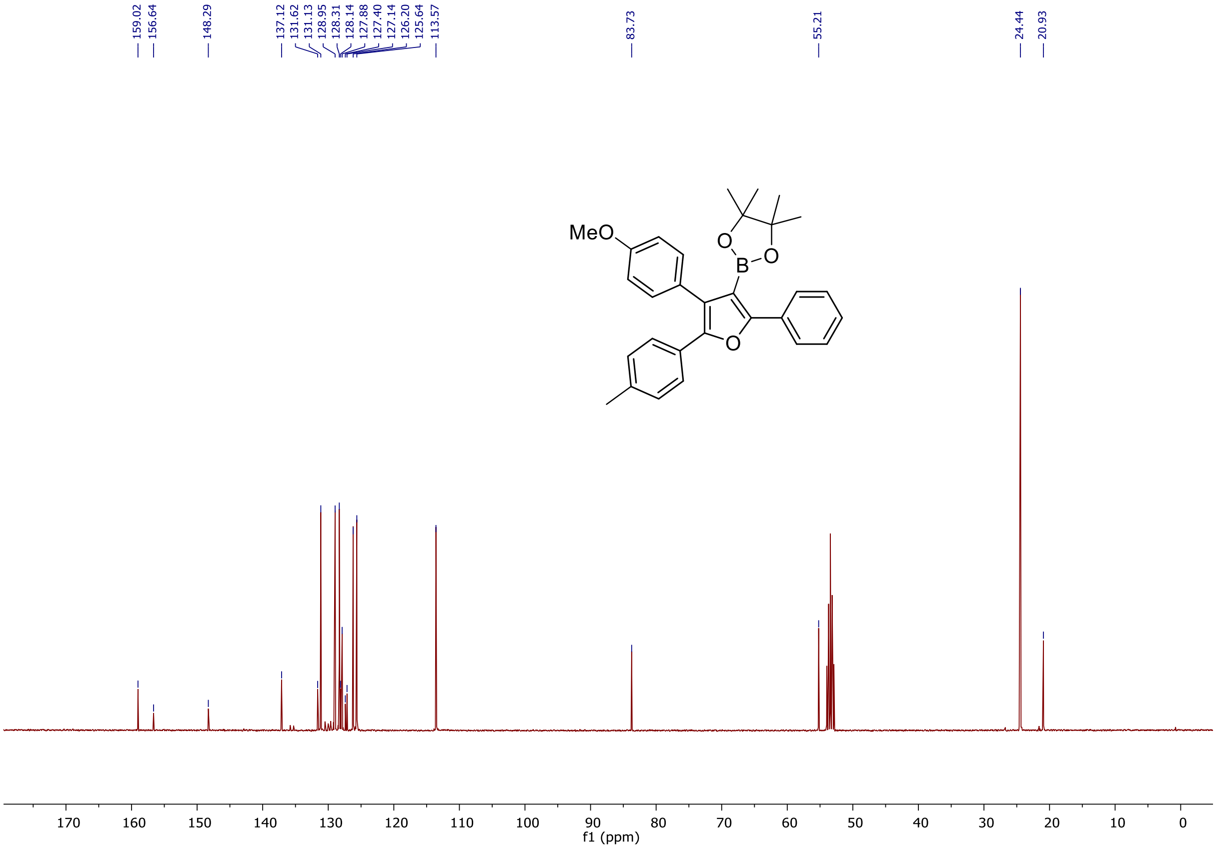
159.02
156.64
148.29
137.12
131.62
131.13
128.95
128.31
128.14
127.88
127.40
127.14
126.20
125.64
113.57
83.73
55.21
24.44
20.93
MeO
O
B
O
O
f1 (ppm)

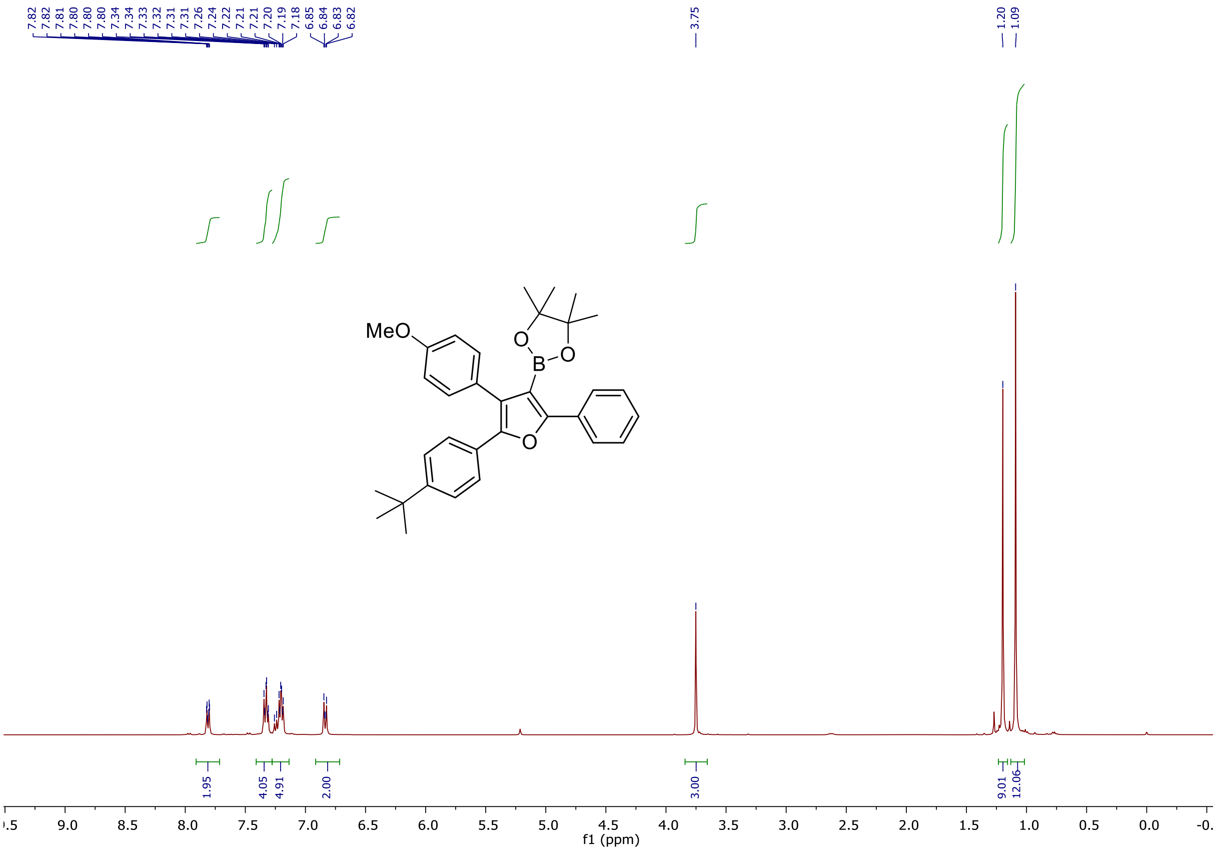

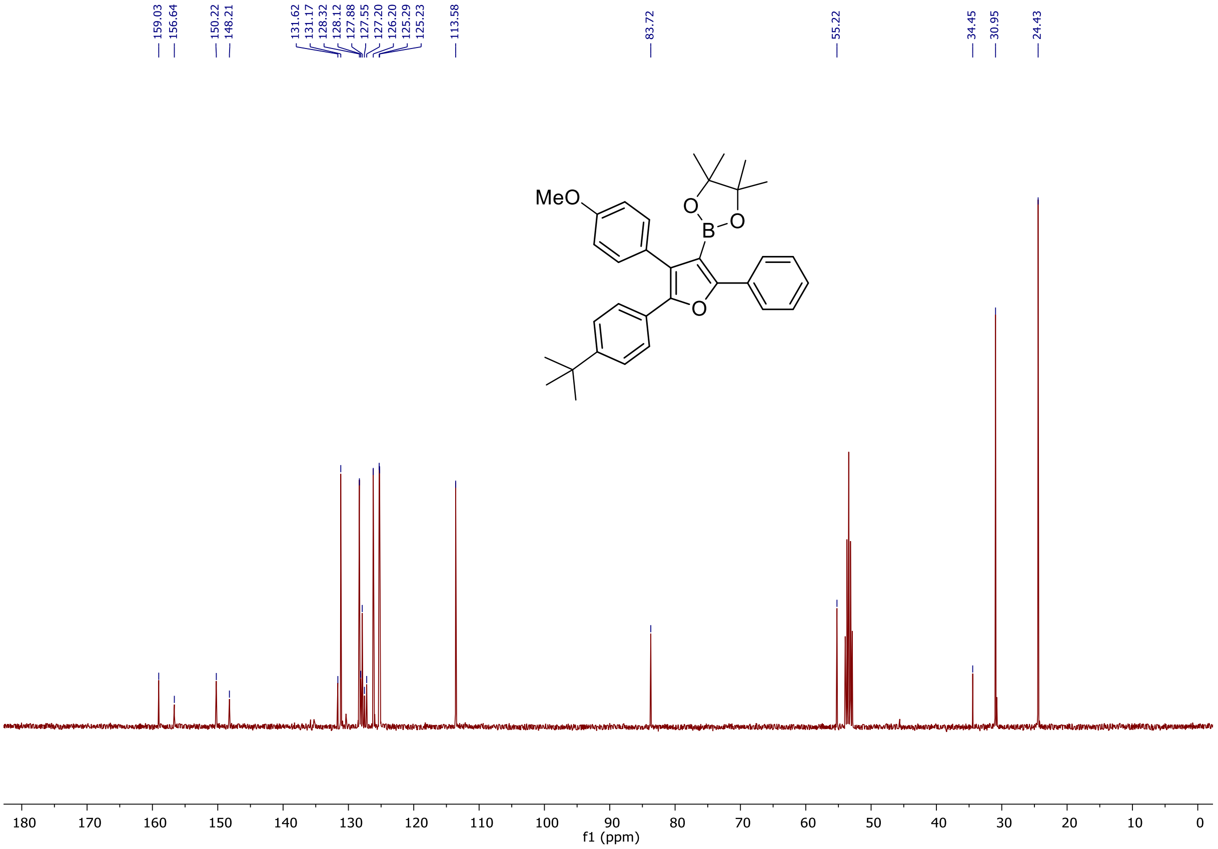

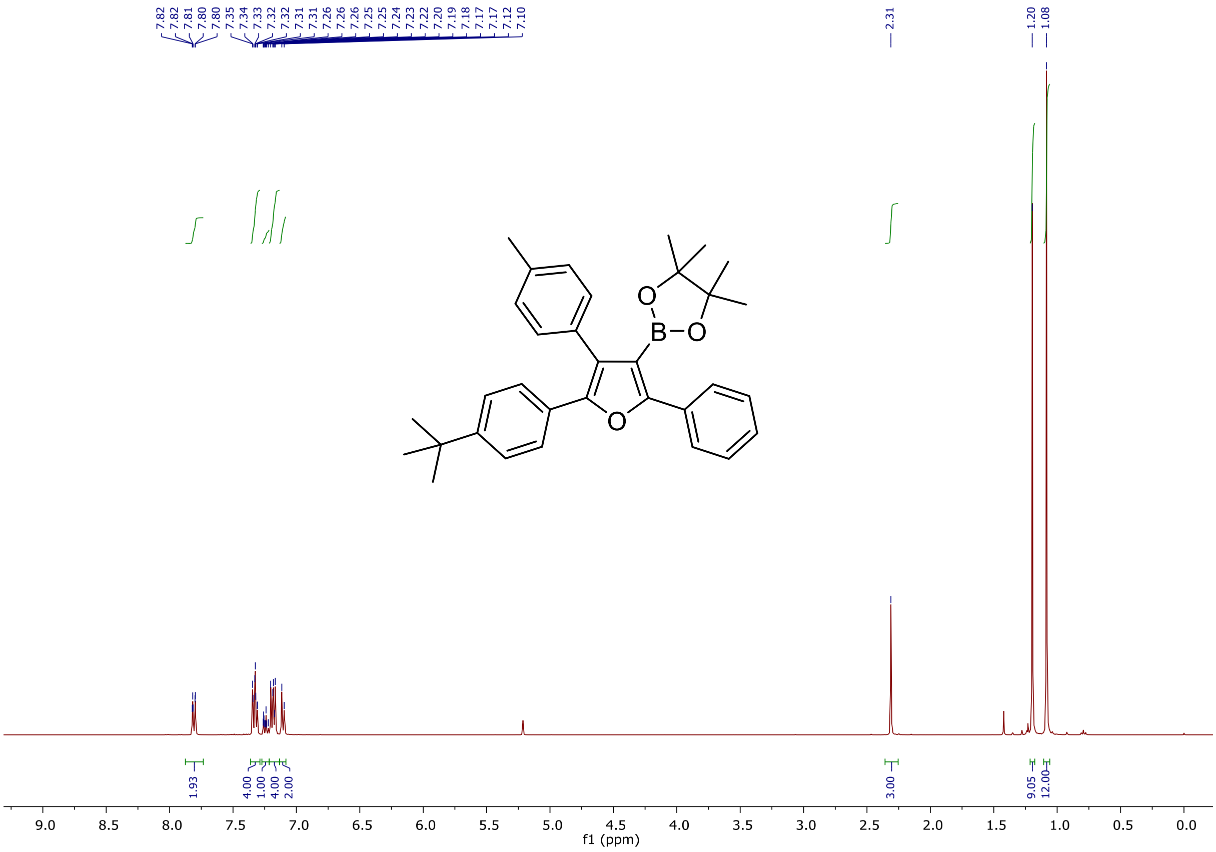
7.82
7.82
7.81
7.80
7.80
7.35
7.34
7.33
7.32
7.32
7.31
7.31
7.26
7.26
7.25
7.25
7.24
7.23
7.22
7.20
7.19
7.18
7.17
7.17
7.12
7.10
2.31
1.20
1.08
1.93
4.00
1.00
4.00
2.00
3.00
9.05
12.00
9.0
8.5
8.0
7.5
7.0
6.5
6.0
5.5
5.0
4.5
4.0
3.5
3.0
2.5
2.0
1.5
1.0
0.5
0.0
f1 (ppm)

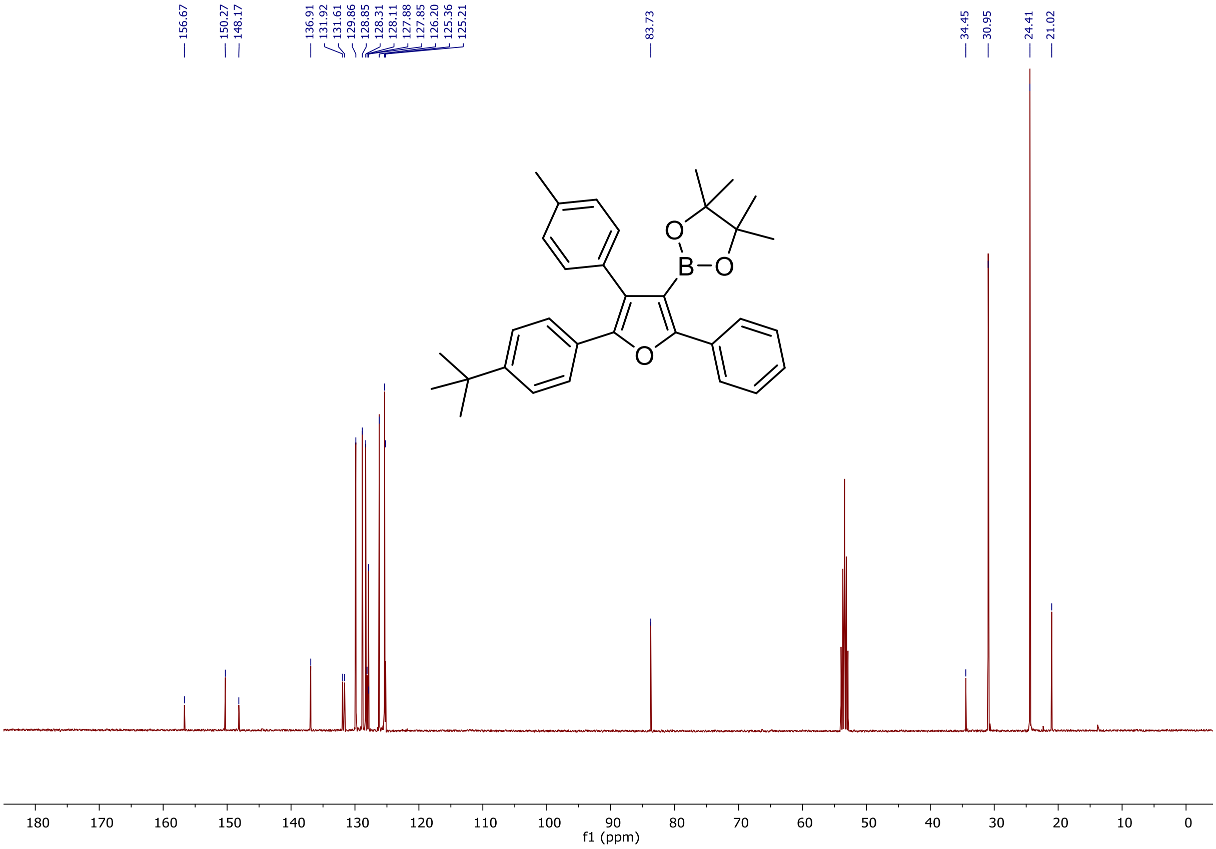
156.67
150.27
148.17
136.91
131.92
131.61
129.86
128.85
128.31
128.11
127.88
127.85
126.20
125.36
125.21
83.73
34.45
30.95
24.41
21.02
180
170
160
150
140
130
120
110
100
90
80
70
60
50
40
30
20
10
0
f1 (ppm)

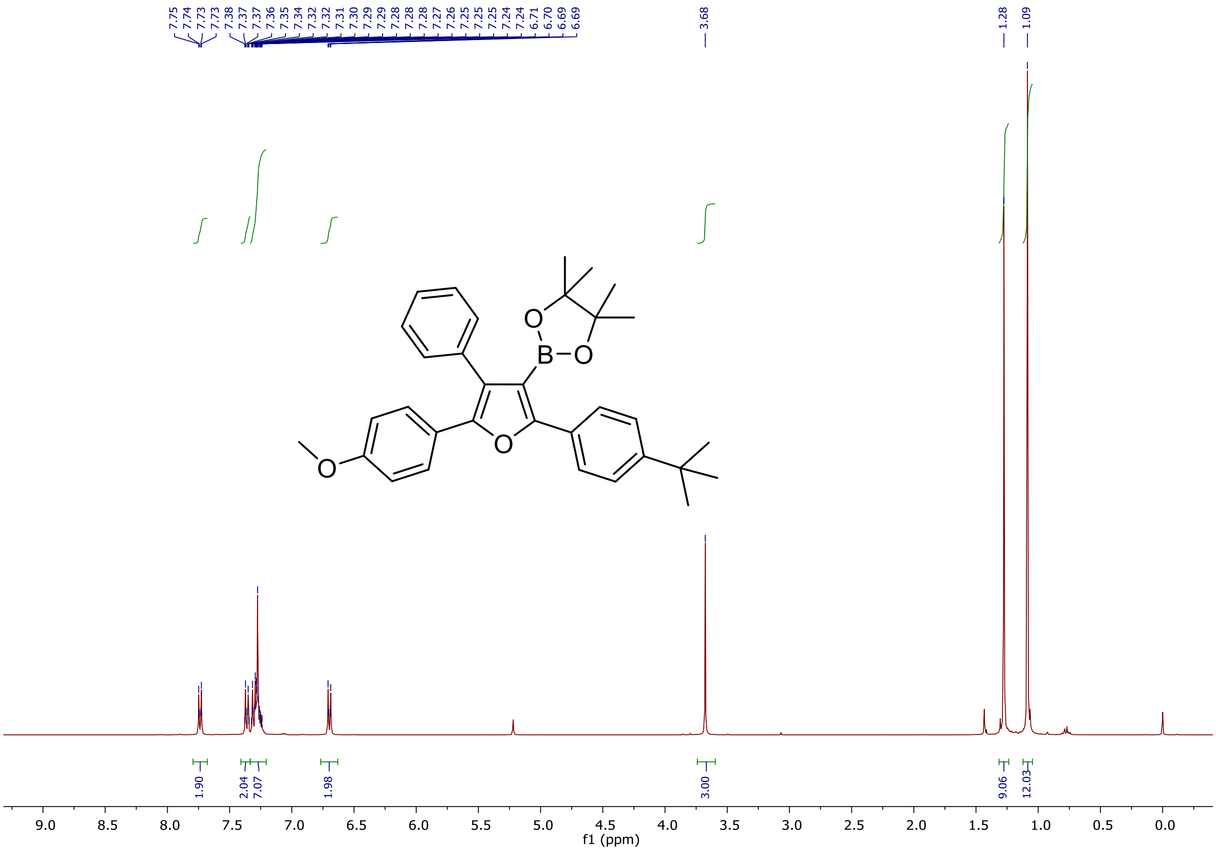
7.75
7.74
7.73
7.73
7.38
7.37
7.37
7.36
7.35
7.34
7.32
7.32
7.32
7.31
7.30
7.29
7.29
7.28
7.28
7.28
7.27
7.26
7.25
7.25
7.25
7.24
7.24
6.71
6.70
6.69
6.69
3.68
1.28
1.09
O
B
O
O
O
1.90
2.04
7.07
1.98
3.00
9.06
12.03
f1 (ppm)

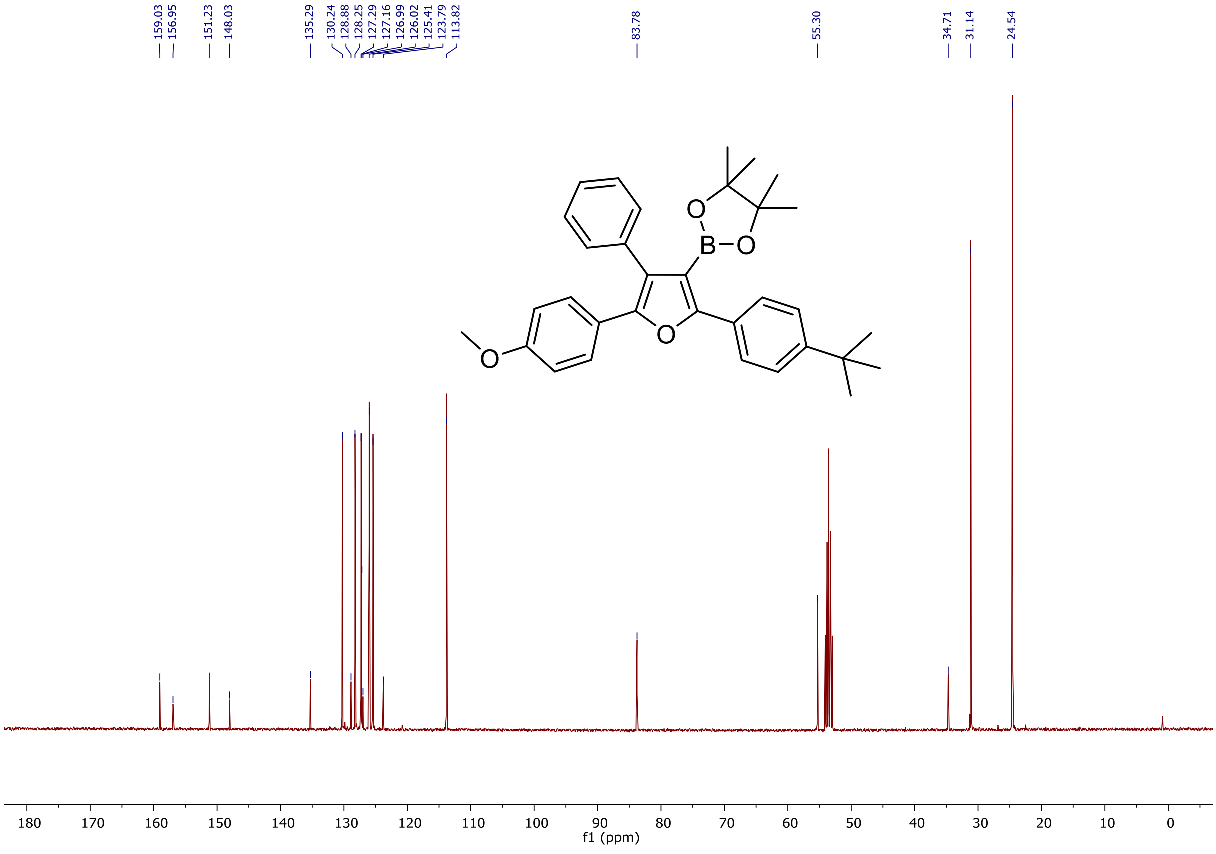
159.03
156.95
151.23
148.03
135.29
130.24
128.88
128.25
127.29
127.16
126.99
126.02
125.41
123.79
113.82
83.78
55.30
34.71
31.14
24.54
O
B
O
O
O
f1 (ppm)

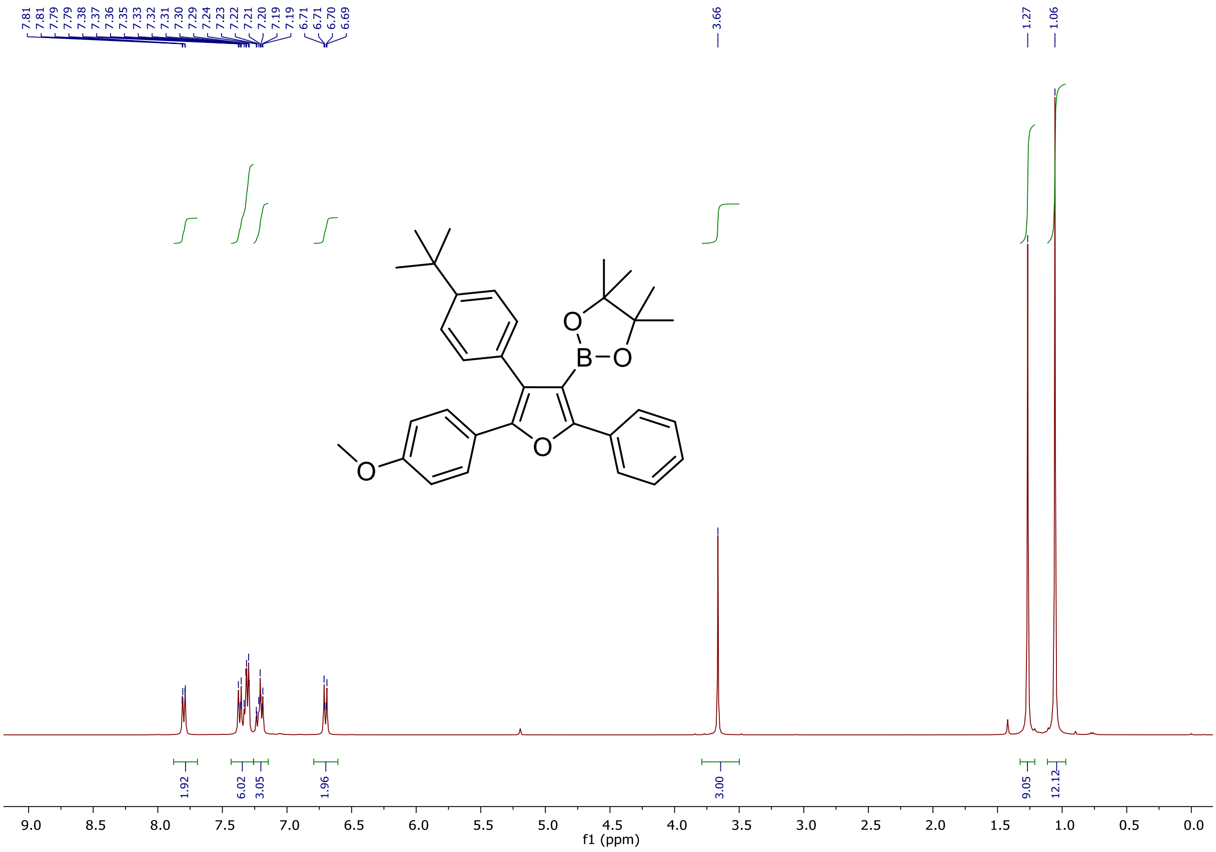
7.81 7.81 7.79 7.79 7.38 7.37 7.36 7.35 7.33 7.32 7.31 7.30 7.29 7.24 7.23 7.22 7.21 7.20 7.19 7.19 6.71 6.71 6.70 6.69
3.66
1.27
1.06
1.92
6.02
3.05
1.96
3.00
9.05
12.12
9.0 8.5 8.0 7.5 7.0 6.5 6.0 5.5 5.0 4.5 4.0 3.5 3.0 2.5 2.0 1.5 1.0 0.5 0.0
f1 (ppm)

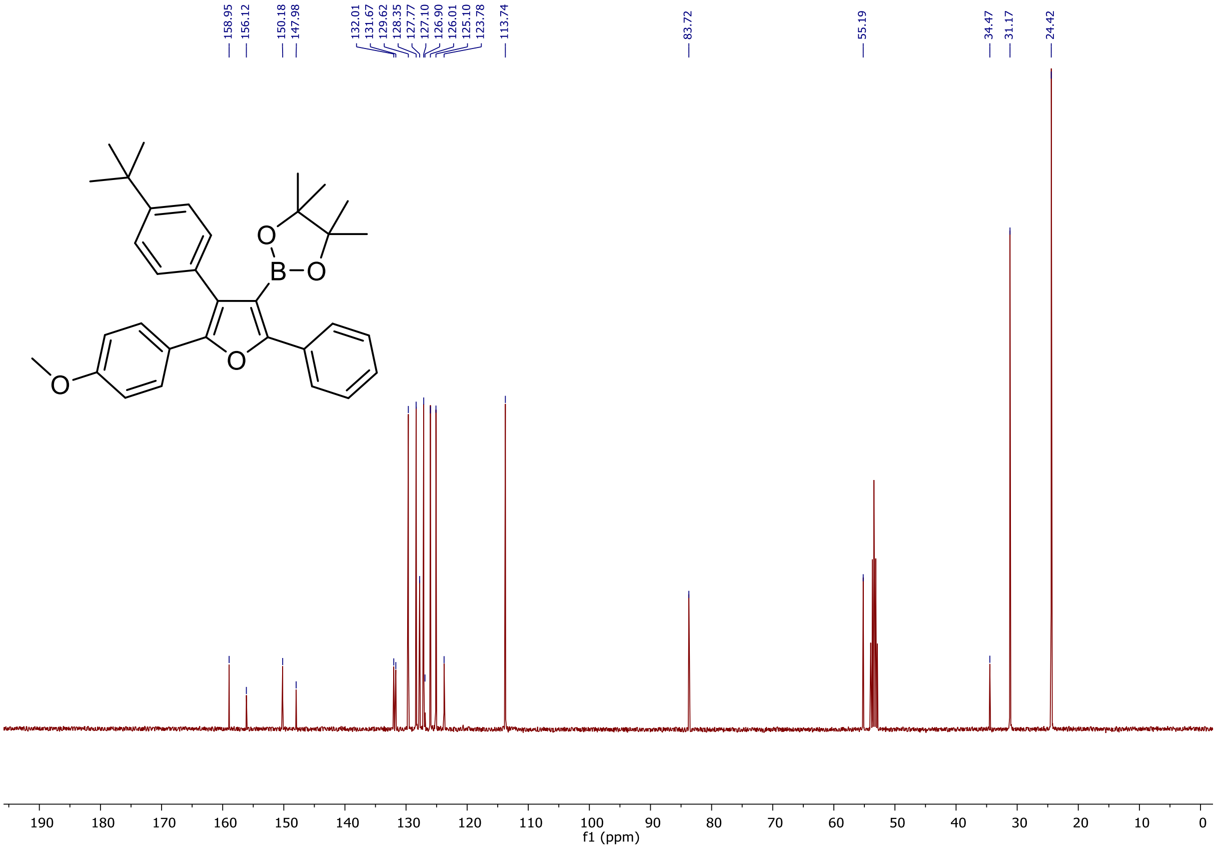
158.95
156.12
150.18
147.98
132.01 131.67 129.62 128.35 127.77 127.10 126.90 126.01 125.10 123.78
113.74
83.72
55.19
34.47
31.17
24.42
190 180 170 160 150 140 130 120 110 100 90 80 70 60 50 40 30 20 10 0
f1 (ppm)

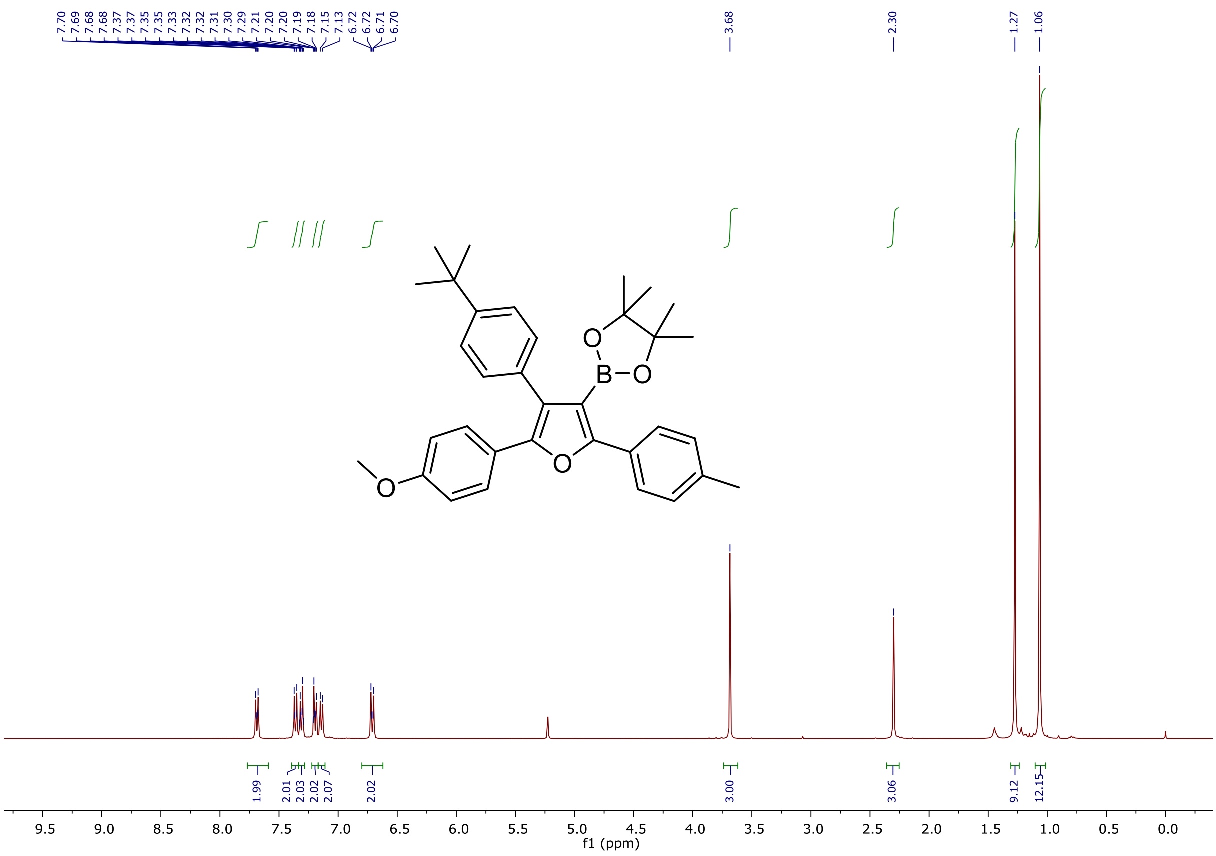
7.70 7.69 7.68 7.68 7.37 7.37 7.35 7.35 7.33 7.32 7.32 7.31 7.30 7.29 7.21 7.20 7.20 7.19 7.18 7.15 7.13 6.72 6.72 6.71 6.70
3.68
2.30
1.27
1.06
1.99 2.01 2.03 2.02 2.07 2.02 3.00 3.06 9.12 12.15
O
B
O
O
O
f1 (ppm)

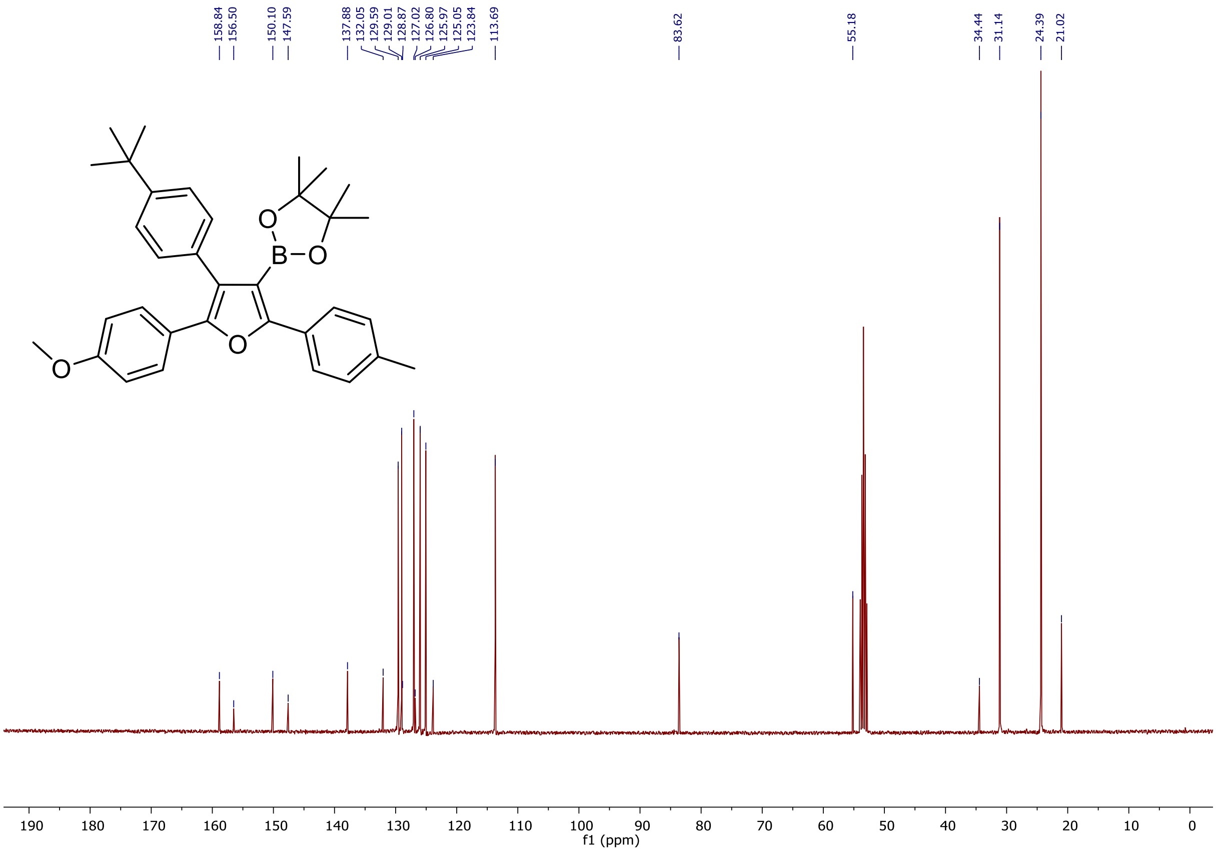
158.84 156.50 150.10 147.59 137.88 132.05 129.59 129.01 128.87 127.02 126.80 125.97 125.05 123.84 113.69
83.62
55.18
34.44 31.14 24.39 21.02
O
B
O
O
O
f1 (ppm)

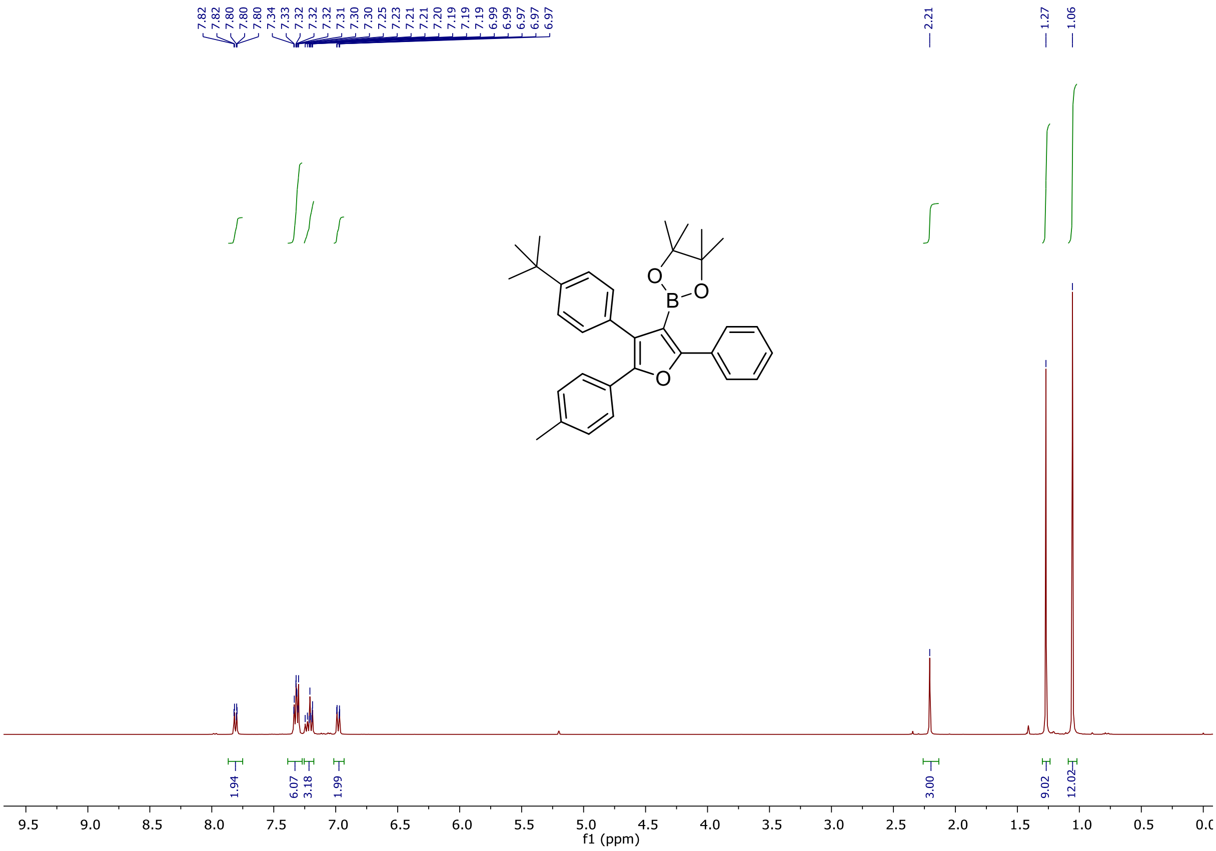
7.82 7.82 7.80 7.80 7.80 7.34 7.33 7.32 7.32 7.32 7.31 7.30 7.30 7.25 7.23 7.21 7.21 7.20 7.19 7.19 7.19 6.99 6.99 6.97 6.97 6.97
2.21
1.27
1.06
1.94
6.07
3.18
1.99
3.00
9.02
12.02
O
B
O
O
f1 (ppm)

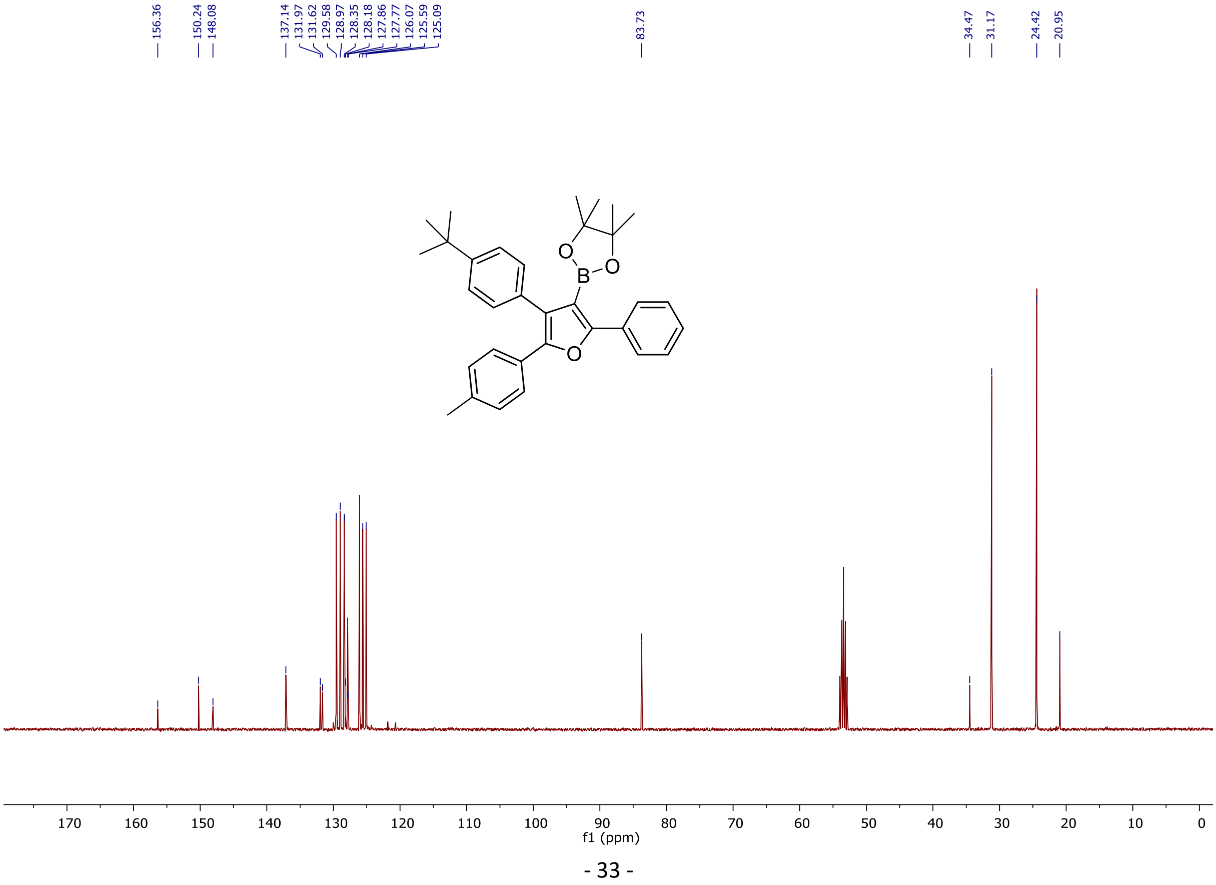
156.36
150.24
148.08
137.14 131.97 131.62 129.58 128.97 128.35 128.18 127.86 127.77 126.07 125.59 125.09
83.73
34.47
31.17
24.42
20.95
O
B
O
O
f1 (ppm)

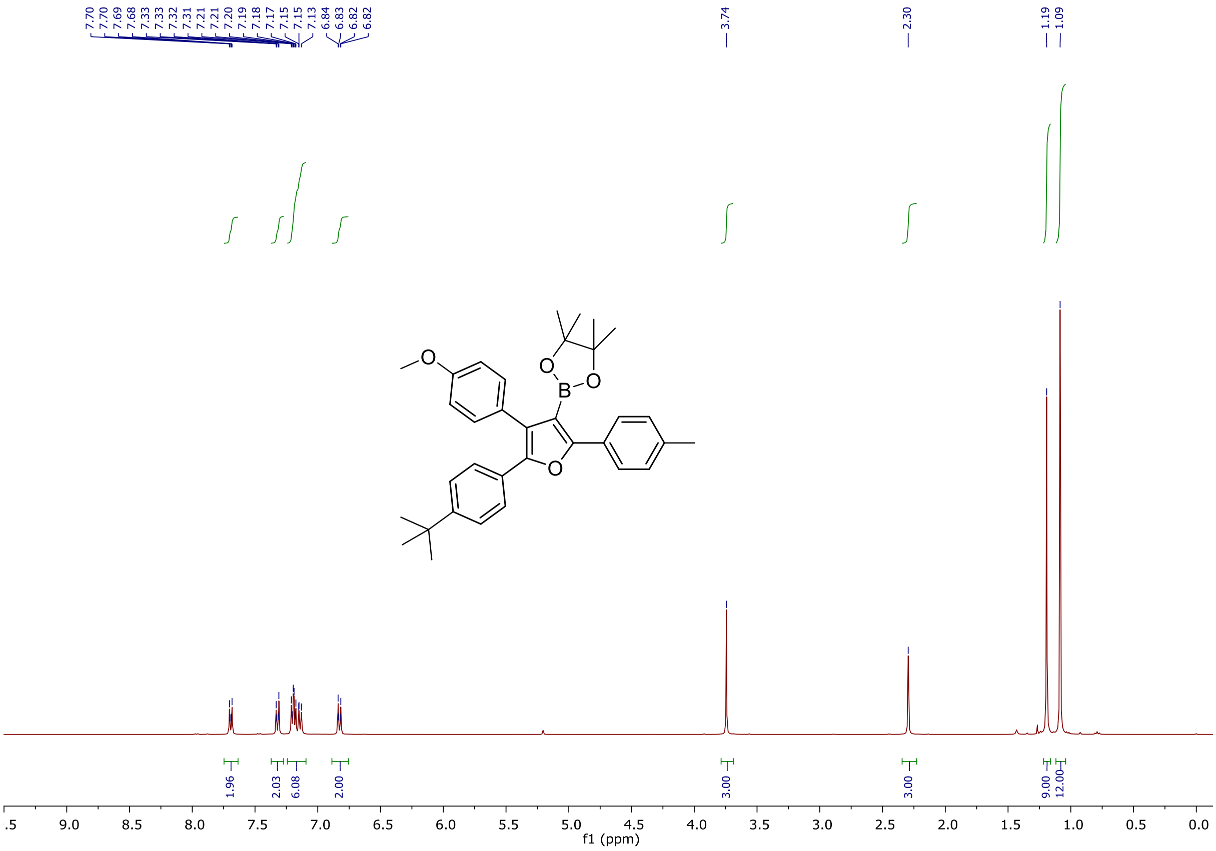

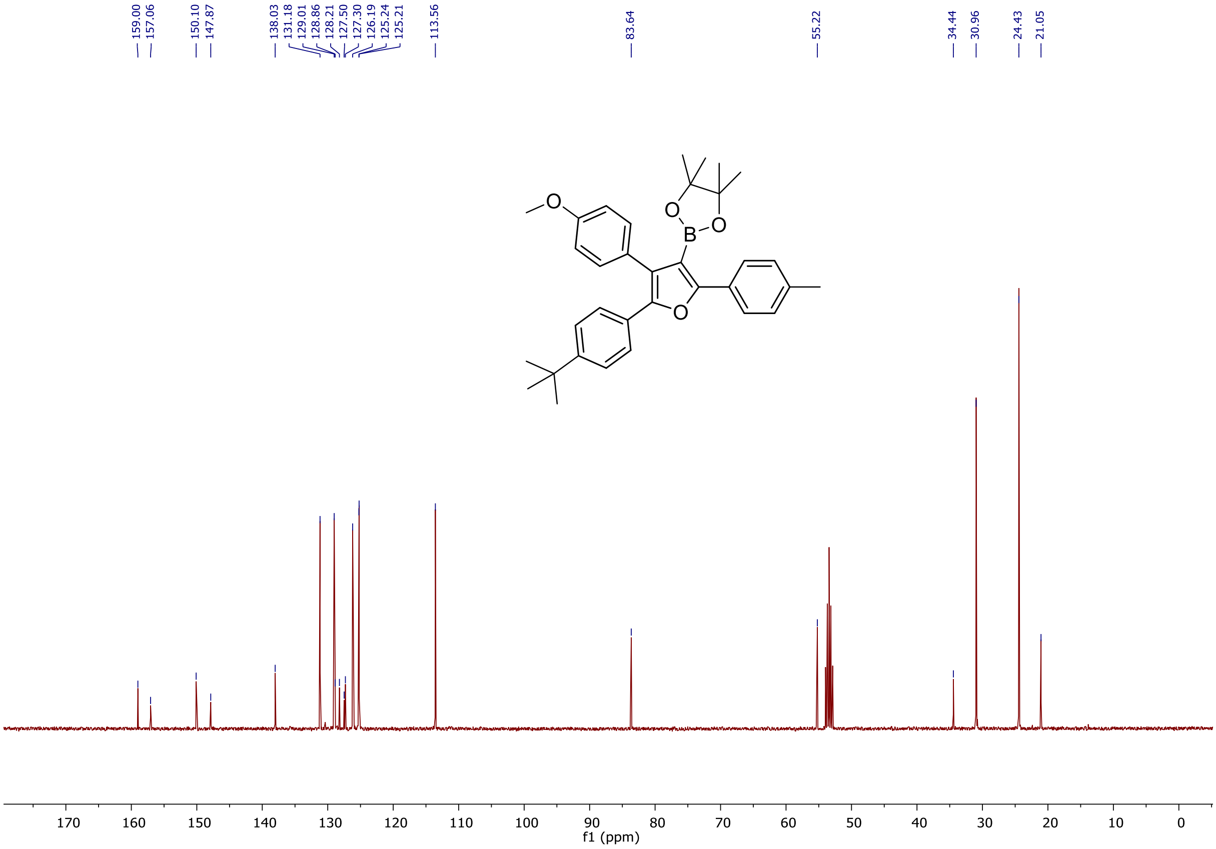

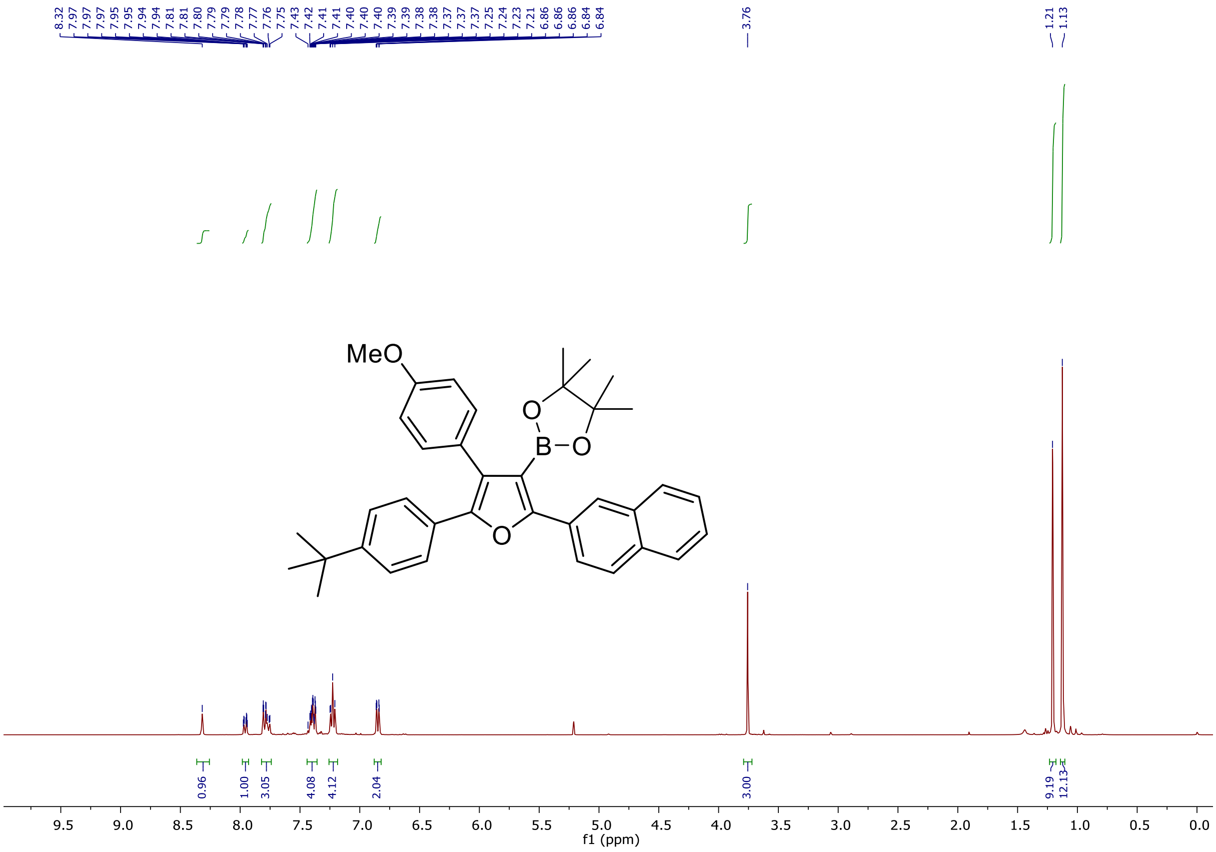
8.32
7.97
7.97
7.95
7.94
7.94
7.81
7.81
7.80
7.79
7.79
7.78
7.77
7.76
7.75
7.43
7.42
7.41
7.41
7.40
7.40
7.40
7.39
7.39
7.38
7.38
7.37
7.37
7.37
7.25
7.24
7.23
7.21
6.86
6.86
6.86
6.84
6.84
3.76
1.21
1.13
MeO
O
B
O
O
0.96
1.00
3.05
4.08
4.12
2.04
3.00
9.19
12.13
9.5 9.0 8.5 8.0 7.5 7.0 6.5 6.0 5.5 5.0 4.5 4.0 3.5 3.0 2.5 2.0 1.5 1.0 0.5 0.0
f1 (ppm)

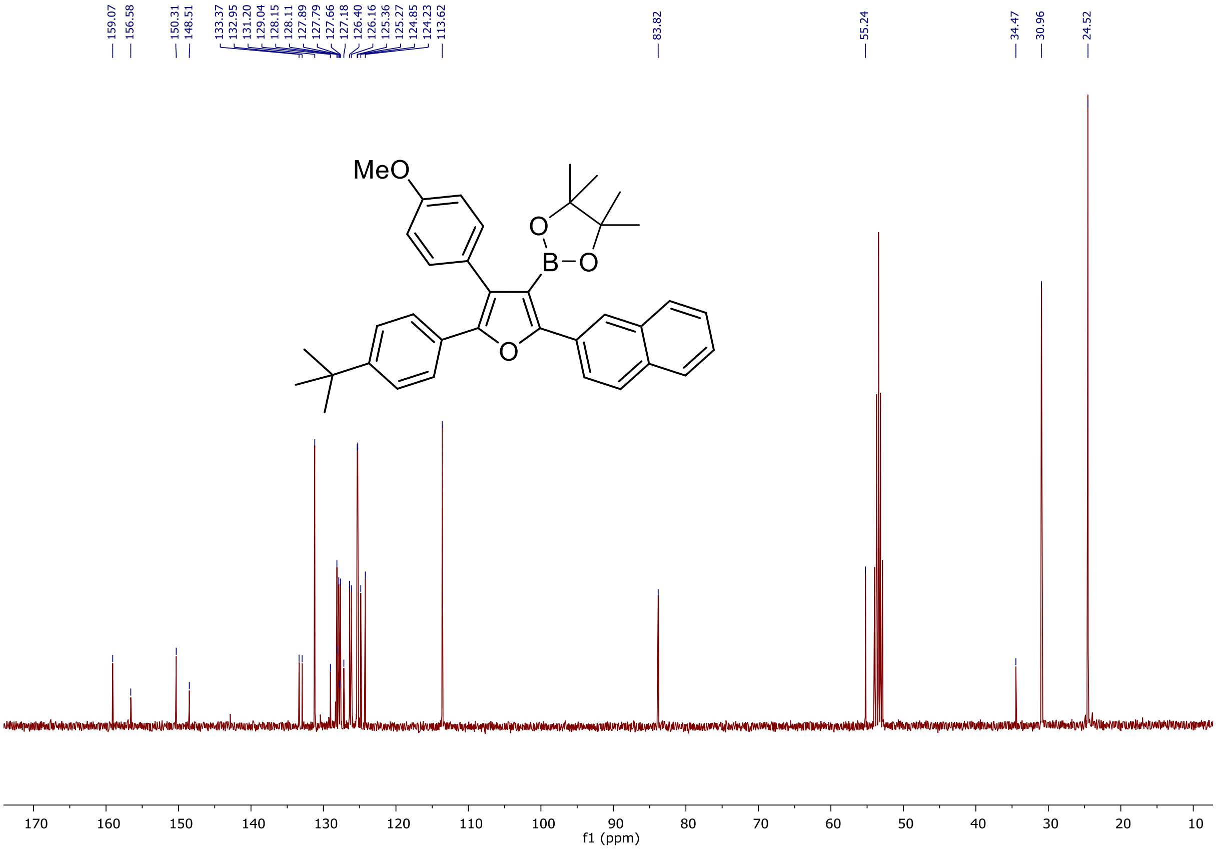
159.07
156.58
150.31
148.51
133.37
132.95
131.20
129.04
128.15
128.11
127.89
127.79
127.66
127.18
126.40
126.16
125.36
125.27
124.85
124.23
113.62
83.82
55.24
34.47
30.96
24.52
MeO
O
B
O
O
170 160 150 140 130 120 110 100 90 80 70 60 50 40 30 20 10
f1 (ppm)

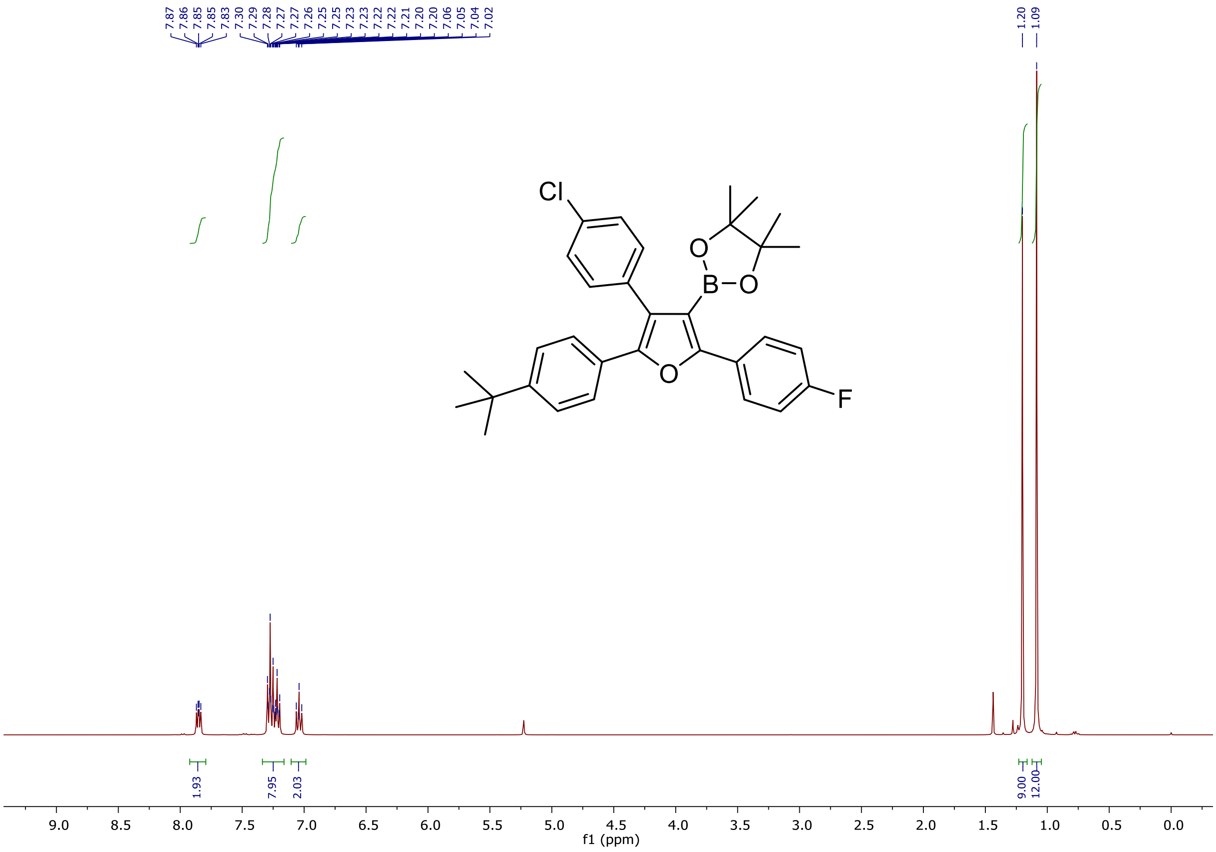
7.87
7.86
7.85
7.85
7.83
7.30
7.29
7.28
7.27
7.27
7.26
7.25
7.25
7.23
7.23
7.22
7.22
7.21
7.20
7.20
7.06
7.05
7.04
7.02
1.20
1.09
Cl
O
B
O
O
F
1.93
7.95
2.03
9.00
12.00
9.0
8.5
8.0
7.5
7.0
6.5
6.0
5.5
5.0
4.5
4.0
3.5
3.0
2.5
2.0
1.5
1.0
0.5
0.0
f1 (ppm)

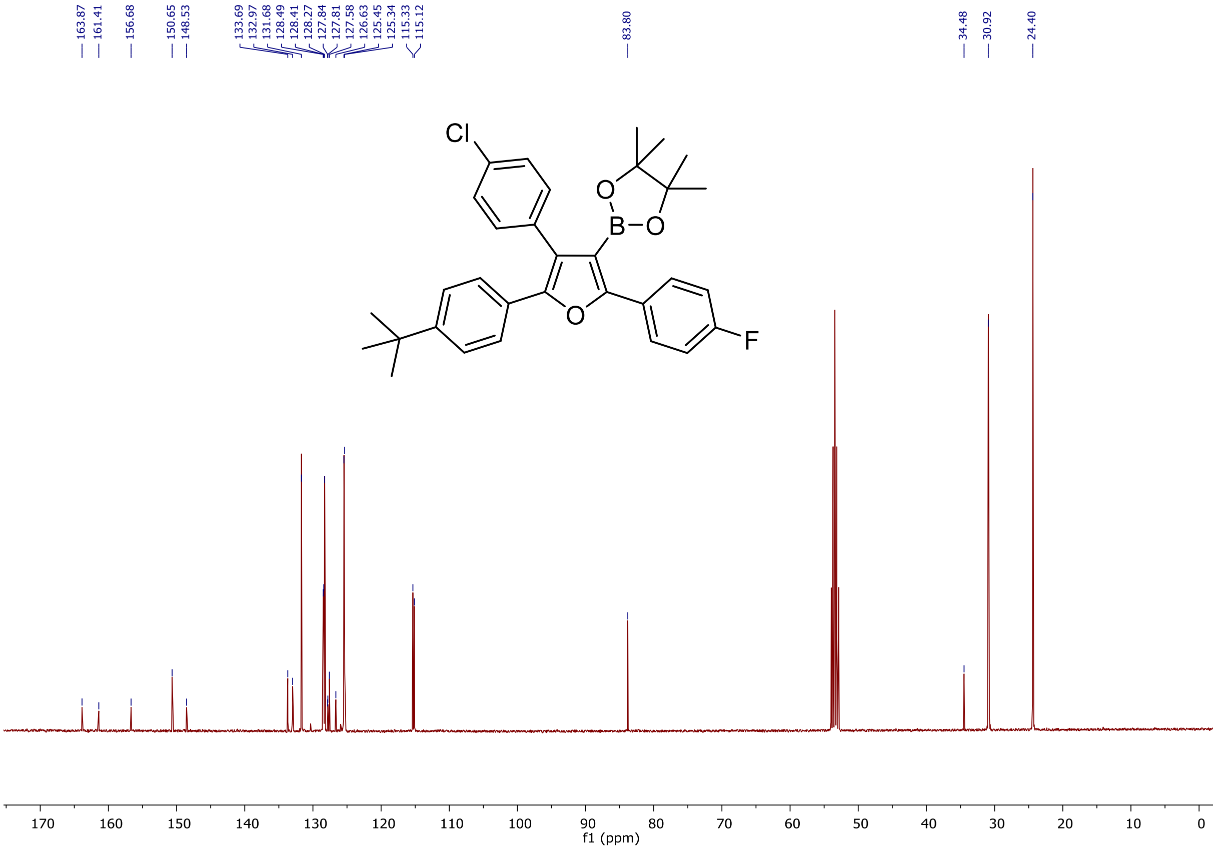
163.87
161.41
156.68
150.65
148.53
133.69
132.97
131.68
128.49
128.41
128.27
127.84
127.81
127.58
126.63
125.45
125.34
115.33
115.12
83.80
34.48
30.92
24.40
Cl
O
B
O
O
F
170
160
150
140
130
120
110
100
90
80
70
60
50
40
30
20
10
0
f1 (ppm)

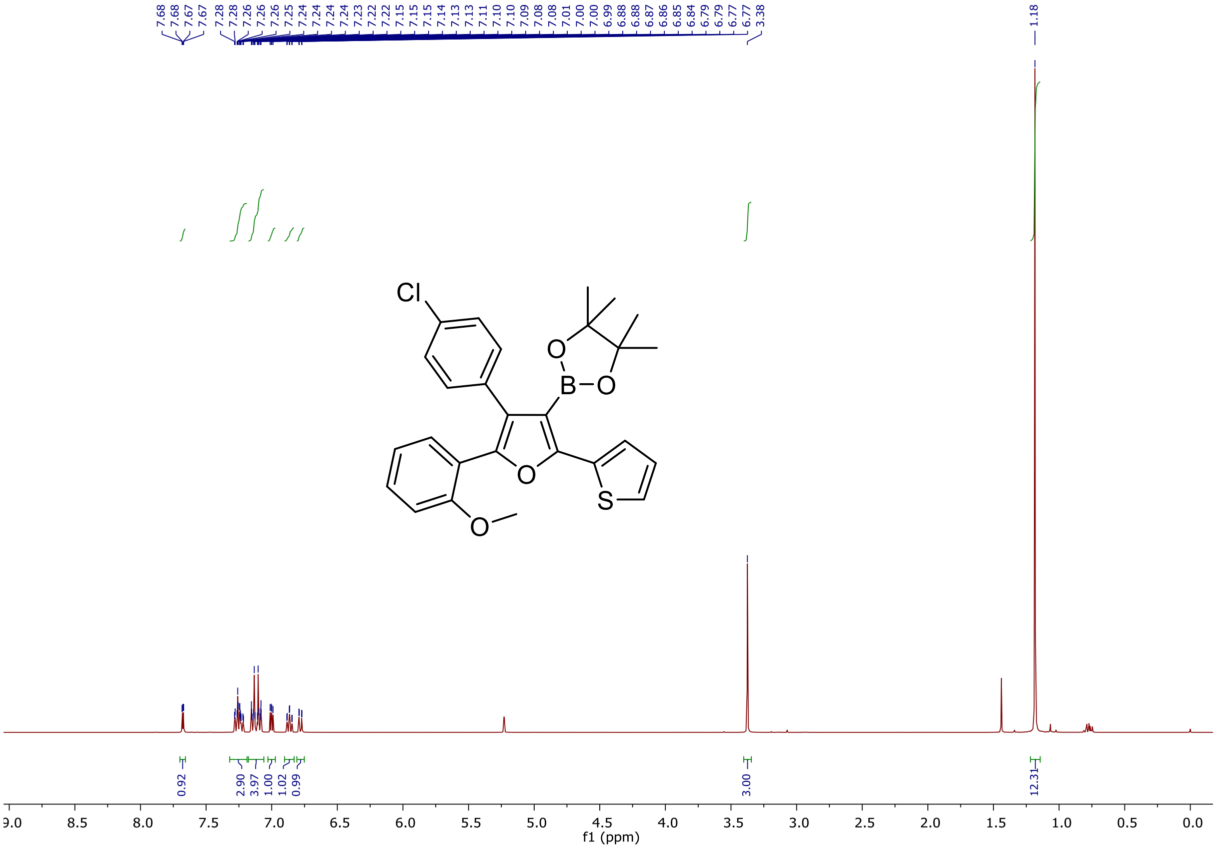

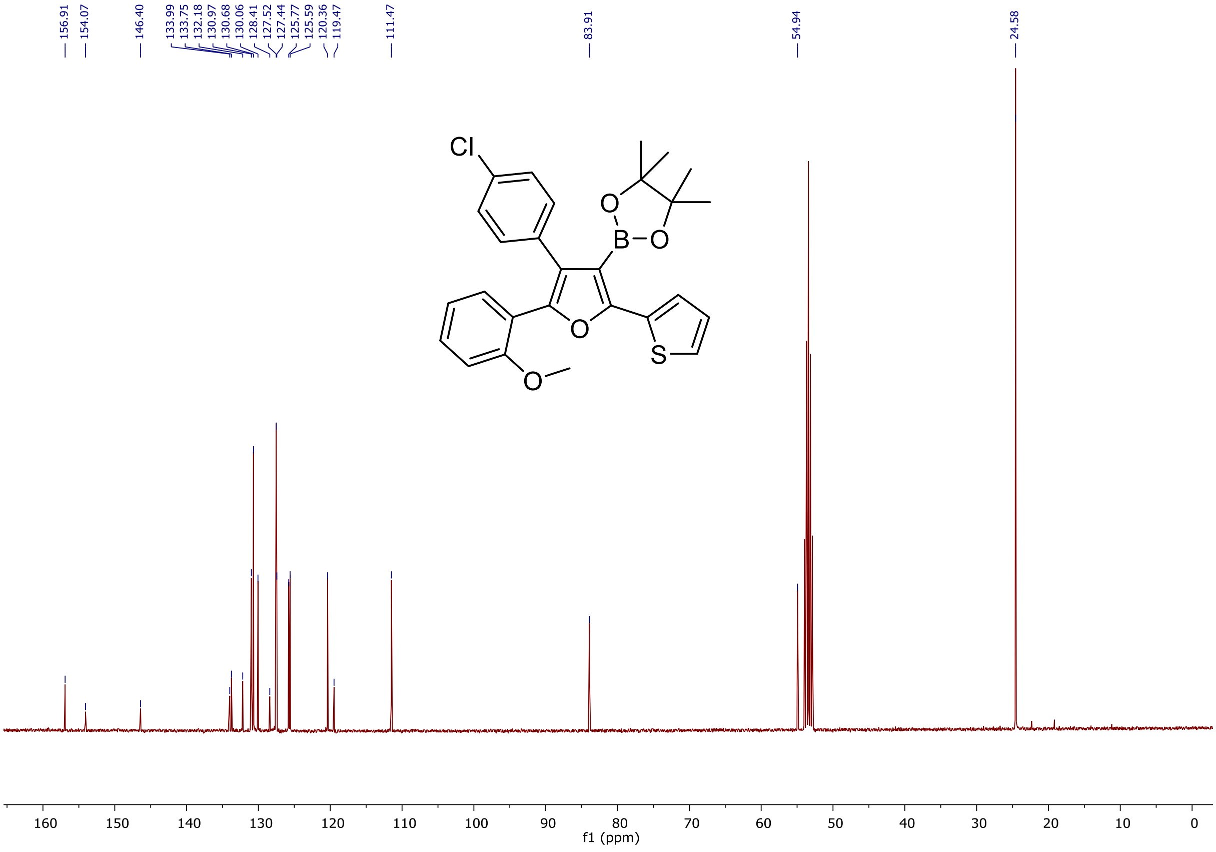

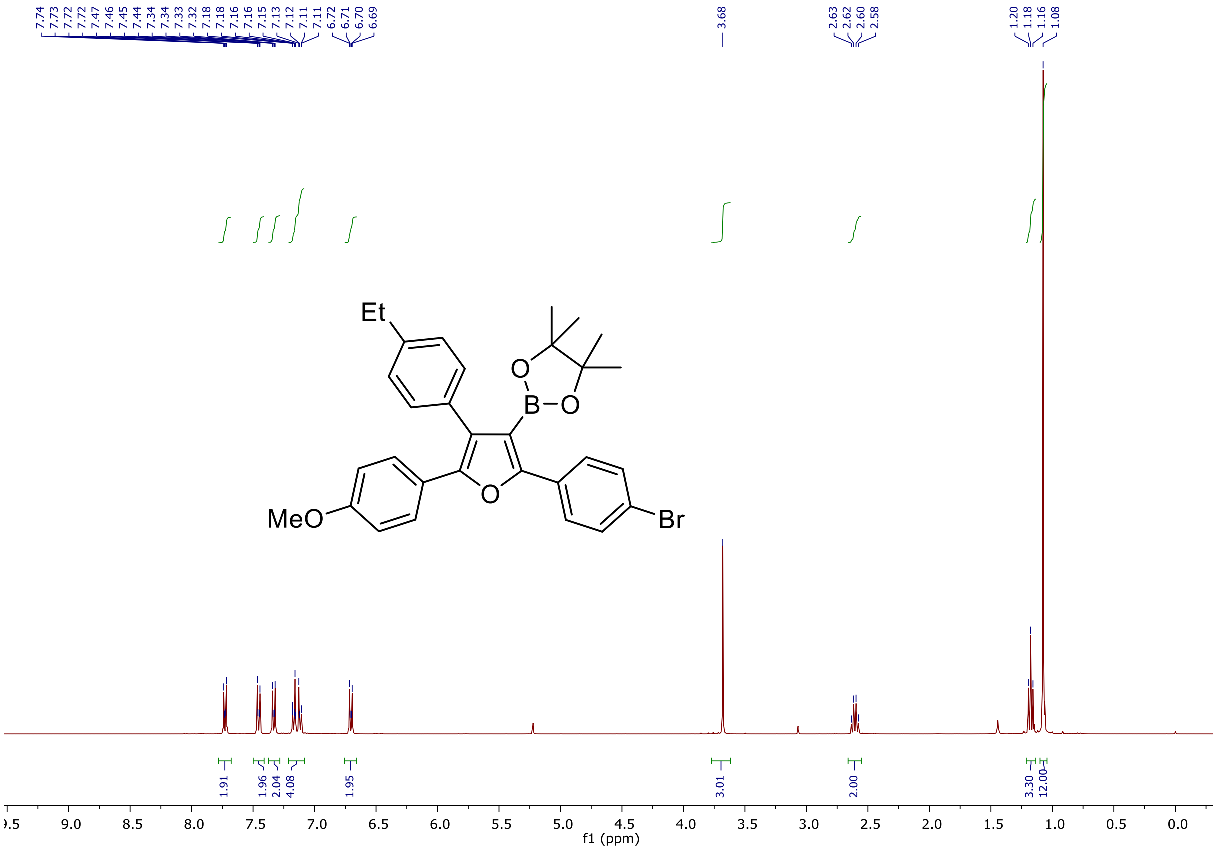
7.74
7.73
7.72
7.72
7.47
7.46
7.45
7.44
7.34
7.34
7.33
7.32
7.18
7.18
7.16
7.16
7.15
7.13
7.12
7.11
7.11
6.72
6.71
6.70
6.69
3.68
2.63
2.62
2.60
2.58
1.20
1.18
1.16
1.08
Et
O
B
O
MeO
O
Br
1.91
1.96
2.04
4.08
1.95
3.01
2.00
3.30
12.00
f1 (ppm)

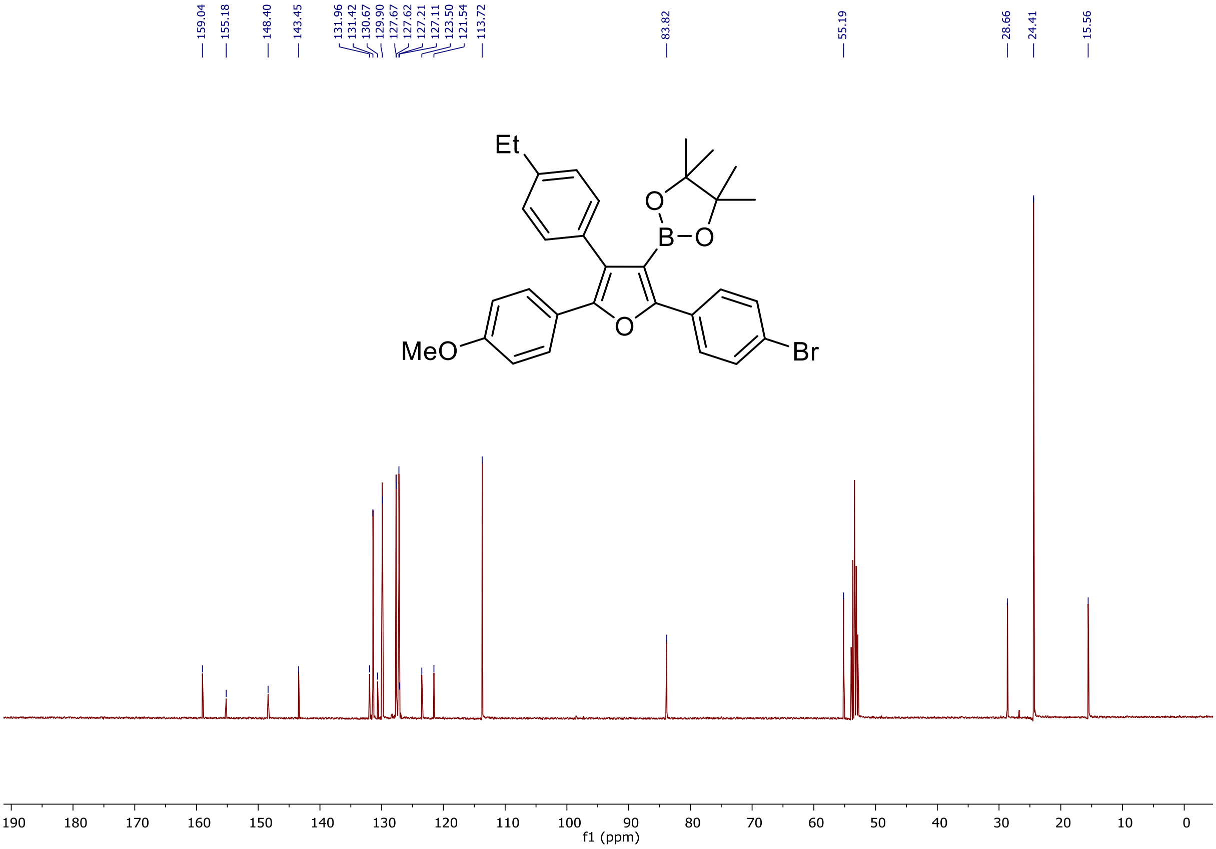
159.04
155.18
148.40
143.45
131.96
131.42
130.67
129.90
127.67
127.62
127.21
127.11
123.50
121.54
113.72
83.82
55.19
28.66
24.41
15.56
Et
O
B
O
MeO
O
Br
f1 (ppm)

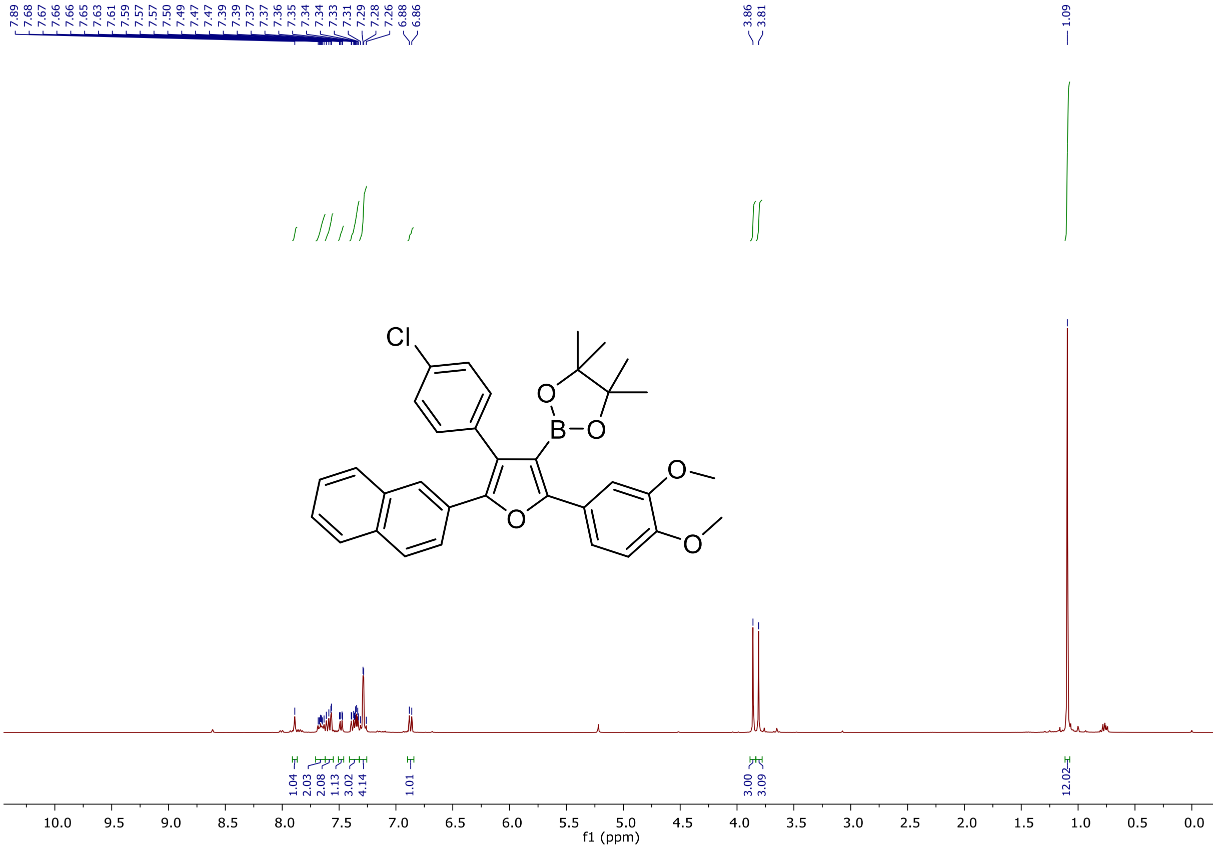
7.89 7.68 7.67 7.66 7.65 7.63 7.61 7.59 7.57 7.50 7.49 7.47 7.47 7.39 7.39 7.37 7.36 7.35 7.34 7.34 7.33 7.31 7.29 7.28 7.26 6.88 6.86
3.86 3.81
1.09
Cl
O
B
O
O
O
O
1.04 2.03 2.08 1.13 3.02 4.14 1.01
3.00 3.09
12.02
10.0 9.5 9.0 8.5 8.0 7.5 7.0 6.5 6.0 5.5 5.0 4.5 4.0 3.5 3.0 2.5 2.0 1.5 1.0 0.5 0.0
f1 (ppm)

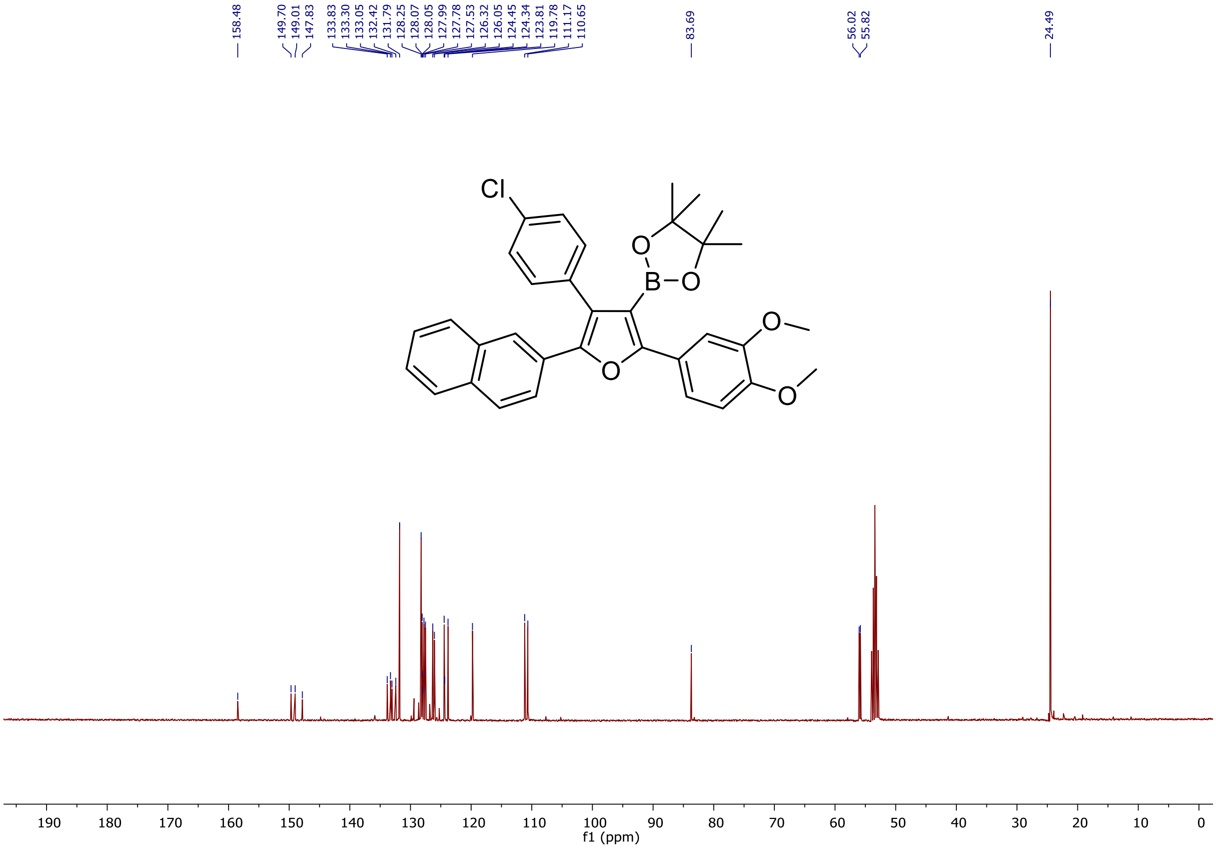
158.48
149.70 149.01 147.83
133.83 133.30 133.05 132.42 131.79 128.25 128.07 128.05 127.99 127.78 127.53 126.32 126.05 124.45 124.34 123.81 119.78 111.17 110.65
83.69
56.02 55.82
24.49
Cl
O
B
O
O
O
O
190 180 170 160 150 140 130 120 110 100 90 80 70 60 50 40 30 20 10 0
f1 (ppm)

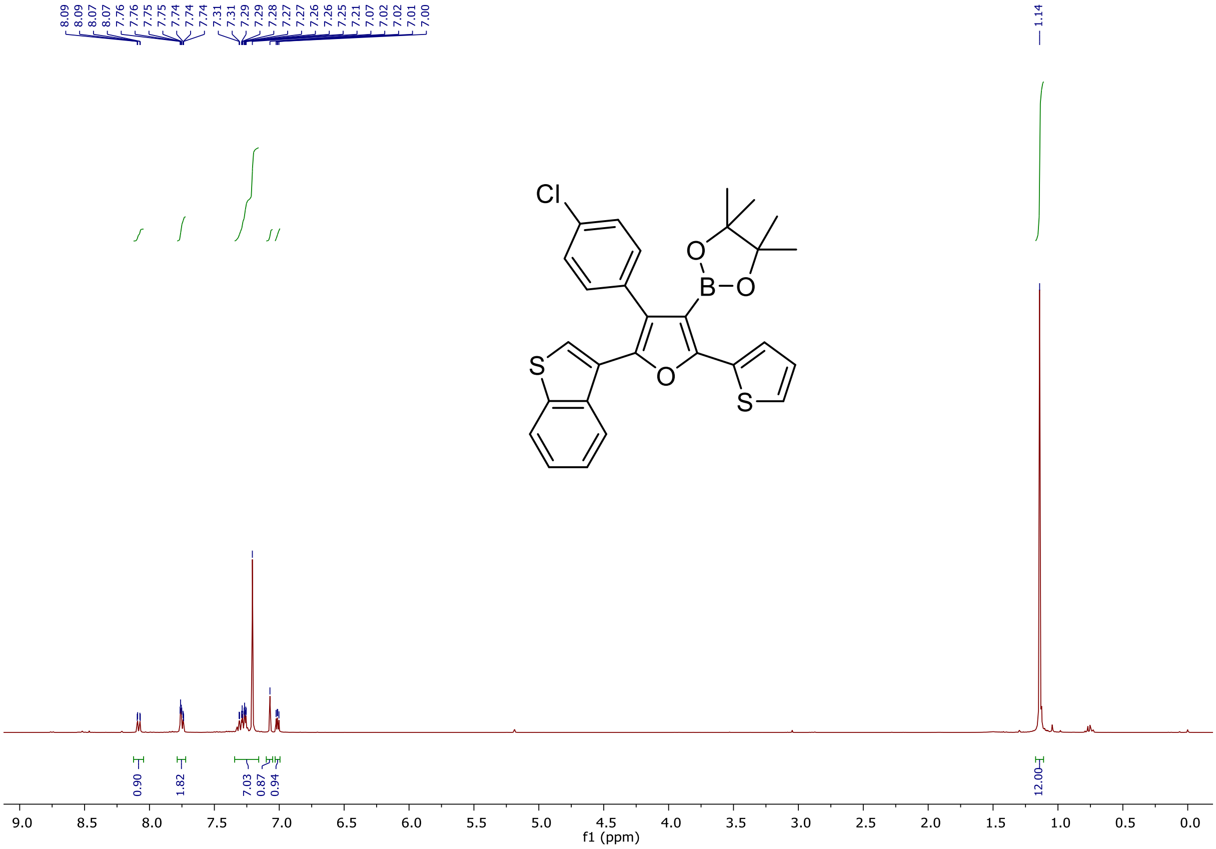

8.09
8.09
8.07
7.76
7.76
7.75
7.75
7.74
7.74
7.74
7.31
7.29
7.29
7.28
7.27
7.27
7.26
7.26
7.25
7.21
7.07
7.02
7.02
7.01
7.00
1.14
0.90
1.82
7.03
0.87
0.94
12.00
9.0
8.5
8.0
7.5
7.0
6.5
6.0
5.5
5.0
4.5
4.0
3.5
3.0
2.5
2.0
1.5
1.0
0.5
0.0
f1 (ppm)
Cl
O
B
O
S
O
S

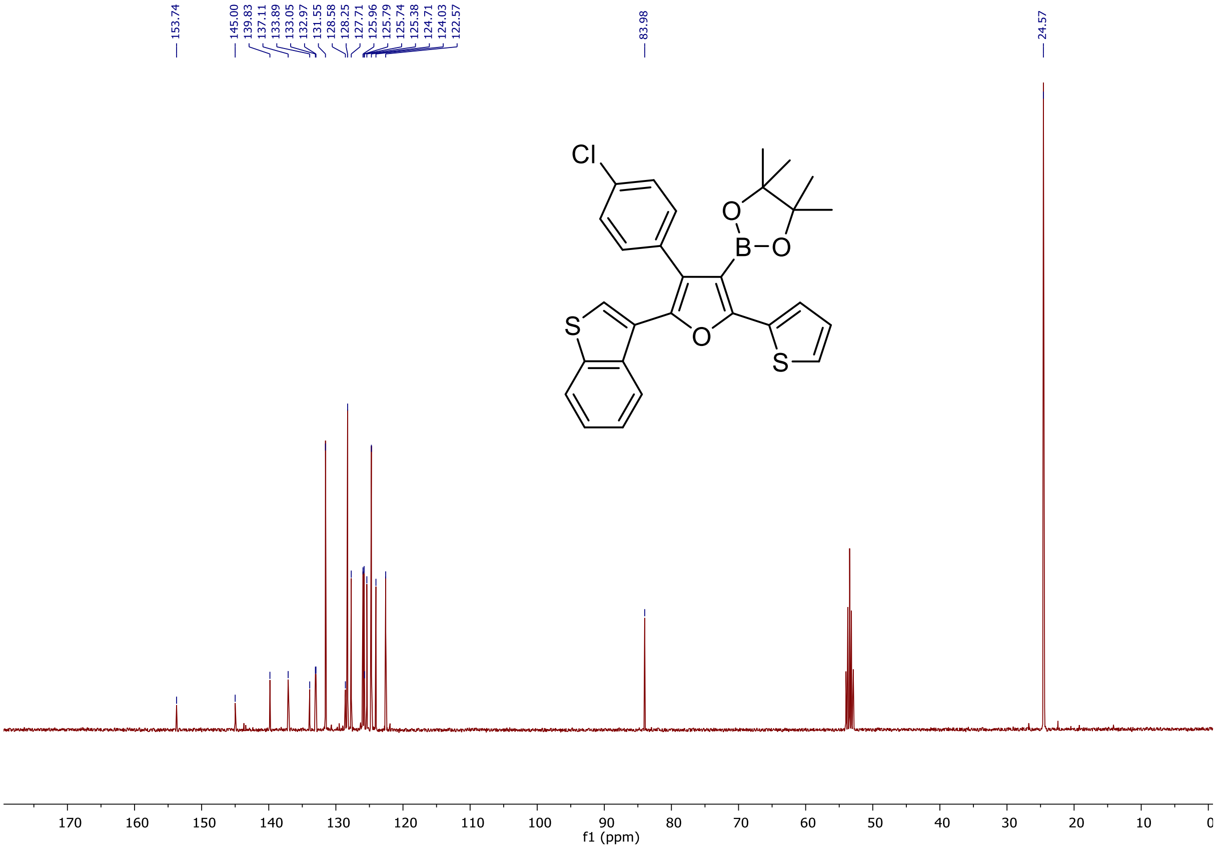

153.74
145.00
139.83
137.11
133.89
133.05
132.97
131.55
128.58
128.25
127.71
125.96
125.79
125.74
125.38
124.71
124.03
122.57
83.98
24.57
170
160
150
140
130
120
110
100
90
80
70
60
50
40
30
20
10
0
f1 (ppm)
Cl
O
B
O
S
O
S

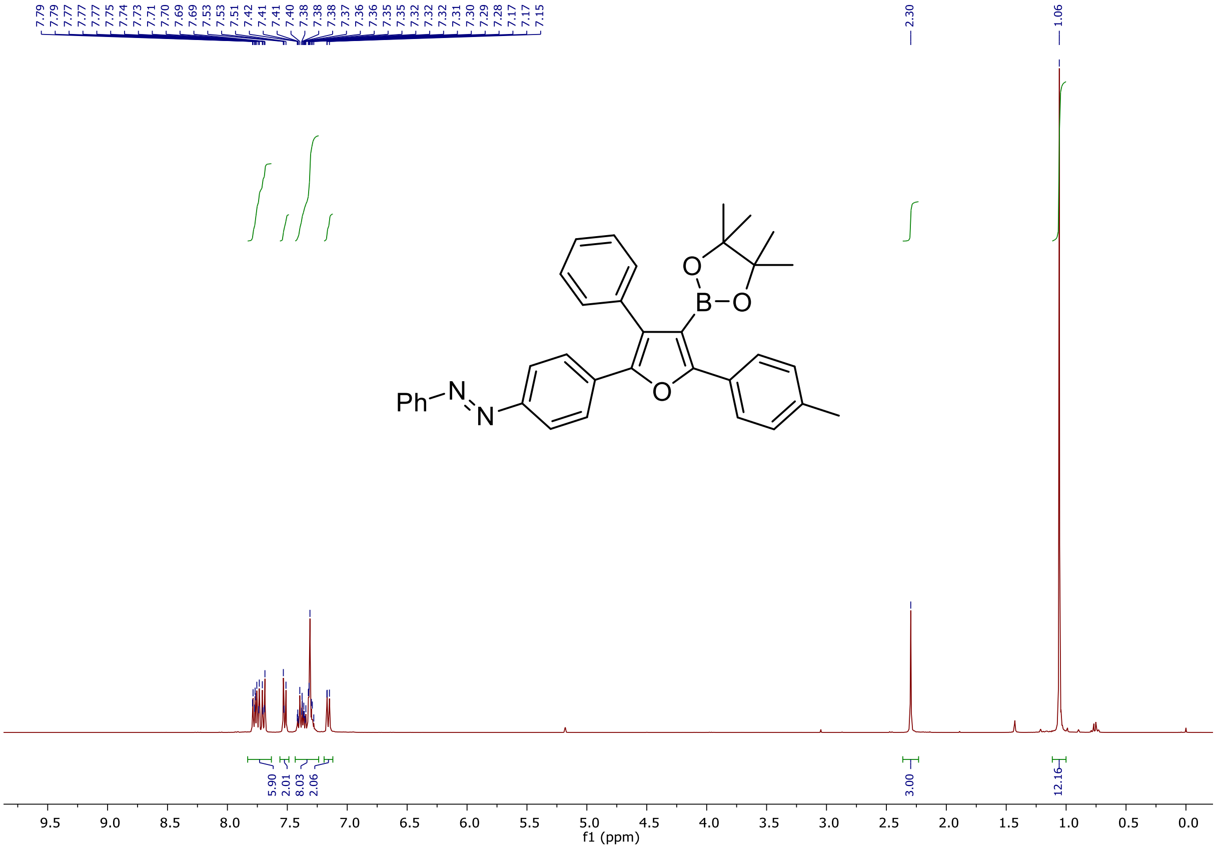
7.79
7.79
7.77
7.77
7.77
7.75
7.74
7.73
7.71
7.70
7.69
7.69
7.53
7.53
7.51
7.42
7.41
7.41
7.40
7.38
7.38
7.38
7.37
7.36
7.36
7.35
7.35
7.32
7.32
7.32
7.31
7.30
7.29
7.28
7.17
7.17
7.15
2.30
1.06
Ph
N
N
O
B
O
O
5.90
2.01
8.03
2.06
3.00
12.16
9.5 9.0 8.5 8.0 7.5 7.0 6.5 6.0 5.5 5.0 4.5 4.0 3.5 3.0 2.5 2.0 1.5 1.0 0.5 0.0
f1 (ppm)

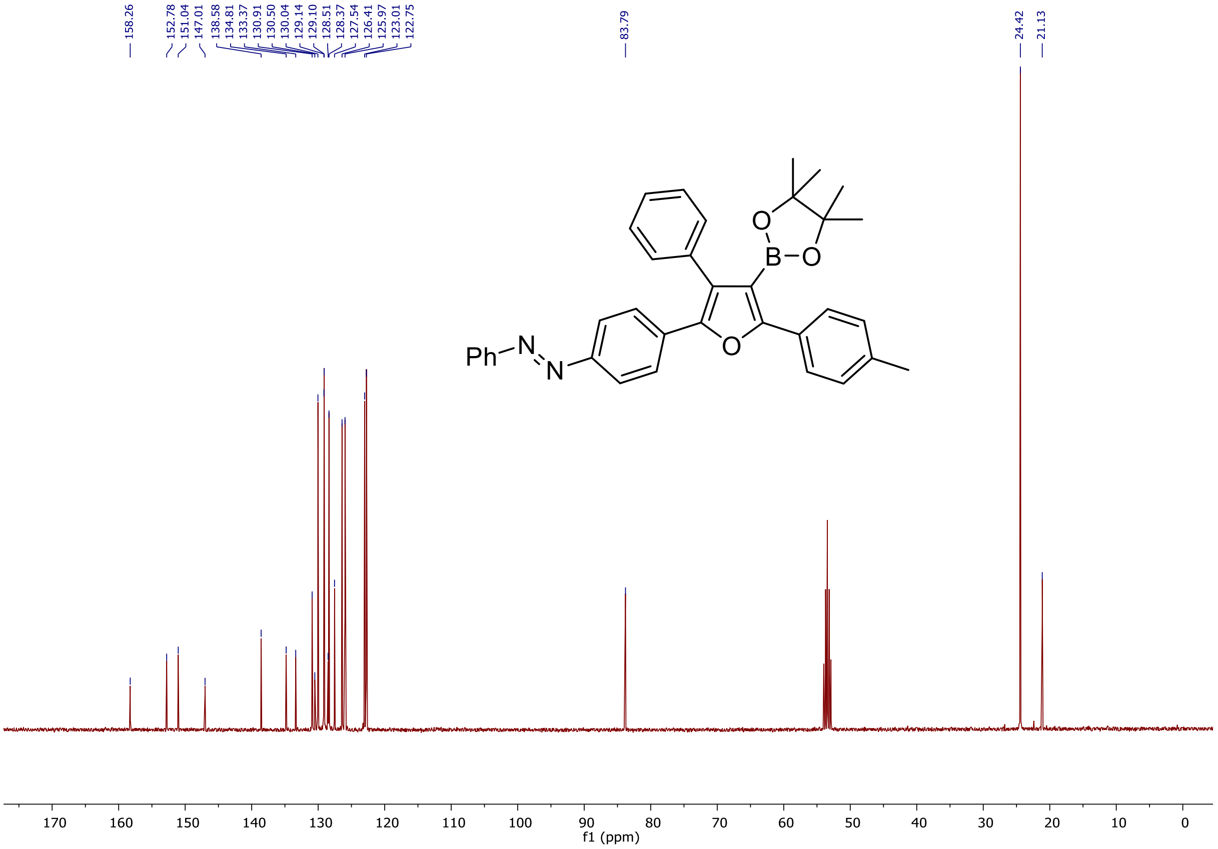
158.26
152.78
151.04
147.01
138.58
134.81
133.37
130.91
130.50
130.04
129.14
129.10
128.51
128.37
127.54
126.41
125.97
123.01
122.75
83.79
24.42
21.13
Ph
N
N
O
B
O
O
170 160 150 140 130 120 110 100 90 80 70 60 50 40 30 20 10 0
f1 (ppm)

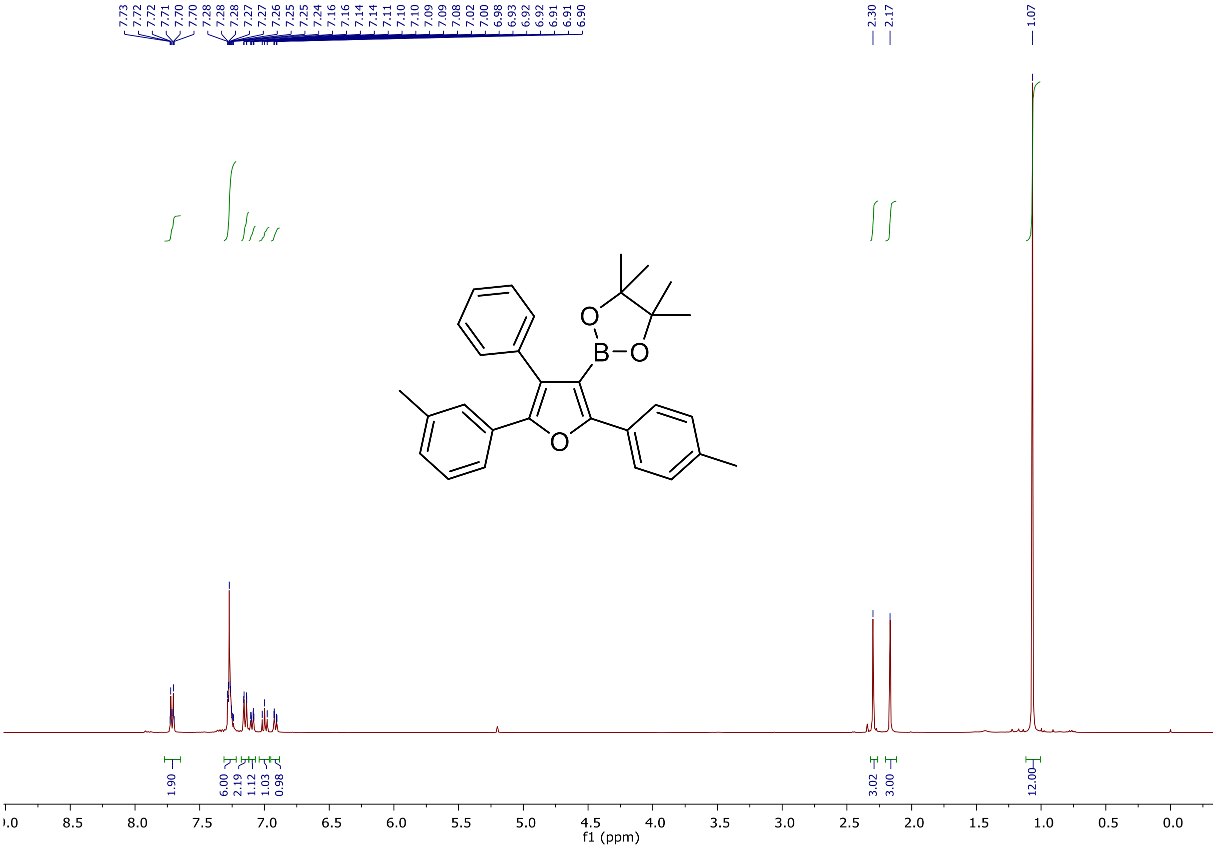
7.73 7.72 7.72 7.71 7.70 7.70 7.28 7.28 7.28 7.27 7.26 7.25 7.25 7.24 7.16 7.16 7.14 7.14 7.11 7.10 7.10 7.09 7.09 7.08 7.02 7.00 6.98 6.93 6.92 6.92 6.91 6.91 6.90
2.30
2.17
1.07
O
B
O
O
1.90
6.00 2.19 1.12 1.03 0.98
3.02
3.00
12.00
8.5 8.0 7.5 7.0 6.5 6.0 5.5 5.0 4.5 4.0 3.5 3.0 2.5 2.0 1.5 1.0 0.5 0.0
f1 (ppm)

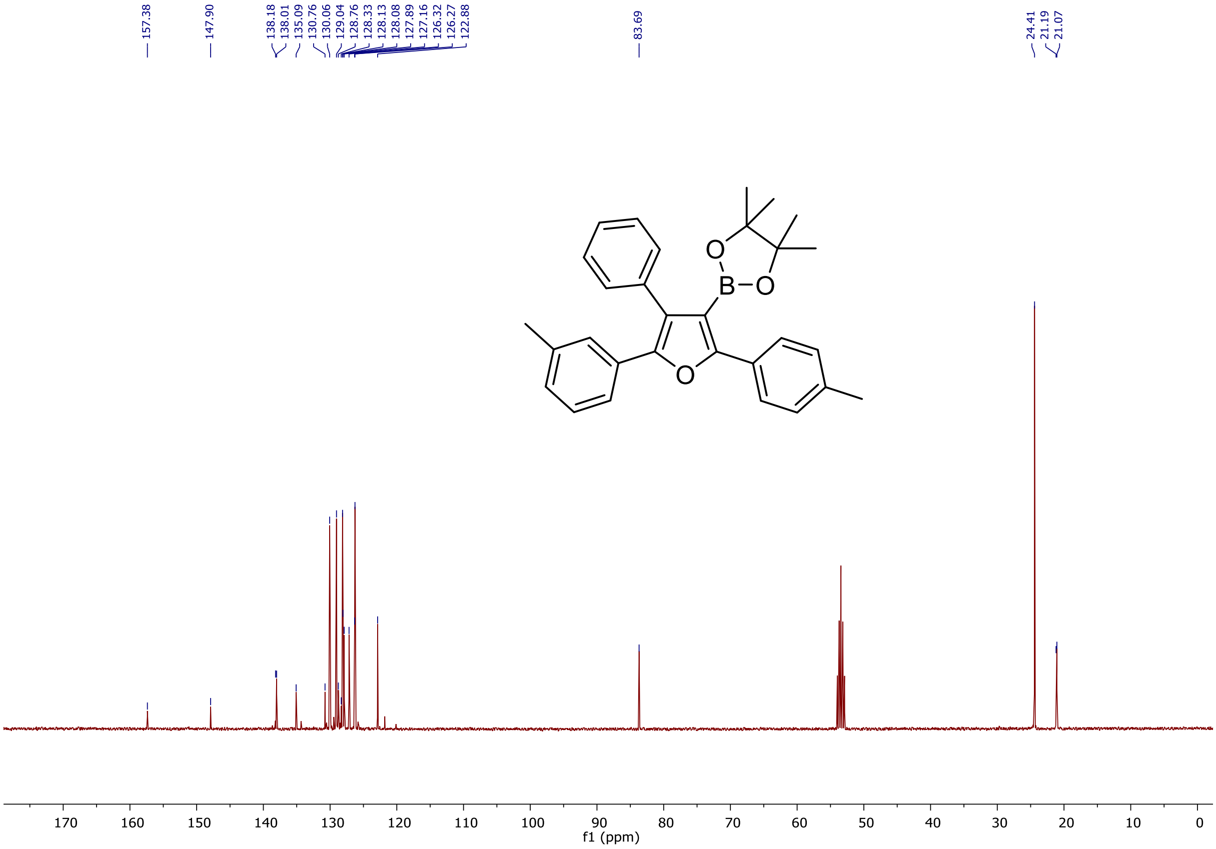
157.38
147.90
138.18 138.01 135.09 130.76 130.06 129.04 128.76 128.33 128.13 128.08 127.89 127.16 126.32 126.27 122.88
83.69
24.41 21.19 21.07
O
B
O
O
170 160 150 140 130 120 110 100 90 80 70 60 50 40 30 20 10 0
f1 (ppm)

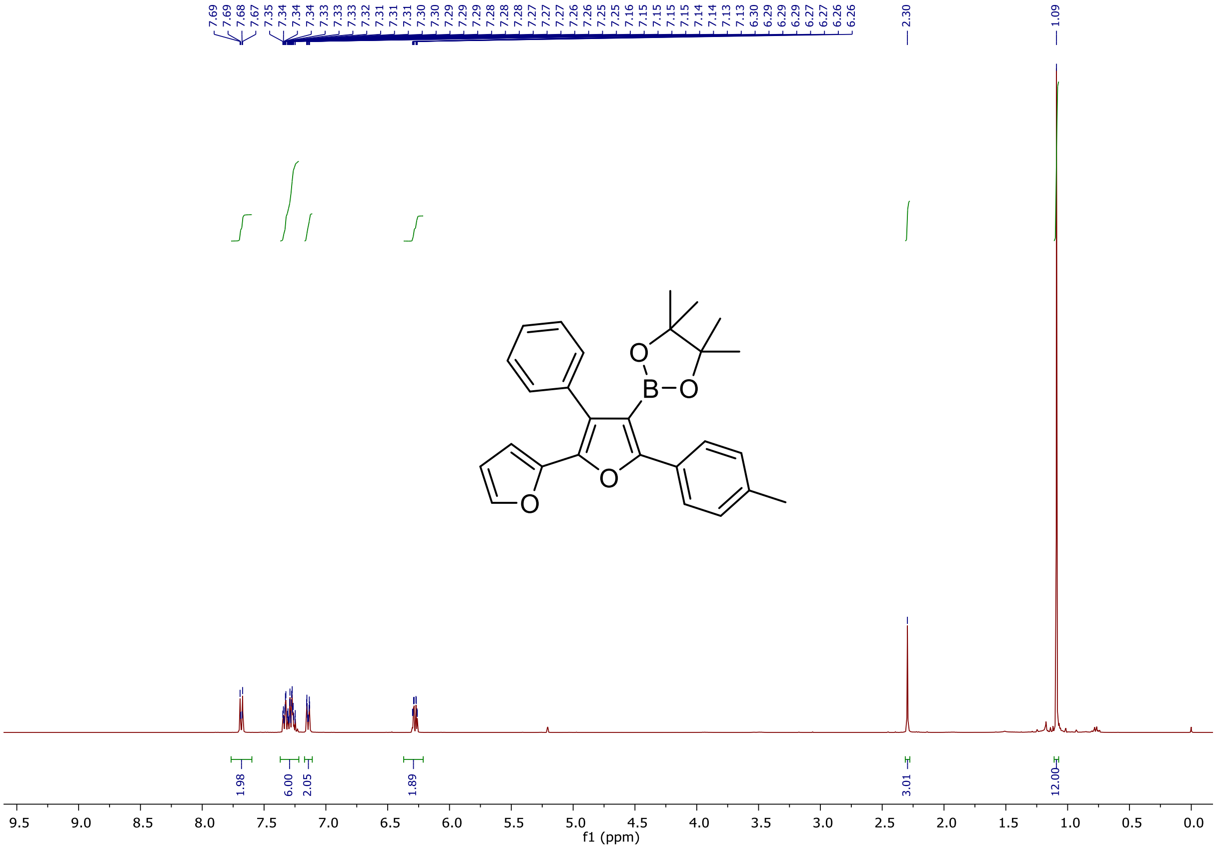
7.69
7.69
7.68
7.67
7.35
7.34
7.34
7.34
7.33
7.33
7.33
7.32
7.31
7.31
7.31
7.30
7.30
7.29
7.29
7.29
7.28
7.28
7.28
7.27
7.27
7.27
7.26
7.25
7.25
7.16
7.15
7.15
7.15
7.15
7.14
7.14
7.13
7.13
6.30
6.29
6.29
6.29
6.27
6.27
6.26
6.26
2.30
1.09
O
B
O
O
O
1.98
6.00
2.05
1.89
3.01
12.00
9.5
9.0
8.5
8.0
7.5
7.0
6.5
6.0
5.5
5.0
4.5
4.0
3.5
3.0
2.5
2.0
1.5
1.0
0.5
0.0
f1 (ppm)

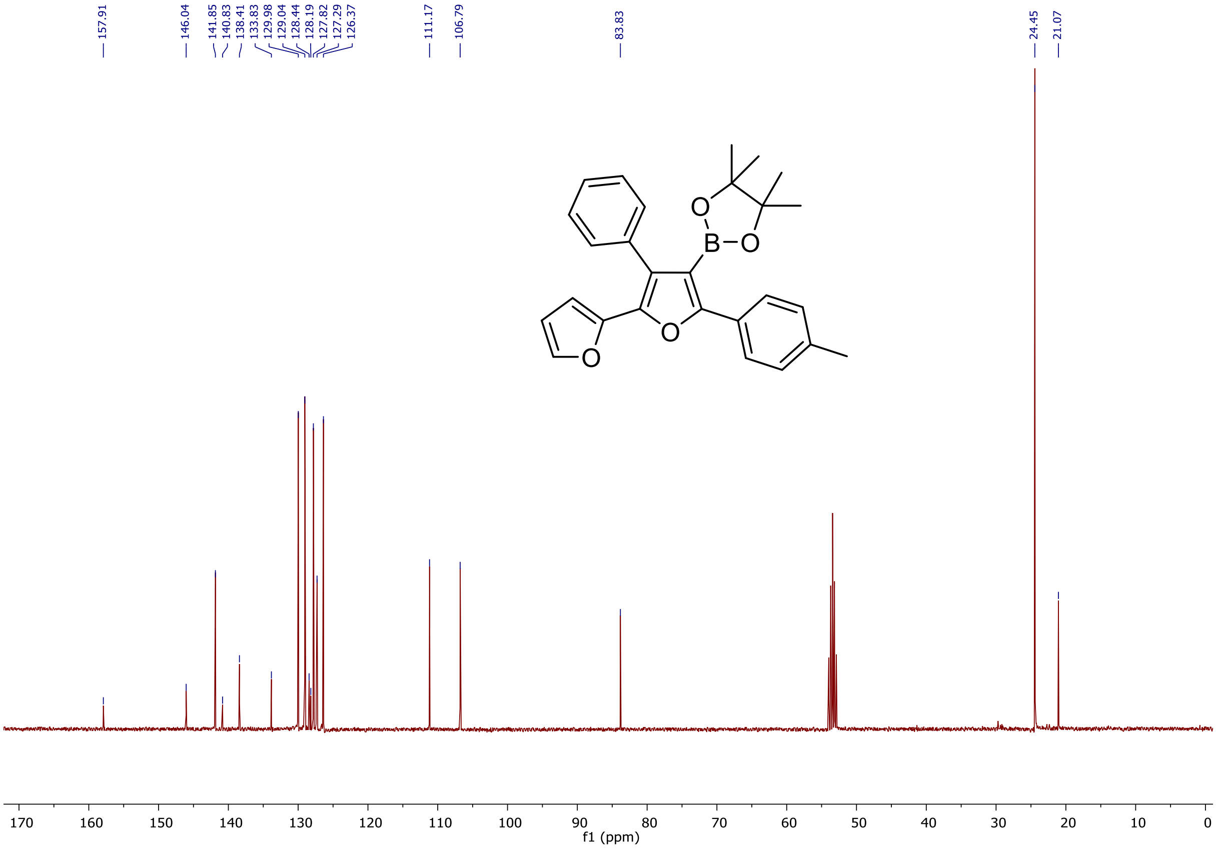
157.91
146.04
141.85
140.83
138.41
133.83
129.98
129.04
128.44
128.19
127.82
127.29
126.37
111.17
106.79
83.83
24.45
21.07
O
B
O
O
O
170
160
150
140
130
120
110
100
90
80
70
60
50
40
30
20
10
0
f1 (ppm)

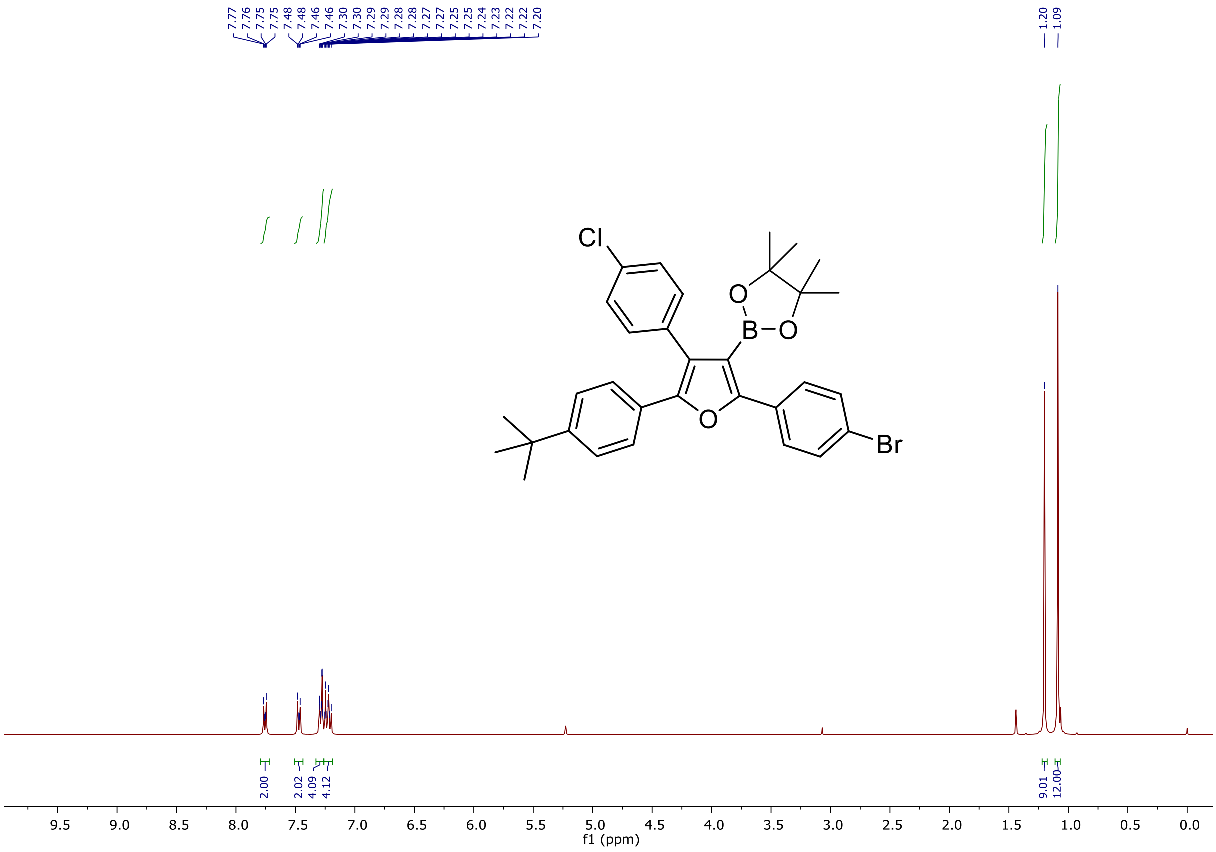
7.77 7.76 7.75 7.75 7.48 7.48 7.46 7.46 7.30 7.30 7.29 7.29 7.28 7.28 7.27 7.27 7.25 7.25 7.24 7.23 7.22 7.22 7.20
1.20 1.09
Cl
O
B
O
O
Br
2.00 2.02 4.09 4.12
9.01 12.00
9.5 9.0 8.5 8.0 7.5 7.0 6.5 6.0 5.5 5.0 4.5 4.0 3.5 3.0 2.5 2.0 1.5 1.0 0.5 0.0
f1 (ppm)

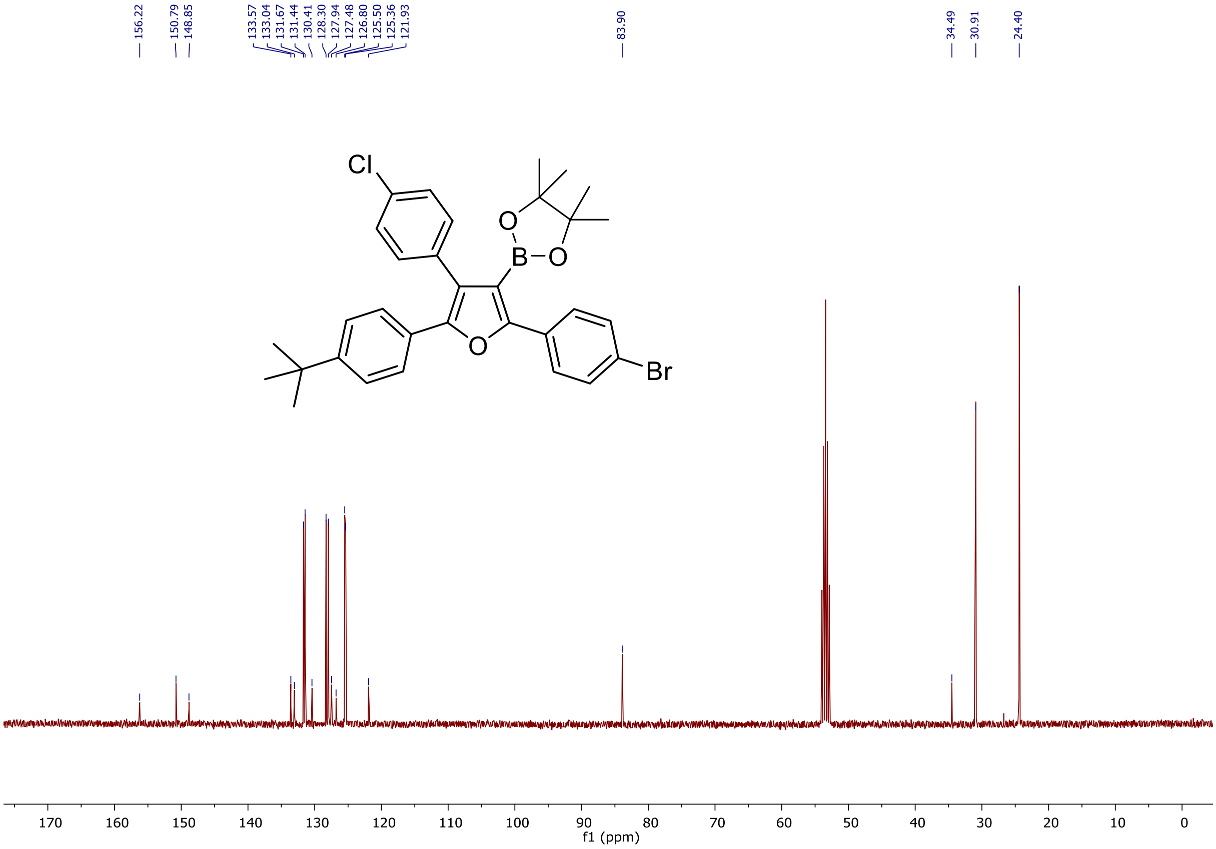
156.22 150.79 148.85
133.57 133.04 131.67 131.44 130.41 128.30 127.94 127.48 126.80 125.50 125.36 121.93
83.90
34.49 30.91 24.40
Cl
O
B
O
O
Br
170 160 150 140 130 120 110 100 90 80 70 60 50 40 30 20 10 0
f1 (ppm)

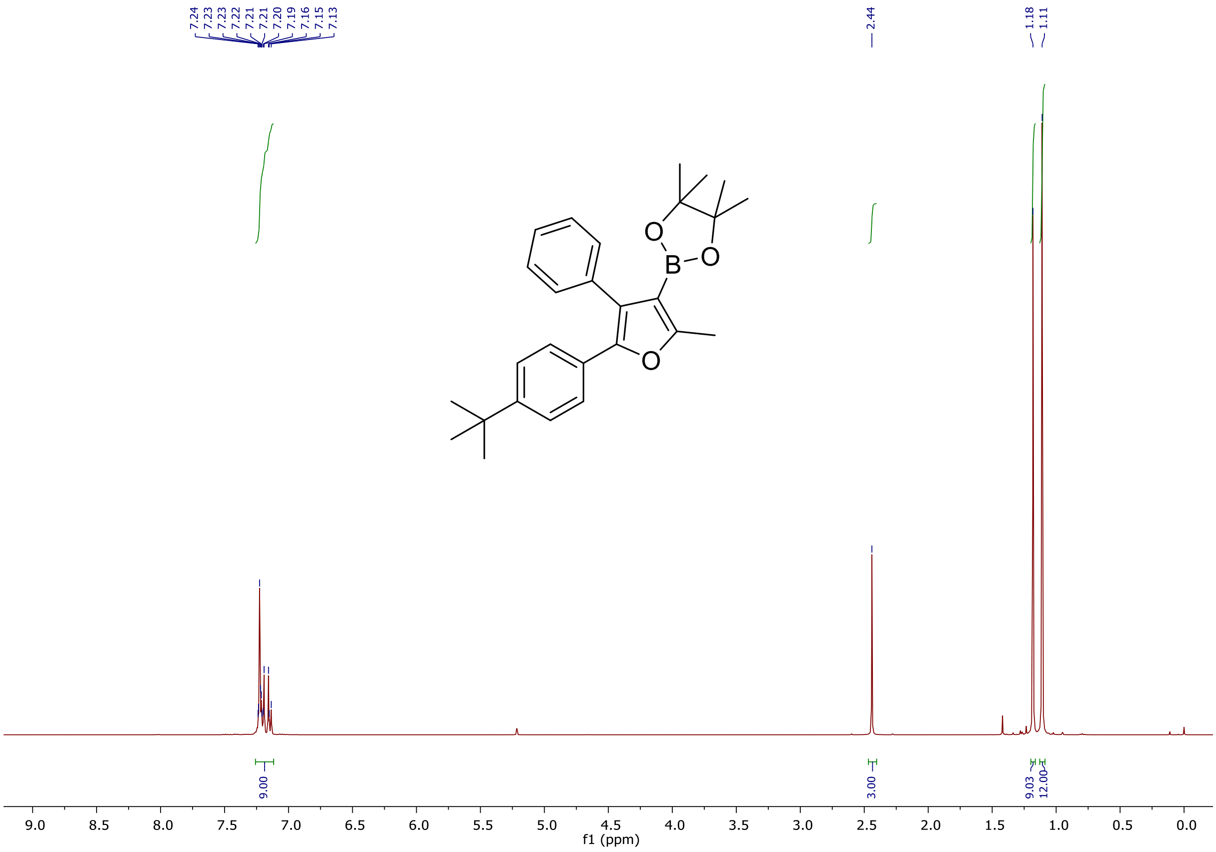
7.24 7.23 7.23 7.22 7.21 7.21 7.20 7.19 7.16 7.15 7.13
2.44
1.18 1.11
9.00
3.00
9.03 12.00
f1 (ppm)

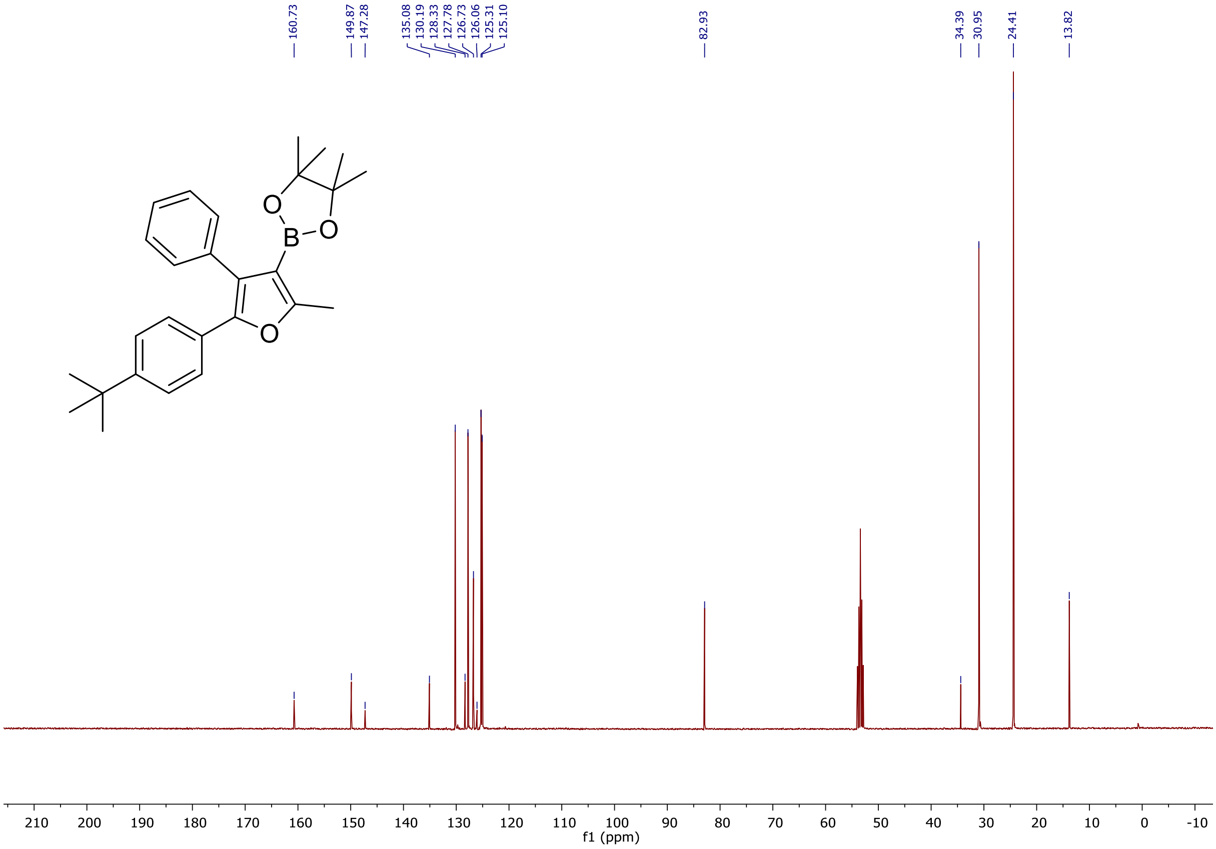
160.73
149.87 147.28
135.08 130.19 128.33 127.78 126.73 126.06 125.31 125.10
82.93
34.39 30.95 24.41 13.82
f1 (ppm)

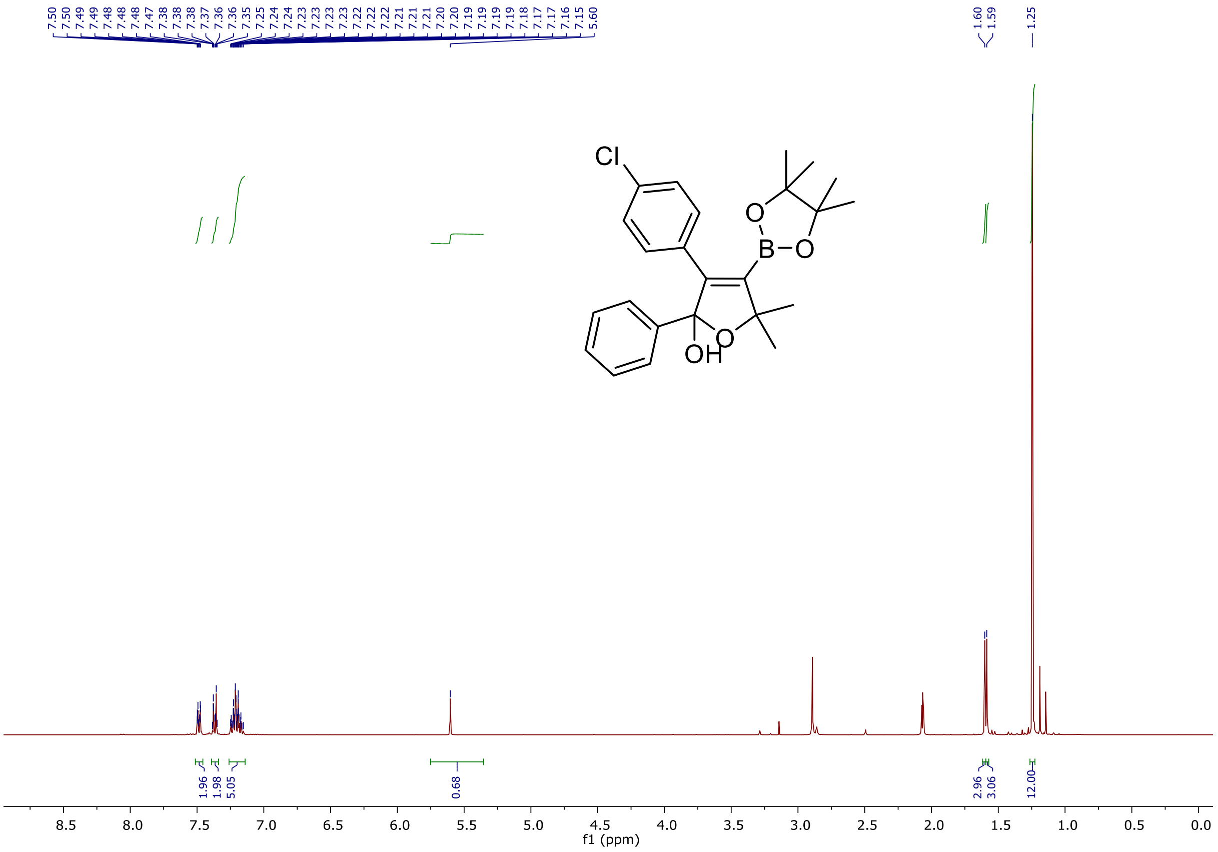

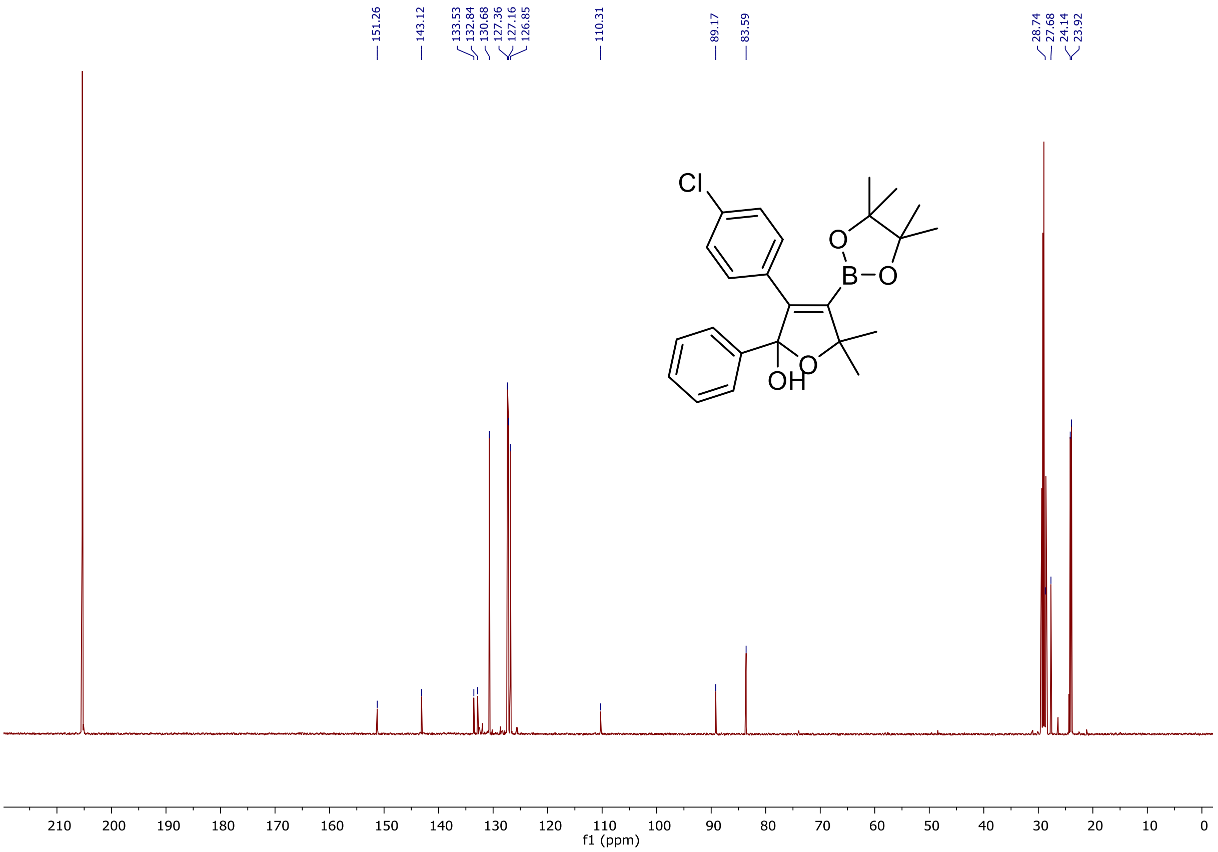

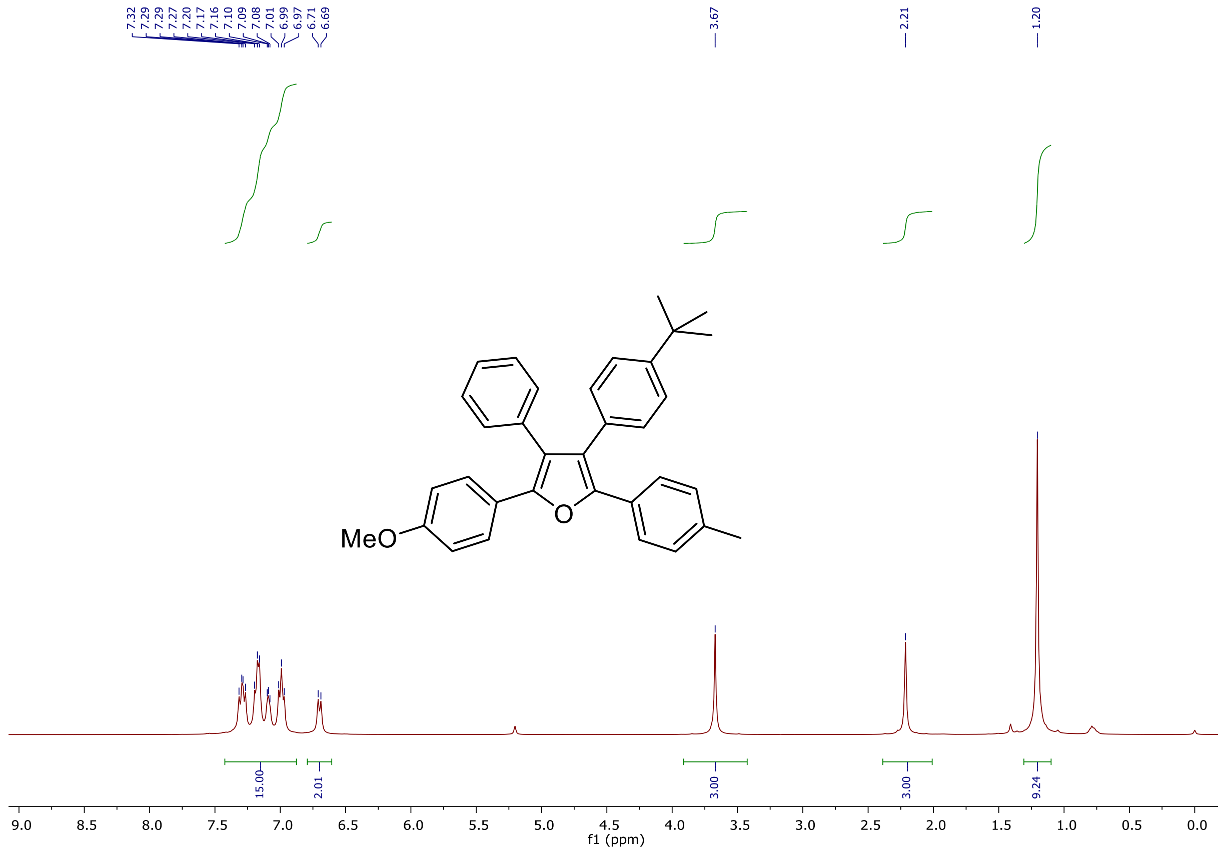
7.32
7.29
7.29
7.27
7.20
7.17
7.16
7.10
7.09
7.08
7.01
6.99
6.97
6.71
6.69
3.67
2.21
1.20
MeO
O
15.00
2.01
3.00
3.00
9.24
f1 (ppm)

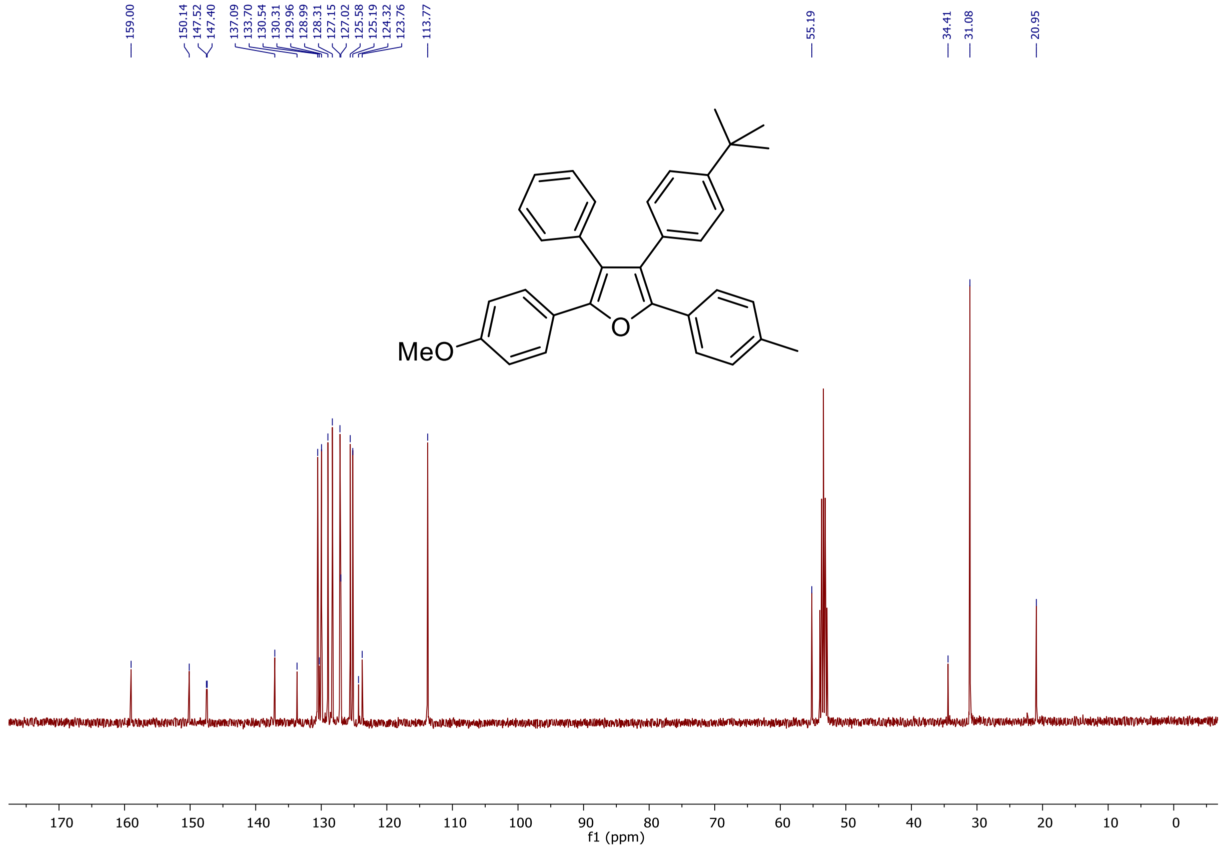
159.00
150.14
147.52
147.40
137.09
133.70
130.54
130.31
129.96
128.99
128.31
127.15
127.02
125.58
125.19
124.32
123.76
113.77
55.19
34.41
31.08
20.95
MeO
O
f1 (ppm)

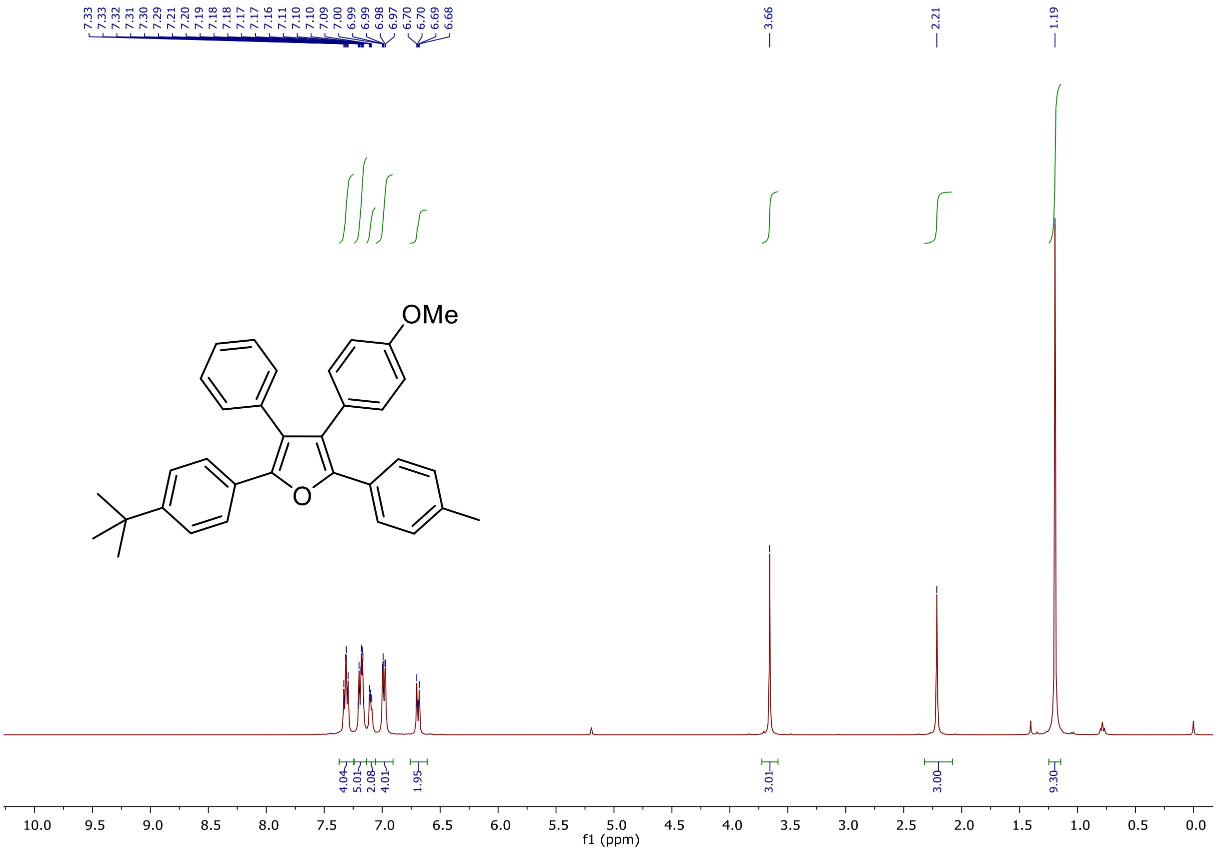
7.33 7.33 7.32 7.31 7.30 7.29 7.21 7.20 7.19 7.18 7.18 7.17 7.17 7.16 7.11 7.10 7.10 7.09 7.00 6.99 6.99 6.98 6.97 6.70 6.70 6.69 6.68
3.66
2.21
1.19
OMe
O
4.04
5.01
2.08
4.01
1.95
3.01
3.00
9.30
10.0 9.5 9.0 8.5 8.0 7.5 7.0 6.5 6.0 5.5 5.0 4.5 4.0 3.5 3.0 2.5 2.0 1.5 1.0 0.5 0.0
f1 (ppm)

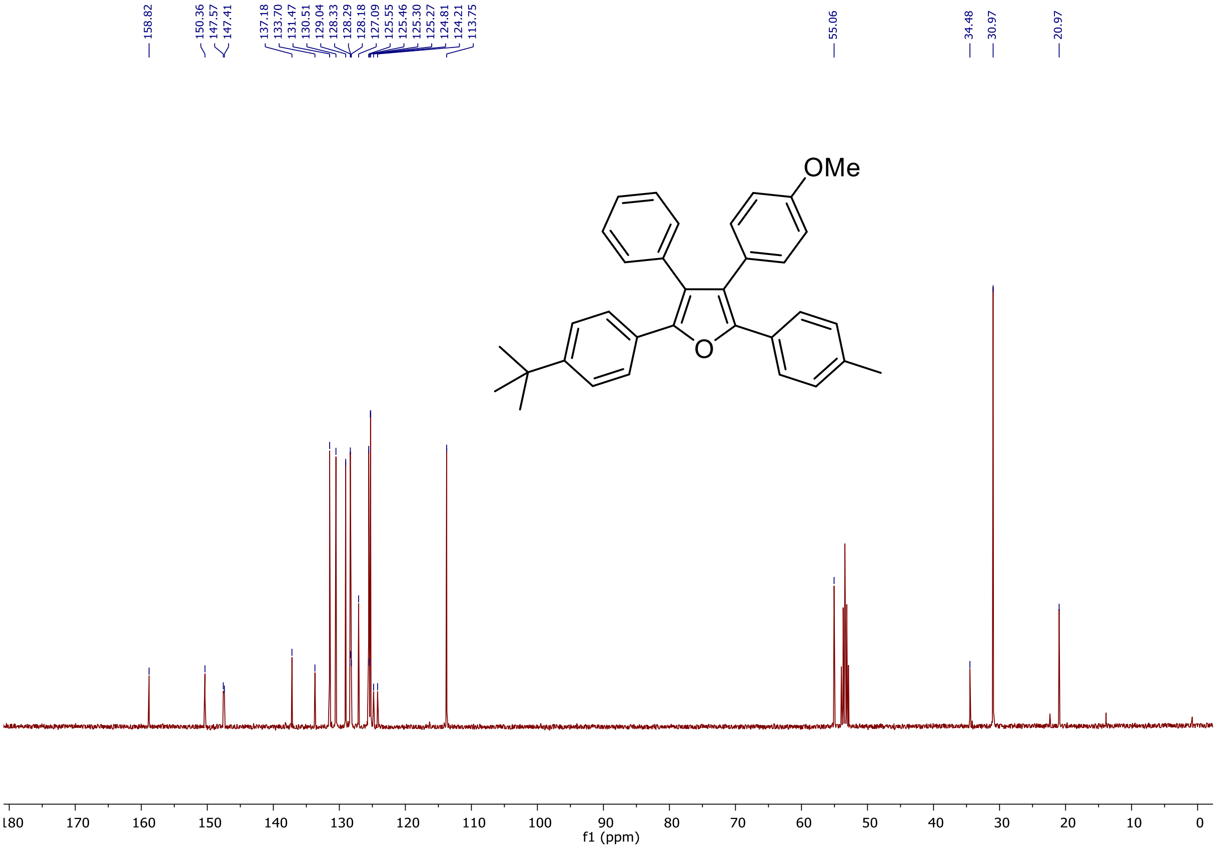
158.82
150.36 147.57 147.41
137.18 133.70 131.47 130.51 129.04 128.33 128.29 128.18 127.09 125.55 125.46 125.30 125.27 124.81 124.21 113.75
55.06
34.48 30.97
20.97
OMe
O
180 170 160 150 140 130 120 110 100 90 80 70 60 50 40 30 20 10 0
f1 (ppm)

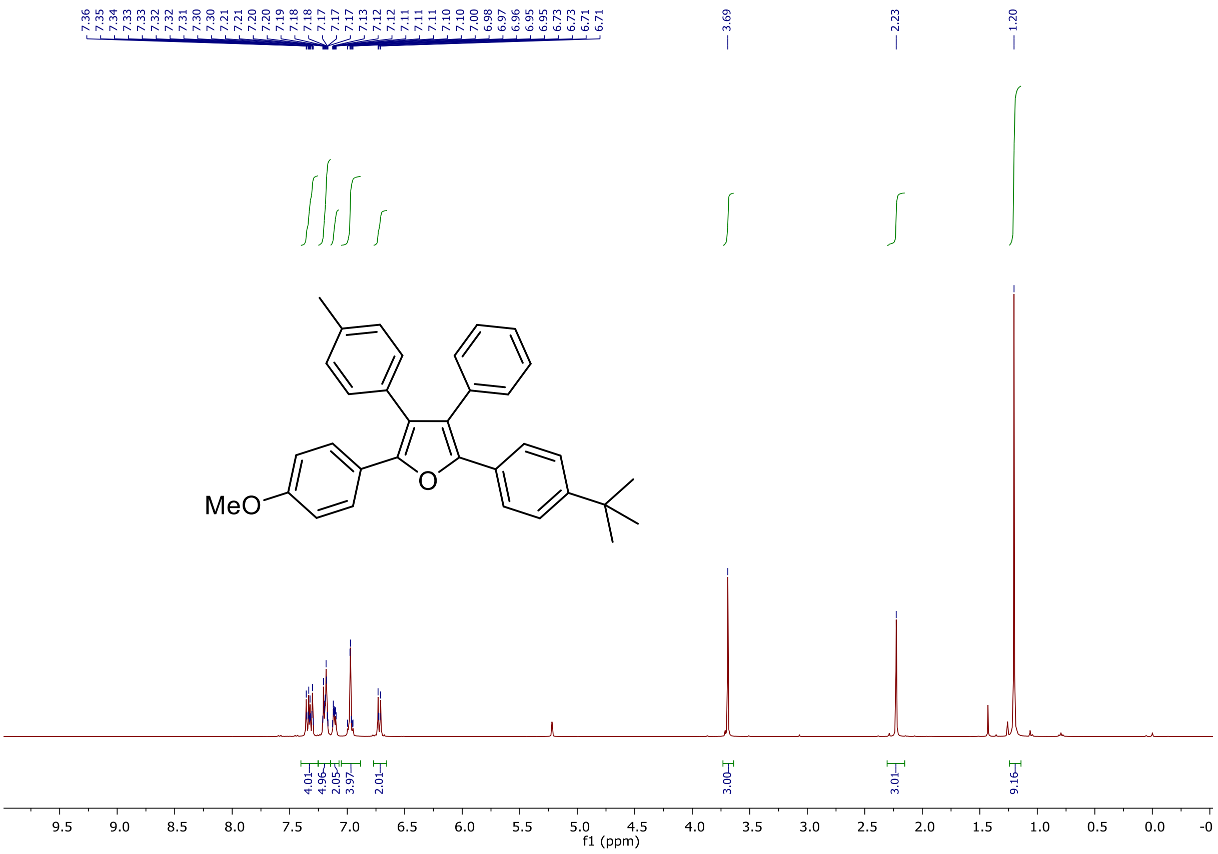

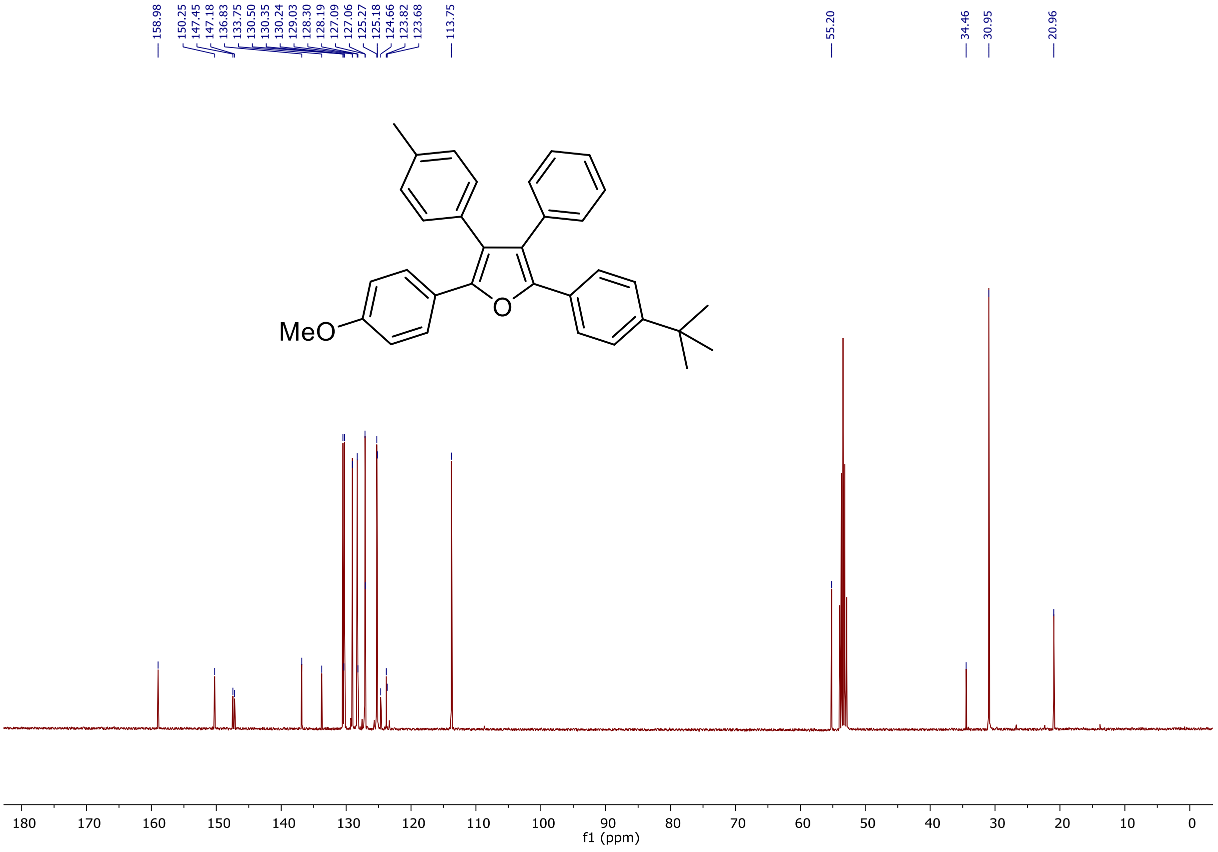

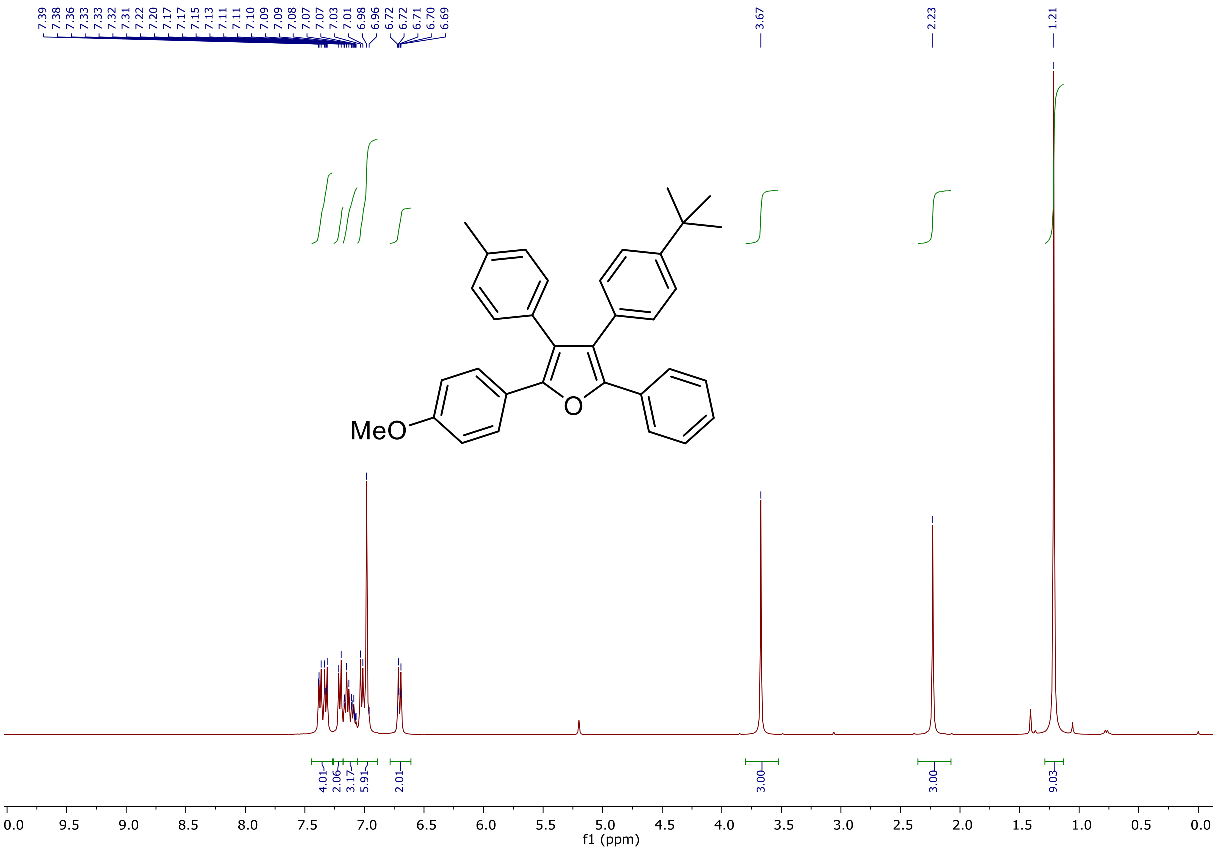
7.39 7.38 7.36 7.33 7.33 7.32 7.31 7.22 7.20 7.17 7.17 7.15 7.13 7.11 7.11 7.10 7.09 7.09 7.08 7.07 7.07 7.03 7.01 6.98 6.96 6.72 6.72 6.71 6.70 6.69
3.67
2.23
1.21
MeO
O
4.01
2.06
3.17
5.91
2.01
3.00
3.00
9.03
f1 (ppm)

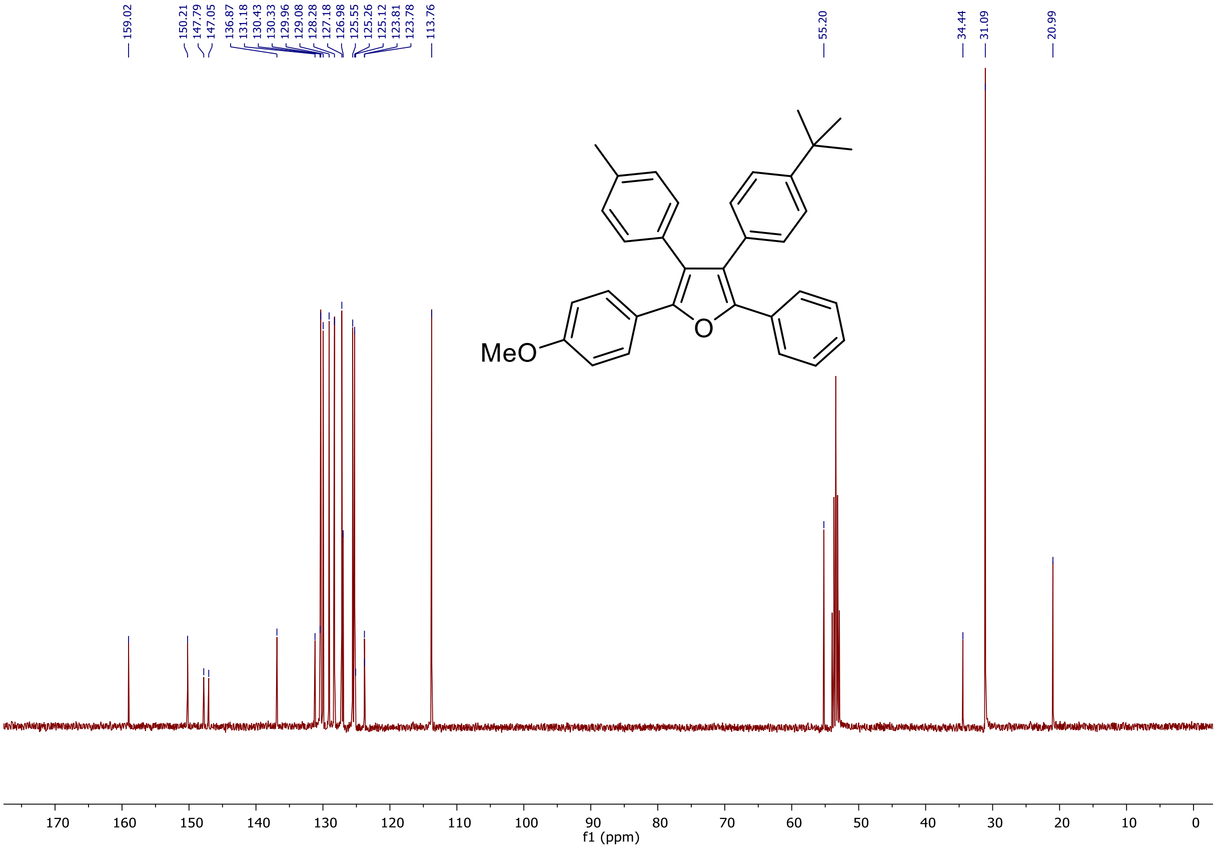
159.02
150.21 147.79 147.05
136.87 131.18 130.43 130.33 129.96 129.08 128.28 127.18 126.98 125.55 125.26 125.12 123.81 123.78
113.76
55.20
34.44 31.09
20.99
MeO
O
f1 (ppm)

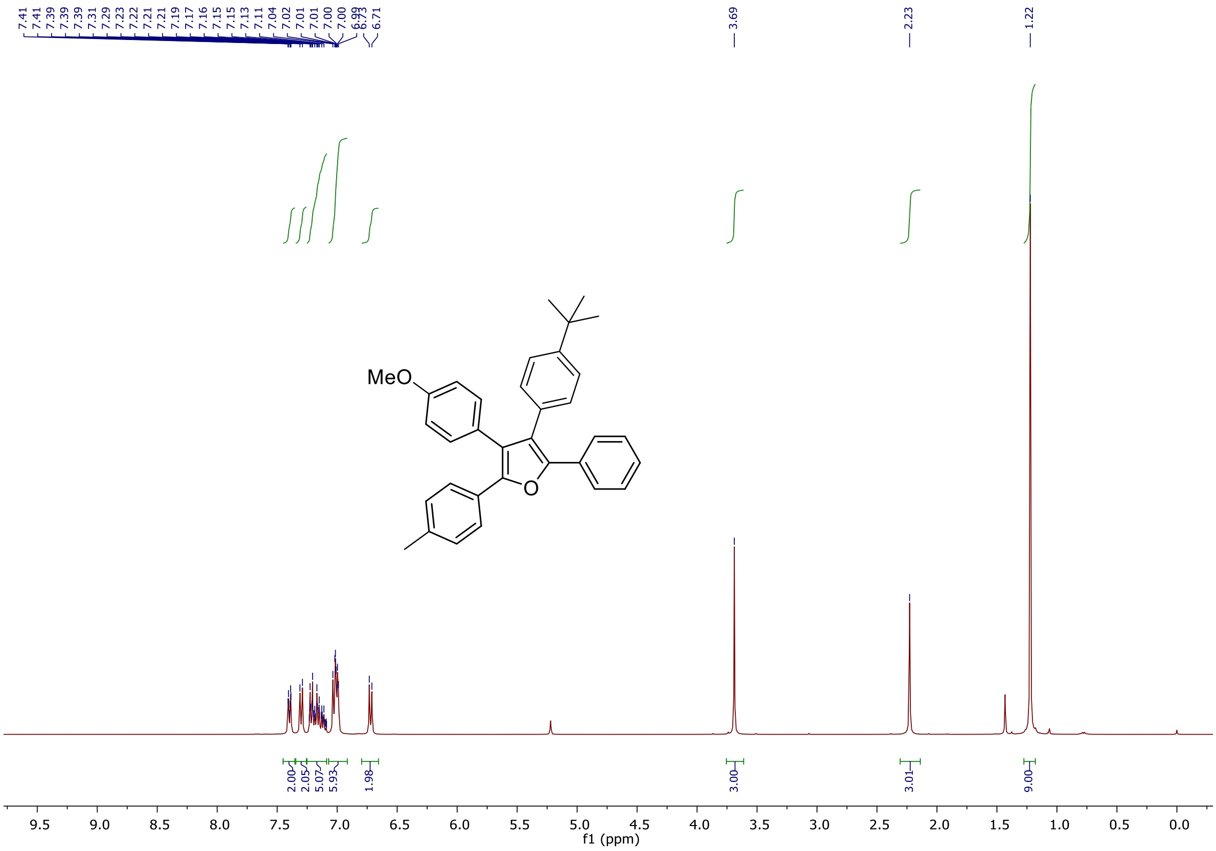
7.41
7.41
7.39
7.39
7.39
7.31
7.29
7.23
7.22
7.21
7.21
7.19
7.17
7.16
7.15
7.15
7.13
7.11
7.04
7.02
7.01
7.01
7.00
7.00
6.99
6.73
6.71
3.69
2.23
1.22
MeO
O
2.00
2.05
5.07
5.93
1.98
3.00
3.01
9.00
f1 (ppm)

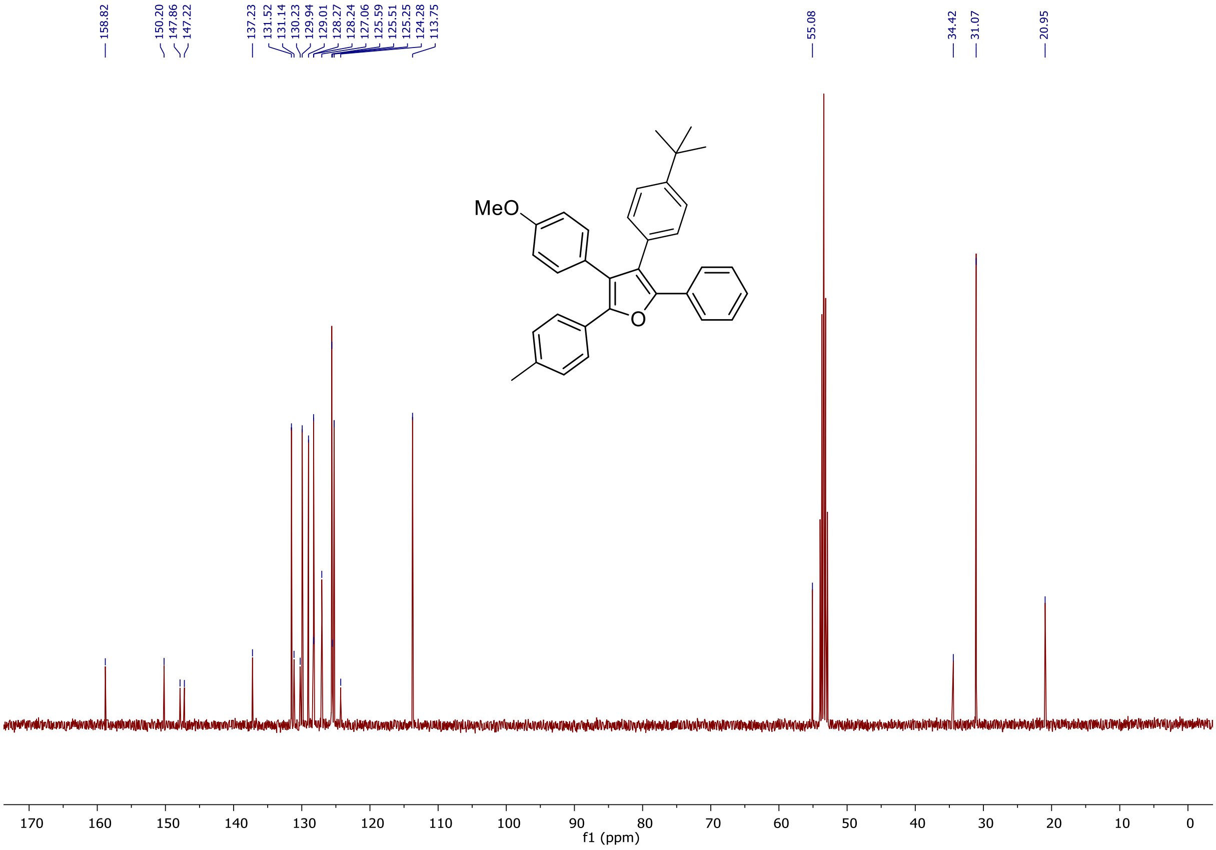
158.82
150.20
147.86
147.22
137.23
131.52
131.14
130.23
129.94
129.01
128.27
128.24
127.06
125.59
125.51
125.25
124.28
113.75
55.08
34.42
31.07
20.95
MeO
O
f1 (ppm)

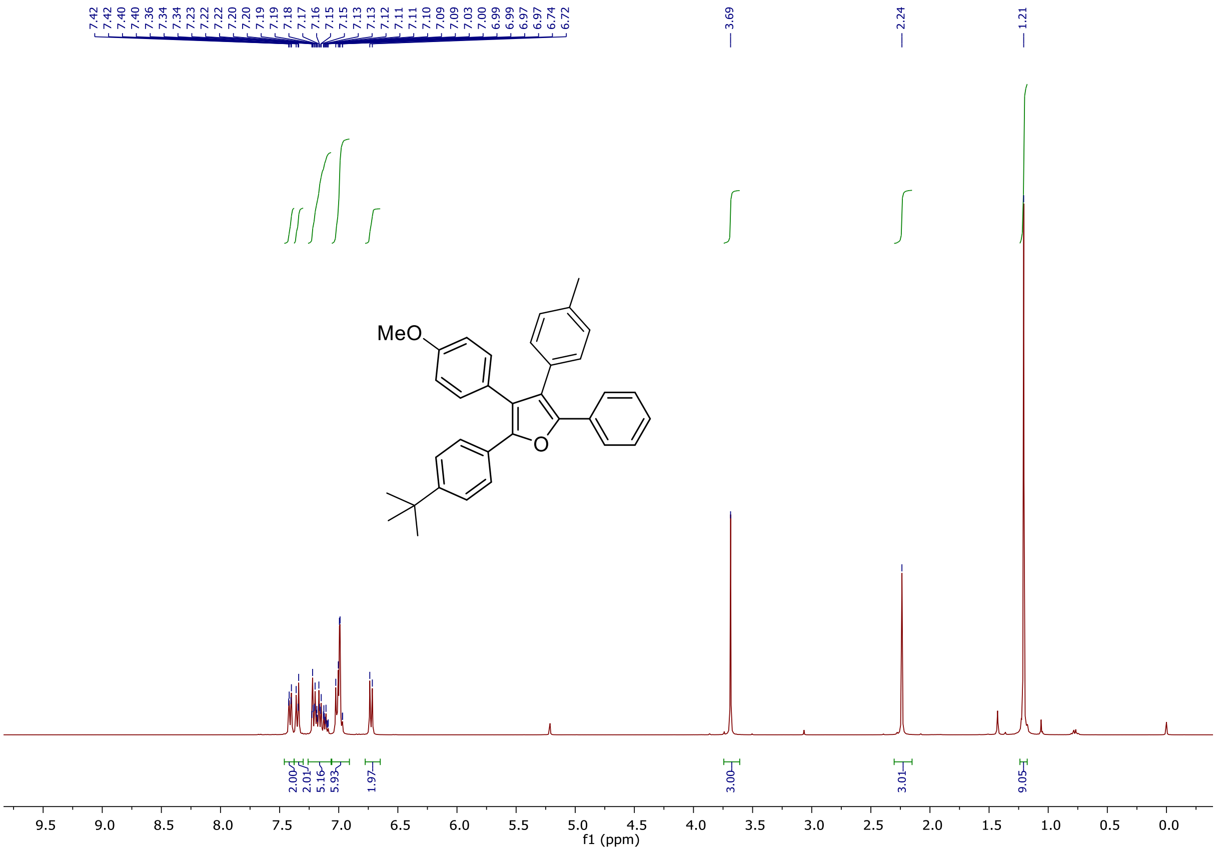
7.42
7.42
7.40
7.36
7.34
7.34
7.23
7.22
7.22
7.20
7.19
7.19
7.18
7.17
7.16
7.15
7.15
7.13
7.13
7.12
7.11
7.10
7.09
7.09
7.03
7.00
6.99
6.99
6.97
6.97
6.74
6.72
3.69
2.24
1.21
MeO
O
2.00
2.01
5.16
5.93
1.97
3.00
3.01
9.05
f1 (ppm)

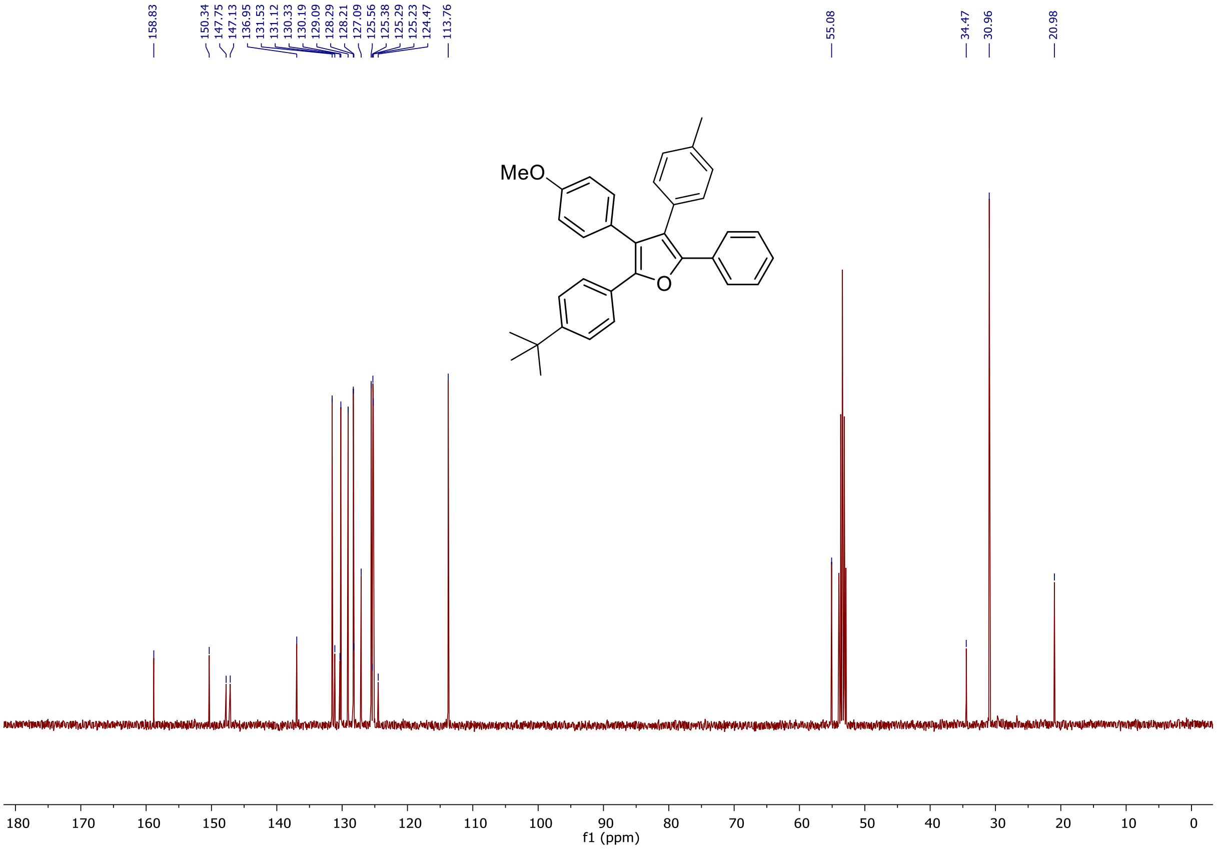
158.83
150.34
147.75
147.13
136.95
131.53
131.12
130.33
130.19
129.09
128.29
128.21
127.09
125.56
125.38
125.29
125.23
124.47
113.76
55.08
34.47
30.96
20.98
MeO
O
f1 (ppm)

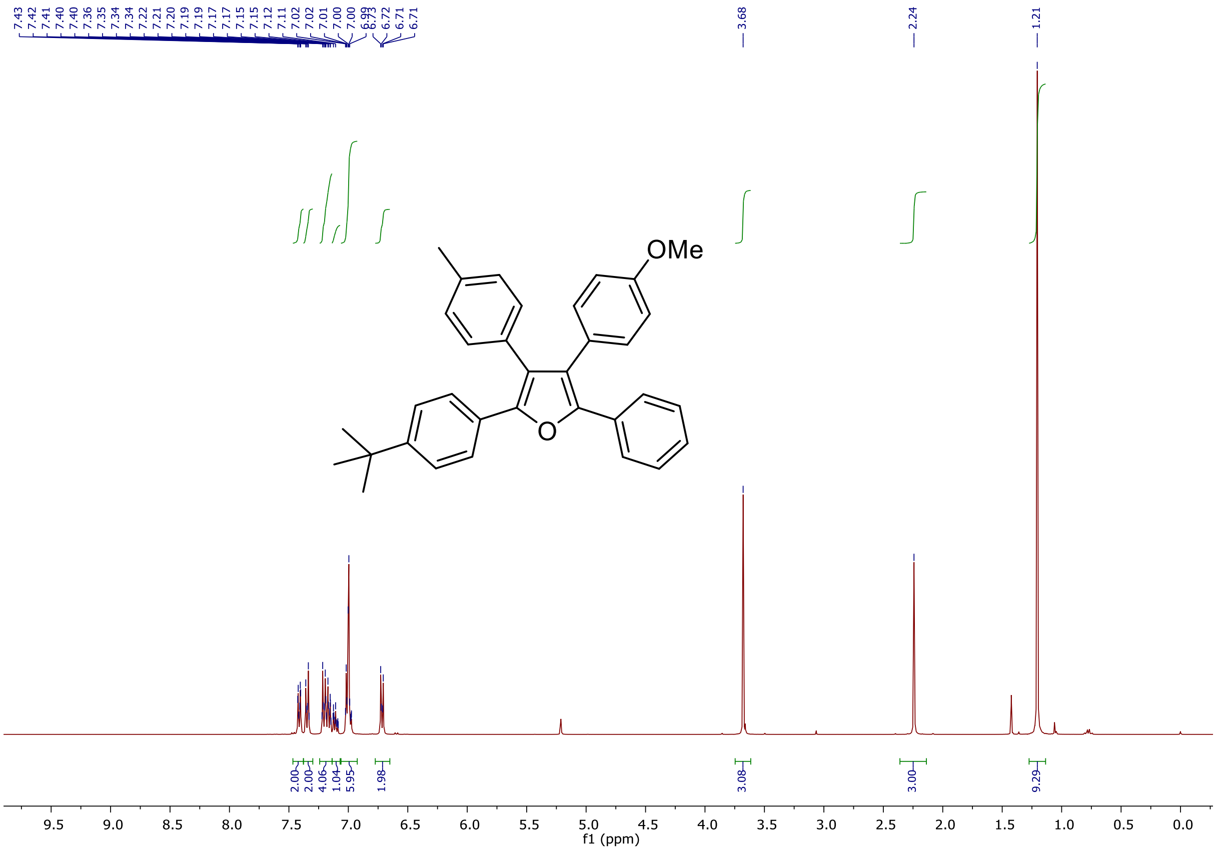
7.43
7.42
7.41
7.40
7.36
7.35
7.34
7.34
7.22
7.21
7.20
7.19
7.19
7.17
7.17
7.15
7.15
7.12
7.11
7.02
7.02
7.01
7.00
7.00
6.99
6.73
6.72
6.71
3.68
2.24
1.21
OMe
O
2.00
2.00
4.06
1.04
5.95
1.98
3.08
3.00
9.29
f1 (ppm)

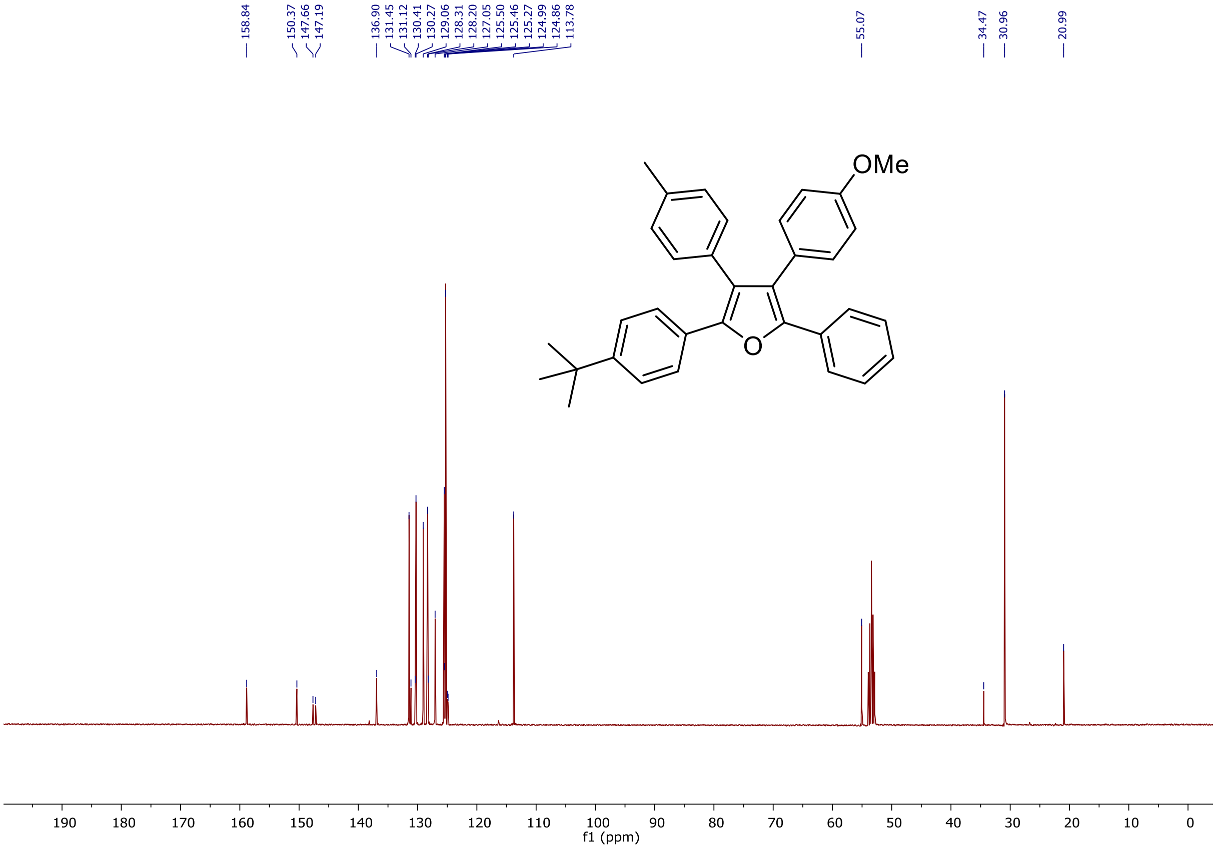
158.84
150.37
147.66
147.19
136.90
131.45
131.12
130.41
130.27
129.06
128.31
128.20
127.05
125.50
125.46
125.27
124.99
124.86
113.78
55.07
34.47
30.96
20.99
OMe
O
f1 (ppm)

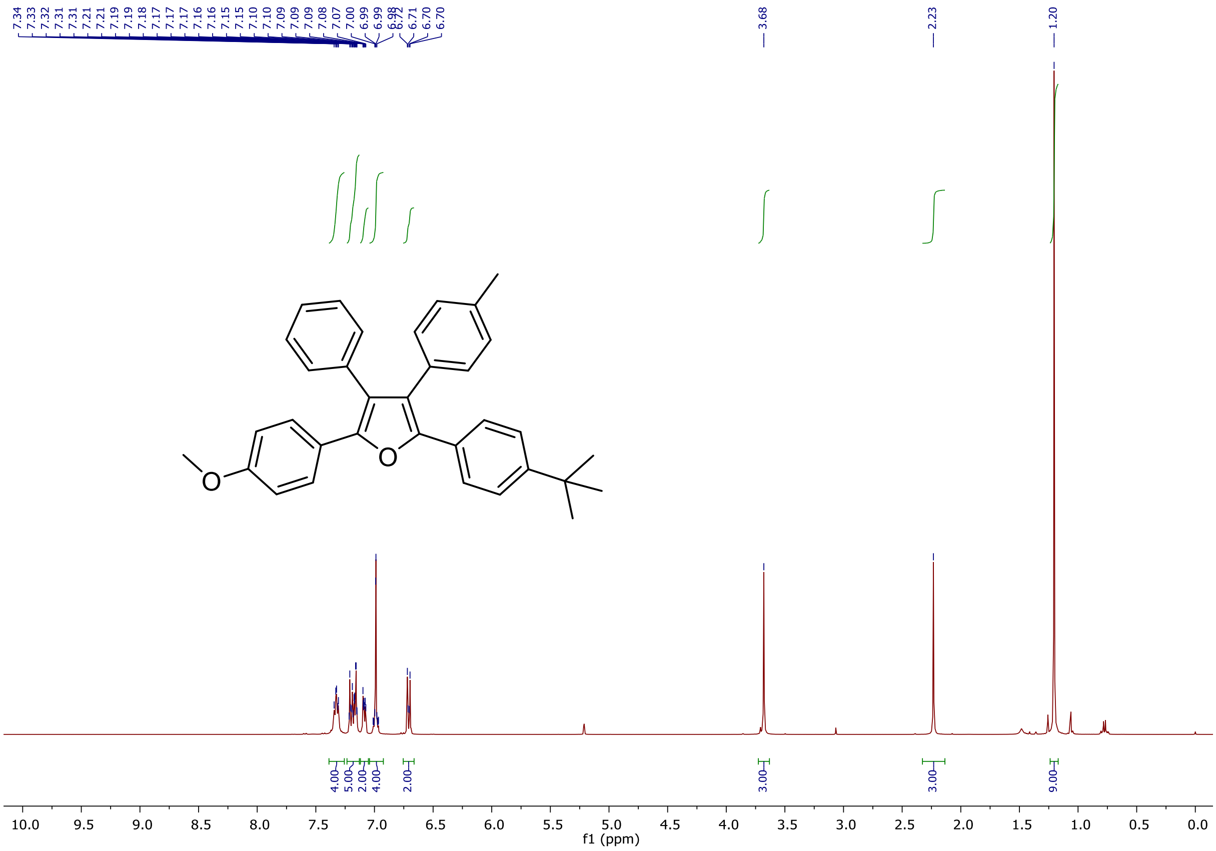
7.34 7.33 7.32 7.31 7.31 7.21 7.21 7.19 7.18 7.17 7.17 7.17 7.16 7.16 7.15 7.15 7.10 7.10 7.09 7.09 7.09 7.09 7.08 7.07 7.00 6.99 6.99 6.73 6.72 6.71 6.70 6.70
3.68
2.23
1.20
O
4.00 5.00 2.00 4.00 2.00
3.00
3.00
9.00
10.0 9.5 9.0 8.5 8.0 7.5 7.0 6.5 6.0 5.5 5.0 4.5 4.0 3.5 3.0 2.5 2.0 1.5 1.0 0.5 0.0
f1 (ppm)

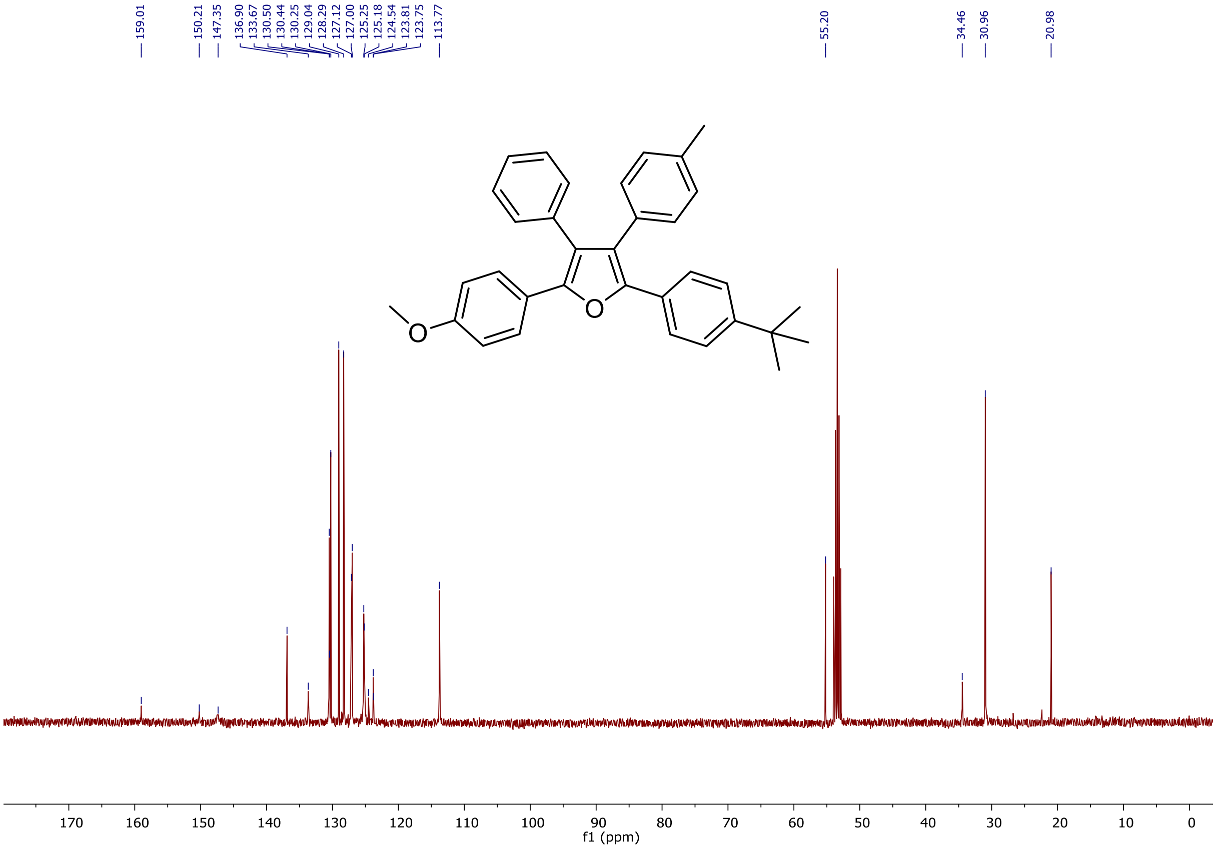
159.01
150.21
147.35
136.90 133.67 130.50 130.44 130.25 129.04 128.29 127.12 127.00 125.25 125.18 124.54 123.81 123.75
113.77
55.20
34.46
30.96
20.98
O
170 160 150 140 130 120 110 100 90 80 70 60 50 40 30 20 10 0
f1 (ppm)

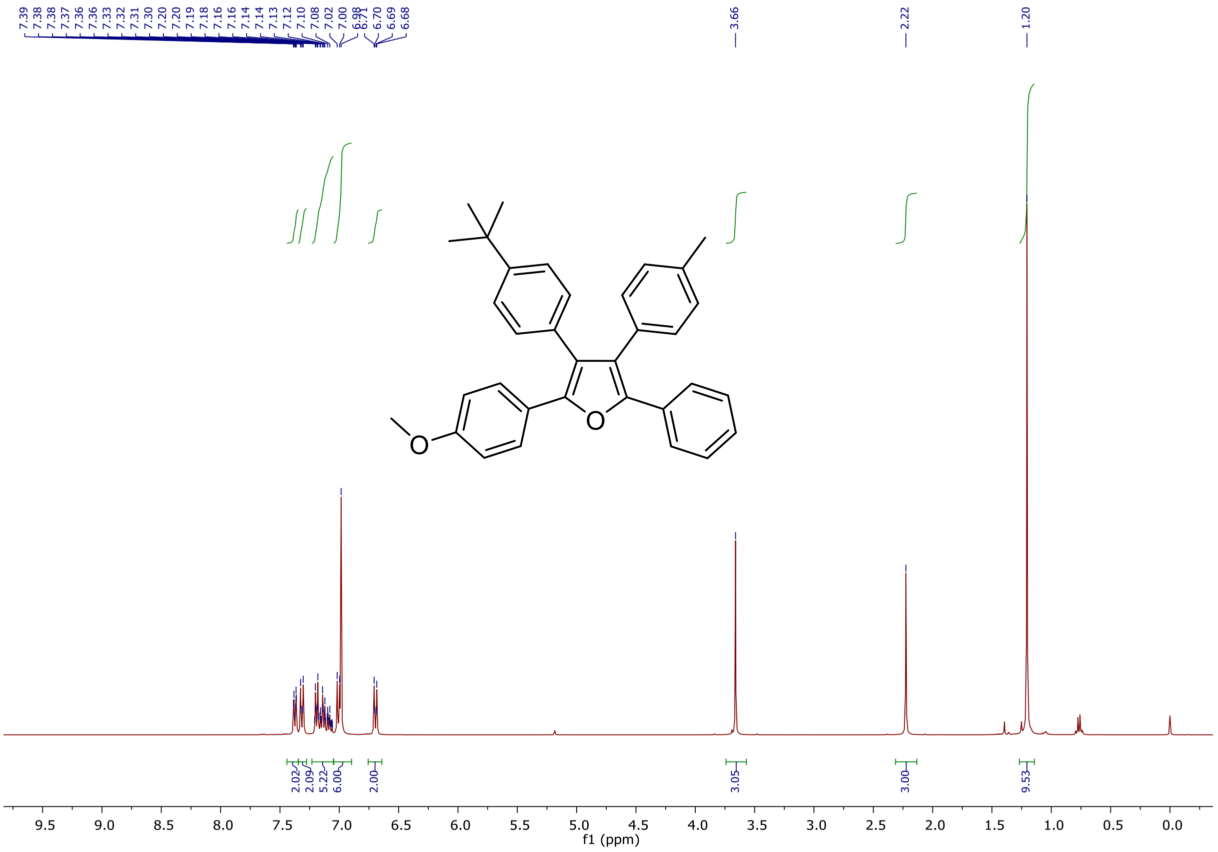

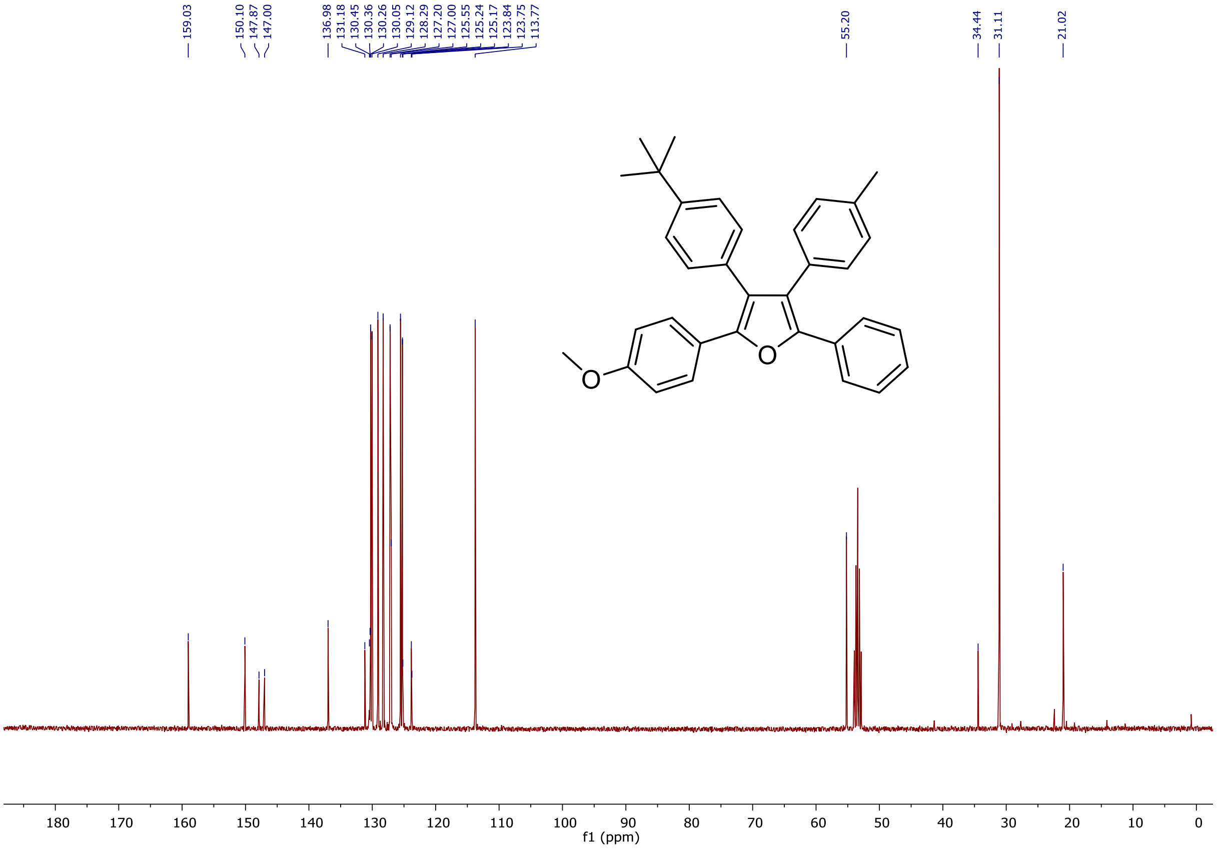

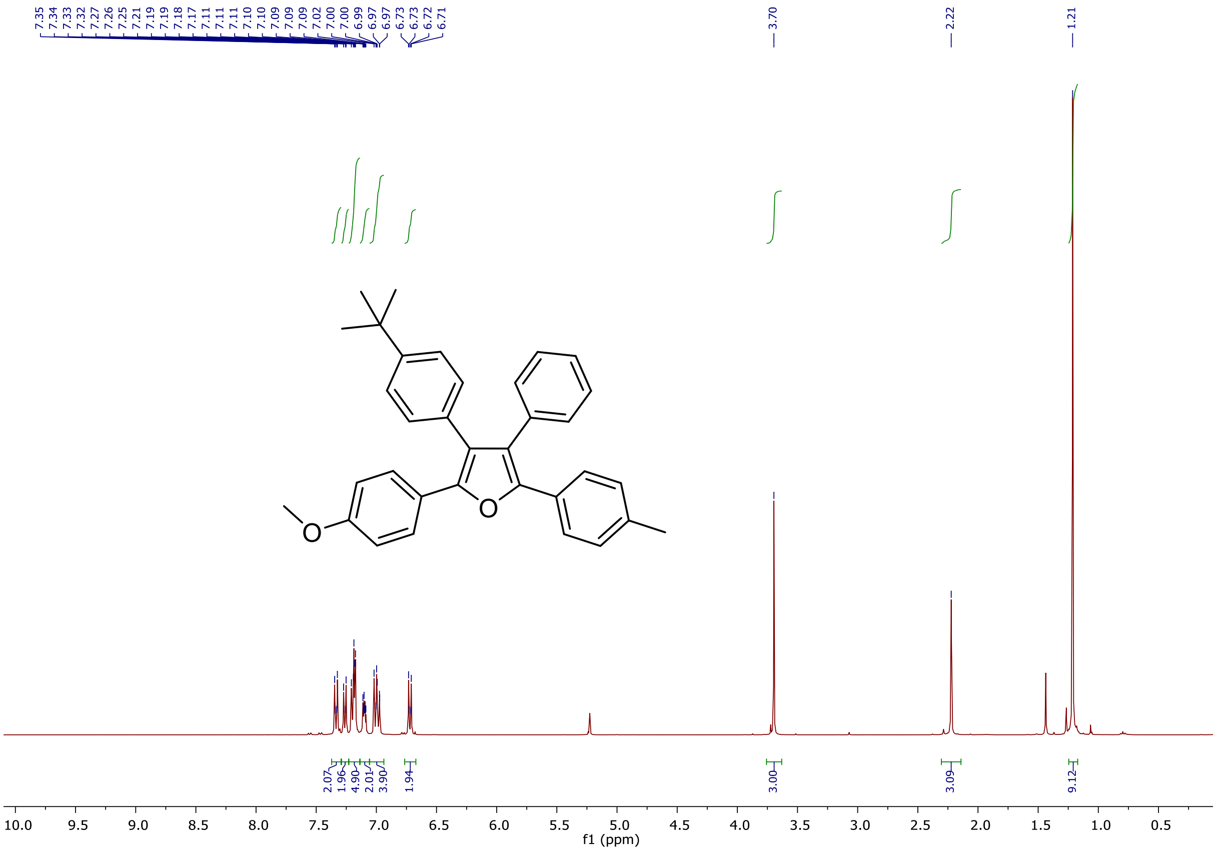
7.35
7.34
7.33
7.32
7.27
7.26
7.25
7.21
7.19
7.19
7.18
7.17
7.11
7.11
7.11
7.10
7.09
7.09
7.09
7.02
7.00
7.00
6.99
6.97
6.97
6.73
6.73
6.72
6.71
3.70
2.22
1.21
2.07
1.96
4.90
2.01
3.90
1.94
3.00
3.09
9.12
O
f1 (ppm)

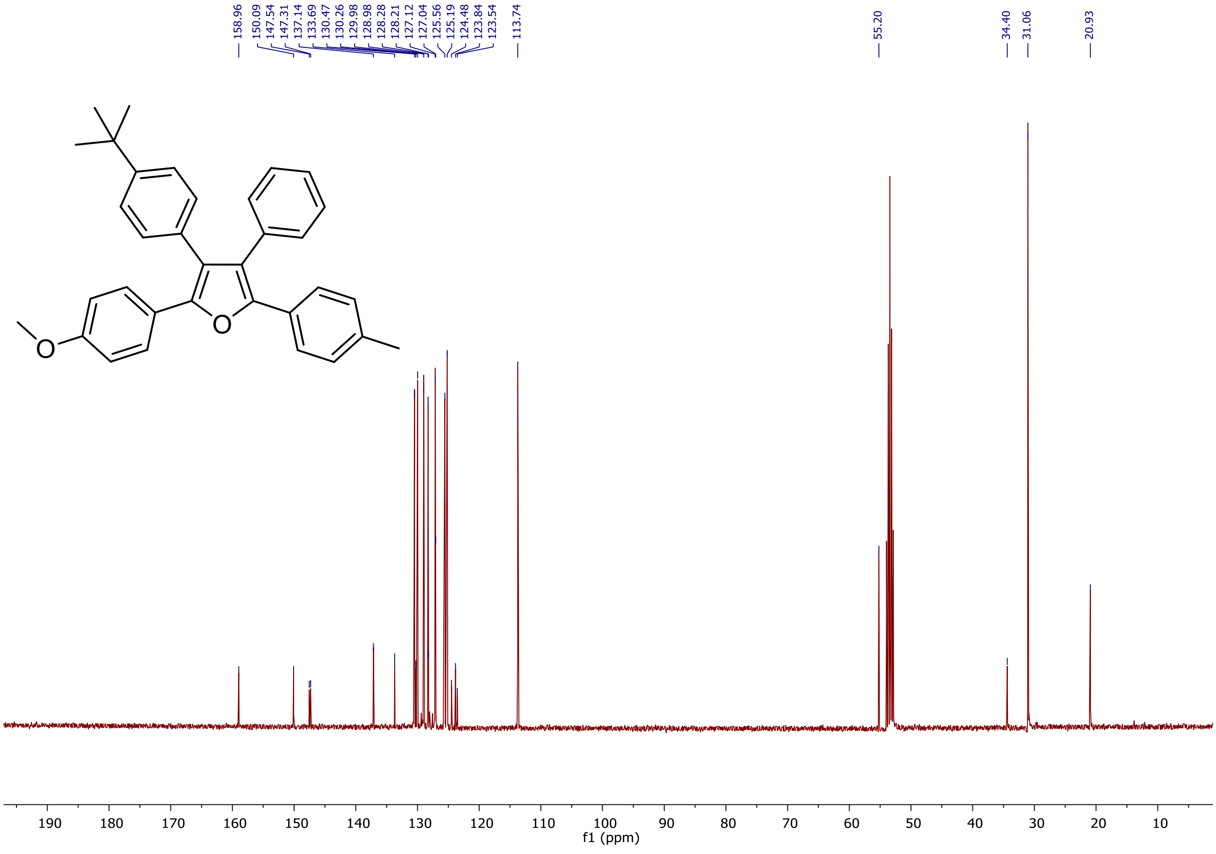
158.96
150.09
147.54
147.31
137.14
133.69
130.47
130.26
129.98
128.98
128.28
128.21
127.12
127.04
125.56
125.19
124.48
123.84
123.54
113.74
55.20
34.40
31.06
20.93
O
f1 (ppm)

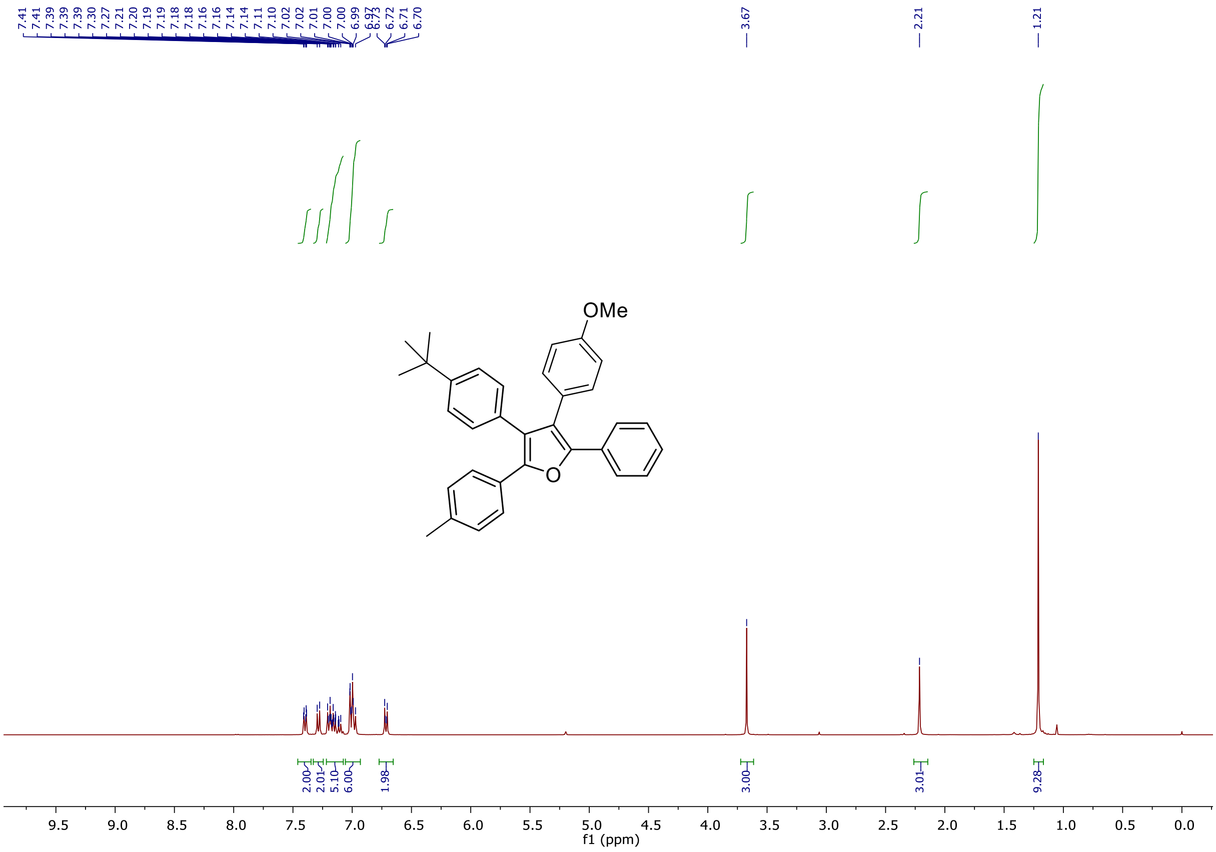

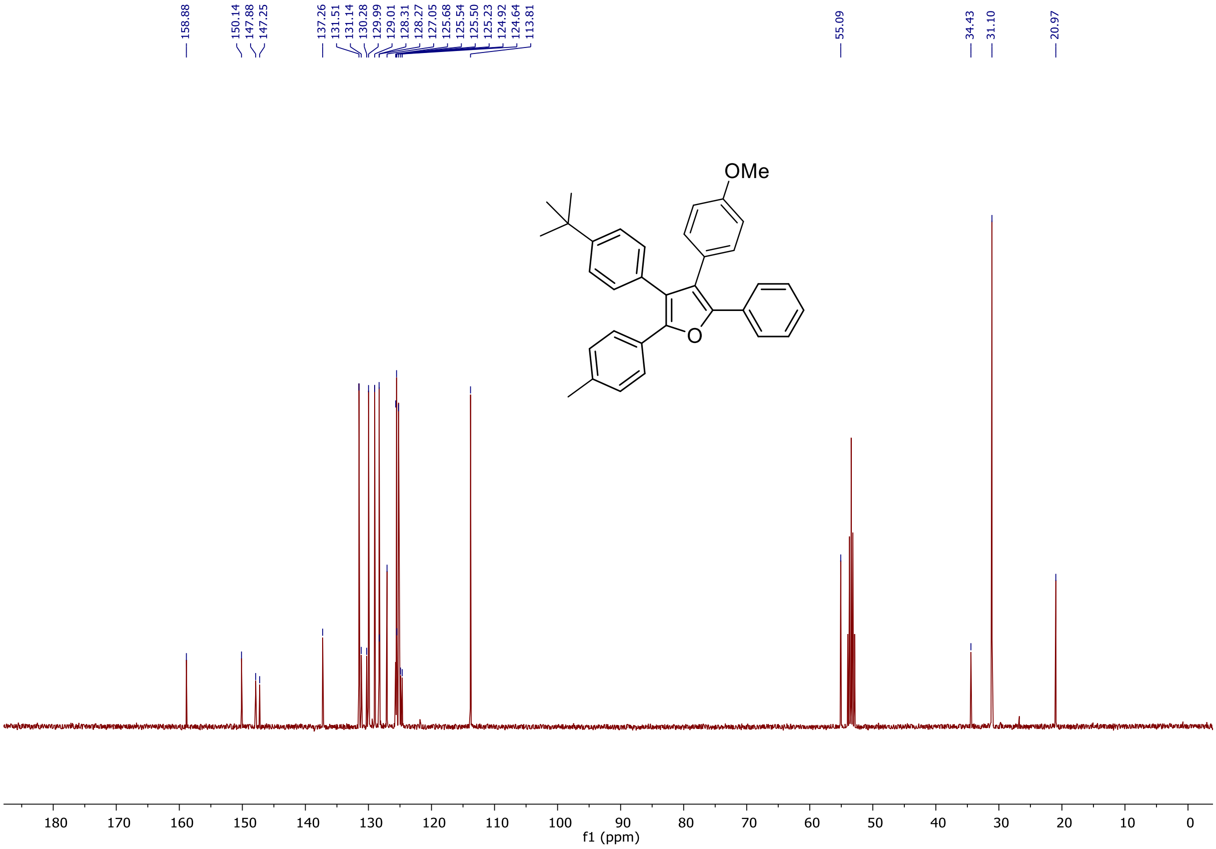

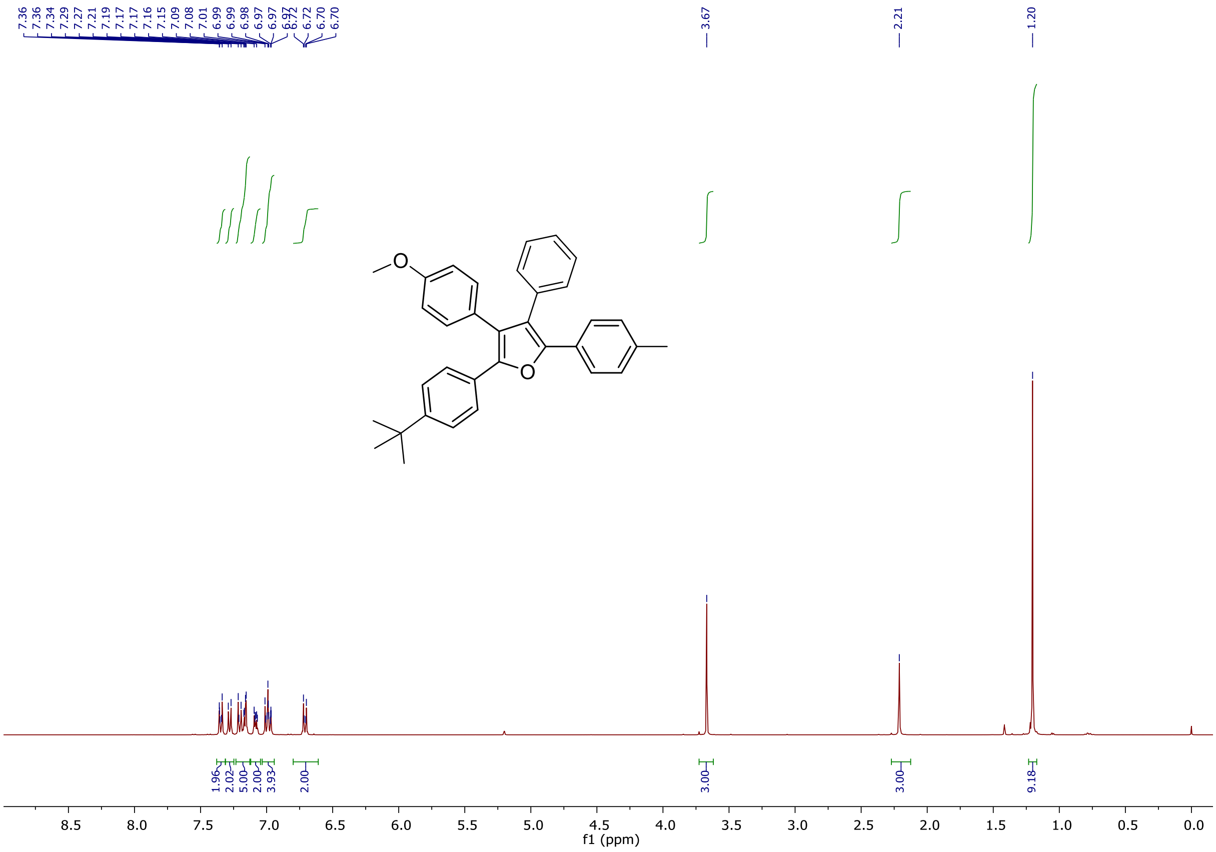

7.36
7.36
7.34
7.29
7.27
7.21
7.19
7.17
7.17
7.16
7.15
7.09
7.08
7.01
6.99
6.99
6.98
6.97
6.97
6.97
6.72
6.72
6.70
6.70
3.67
2.21
1.20
1.96
2.02
5.00
2.00
3.93
2.00
3.00
3.00
9.18
O
O
8.5
8.0
7.5
7.0
6.5
6.0
5.5
5.0
4.5
4.0
3.5
3.0
2.5
2.0
1.5
1.0
0.5
0.0
f1 (ppm)

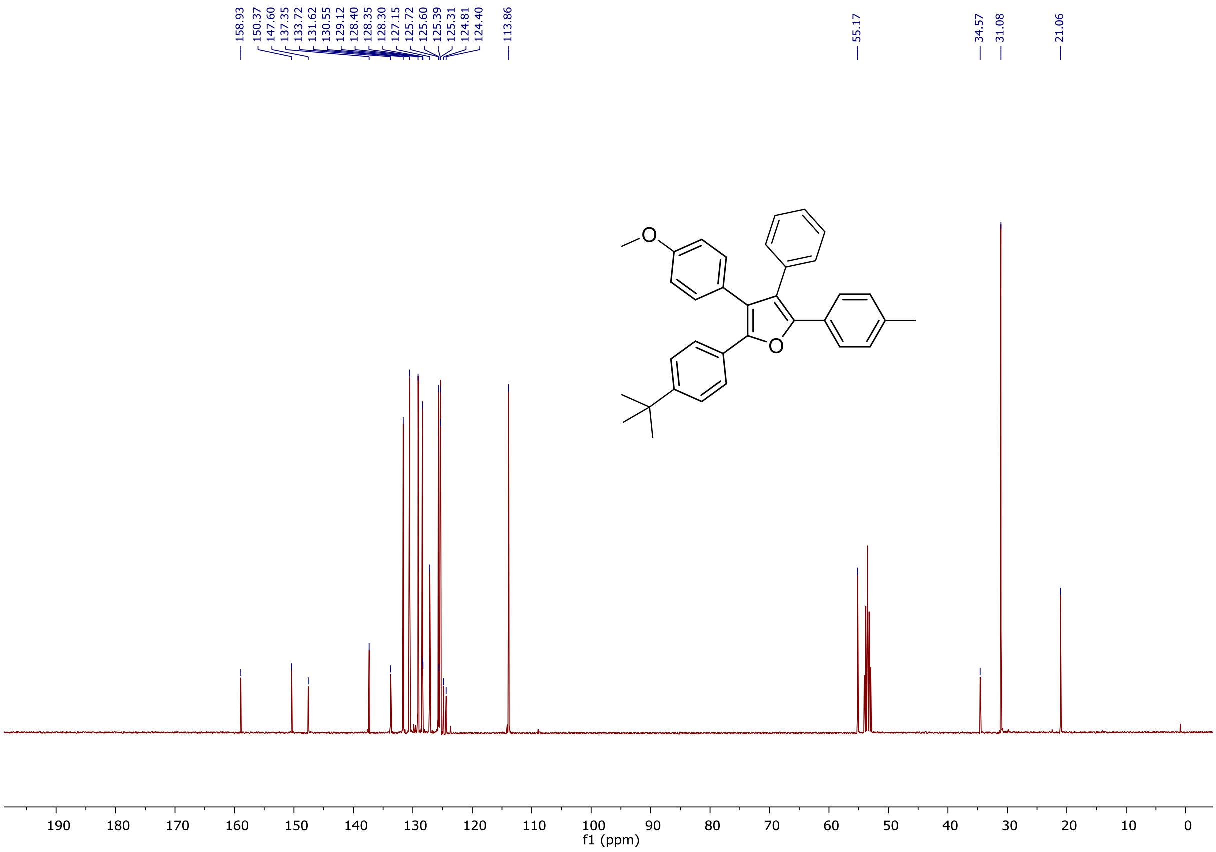

158.93
150.37
147.60
137.35
133.72
131.62
130.55
129.12
128.40
128.35
128.30
127.15
125.72
125.60
125.39
125.31
124.81
124.40
113.86
55.17
34.57
31.08
21.06
O
O
190
180
170
160
150
140
130
120
110
100
90
80
70
60
50
40
30
20
10
0
f1 (ppm)

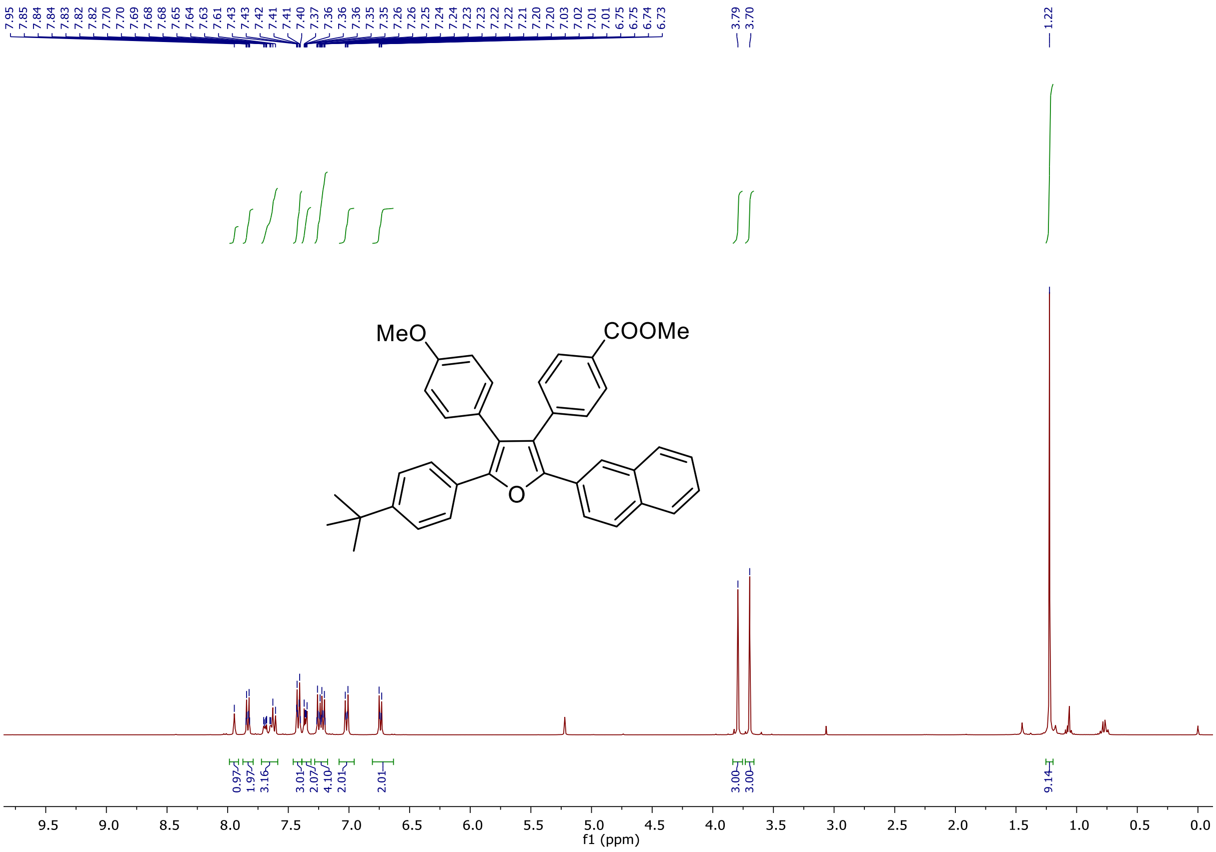
7.95 7.85 7.84 7.84 7.83 7.82 7.82 7.70 7.70 7.69 7.68 7.68 7.65 7.64 7.63 7.61 7.43 7.42 7.41 7.40 7.37 7.36 7.36 7.35 7.26 7.26 7.25 7.24 7.24 7.23 7.23 7.22 7.22 7.21 7.20 7.20 7.03 7.02 7.01 6.75 6.75 6.74 6.73
3.79
3.70
1.22
MeO
COOMe
O
0.97
1.97
3.16
3.01
2.07
4.10
2.01
2.01
3.00
3.00
9.14
9.5 9.0 8.5 8.0 7.5 7.0 6.5 6.0 5.5 5.0 4.5 4.0 3.5 3.0 2.5 2.0 1.5 1.0 0.5 0.0
f1 (ppm)

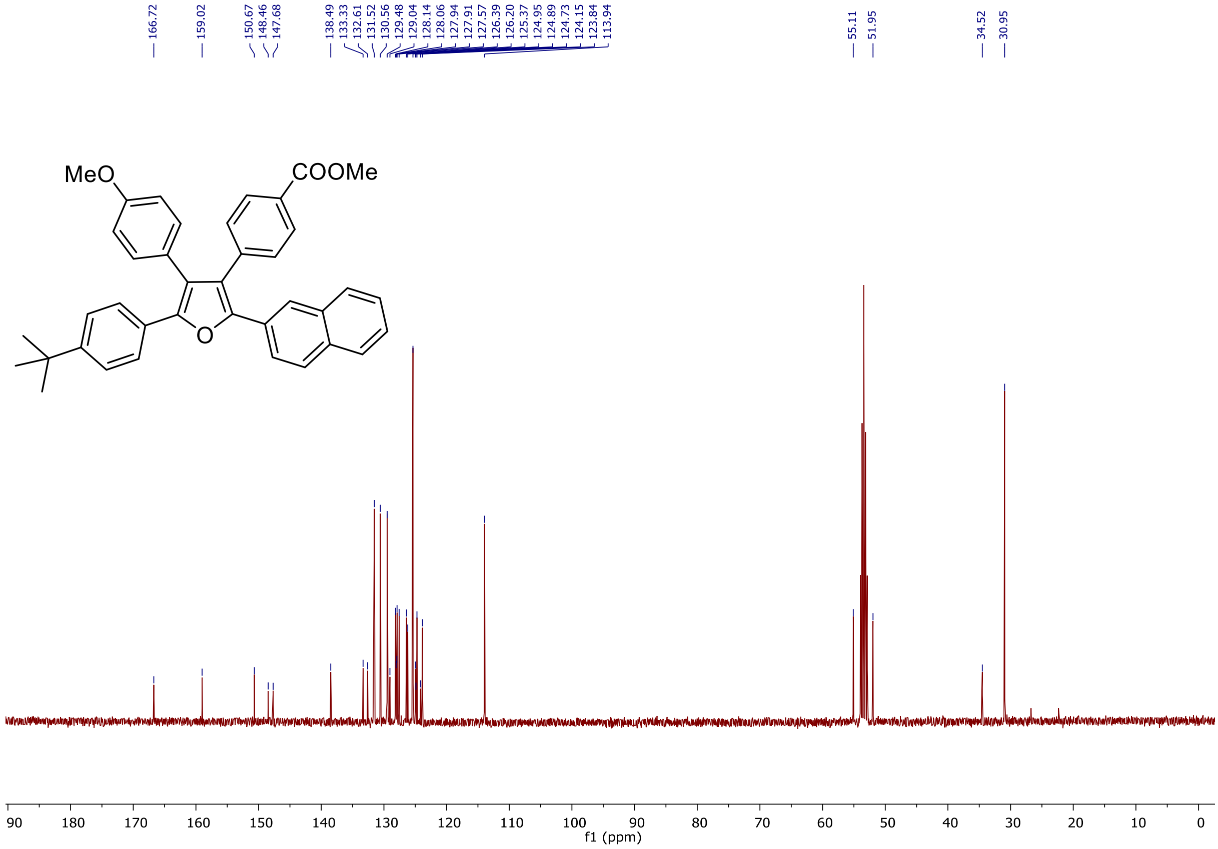
166.72
159.02
150.67 148.46 147.68
138.49 133.33 132.61 131.52 130.56 129.48 129.04 128.14 128.06 127.94 127.91 127.57 126.39 126.20 125.37 124.95 124.89 124.73 124.15 123.84 113.94
55.11
51.95
34.52
30.95
MeO
COOMe
O
90 180 170 160 150 140 130 120 110 100 90 80 70 60 50 40 30 20 10 0
f1 (ppm)

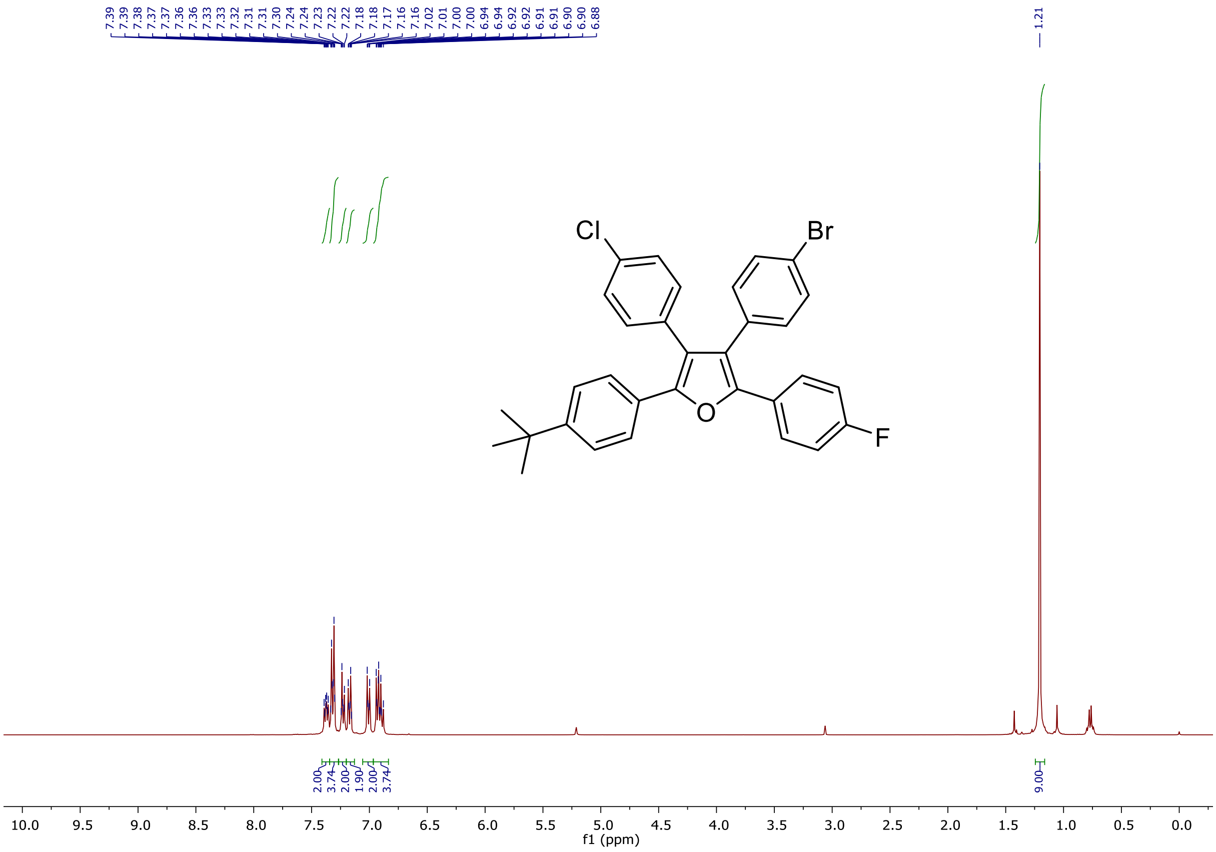
7.39 7.39 7.38 7.37 7.37 7.36 7.36 7.33 7.33 7.32 7.31 7.31 7.30 7.24 7.24 7.23 7.22 7.22 7.18 7.18 7.17 7.16 7.16 7.02 7.01 7.00 7.00 6.94 6.94 6.92 6.92 6.91 6.91 6.90 6.88
1.21
Cl
Br
O
F
2.00 3.74 2.00 1.90 2.00 3.74
9.00
10.0 9.5 9.0 8.5 8.0 7.5 7.0 6.5 6.0 5.5 5.0 4.5 4.0 3.5 3.0 2.5 2.0 1.5 1.0 0.5 0.0
f1 (ppm)

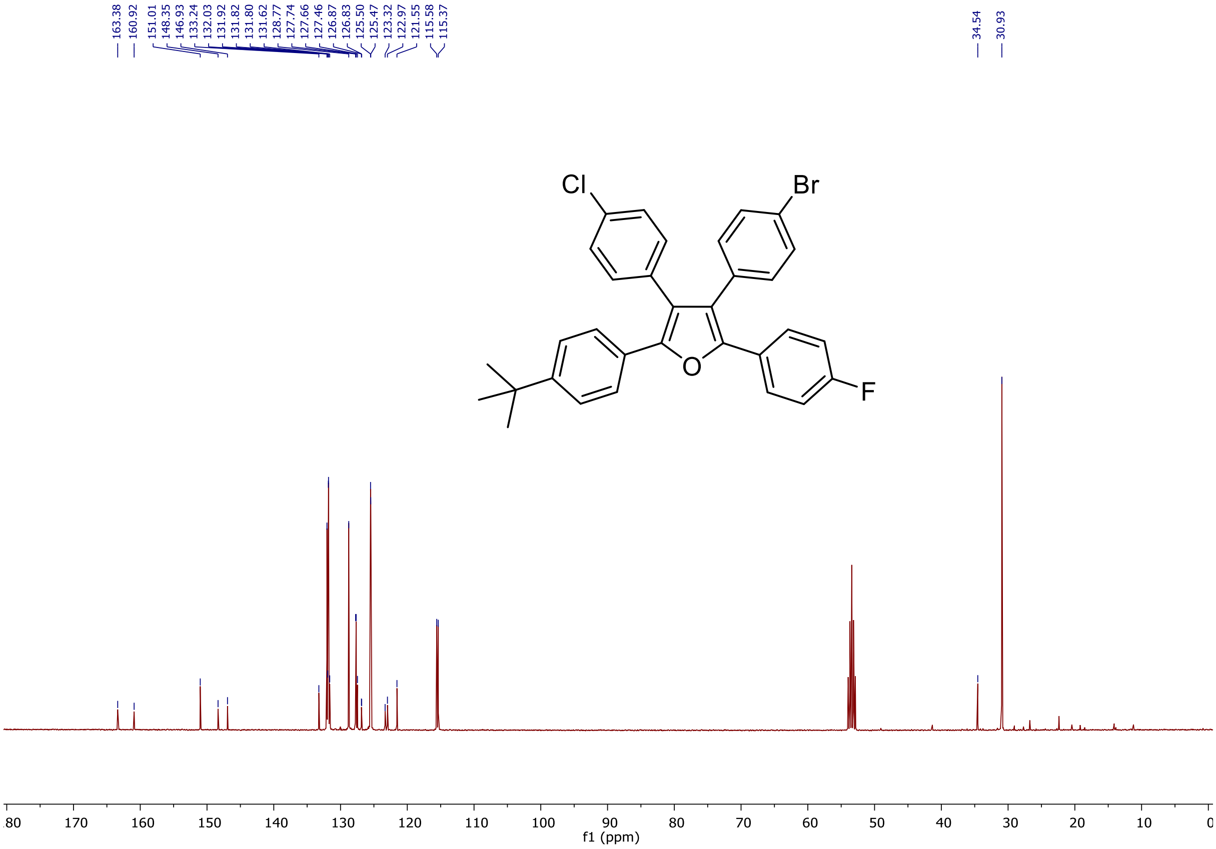
163.38 160.92 151.01 148.35 146.93 133.24 132.03 131.92 131.82 131.80 131.62 128.77 127.74 127.66 127.46 126.87 126.83 125.50 125.47 123.32 122.97 121.55 115.58 115.37
34.54 30.93
Cl
Br
O
F
180 170 160 150 140 130 120 110 100 90 80 70 60 50 40 30 20 10 0
f1 (ppm)

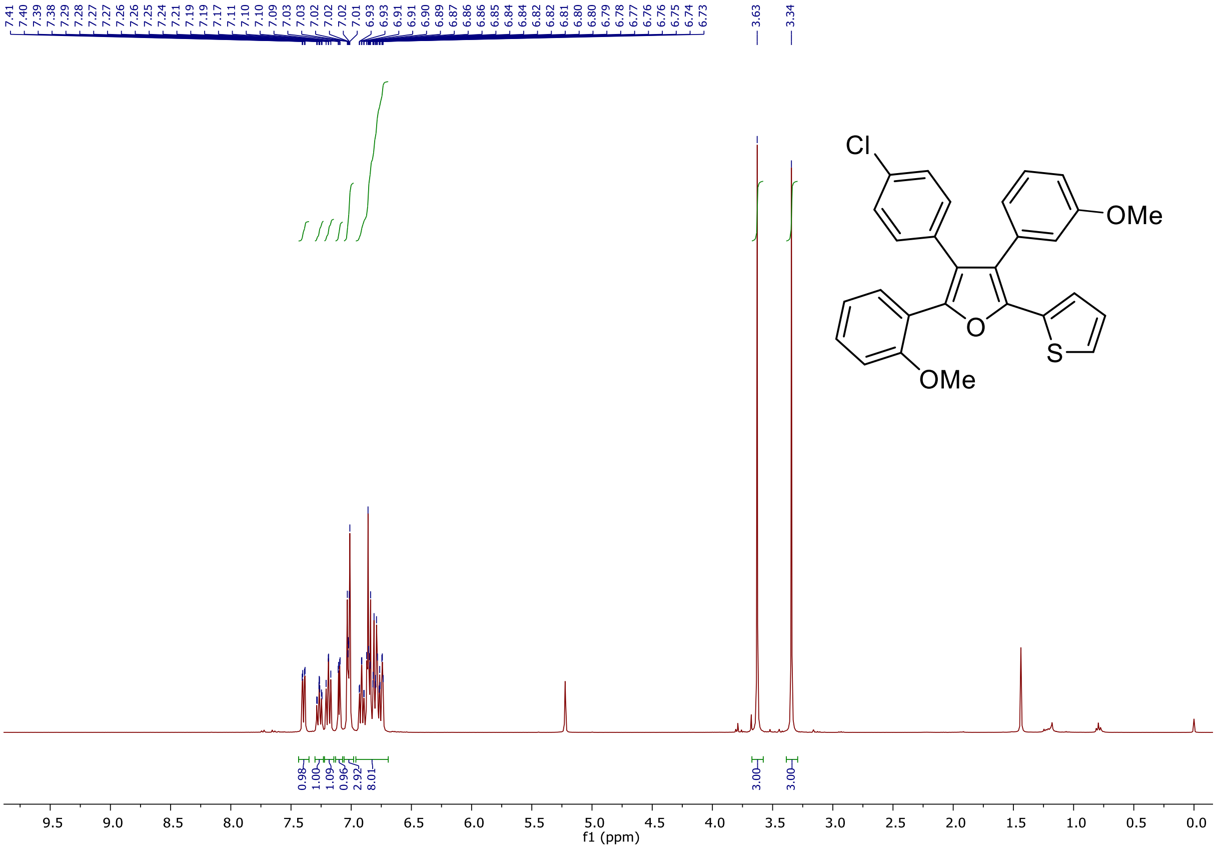
7.41
7.40
7.39
7.38
7.29
7.28
7.27
7.27
7.26
7.26
7.25
7.24
7.21
7.19
7.19
7.17
7.11
7.10
7.10
7.09
7.03
7.03
7.02
7.02
7.02
7.01
6.93
6.93
6.91
6.91
6.90
6.89
6.87
6.86
6.86
6.85
6.84
6.84
6.82
6.82
6.81
6.80
6.80
6.79
6.78
6.77
6.76
6.76
6.75
6.74
6.73
3.63
3.34
Cl
OMe
O
S
OMe
0.98
1.00
1.09
0.96
2.92
8.01
3.00
3.00
9.5 9.0 8.5 8.0 7.5 7.0 6.5 6.0 5.5 5.0 4.5 4.0 3.5 3.0 2.5 2.0 1.5 1.0 0.5 0.0
f1 (ppm)

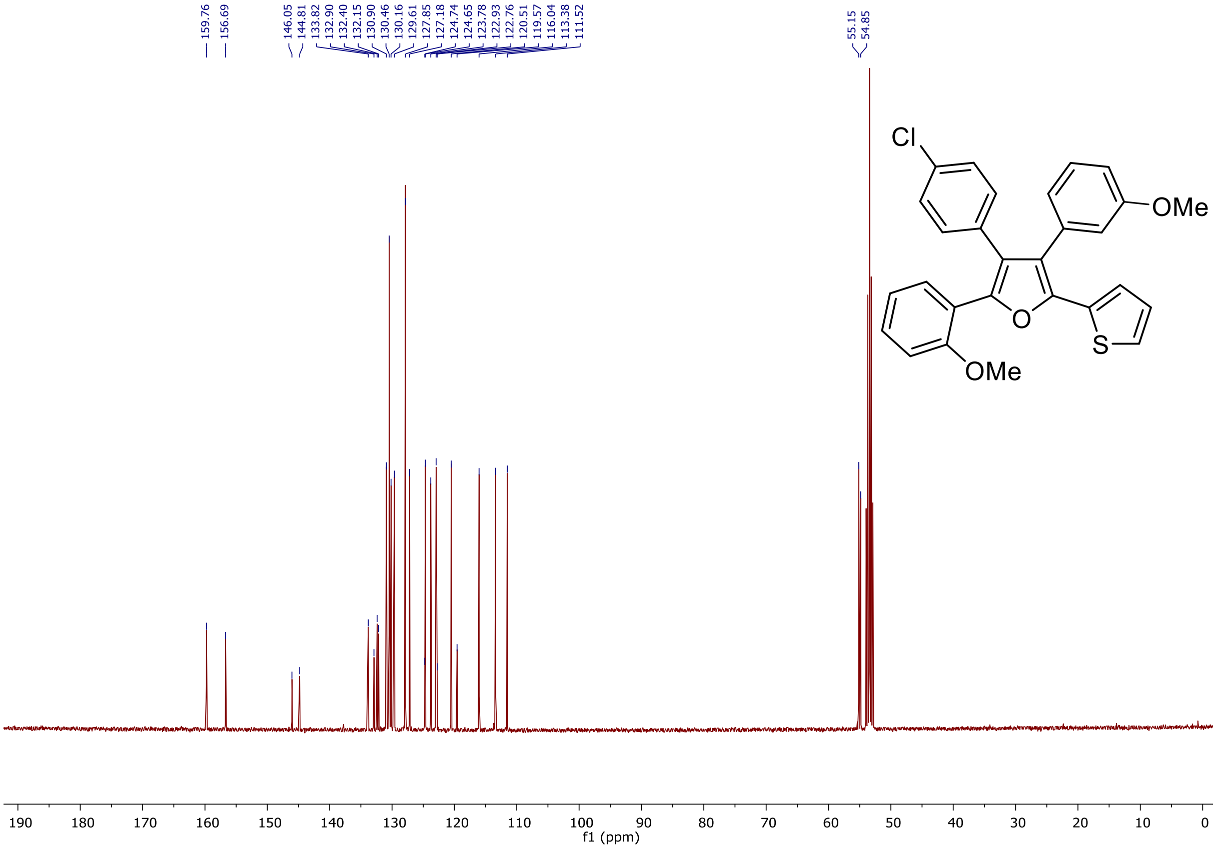
159.76
156.69
146.05
144.81
133.82
132.90
132.40
132.15
130.90
130.46
130.16
129.61
127.85
127.18
124.74
124.65
123.78
122.93
122.76
120.51
119.57
116.04
113.38
111.52
55.15
54.85
Cl
OMe
O
S
OMe
190 180 170 160 150 140 130 120 110 100 90 80 70 60 50 40 30 20 10 0
f1 (ppm)

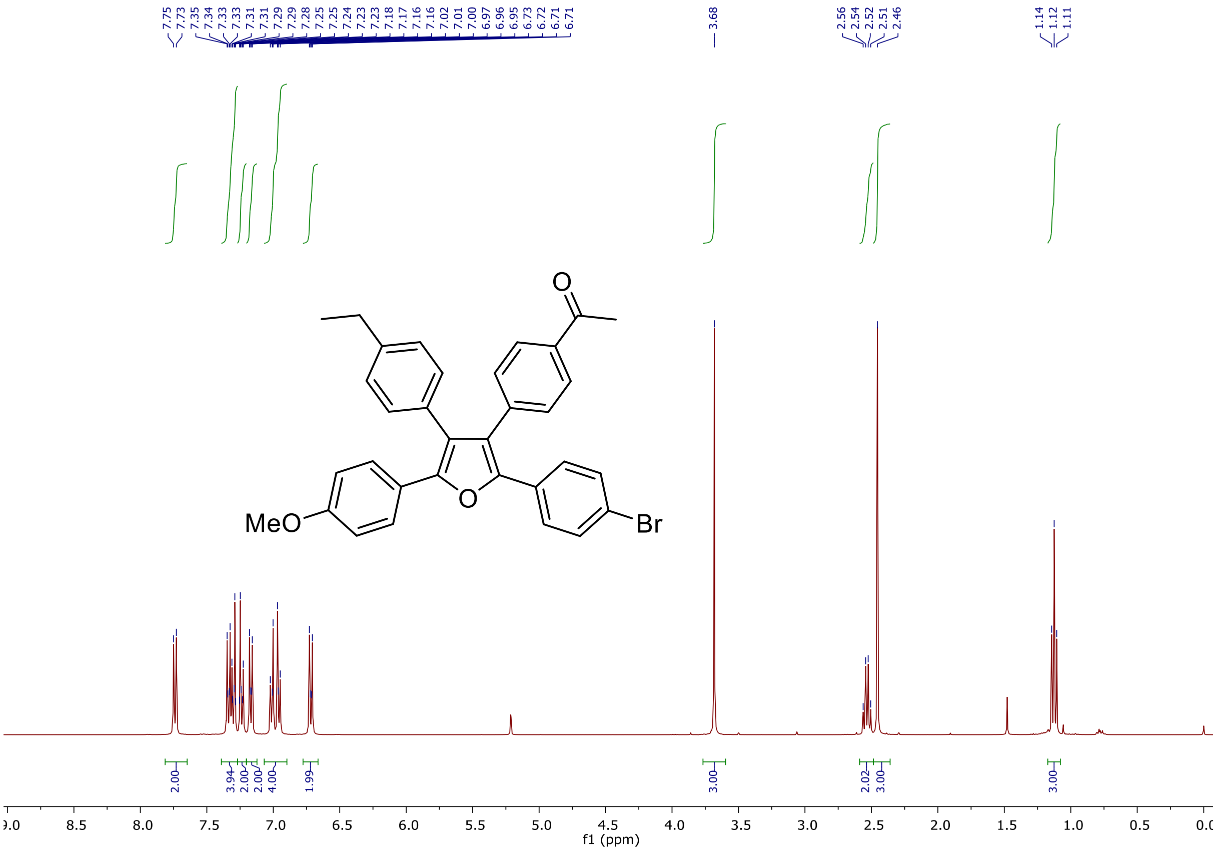

7.75
7.73
7.35
7.34
7.33
7.33
7.31
7.31
7.29
7.29
7.28
7.25
7.25
7.24
7.23
7.23
7.18
7.17
7.16
7.16
7.02
7.01
7.00
6.97
6.96
6.95
6.73
6.72
6.71
6.71
3.68
2.56
2.54
2.52
2.51
2.46
1.14
1.12
1.11
O
MeO
Br
2.00
3.94
2.00
2.00
4.00
1.99
3.00
2.02
3.00
3.00
f1 (ppm)

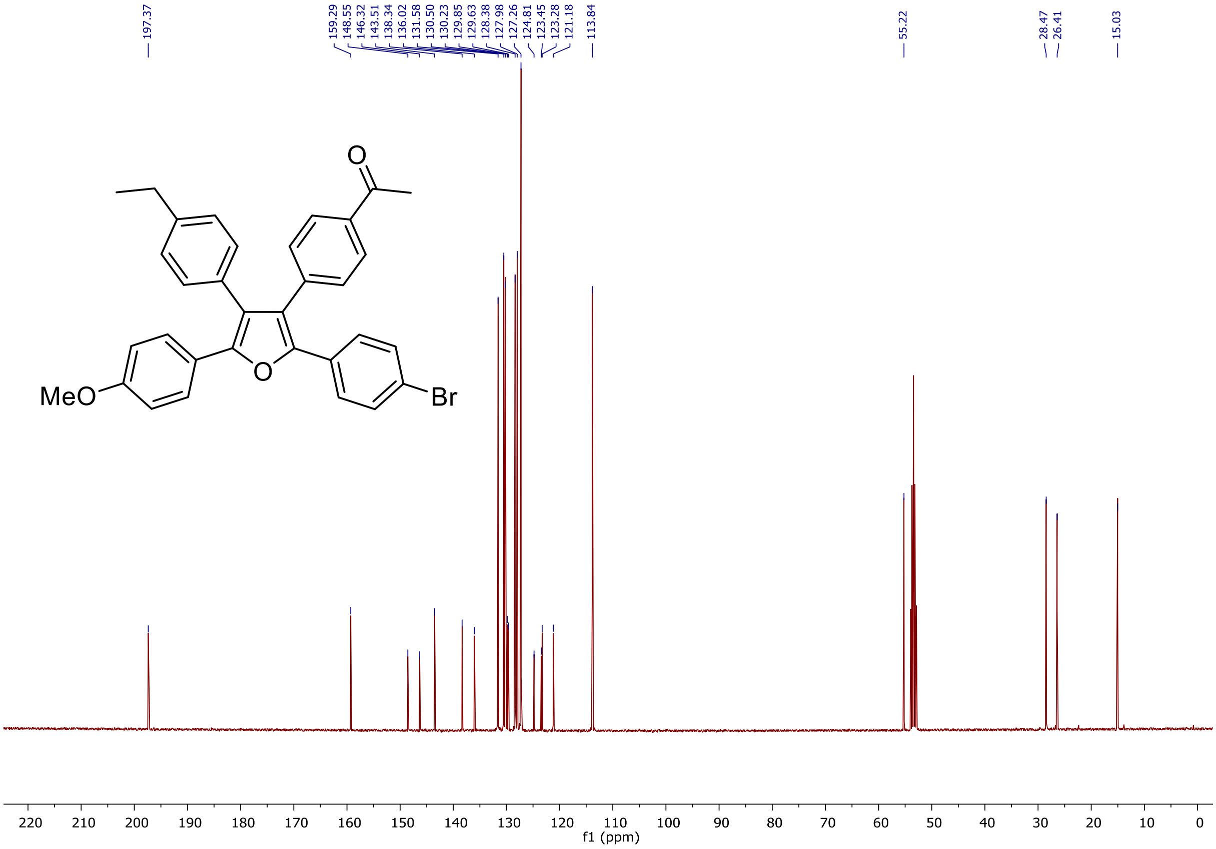

197.37
159.29
148.55
146.32
143.51
138.34
136.02
131.58
130.50
130.23
129.85
129.63
128.38
127.98
127.26
124.81
123.45
123.28
121.18
113.84
55.22
28.47
26.41
15.03
O
MeO
Br
f1 (ppm)

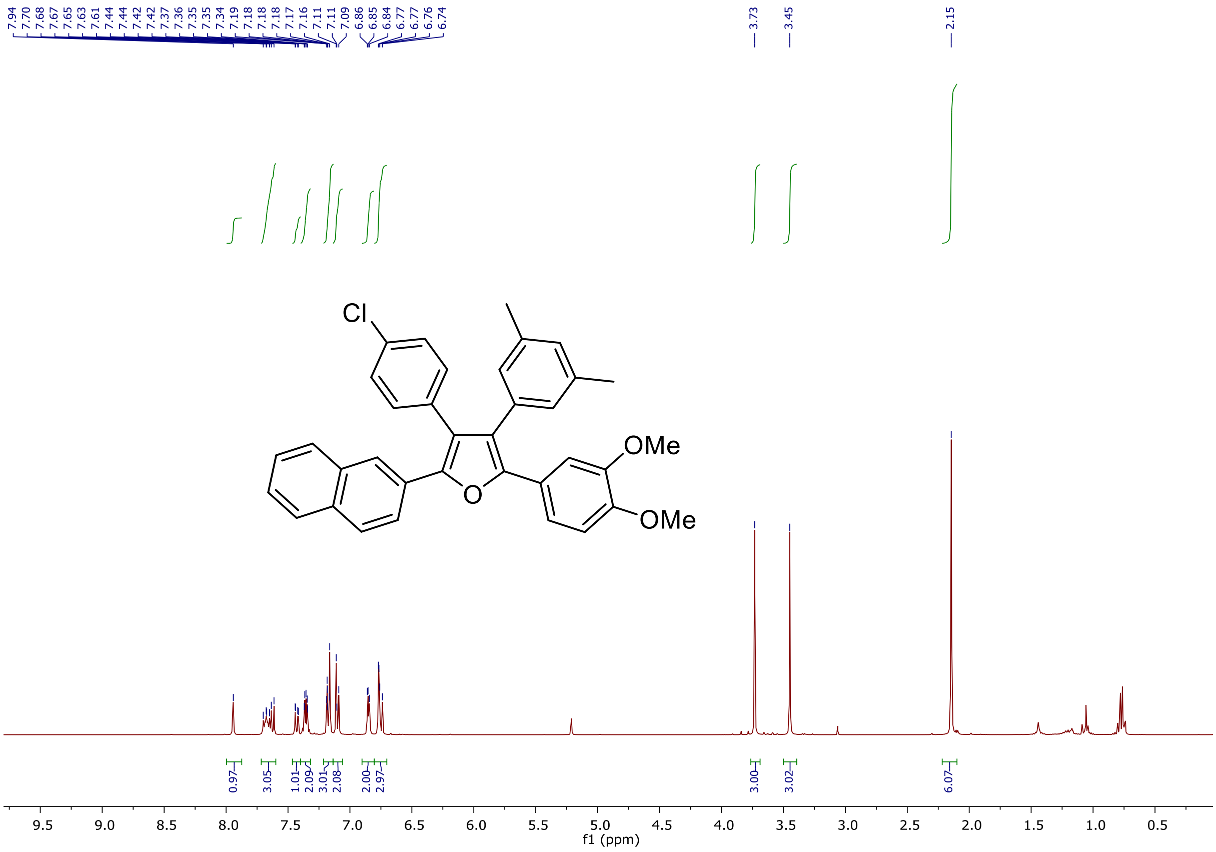
7.94
7.70
7.68
7.67
7.65
7.63
7.61
7.44
7.44
7.42
7.42
7.37
7.36
7.35
7.35
7.34
7.19
7.18
7.18
7.17
7.16
7.11
7.11
7.09
6.86
6.85
6.84
6.77
6.77
6.76
6.74
3.73
3.45
2.15
Cl
OMe
OMe
O
0.97
3.05
1.01
2.09
3.01
2.08
2.00
2.97
3.00
3.02
6.07
f1 (ppm)

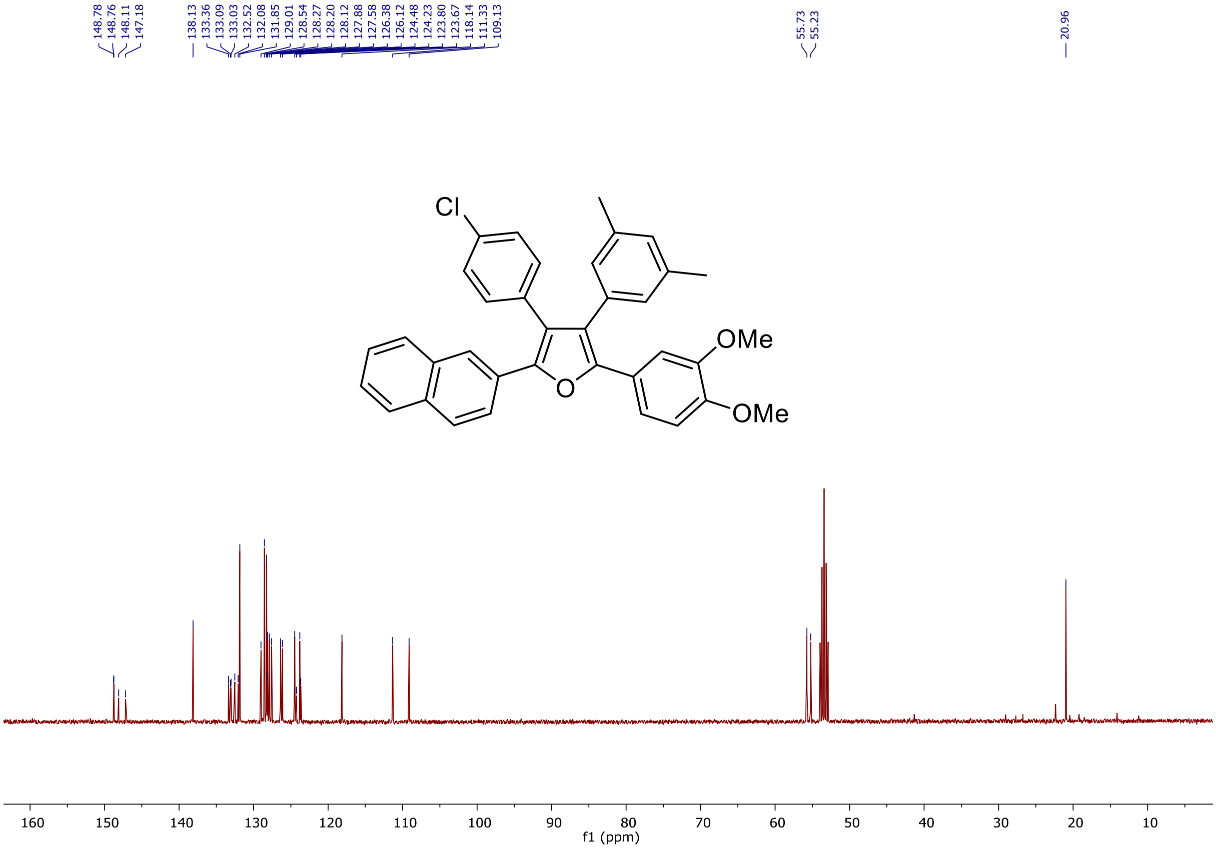
148.78
148.76
148.11
147.18
138.13
133.36
133.09
133.03
132.52
132.08
131.85
129.01
128.54
128.27
128.20
128.12
127.88
127.58
126.38
126.12
124.48
124.23
123.80
123.67
118.14
111.33
109.13
55.73
55.23
20.96
Cl
OMe
OMe
O
f1 (ppm)

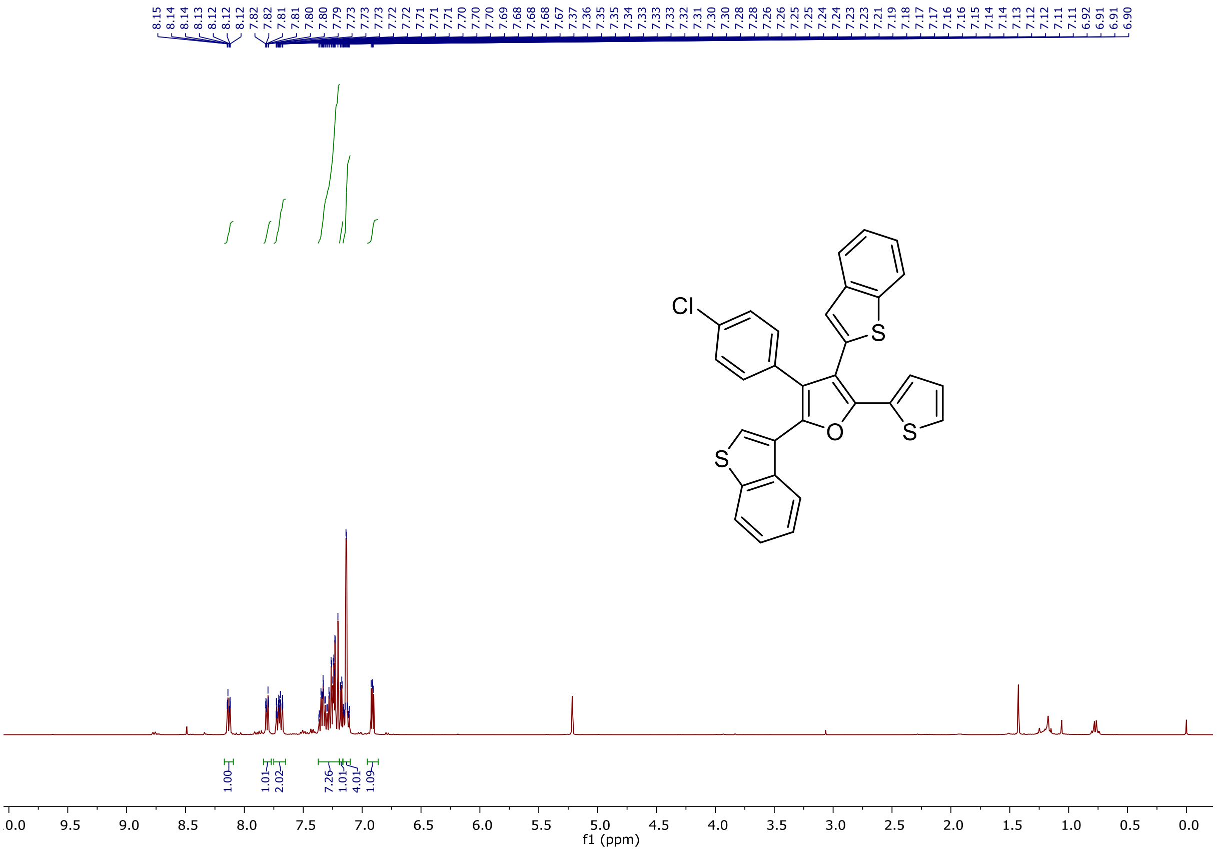
8.15 8.14 8.14 8.13 8.12 8.12 8.12 7.82 7.82 7.81 7.81 7.80 7.80 7.79 7.73 7.73 7.73 7.72 7.72 7.71 7.71 7.71 7.70 7.70 7.69 7.68 7.68 7.67 7.37 7.36 7.35 7.35 7.34 7.33 7.33 7.33 7.32 7.31 7.30 7.30 7.28 7.28 7.26 7.26 7.25 7.25 7.24 7.24 7.23 7.23 7.21 7.19 7.18 7.17 7.17 7.16 7.15 7.14 7.14 7.13 7.12 7.12 7.11 7.11 6.92 6.91 6.91 6.90
Cl
S
O
S
S
1.00
1.01
2.02
7.26
1.01
4.01
1.09
0.0 9.5 9.0 8.5 8.0 7.5 7.0 6.5 6.0 5.5 5.0 4.5 4.0 3.5 3.0 2.5 2.0 1.5 1.0 0.5 0.0
f1 (ppm)

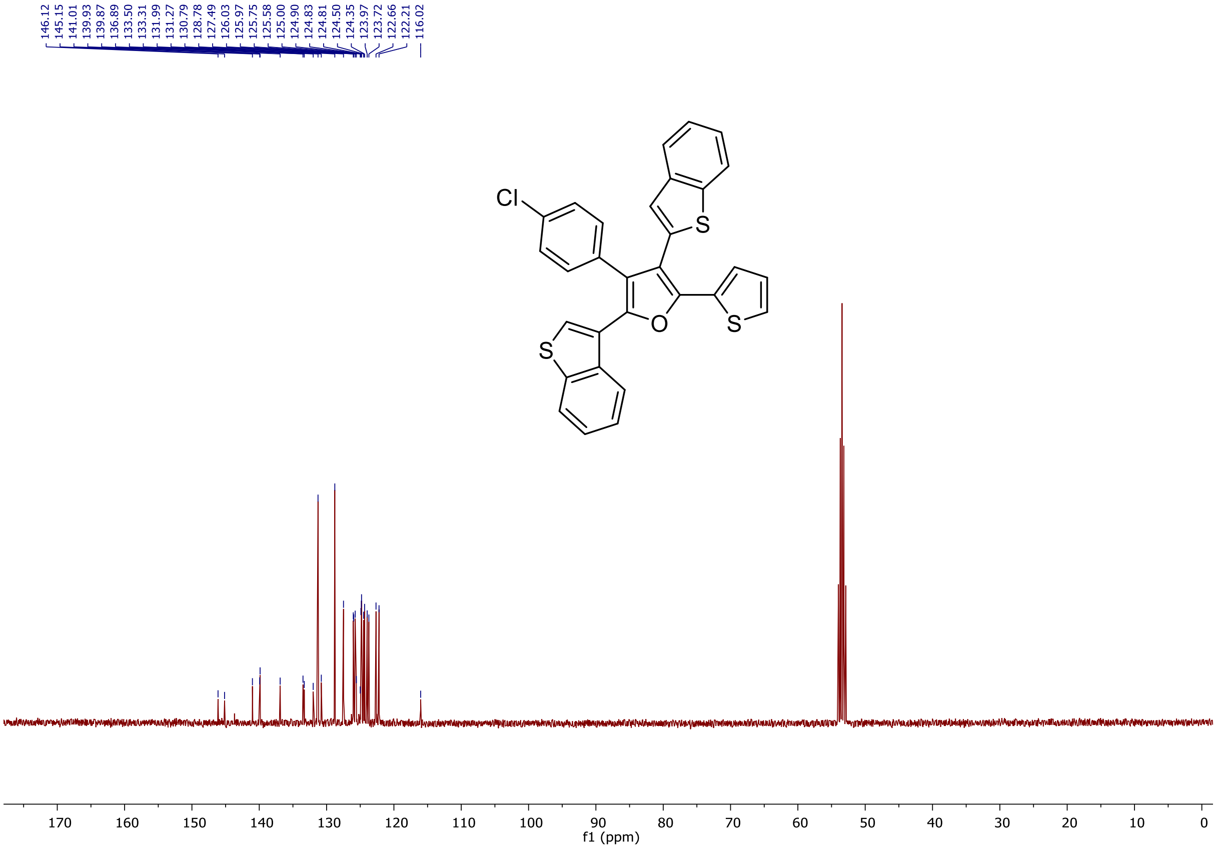
146.12 145.15 141.01 139.93 139.87 136.89 133.50 133.31 131.99 131.27 130.79 128.78 127.49 126.03 125.97 125.75 125.58 125.00 124.90 124.83 124.81 124.50 124.35 123.97 123.72 122.66 122.21 116.02
Cl
S
O
S
S
170 160 150 140 130 120 110 100 90 80 70 60 50 40 30 20 10 0
f1 (ppm)

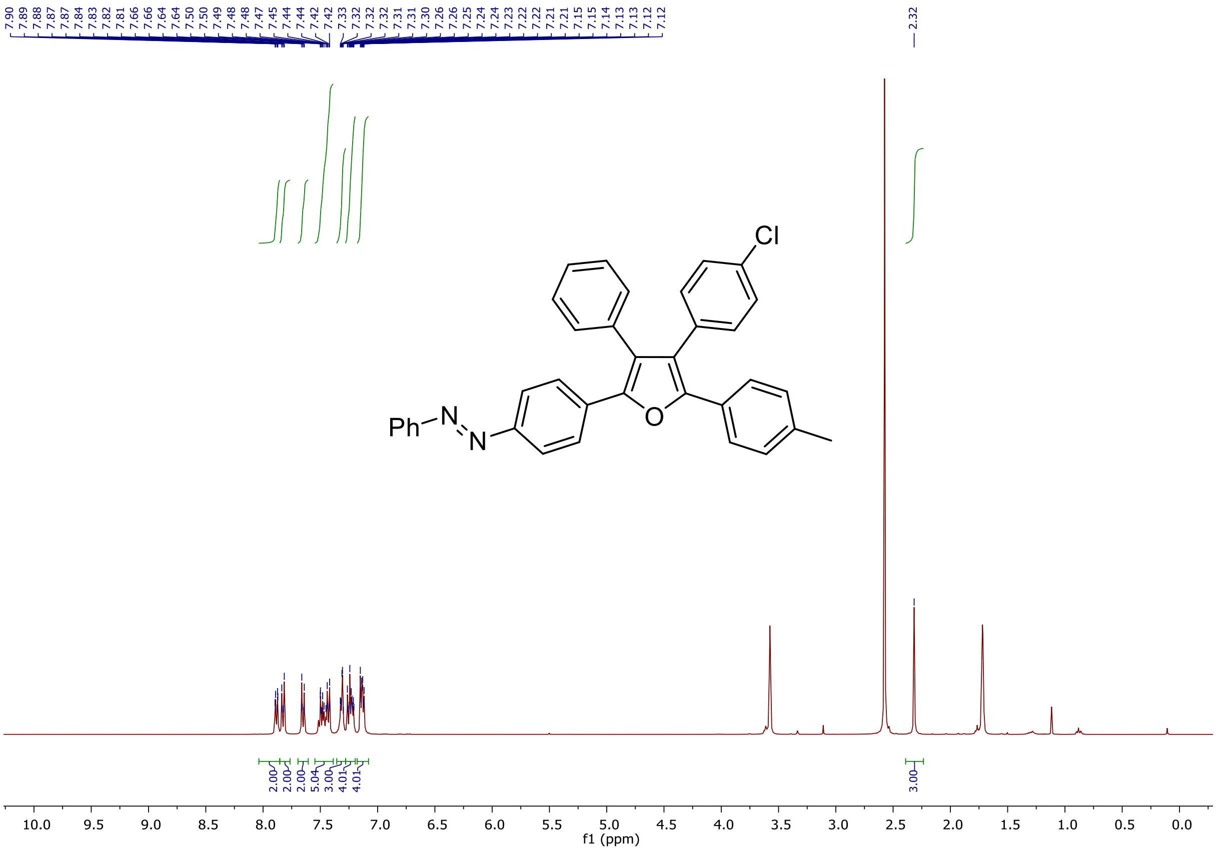
7.90
7.89
7.88
7.87
7.87
7.84
7.83
7.82
7.81
7.66
7.66
7.64
7.64
7.50
7.50
7.49
7.48
7.48
7.47
7.45
7.44
7.44
7.42
7.42
7.33
7.32
7.32
7.32
7.31
7.30
7.26
7.26
7.25
7.24
7.24
7.23
7.22
7.22
7.21
7.21
7.15
7.15
7.14
7.13
7.13
7.12
7.12
2.32
Cl
Ph
N
N
O
2.00
2.00
2.00
5.04
3.00
4.01
4.01
3.00
f1 (ppm)

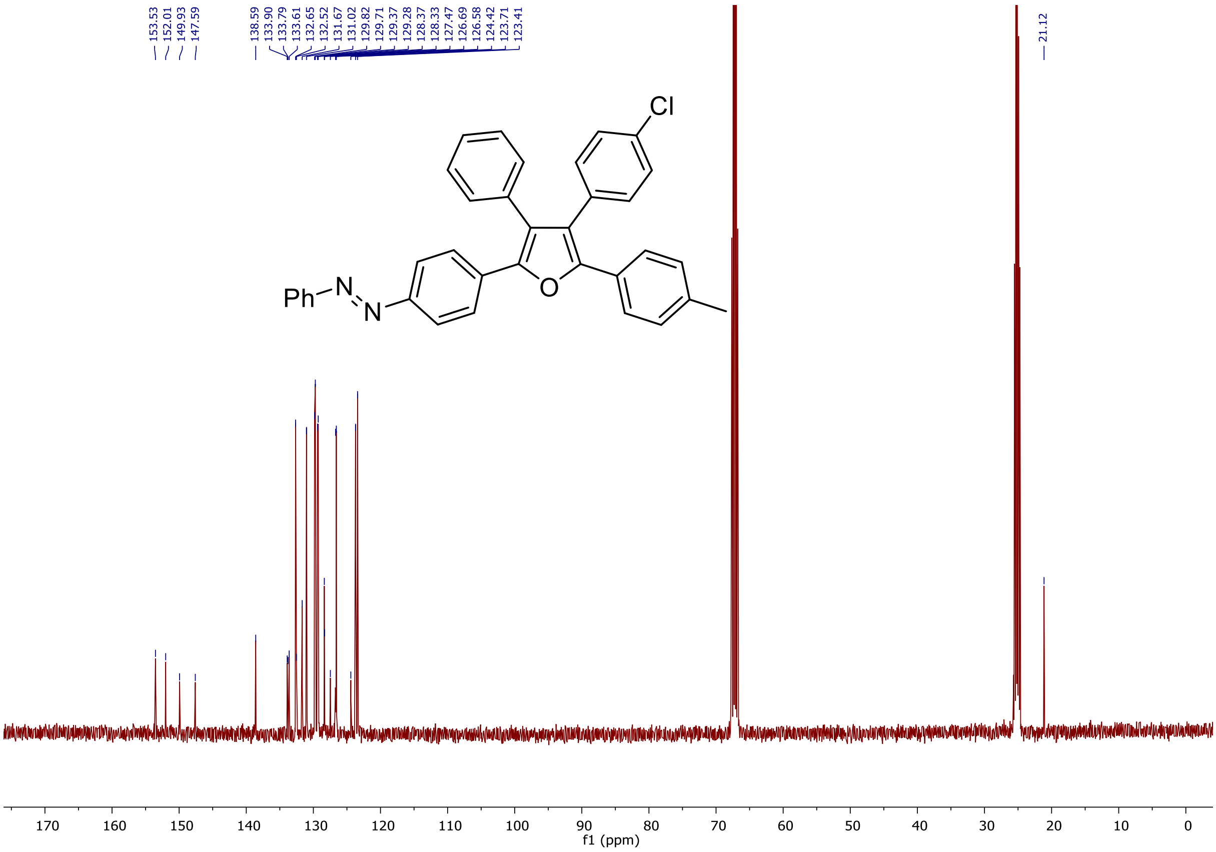
153.53
152.01
149.93
147.59
138.59
133.90
133.79
133.61
132.65
132.52
131.67
131.02
129.82
129.71
129.37
129.28
128.37
128.33
127.47
126.69
126.58
124.42
123.71
123.41
21.12
Cl
Ph
N
N
O
f1 (ppm)

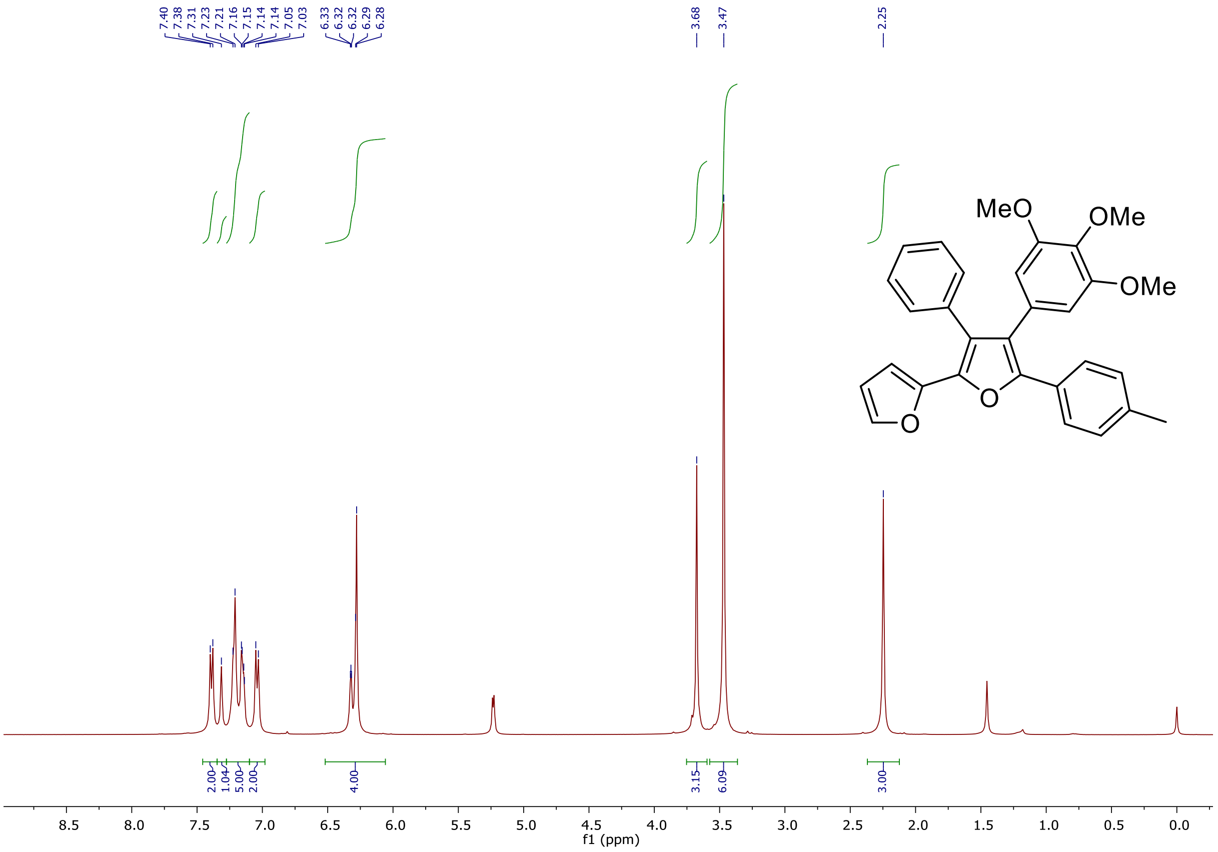
7.40 7.38 7.31 7.23 7.21 7.16 7.15 7.14 7.14 7.05 7.03 6.33 6.32 6.32 6.29 6.28
3.68
3.47
2.25
2.00 1.04 5.00 2.00
4.00
3.15 6.09
3.00
MeO
OMe
OMe
O
O
f1 (ppm)

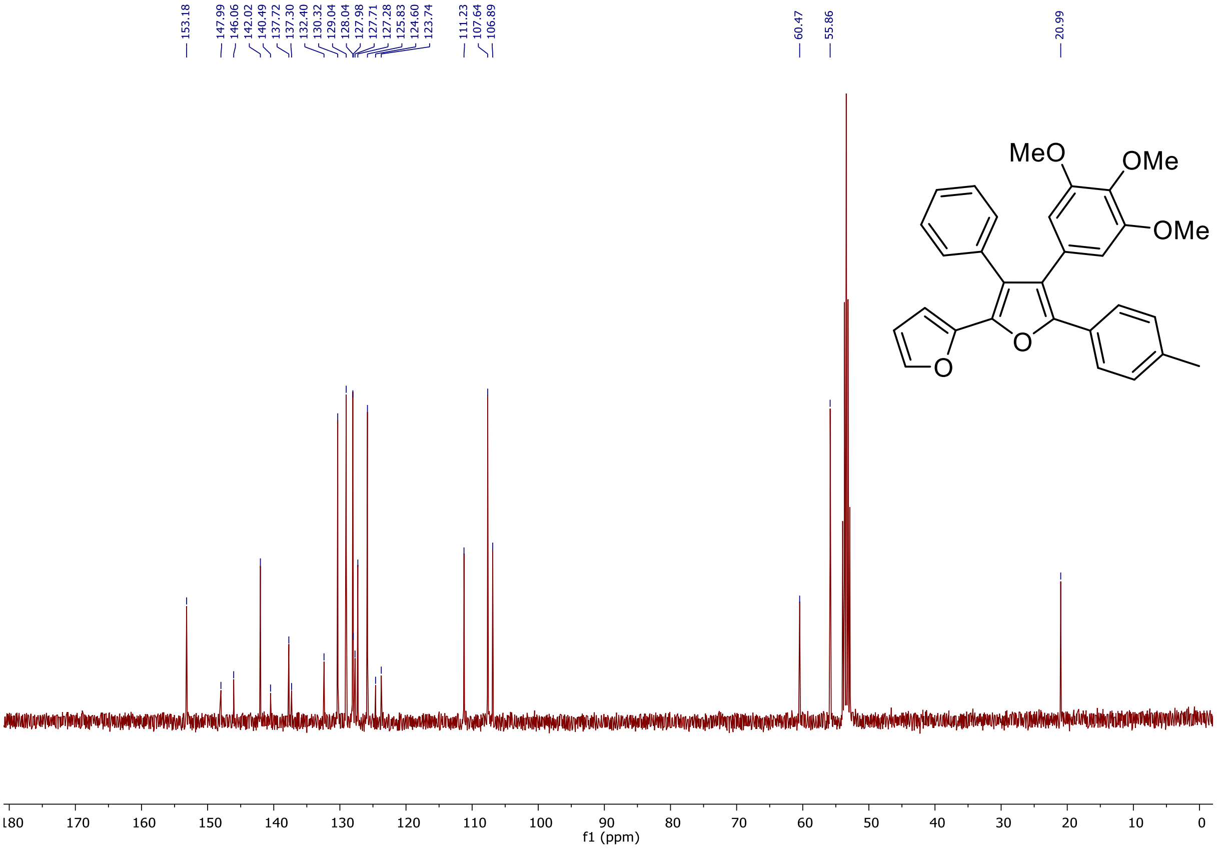
153.18
147.99 146.06 142.02 140.49 137.72 137.30 132.40 130.32 129.04 128.04 127.98 127.71 127.28 125.83 124.60 123.74
111.23 107.64 106.89
60.47
55.86
20.99
MeO
OMe
OMe
O
O
f1 (ppm)

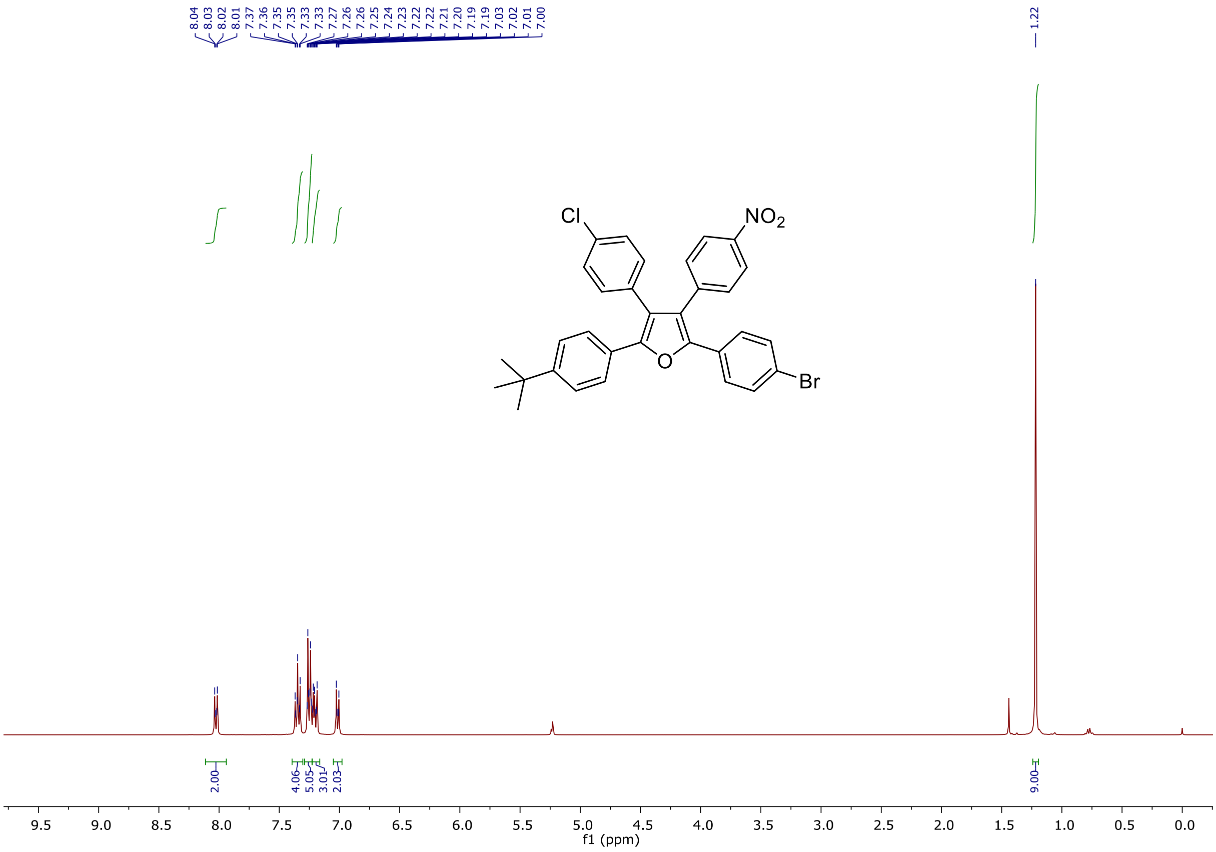
8.04
8.03
8.02
8.01
7.37
7.36
7.35
7.33
7.33
7.27
7.26
7.25
7.24
7.23
7.22
7.22
7.21
7.20
7.19
7.19
7.03
7.02
7.01
7.00
1.22
Cl
NO2
O
Br
2.00
4.06
5.05
3.01
2.03
9.00
f1 (ppm)

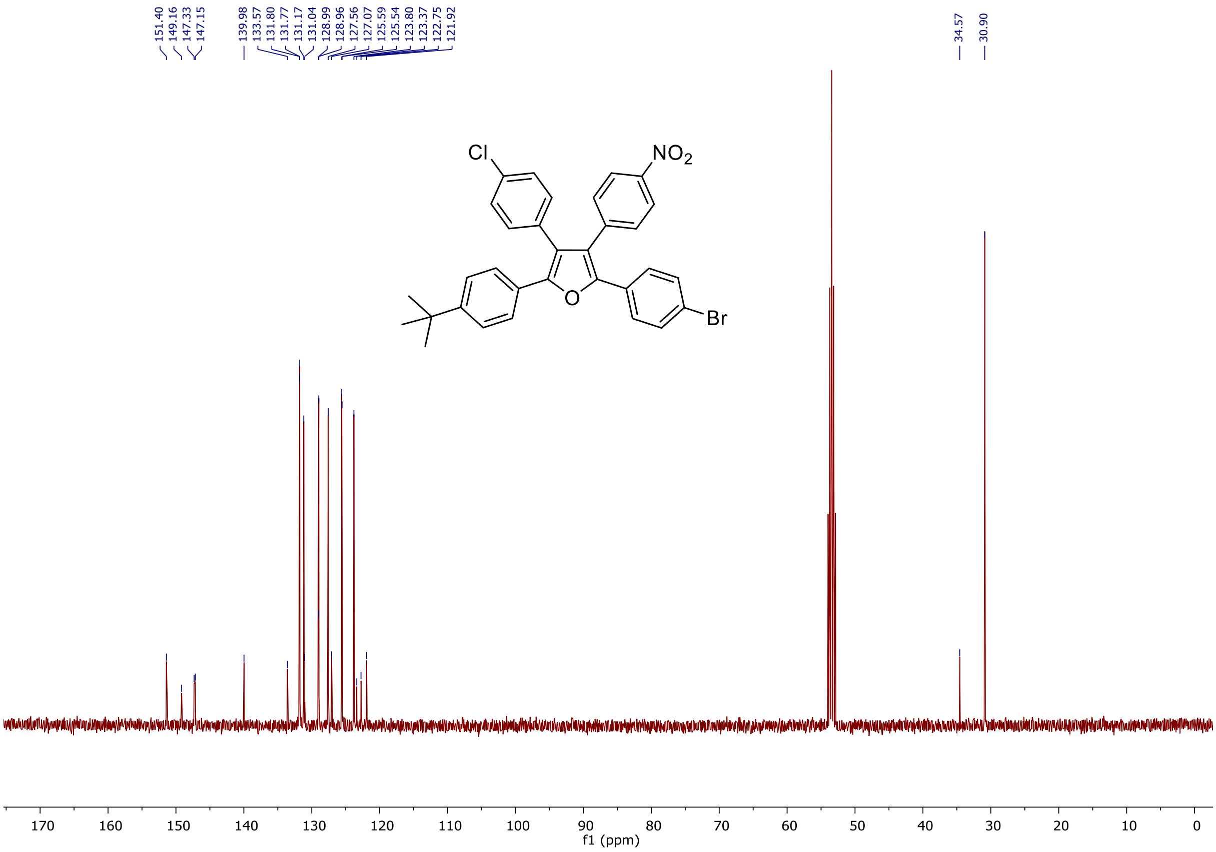
151.40
149.16
147.33
147.15
139.98
133.57
131.80
131.77
131.17
131.04
128.99
128.96
127.56
127.07
125.59
125.54
123.80
123.37
122.75
121.92
34.57
30.90
Cl
NO2
O
Br
f1 (ppm)

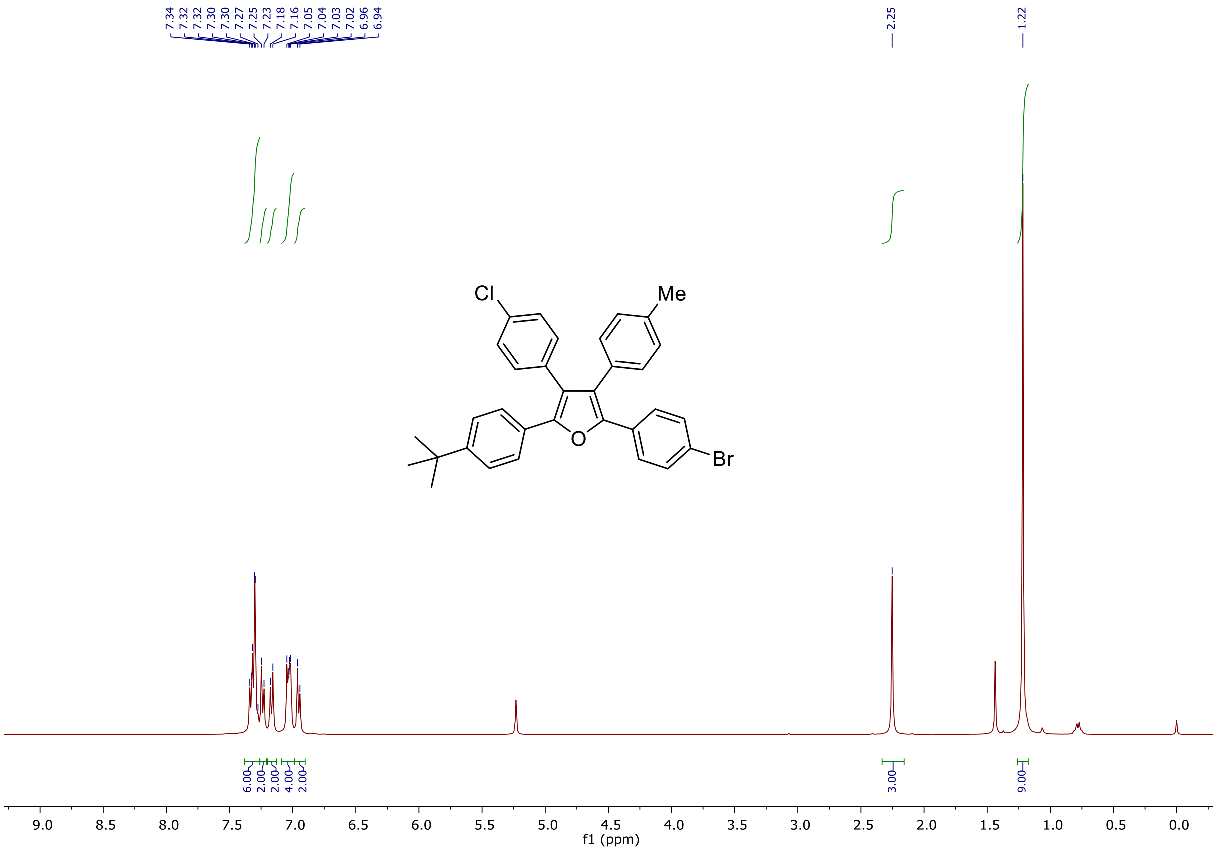
7.34
7.32
7.32
7.30
7.30
7.27
7.25
7.23
7.18
7.16
7.05
7.04
7.03
7.02
6.96
6.94
2.25
1.22
Cl
Me
O
Br
6.00
2.00
2.00
4.00
2.00
3.00
9.00
9.0
8.5
8.0
7.5
7.0
6.5
6.0
5.5
5.0
4.5
4.0
3.5
3.0
2.5
2.0
1.5
1.0
0.5
0.0
f1 (ppm)

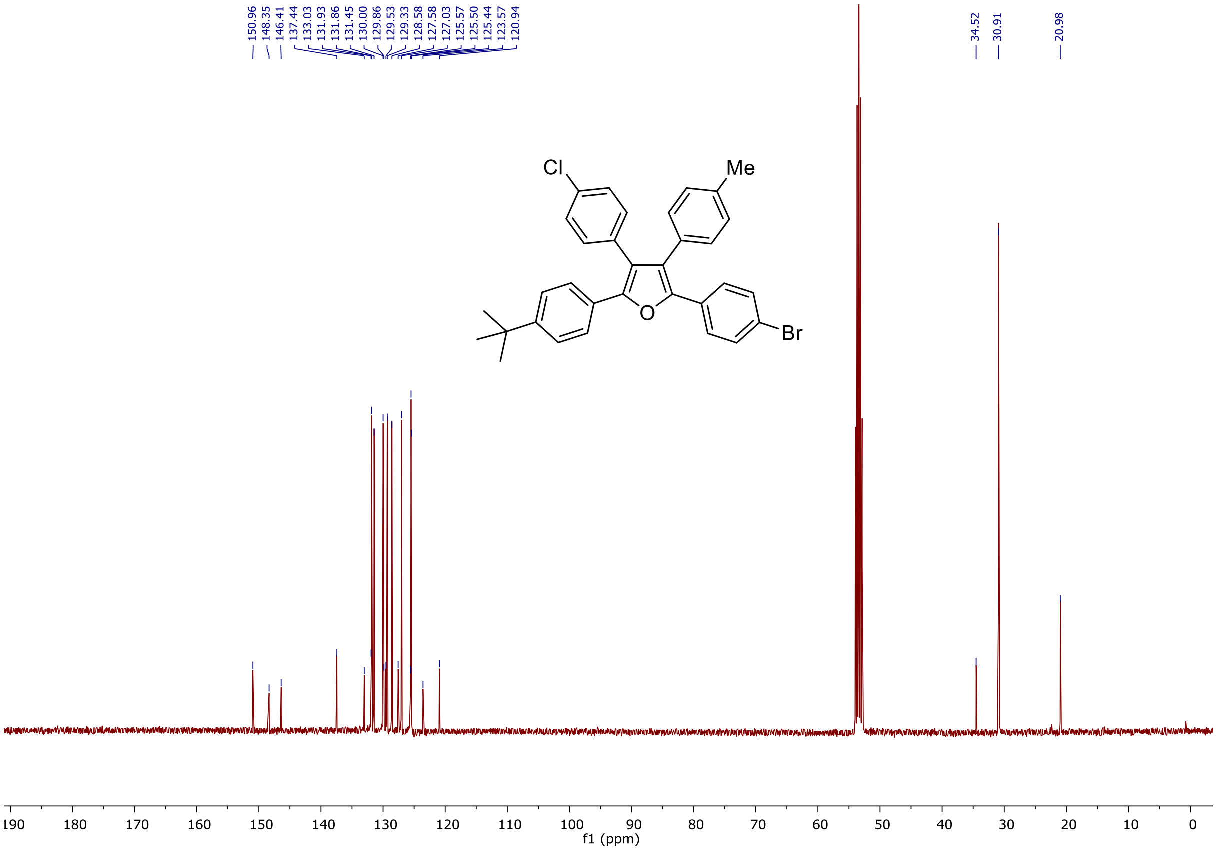
150.96
148.35
146.41
137.44
133.03
131.93
131.86
131.45
130.00
129.86
129.53
129.33
128.58
127.58
127.03
125.57
125.50
125.44
123.57
120.94
34.52
30.91
20.98
Cl
Me
O
Br
190
180
170
160
150
140
130
120
110
100
90
80
70
60
50
40
30
20
10
0
f1 (ppm)

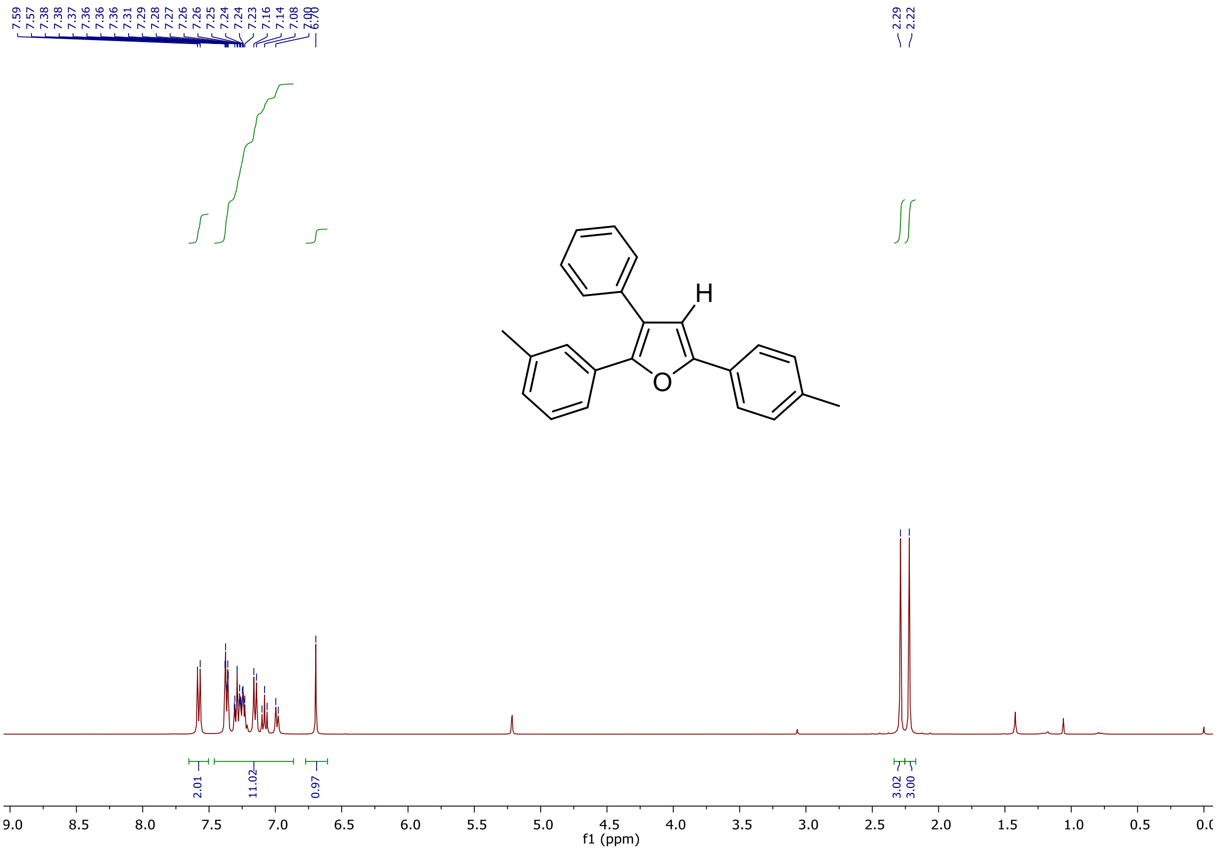

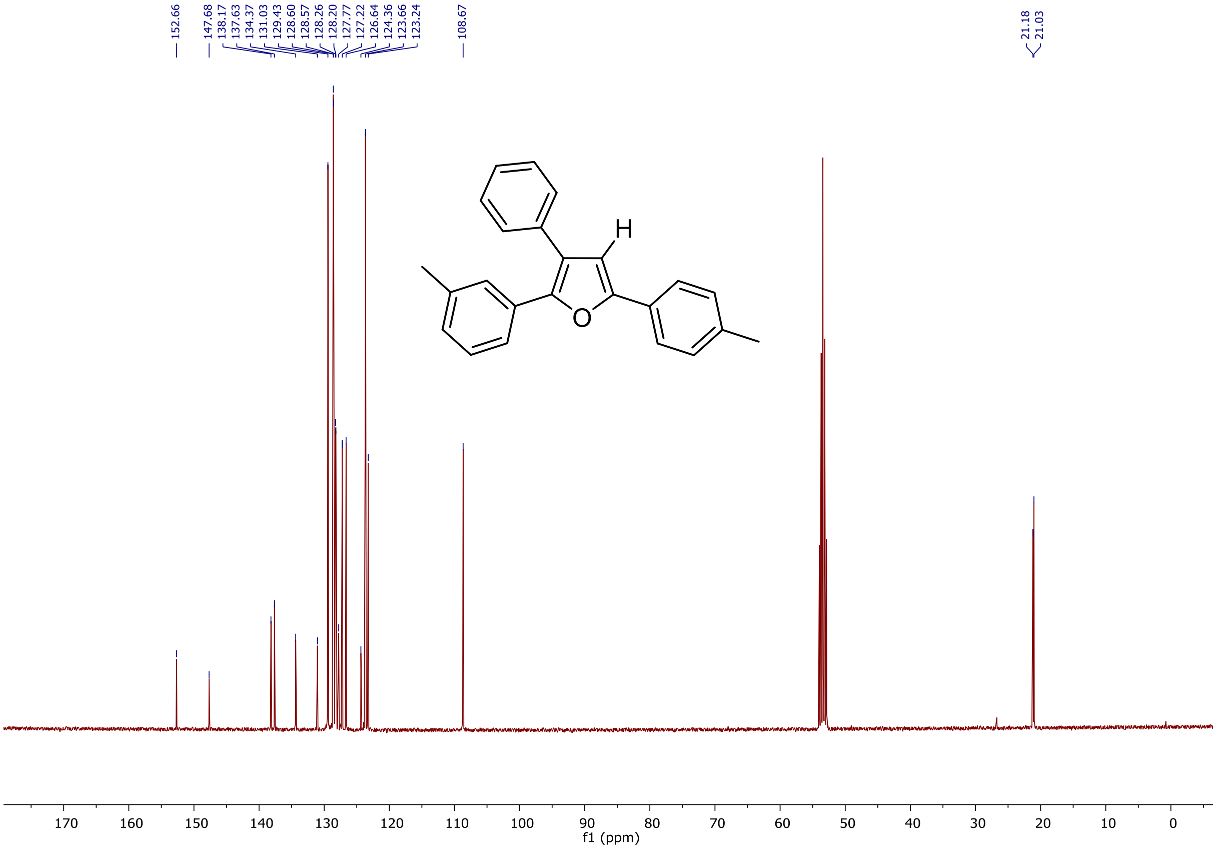

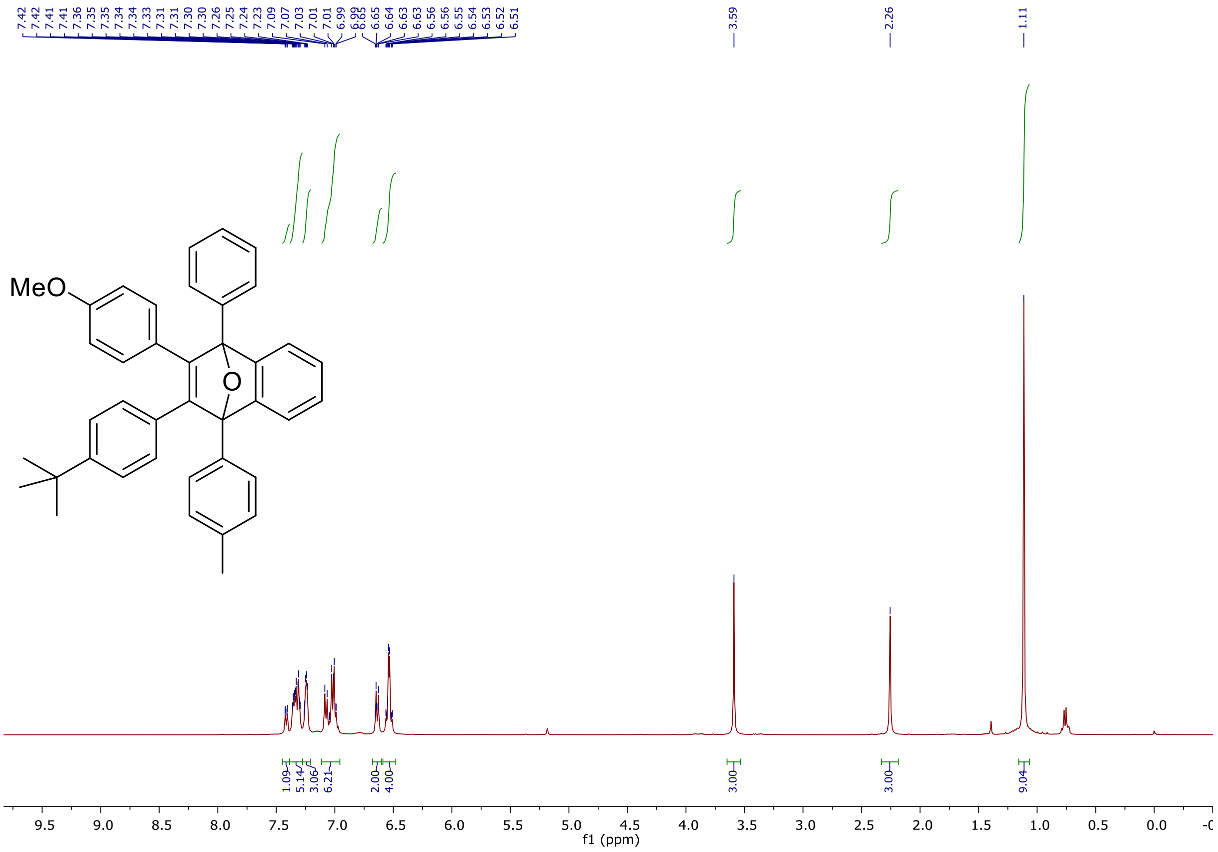

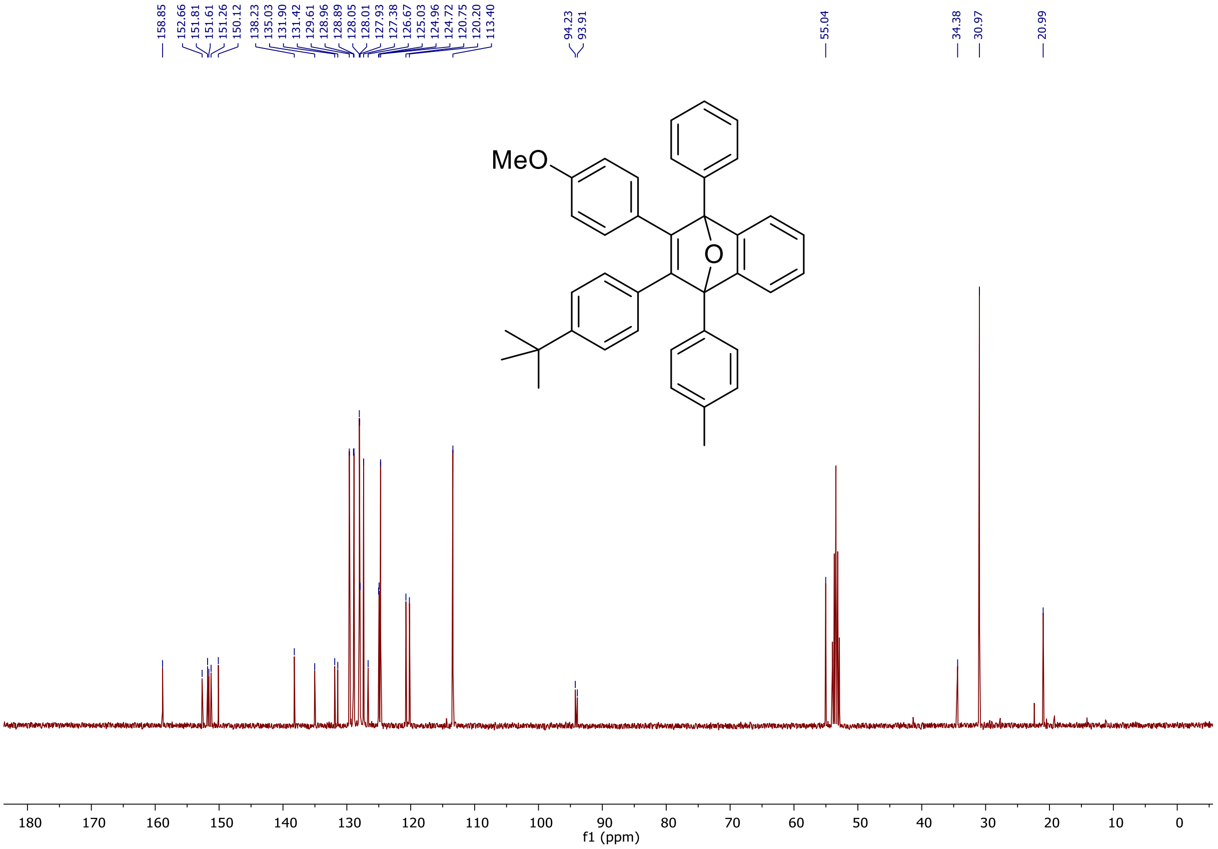

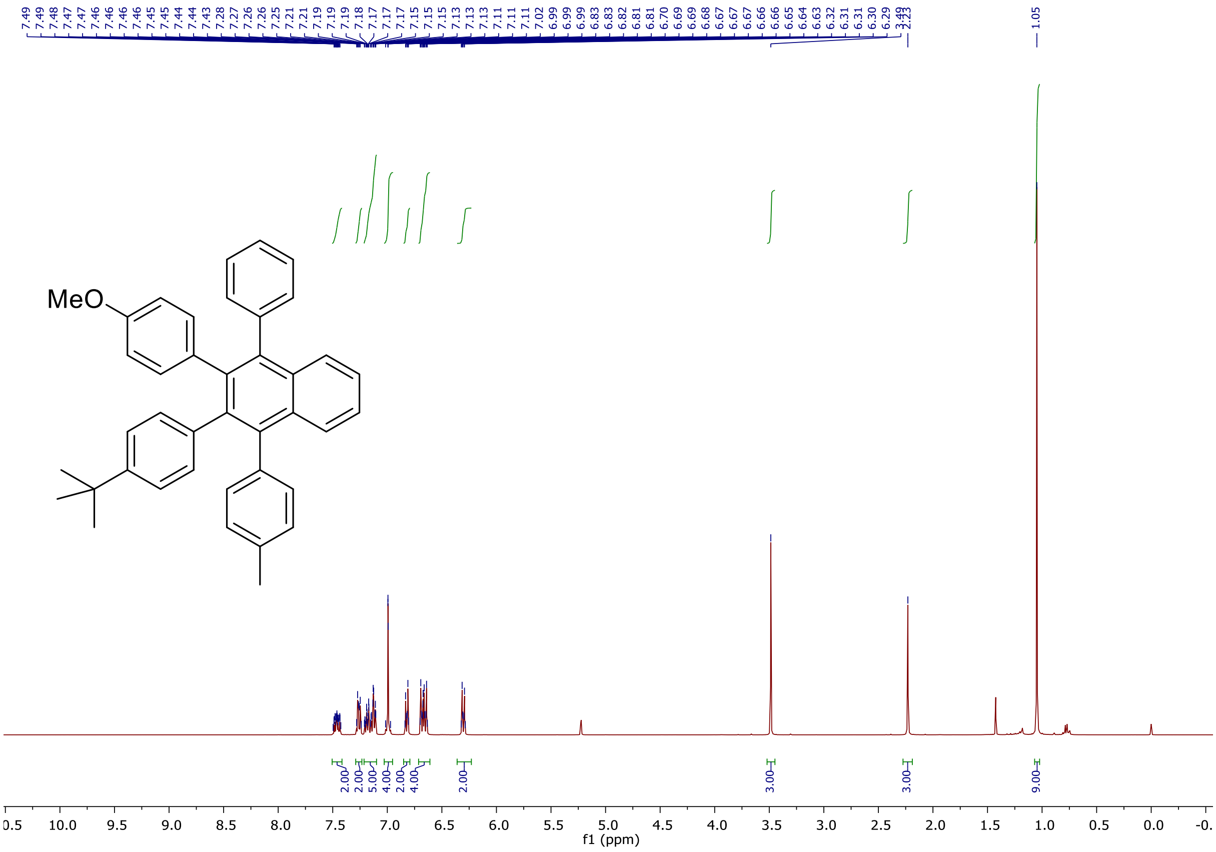

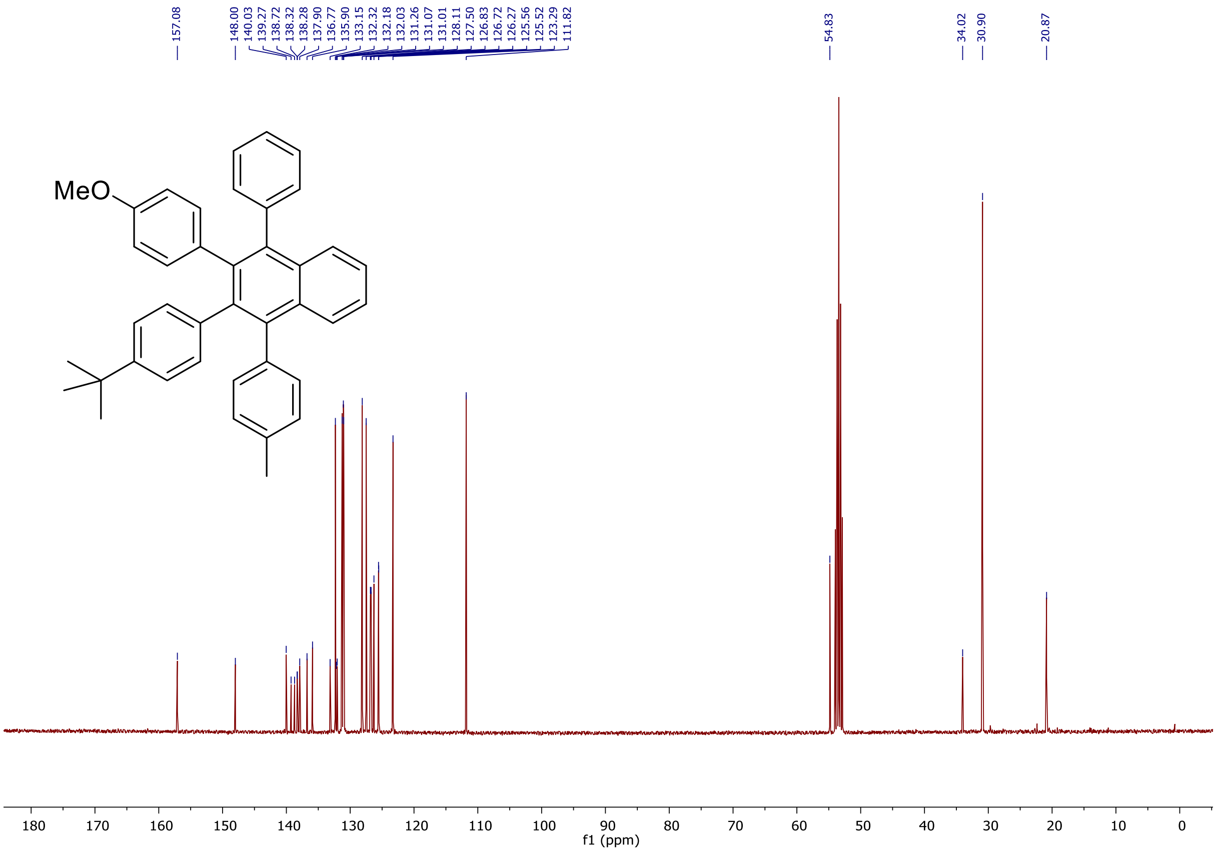

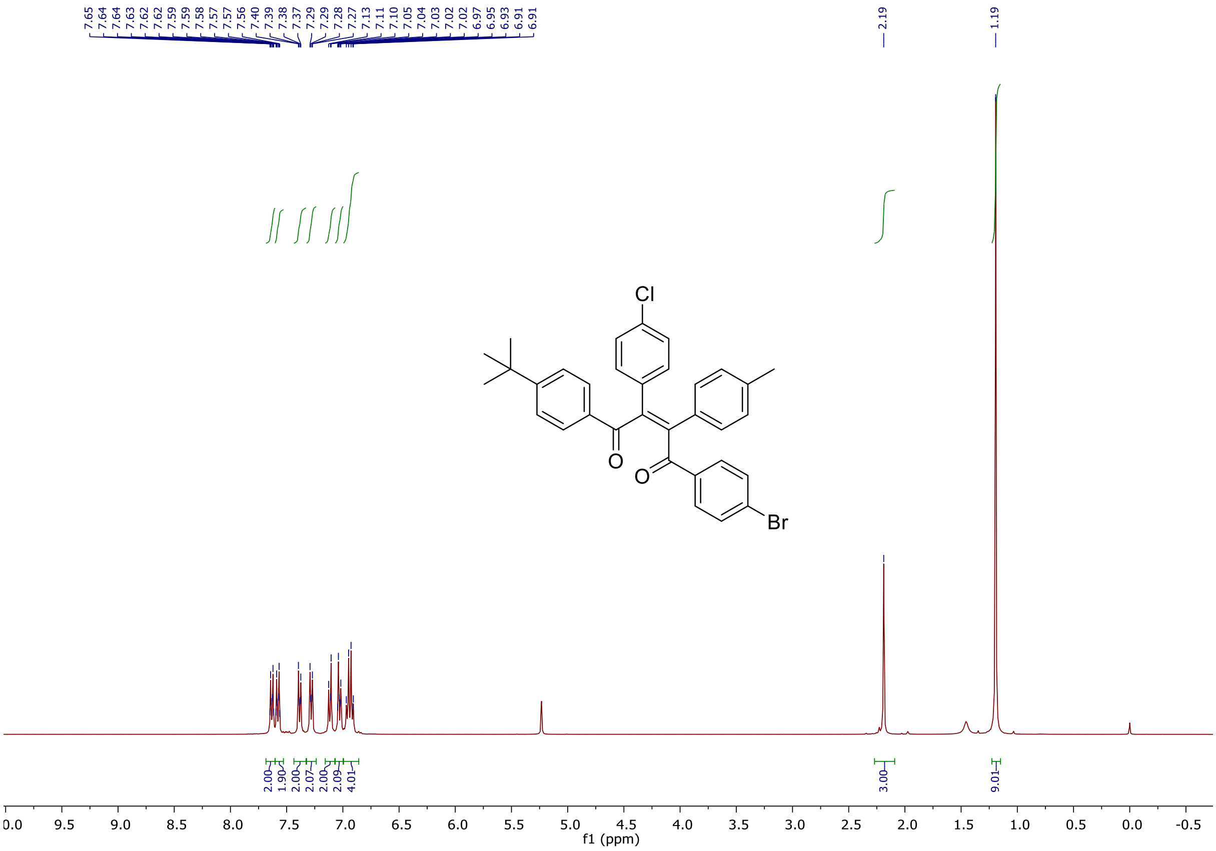
7.65 7.64 7.64 7.63 7.62 7.62 7.59 7.59 7.58 7.57 7.57 7.56 7.40 7.39 7.38 7.37 7.29 7.29 7.28 7.27 7.13 7.11 7.10 7.05 7.04 7.03 7.02 7.02 6.97 6.95 6.93 6.91 6.91
2.19
1.19
Cl
O
O
Br
2.00 1.90 2.00 2.07 2.00 2.09 4.01
3.00
9.01
f1 (ppm)

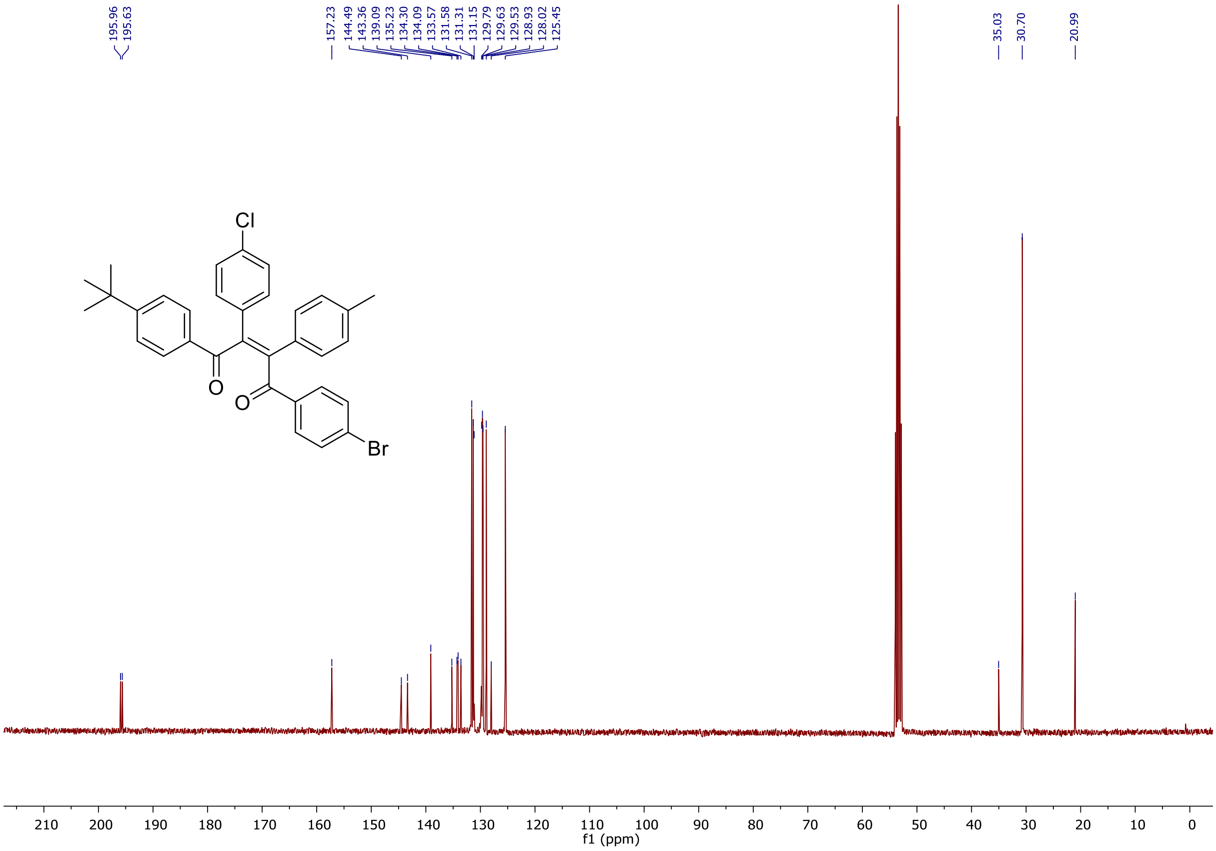
195.96 195.63
157.23 144.49 143.36 139.09 135.23 134.30 134.09 133.57 131.58 131.31 131.15 129.79 129.63 129.53 128.93 128.02 125.45
35.03
30.70
20.99
Cl
O
O
Br
f1 (ppm)

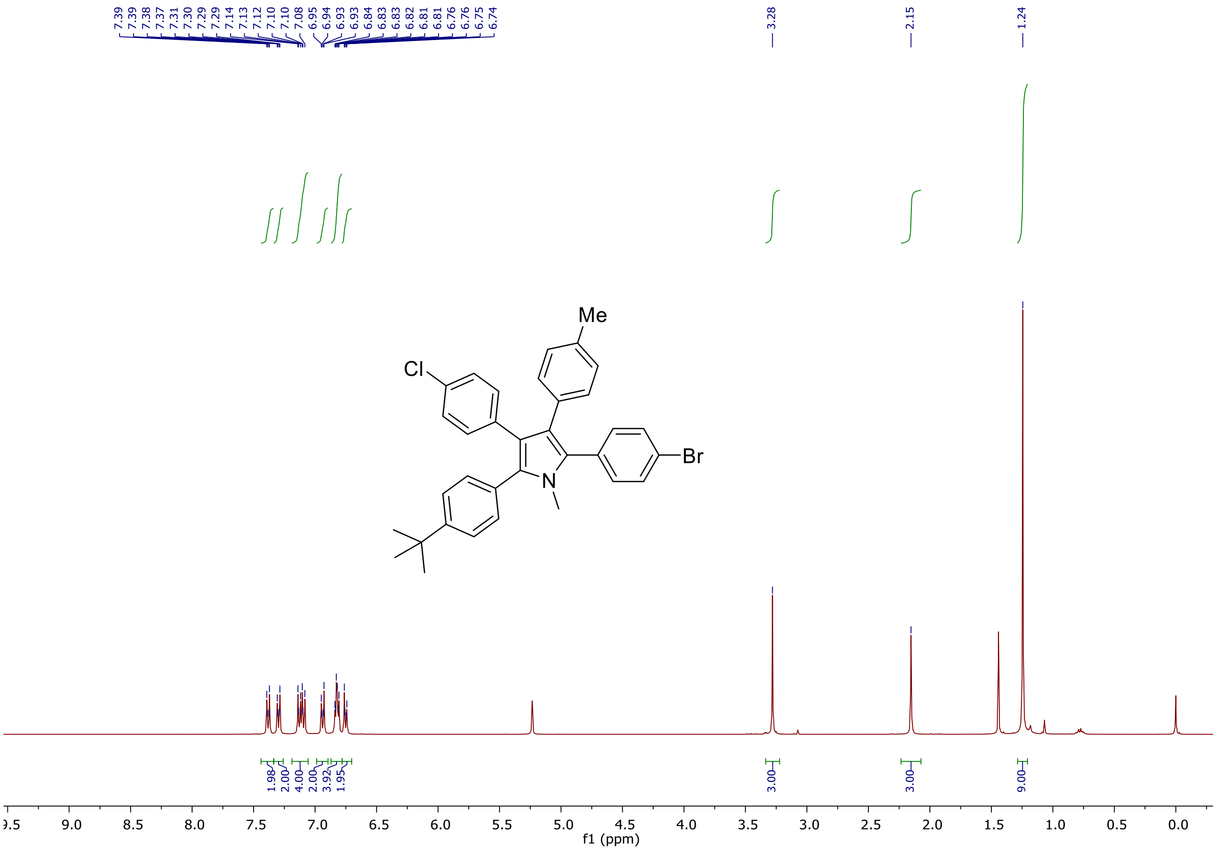

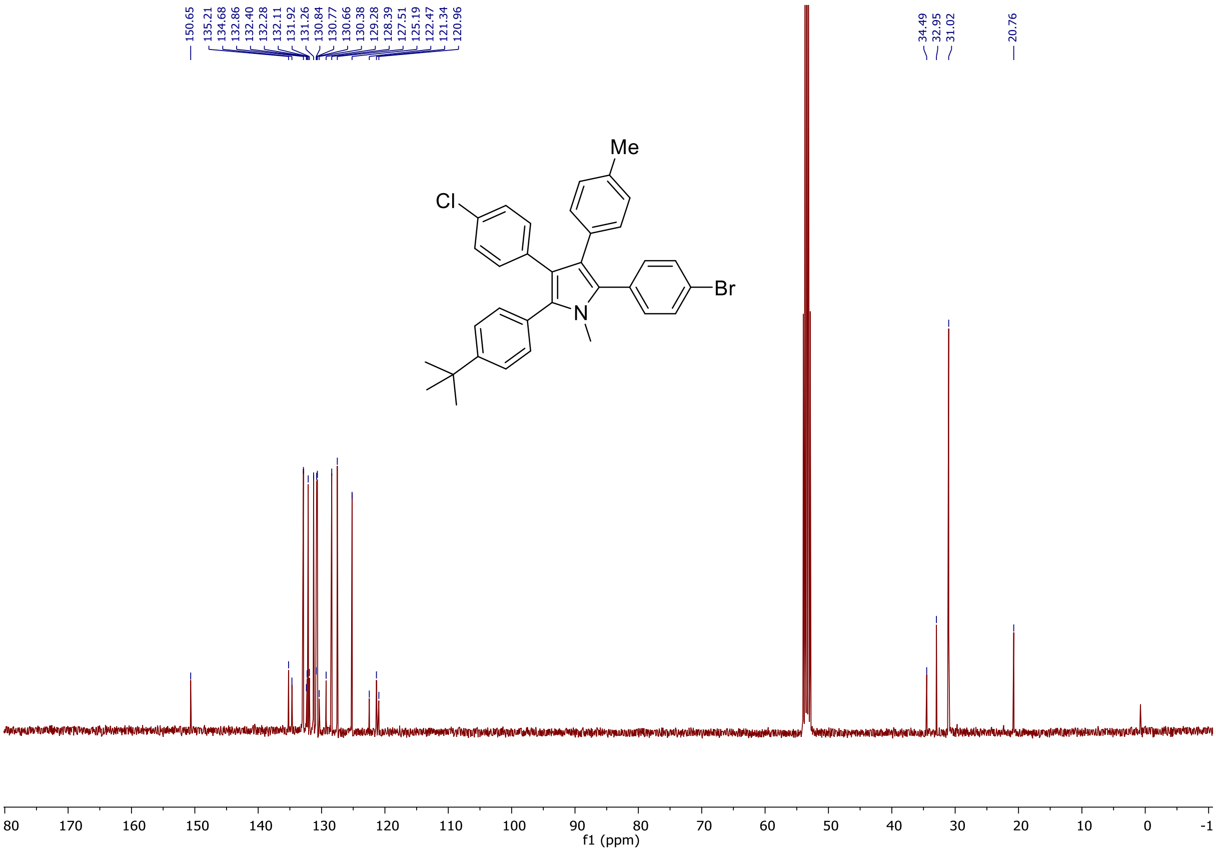

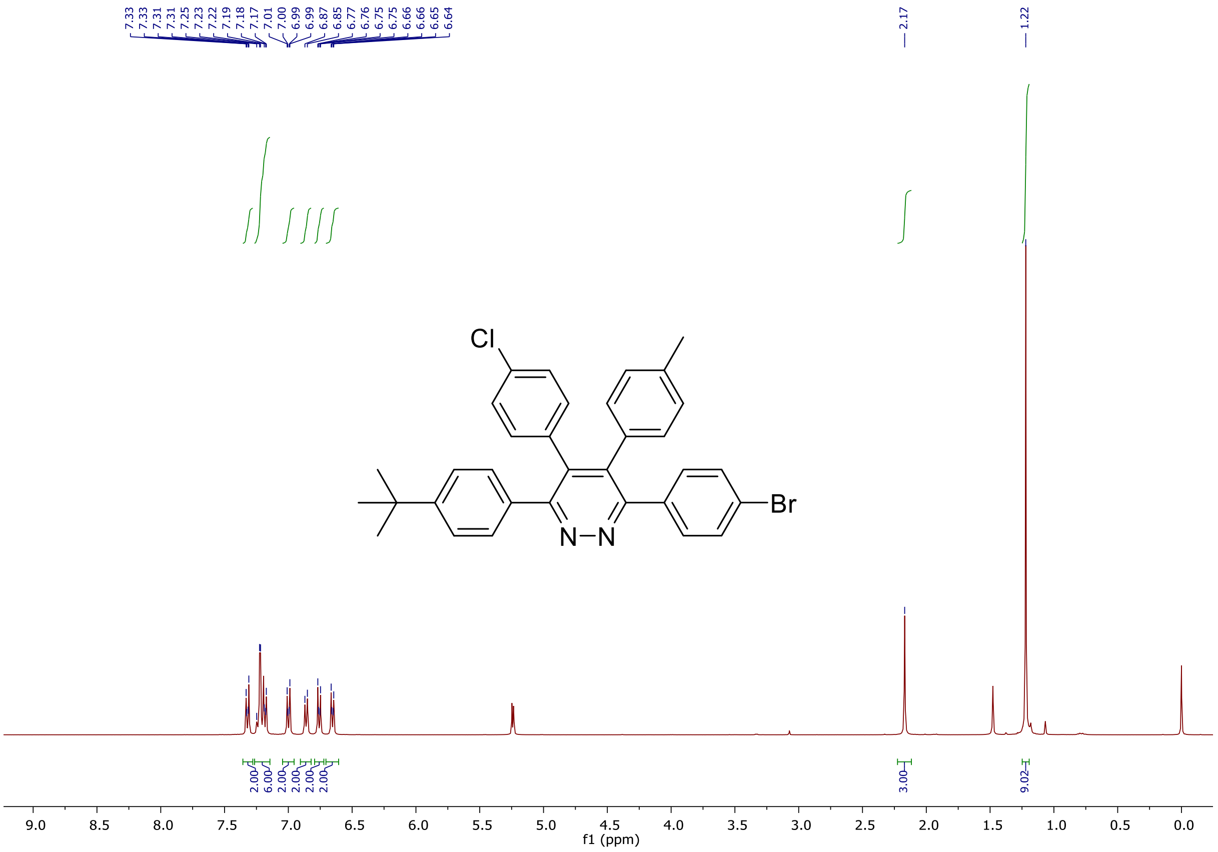
7.33 7.33 7.31 7.31 7.25 7.23 7.22 7.19 7.18 7.17 7.01 7.00 6.99 6.99 6.87 6.85 6.77 6.76 6.75 6.75 6.66 6.66 6.65 6.64
2.17
1.22
Cl
Br
N–N
2.00 6.00 2.00 2.00 2.00 2.00
3.00
9.02
f1 (ppm)

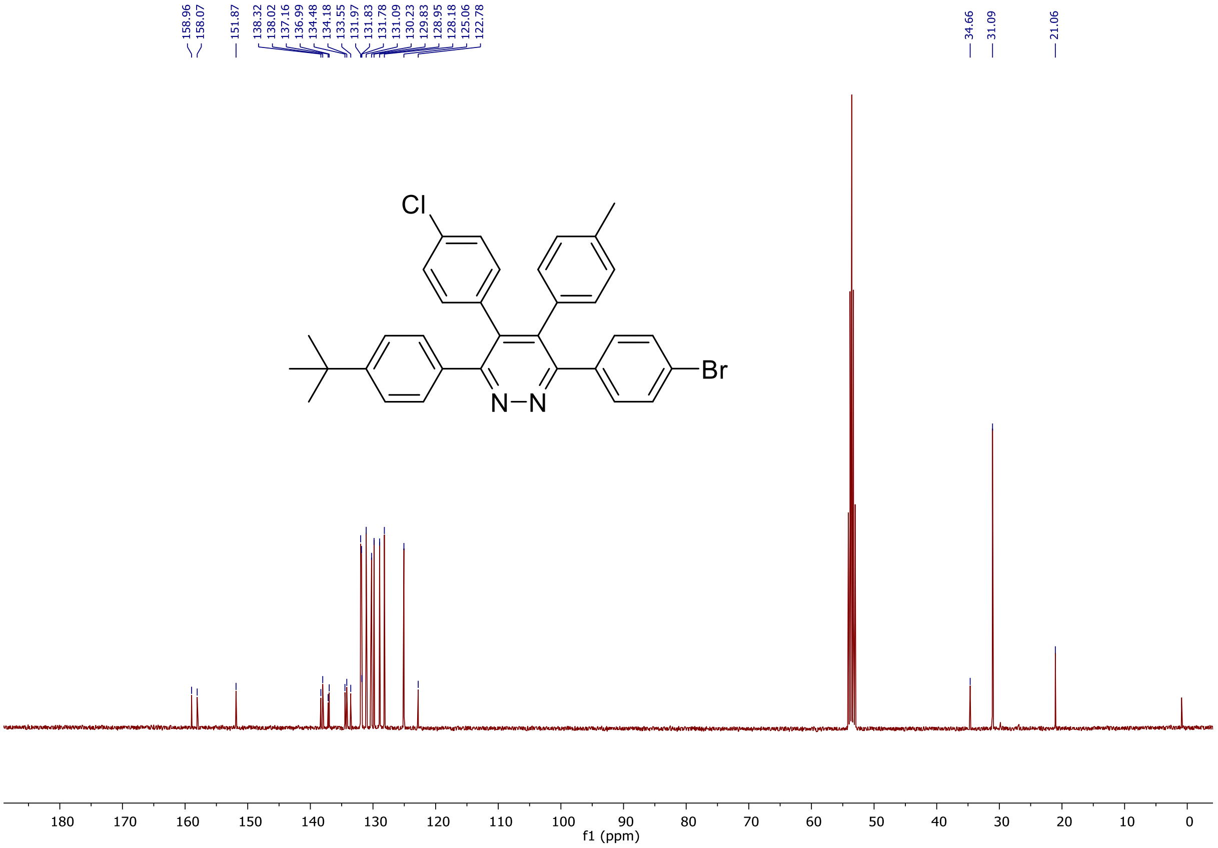
158.96 158.07
151.87
138.32 138.02 137.16 136.99 134.48 134.18 133.55 131.97 131.83 131.78 131.09 130.23 129.83 128.95 128.18 125.06 122.78
34.66
31.09
21.06
Cl
Br
N–N
f1 (ppm)
